# Venom Down Under: Dynamic Evolution of Australian Elapid Snake Toxins

**DOI:** 10.3390/toxins5122621

**Published:** 2013-12-18

**Authors:** Timothy N. W. Jackson, Kartik Sunagar, Eivind A. B. Undheim, Ivan Koludarov, Angelo H. C. Chan, Kate Sanders, Syed A. Ali, Iwan Hendrikx, Nathan Dunstan, Bryan G. Fry

**Affiliations:** 1 Venom Evolution Lab, School of Biological Sciences, The University of Queensland, St. Lucia QLD 4072, Australia; 2 Institute for Molecular Bioscience, The University of Queensland, St. Lucia QLD 4072, Australia; 3 Departamento de Biologia, Faculdade de Ciências, Universidade do Porto, Rua do Campo Alegre, 4169-007, Porto, Portugal; 4 CIIMAR/CIMAR-Interdisciplinary Centre of Marine and Environmental Research, University of Porto, Rua dos Bragas 289, P 4050-123 Porto, Portugal; 5 School of Earth and Environmental Sciences, University of Adelaide, SA 5005, Australia; 6 HEJ Research Institute of Chemistry, International Centre for Chemical and Biological Sciences (ICCBS), University of Karachi, Karachi-75270, Pakistan; 7 Venom Supplies Pty Ltd, Stonewell Rd, Tanunda SA 5352, Australia

**Keywords:** venom, evolution, phylogeny, elapid, Australia, molecular evolution, Darwinian selection, toxin phylogenies

## Abstract

Despite the unparalleled diversity of venomous snakes in Australia, research has concentrated on a handful of medically significant species and even of these very few toxins have been fully sequenced. In this study, venom gland transcriptomes were sequenced from eleven species of small Australian elapid snakes, from eleven genera, spanning a broad phylogenetic range. The particularly large number of sequences obtained for three-finger toxin (3FTx) peptides allowed for robust reconstructions of their dynamic molecular evolutionary histories. We demonstrated that each species preferentially favoured different types of α-neurotoxic 3FTx, probably as a result of differing feeding ecologies. The three forms of α-neurotoxin [Type I (also known as (aka): short-chain), Type II (aka: long-chain) and Type III] not only adopted differential rates of evolution, but have also conserved a diversity of residues, presumably to potentiate prey-specific toxicity. Despite these differences, the different α-neurotoxin types were shown to accumulate mutations in similar regions of the protein, largely in the loops and structurally unimportant regions, highlighting the significant role of focal mutagenesis. We theorize that this phenomenon not only affects toxin potency or specificity, but also generates necessary variation for preventing/delaying prey animals from acquiring venom-resistance. This study also recovered the first full-length sequences for multimeric phospholipase A_2_ (PLA_2_) ‘taipoxin/paradoxin’ subunits from non-*Oxyuranus* species, confirming the early recruitment of this extremely potent neurotoxin complex to the venom arsenal of Australian elapid snakes. We also recovered the first natriuretic peptides from an elapid that lack the derived C-terminal tail and resemble the plesiotypic form (ancestral character state) found in viper venoms. This provides supporting evidence for a single early recruitment of natriuretic peptides into snake venoms. Novel forms of kunitz and waprin peptides were recovered, including dual domain kunitz-kunitz precursors and the first kunitz-waprin hybrid precursors from elapid snakes. The novel sequences recovered in this study reveal that the huge diversity of unstudied venomous Australian snakes are of considerable interest not only for the investigation of venom and whole organism evolution but also represent an untapped bioresource in the search for novel compounds for use in drug design and development.

## 1. Introduction

Snake venoms are cocktails of toxins which have evolved from ordinary body proteins [1] to rapidly disrupt key physiological processes in prey animals. Since venom is energetically expensive to synthesize [2], an ideal venom-component would be effective even at lower concentrations. Therefore, target specificity of toxins is of paramount importance [3]. Since these toxins have evolved over millions of years of evolutionary time to rapidly and systematically breakdown prey homeostasis, they are invaluable as investigational ligands in elucidating physiological pathways or as lead compounds in drug design and therapeutics [4,5,6,7,8]. 

Australia is the stronghold of one of the world’s most medically significant families of venomous snakes—the front-fanged clade Elapidae. Elapid snakes include many of the world’s most infamous venomous snakes: the cobras of Asia and Africa; the mambas of Africa; the coral snakes of Asia and the Americas; the sea snakes and all of Australia’s medically significant venomous land snakes. The Australian continent is home to at least 130 (including sea snakes) of the world’s 320+ species of elapid snake [9]. Despite this tremendous diversity, to date, the vast majority of toxinological research conducted on the venoms of Australian snakes has focused on just five of Australia's 26 genera of terrestrial elapid snakes. Even within the five most studied genera, typically only one or two species per genus has received a significant amount of attention. These five genera (*Acanthophis*; *Notechis*; *Pseudechis; Pseudonaja;* and *Oxyuranus*) are considered the most medically significant of Australia’s venomous snakes [10], where "medical significance" is defined as the "danger posed to a human through bite". A further three genera (*Austrelaps*, *Hoplocephalus* and *Tropidechis*) have received a moderate amount of research attention, while the remaining 18 genera of terrestrial elapid snakes have historically been almost completely neglected by toxinologists. As more species have been investigated with novel methods, our knowledge of toxin evolution and structure-function relationships of these toxin types has increased. An initial investigation of the venoms of some small Australian elapids has shown them to be equally as complex as those of their larger, better-investigated cousins [11]. For this reason the small elapid fauna of Australia may be viewed as a rich and untapped bioresource, despite the fact that bites from many of these snakes is far from being “medically significant”.

**Figure 1:**
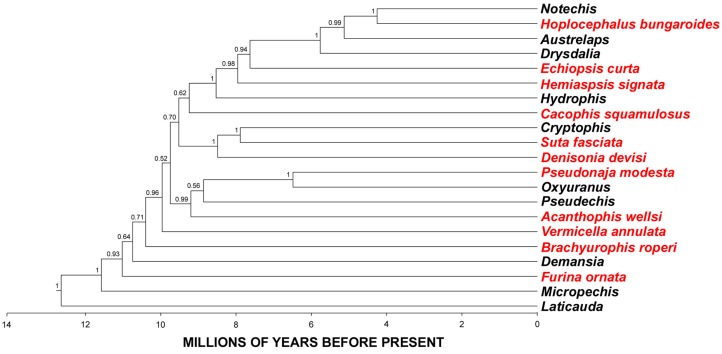
BEAST maximum credibility ultrametric tree for in-group taxa [12]. Node values indicate 95% highest posterior distributions for calibration points. Posterior probability support values are shown for each node. Species included in this study are indicated in red.

A wide variety of toxin types have been previously sequenced from Australian elapids, including cysteine-rich secretory proteins (CRiSP), toxin homologues of coagulation factors Va (fVaTx) and Xa (fXaTx), kunitz and waprin peptides, lectin, natriuretic peptides, phospholipase A_2_ (PLA_2_), metalloproteinases (SVMP), and three-finger toxins (3FTx) [13,14]. However, partly due to the limited range of taxa sampled, our current understanding of the true distribution and diversity of venom-components in these snakes remains far from complete. Thus many toxin types that are now considered “unique” to certain species or clades may only appear to be so because of the sampling bias towards medically significant large Australian Elapids.

Therefore, we investigated a wide taxonomical and ecological diversity of the under-studied Australian elapid snakes (Figure 1) through random sequencing of cDNA libraries; a method known to be efficient for biodiscovery and understanding the biochemical composition of snake venoms (*c.f.* [15,16,17,18,19,20,21,22,23,24,25,26]). The results of this study not only contribute to our understanding of the molecular evolution of Australian elapid snake venom, particularly the influence of feeding ecology on venom composition, but will also constitute a platform for biodiscovery. 

## 2. Results and Discussion

Random sequencing recovered a myriad of toxin types, previously only known from the other well studied species (Table 1). All venom gland transcriptomes contained sequences of multiple toxin types. Large globular proteins, such as acetylcholinesterase, CVF/C3 (cobra venom factor/complement 3), fXaTx, fVaTx, hyaluronidase, l-amino acid oxidase and SVMP, displayed very little variation in their coding sequences, as did CRiSP & nerve growth factor (NGF). This is consistent with the mode of evolution generally adopted by large globular proteins [1]. In contrast, extensive variation was seen for 3FTx, lectin, natriuretic, PLA_2_, kunitz and waprin toxin types. By examining the venom gland transcriptomes of a wide taxonomic range of neglected Australian elapid snake species, we have been able to gain deep insight into the molecular evolutionary history of major toxin classes. In addition to this, we have revealed that the toxic arsenals of the small Australian elapids, many of which are typically considered harmless to humans, are potentially similar in complexity to those of larger, “medically significant” species.

**Table 1:** Diversity of toxin transcripts recovered from each elapid snake species.

Species/Toxin	CRiSP	fXaTx	fVaTx	Kunitz	Lectin	Natriuretic	PLA_2_	SVMP	3FTx	Waprin
*Acanthophis wellsi*	X			X	X	X	X	X	X	X
*Brachyurophis roperi*	X			X	X	X	X		X	
*Cacophis squamulosus*	X			X	X	X	X		X	X
*Denisonia devisi*	X			X	X	X	X	X	X	X
*Echiopsis curta*	X			X	X	X		X	X	X
*Furina ornata*	X			X	X				X	
*Hemiaspis signata*	X	X		X	X	X	X	X	X	
*Hoplocephalus bungaroides*	X	X		X	X	X	X		X	
*Pseudonaja modesta*	X		X	X	X	X	X		X	X
*Suta fasciata*	X	X		X	X	X	X	X	X	X
*Vermicella annulata*	X			X	X			X	X	X

fXaTx = factor Xa; fVaTx = factor Va; PLA2 = phospholipase A2; SVMP = snake venom metalloprotease; 3FTx = three finger toxin.

### 2.1. 3FTx

3FTx are amongst the most abundant and well-studied components of elapid snake venoms [27]. The α-neurotoxic 3FTx from the venoms of Australian elapid snakes have been characterized into three groups: Types I, II (both also found in African and Asian elapids) and III (unique to Australian elapids [27]). Their cysteine arrangements and the number of residues present between cysteines [27] distinguish these three forms from one another. Type I (AKA short chain) α-neurotoxins are characterised by having lost the second and third cysteine residues present in the plesiotypic 3FTx form (leaving them with 8 cysteines), a change which may have resulted in a 100-fold increase in neurotoxicity [27]. Type II α-neurotoxins (AKA long chain) are characterised by having the same eight cysteines, but with an additionally derived pair located between the fourth and fifth plesiotypic cysteine (third and fourth of the cysteines shared with Type I α-neurotoxins) [27]. The presence/absence of these two derived cysteines is key to the potency and specificity of the two α-neurotoxins. While both bind strongly to neuromuscular post-synaptic nicotinic acetylcholine receptors (nAChR), only the Type II can bind to neuronal nAChR [27].

**Figure 2:**
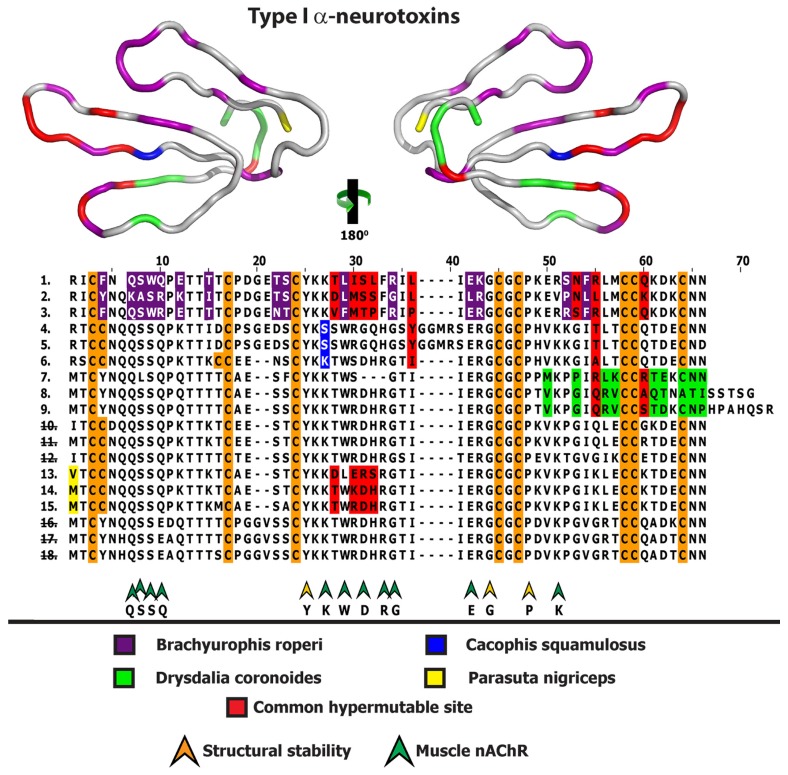
Homology model depicting the locations of positively selected sites from various species, indicated by different colour codes. Multiple sequence alignment of Type II α-ntxs depicting the locations of positively selected sites is also presented. Representative sequences are from *Brachyurophis roperi* (1. GAHA01000012, 2. GAHA01000013, 3. GAHA01000016), *Cacophis squamulosus* (4. GAHB01000003, 5. GAHB01000008, 6. GAHB01000008), *Drysdalia coronoides* (7. FJ752483, 8. FJ752485, 9. FJ752487), *Hemiaspis signata* (10. GAHF01000010, 11. GAHF01000011, 12. GAHF01000014), *Parasuta nigriceps* (13. FJ790454, 14. FJ790448, 15. FJ790450), *Vermicella annulata* (16. GAHJ01000013, 17. GAHJ01000014, 18. GAHJ01000015). Numerical IDs representing species lacking unique mutations are indicated by strikethrough.

The key functional sites for nAChR antagonizing activity in α-neurotoxins have been identified as being between residues 49 and 55 (between cysteines 3 and 4) in Type I α-neurotoxin and between residues 47 and 58 (between cysteines 3 and 6) in Type II α-neurotoxin [28]. The functional residues of the much smaller Type III α-neurotoxin remain to be elucidated. Variations at these functional sites, as well as variations in cysteine arrangements, are likely to have important consequences for the potency or affinity of these toxins and are thus of considerable interest in bioprospecting studies.

**Figure 3:**
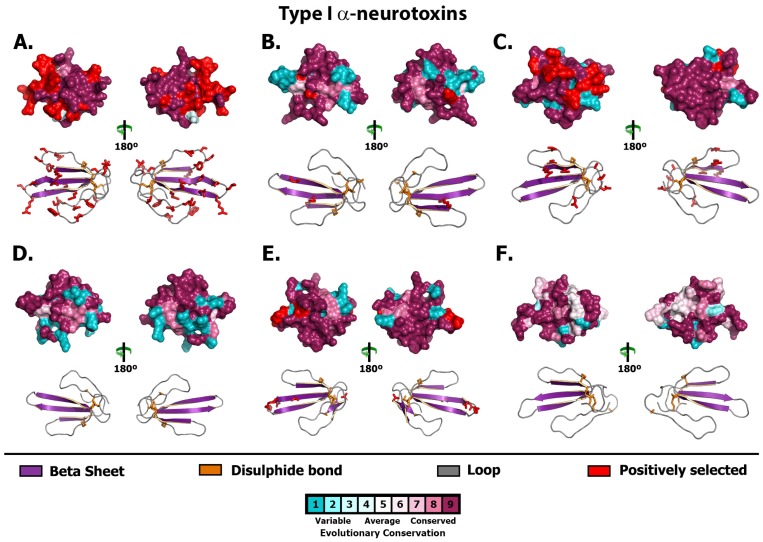
Molecular evolution of Type I (aka: short-chain) α-neurotoxins. Three-dimensional homology models of Type I α-neurotoxins from various species, depicting the locations of positively selected sites (Model 8, *PP* ≥ 0.95, Bayes-Empirical Bayes approach) is presented here. Species are: (A) *Brachyurophis roperi*, (B) *Cacophis squamulosus*, (C) *Drysdalia coronoides*, (D) *Hemiaspis signata*, (E) *Parasuta nigriceps* and (F) *Vermicella annulata*.

**Figure 4:**
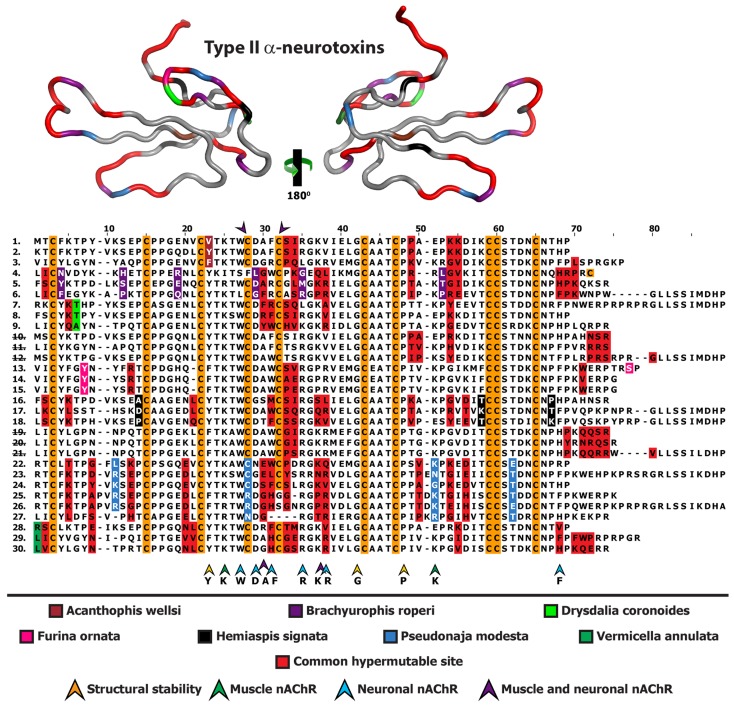
Homology model depicting the locations of positively selected sites from various species, indicated by different colour codes. Multiple sequence alignment of Type II α-ntxs depicting the locations of positively selected sites is also presented. Representative sequences are from *Acanthophis wellsi* (1. GAGZ01000001, 2. GAGZ01000004, 3. GAGZ01000006)*, Brachyurophis roperi* (4. GAHA01000003, 5. GAHA01000001, 6. GAHA01000002), *Drysdalia coronoides* (7. FJ481928, 8. FJ752461, 9. FJ752459), *Echiopsis curta* (10. GAHD01000001, 11. GAHD01000004, 12. GAHD01000006), *Furina ornata* (13. GAHE01000001, 14. GAHE01000009, 15. GAHE01000014), *Hemiaspis signata* (16. GAHF01000001, 17. GAHF01000005, 18. GAHF01000006), *Suta fasciata* (19. GAHI01000001, 20. GAHI01000004), 21. *Parasuta nigriceps* FJ790457, *Pseudonaja modesta* (22. GAHH01000040, 23. GAHH01000045, 24. GAHH01000046, 25. GAHH01000043, 26. GAHH01000042, 27. GAHH01000035) and *Vermicella annulata* (28. GAHJ01000009, 29. GAHJ01000010, 30. GAHJ01000016). Numerical IDs representing species lacking unique mutations are indicated by strikethrough.

**Figure 5:**
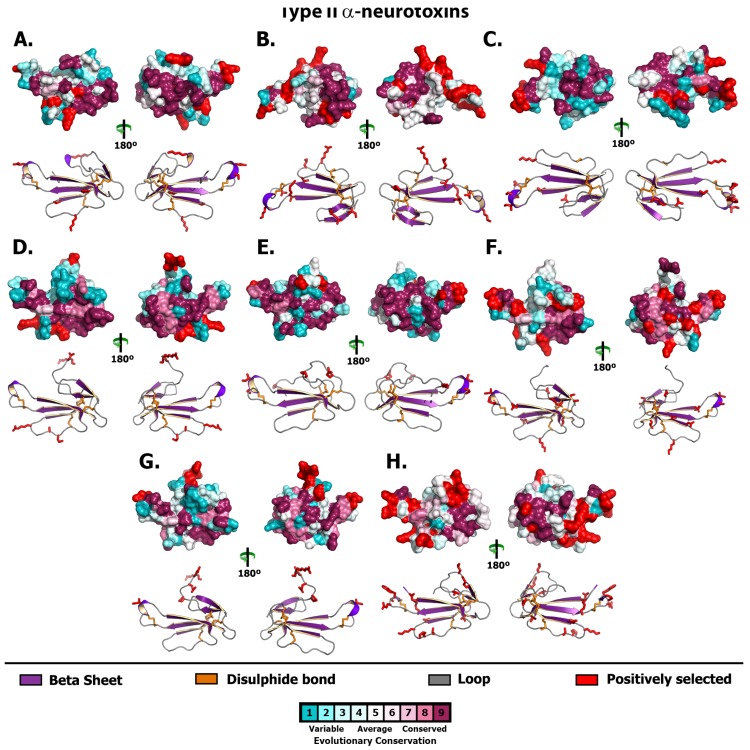
Molecular evolution of Type II (aka: long-chain) α-neurotoxins. Three-dimensional homology models of Type II α-neurotoxins from various species, depicting the locations of positively selected sites (Model 8, *PP* ≥ 0.95, Bayes-Empirical Bayes approach). Species are: (A) *Acanthophis wellsi*, (B) *Brachyurophis roperi*, (C) *Drysdalia coronoides*, (D) *Echiopsis curta*, (E) *Furina ornata*, (F) *Hemiaspis signata*, (G) *Parasuta nigriceps* and (H) *Pseudonaja modesta*.

**Figure 6:**
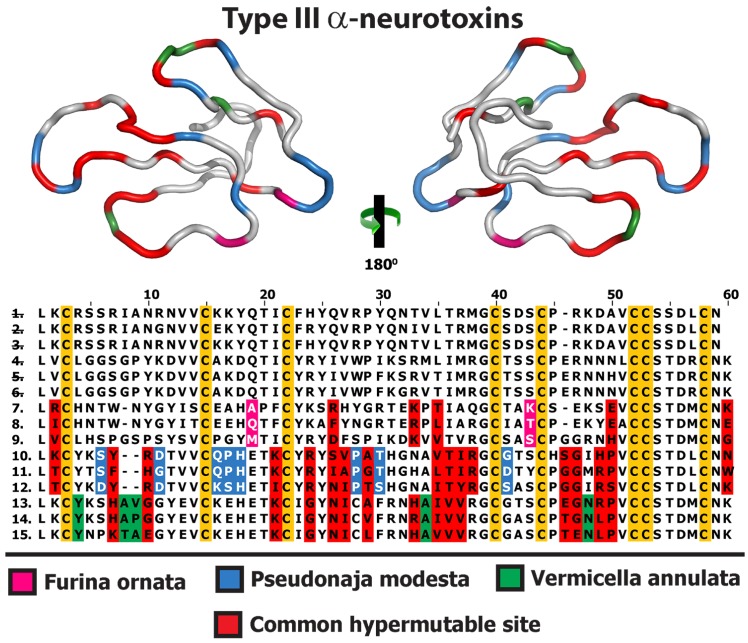
Structural and functional evolution of Type III α-neurotoxins. Multiple sequence alignment of Type III α-ntxs depicting the locations of positively selected sites (Model 8, *PP* ≥ 0.95, Bayes-Empirical Bayes approach) in various species of Australian elapids is presented here. Homology model depicting the locations of positively selected sites from various species, indicated by different colour codes, is also presented. Representative sequences are from *Brachyurophis roperi* (1. GAHA01000009, 2. GAHA01000010, 3. GAHA01000011), *Cacophis squamulosus* (4. GAHB01000009, 5. GAHB01000010, 6. GAHB01000011), *Furina ornata* (7. GAHE01000022, 8. GAHE01000023, 9. GAHE01000020, *Pseudonaja modesta* (10. GAHH01000009, 11. GAHH01000015, 12. GAHH01000022) and *Vermicella annulata* (13. GAHJ01000001, 14. GAHJ01000003, 15. GAHJ01000004). Numerical IDs representing species lacking unique mutations are indicated by strikethrough.

156 distinct 3FTx sequences were recovered in this study (46 from *P. modesta* alone), making this by far the most diverse toxin type. Of particular interest amongst these are Type I and II α-neurotoxins with novel cysteine arrangements (Figure 2, Figure 3, Figure 4, Figure 5 and Figure 6). A Type 1 isoform was recovered from *C. squamulosus* that possessed both the double cysteine (plesiotypic cysteine 1 and a novel cysteine) characteristic of and unique to some Australian elapid snake Type I α-neurotoxins, as well as an additional double cysteine (plesiotypic cysteine 2 paired with a novel cysteine). These toxins are part of an Australian elapid snake Type I α-neurotoxin clade with members that typically have an extra cysteine, so in this case the addition of a novel cysteine resulted in a unique ten cysteine arrangement, which may result in a novel folding pattern and activity.

The Type II α-neurotoxins recovered included isoforms with newly evolved cysteines in addition to variants with cysteine deletions (Figure 4 and Figure 5). An isoform recovered from *B. roperi* exhibiting deletion of the first of the newly evolved cysteines characteristic of Type II α-neurotoxins (located between plesiotypic cysteines 4 and 5 [27]), possessed an additional cysteine present near the C-terminus. This novel arrangement may have radical effects on disulphide bridging, thus exposing different residues upon the molecular surface of the toxin, with potential implications for relative bioactivity or potency. In contrast, isoforms from *P. modesta* had deletions of both of the Type II α-neurotoxin characteristic cysteines. Sequences lacking these two cysteines have previously been reported from the venom of *Pseudonaja textilis*, *Oxyuranus microlepidotus* and *O. scutellatus* [28]. Such a form from *P. textilis* has been shown in a prior study to be a blocker of both neuronal and neuromuscular nAChR [28]. This study did not, however, compare the neuronal binding affinity of this form to that of Type II α-neurotoxins with both these cysteines present. Thus change in relative potency or taxon specificity resulting from the loss of these cysteines remains unknown. The recovered diversity of molecular scaffolds is probably a result of the extreme influence of positive Darwinian selection experienced by α-neurotoxins [29].

**Figure 7:**
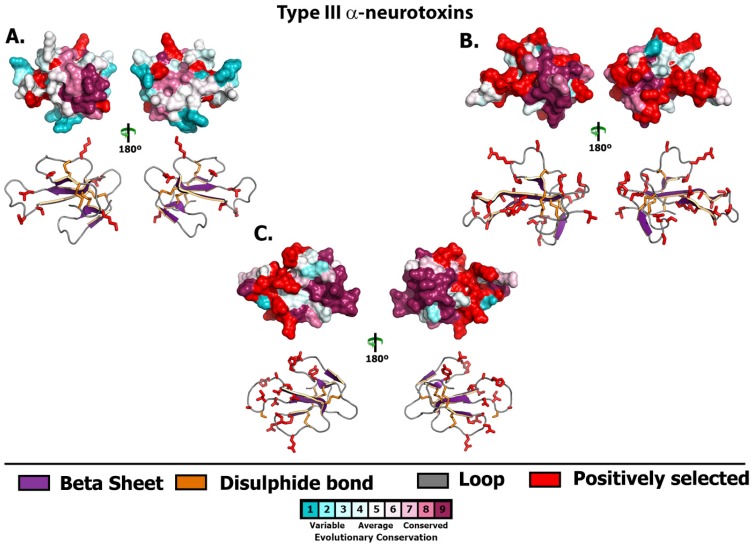
Molecular evolution of Type III α-neurotoxins. Three-dimensional homology models of Type III α-neurotoxins from various species, depicting the locations of positively selected sites (Model 8, *PP* ≥ 0.95, Bayes-Empirical Bayes approach). Species are: (A) *Furina ornata*, (B) *Pseudonaja modesta* and (C) *Vermicella annulata*.

3FTx sequence diversity extended beyond structural residues, with 3FTx recovered in this study differing from previously published 3FTx at key functional sites believed to be responsible for α-neurotoxic activity (Figure 2, Figure 4 and Figure 6). These include, amongst the Type I α-neurotoxin sequences, isoforms from *B. roperi, C. squamulosus, F. ornata, H. signata* and *V. annulata*. Amongst the Type II α-neurotoxin sequences, those with novel residues at functional sites include the aforementioned sequences (with deletion of one or both Type II characteristic cysteines) from *B. roperi* and *P. modesta*. While the key functional residues for the Type III α-neurotoxins have not been elucidated, the significant sequence diversity in the region of the loop-tips (Figure 6 and Figure 7) suggests that significant variation in potency or specificity may exist for this toxin type as well.

Phylogenetic analysis of 3FTx revealed that all sequences recovered in this study were placed into the Type I, II, or III α-neurotoxin clades previously characterised from Australian elapids [27]. Type III α-neurotoxins were recovered from *P. modesta,* which was expected as they have previously been characterised from other *Pseudonaja* as well as *Oxyuranus* venoms [28,30,31]. Interestingly, they were also recovered from *B. roperi*, *C. squamulosus, F. ornata* and *V. annulata*, representing the first time this toxin type has been recovered from species outside the *Pseudonaja*/*Oxyuranus* clade. Thus, it appears that this unique form was derived at the base of the Australian elapid snake radiation.

As 3FTx sequences were by far the most numerous toxin sequences recovered, the molecular evolution of this toxin type in the venom system of Australian elapid snakes was further investigated. Integrative selection assessment using various methodologies (codeml site-specific models: M8, M2a, M3, M0; HyPhy: SLAC, FEL, REL, MEME, FUBAR, integrative analyses, branch-site REL and the evolutionary fingerprint analyses) revealed that most α-neurotoxins in the venoms of small Australian elapid snakes have evolved rapidly and episodically under the significant influence of positive selection (Figure 2, Figure 3, Figure 4, Figure 5, Figure 6 and Figure 7; Supplementary Table 1). An exception is that of a *V. annulata* Type I α-neurotoxin, which was found to lack variation in coding sequences and appeared to have evolved under a regime of negative selection. Interestingly, Type II and Type III α-neurotoxins from this species accumulated variations rapidly under the influence of positive selection. Similarly, most Type I α-neurotoxin sequences recovered from *H. signata* were well conserved, while Type II α-neurotoxins from the same species were found to harbour large number of hypermutable sites. Since we only recovered three Type III α-neurotoxin sequences from *B. roperi* and *C. squamulosus,* selection assessment was not conducted for these sets. However, all Type III sequences in both these species shared a very high degree of sequence identity. In contrast, Type I and Type II α-neurotoxin sequences from *B. roperi* and Type I α-neurotoxin from *C. squamulosus* contained a greater amount of coding sequence variation. Thus, molecular evolutionary assessments revealed that the three kinds of α-neurotoxins in the venoms of small Australian elapid species have adopted differential evolutionary rates. Within each species, while one of the three forms of α-neurotoxin remains conserved, the other two accumulate significant variations, with the forms conserved or varied differing between species.

We define focal mutagenesis as a phenomenon where the rapid accumulation of non-synonymous mutations under the influence of positive Darwinian selection in certain regions of the protein, such as the loops and the molecular surface of the toxin [29], has adaptive significance. Although, mutations occur randomly and the probability of a mutation occurring in structurally/functionally important and unimportant regions is theoretically equal, mutations in structurally/functionally important regions could result in the formation of destabilised and defective toxins. Since venom is energetically very expensive to produce [2], it would be a huge waste of resources for a venomous organism to secrete defective venom components. As a result, during the course of evolution, negative selection filters harmful mutations out of the populations and a greater majority of mutations are found in those regions that are structurally/functionally unimportant. Cysteine residues that allow the formation of disulphide bonds and in turn stabilise the toxin structure are one of such very important regions and hence are extremely well conserved in various venom-components. The mutation of surface chemistry under the influence of positive Darwinian selection, may not only enable toxins to non-specifically interact with novel prey receptors and generate a plethora of pharmacological reactions in the prey, but at the same time might also allow them to escape/delay immunogenic response of prey animals. Not-surprisingly, focal mutagenesis has been documented in a diversity of venomous animal lineages [20,32,33,34,35,36,37,38,39,40]. 

Recently, we have demonstrated that various types of three-finger toxins in elapid venoms have adopted RAVER, and accumulate hypermutable sites in their loops and on the molecular surface, while conserving structurally and functionally important residues [29]. Mapping of hypermutable sites on the alignments and three-dimensional homology models of various 3FTx functional forms recovered from various small Australian elapids, revealed a number of interesting evolutionary phenomena: (i) α-neurotoxins from various species have accumulated variations in similar regions of the toxin, highlighting the role of focal mutagenesis in the evolution of these genes; (ii) certain species have harboured unique hypermutable sites, which might account for the differences in efficacy and/or potency of these toxins; (iii) residues in α-neurotoxins that have been elucidated as structurally or functionally important in other species were found to be extremely well conserved in most Australian elapids, while most hypermutable sites were found in regions not known to be important for function or structural stability; (iv) most hypermutable sites were concentrated in the loops of the toxins and (v) sequences from different species have different sets of residues that exhibit conservation, indicating that the diet of these snakes could have influenced the evolution of α-neurotoxin gene. Thus, molecular evolution assessment not only revealed that α-neurotoxins in the venoms of small Australian elapid snakes have adopted differential rates of evolution and focal mutagenesis, but also suggested that there may be a correlation between the feeding ecology of a species and the evolution of its α-neurotoxin-encoding gene. The diversity of residues found in functionally important sites of α-neurotoxins from species with differing feeding ecologies indicates that the evolution of these toxins in small Australian elapid snakes may have been driven by dietary specialisation. Investigations of the venom proteome of these snakes may be illuminating in this regard as may pharmacological investigation of the potential prey-specificity of individual α-neurotoxin isoforms and/or clades.

### 2.2. Lectin

The lectin sequences recovered in this study considerably expand the number of lectin sequences recovered from the venoms of elapid snakes. The plesiotypic form of lectins from reptile venom has been demonstrated to possess a “carbohydrate specificity triad” of functional residues that confers either galactose-binding (QPD motif—[41]) or mannose-binding (EPN motif—[42]) mediated haemagglutination activity, with EPN as the plesiotypic state. The majority of lectins recovered from this study had the EPN motive. However, sequences with the galactose-binding QPD motif were recovered from *Cacophis squamulosus* and *Pseudonaja modesta*. In addition to these two functionally-characterised motifs, sequences were recovered in this study that had the novel motifs KPG (*Acanthophis wellsi*) or YRH (*Cacophis squamulosus*) at the carbohydrate binding site (data not shown). These divergent sequences may prove to have novel activities.

### 2.3. Natriuretic Peptides

The venom gland transcriptomes of several species of elapid snake examined in this study (e.g., *E. curtus, H. signata*) contained numerous distinct isoforms of natriuretic peptide, suggesting that this toxin type may be a more important component of the venom of some species than hitherto realised. C-type natriuretic peptides (CNP) from snake venom are potent vasodilators and hypotensive agents (*c.f.* [43,44]). In addition, the precursor contains multiple proline rich peptides, one of which was utilised in the development of the multibillion-dollar drug Captopril [45]. For this reason they are considered to be amongst the most promising of all animal toxins for pharmaceutical bioprospecting [4,5,6,7,46]. Despite this high level of interest, very few complete sequence precursors of snake venom natriuretic peptide precursors have been recovered. The final processed peptide of the natriuretic domain has been sequenced from *Austrelaps superbus*; *Cryptophis nigrescens*; *Hoplopcephalus stephensii*; *Notechis scutatus*; *Oxyuranus microlepidotus*; *Oxyuranus scutellatus*; *Pseudechis australis*; *Pseudechis porphyriacus*; *Pseudonaja textilis*; and *Tropidechis carinatus* [43,47,48] while only a single complete precursor had been sequenced prior to this study (from *O. scutellatus* (Uniprot accession P83228)). Due to this low level of sequencing, our understanding of the evolutionary history of this toxin type was far from complete.

**Figure 8:**
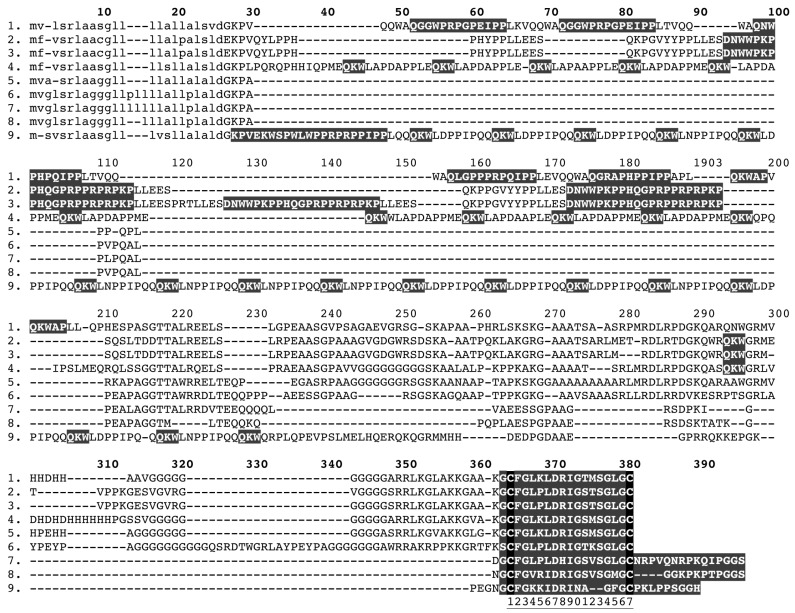
**Sequence alignment of natriuretic peptides**. (1). P68515 *Bothrops insularis*, (2). K4J3K2 *Azemmiops feae*, (3). K4IT20 *Azemmiops feae*, (4). A8YPR6 *Echis ocellatus*, (5). Q09GK2 *Philodryas olfersii*, (6). GAHI01000013 *Suta fasciata*, (7). P83228 *Oxyuranus scutellatus*, (8). GAHI01000016 *Suta fasciata*, (9). A8YPR9 *Cerastes cerastes* Post-translationally cleaved peptides in shaded in gray.

**Figure 9:**
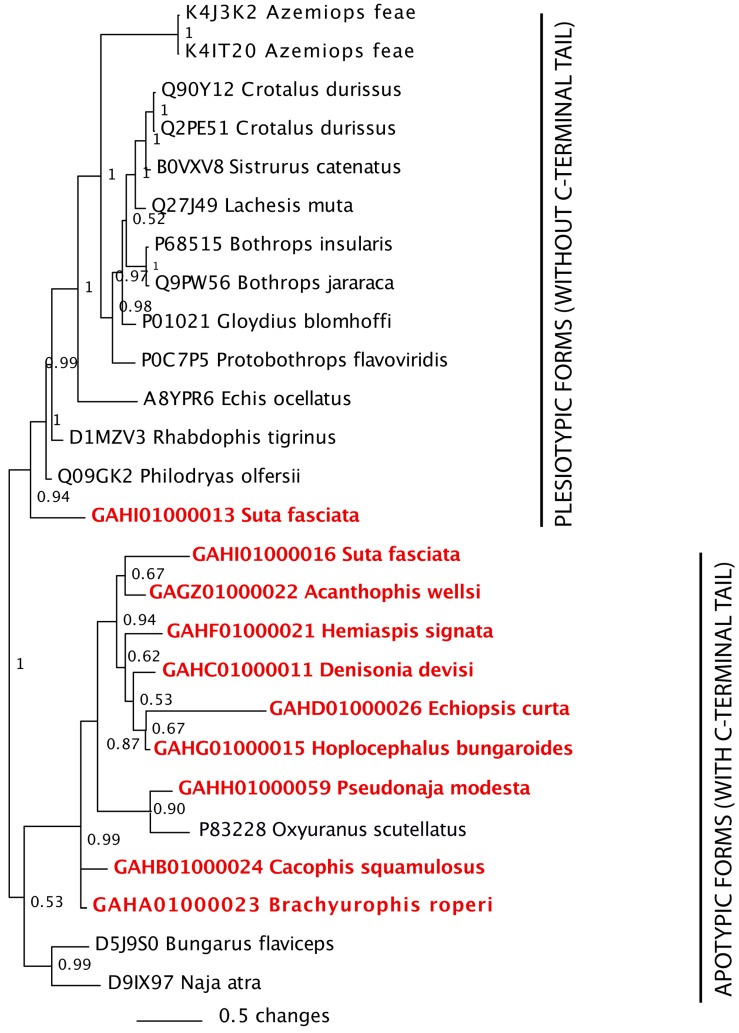
Phylogenetic reconstruction of the molecular evolutionary history of natriuretic peptides. Non-toxin outgroup sequences (P23582 and P55207) not shown. Representative sequences obtained in this study are shown in red. Node labels indicate posterior probabilities.

In this study, in addition to retrieving natriuretic sequences, which were similar to the previously characterised precursors from the elapid snake *Oxyuranus scutellatus* (Uniprot accession P83228),isoforms were recovered that lacked the derived C-terminal tail typical of elapid natriuretic toxin variants (Figure 8). Instead, these toxins resembled viperid and “non-front-fanged” (NFF) advanced snake natriuretic homologues in lacking the tail and possessing glycine rich regions characteristic of the aforementioned forms. Phylogenetic analyses demonstrated the relationship of these novel elapid snake sequences to the tail-less natriuretic sequences recovered from viperid and colubrid snakes (Figure 9).

Natriuretic peptides in elapid snake venoms have previously been inferred to have been derived from ANP (atrial natriuretic peptide) or BNP (brain natriuretic peptide) due to the presence of the C-terminal tail, while those of viperid snakes were said to be derived from CNP (c-type natriuretic peptide) due to lack of the tail [1,44,49,50,51]. However, recent studies have shown that all snake natriuretic toxins form a monophyletic group within the CNP clade [15,22]. Despite this, the evolutionary linkage between elapid and viperid sequences has remained enigmatic. It has been suggested that presence of the C-terminal extension is an apotypic (derived) condition of the peptide and that the tail-less form, previously only recovered from viperid snakes, is the plesiotypic condition [22]. The recovery in this study, for the first time, of tail-less forms from elapid snakes (*C. squamulosus* and *S. fasciata*) supports this hypothesis, particularly as these sequences also possess the glycine-rich region of the propeptide that is characteristic of the tail-less viperid snake forms. In phylogenetic analyses performed in the present study, the tail-less elapid snake forms grouped with tail-less forms from viperid and colubrid snakes and the sole published viperid snake precursor (from *C. cerastes*) possessing the C-terminal extension grouped with elapid and lamprophiid snake sequences that share the tail (Figure 9). These results add further strength to the hypothesis that the C-terminal extension represents a derivation of this toxin type and thus the action of the tailed forms on GC-A [43] is not indicative of descent from the atrial natriuretic peptide (ANP) but instead represents a remarkable case of convergence for receptor-specific targeting [22]. The C-terminal extension appears to have evolved in elapid snake venom natriuretic peptides to confer greater affinity for GC-A (guanylate cyclase A) and NPR-C (natriuretic peptide receptor C) receptors [43] and greater resistance to proteolytic cleavage from the receptors [52]. Recently a study was published in which a natriuretic peptide possessing the C-terminal extension was sequenced from the venom of the rattlesnake *Crotalus oreganus abyssus* [53]. The published sequence represents only the final processed natriuretic peptide amino acid sequence and does not provide precursor information. As all snake sequences group into a monophyletic clade nested within the C-type natriuretic peptide (CNP) clade of non-venom precursors (Figure 9) [15,22], the venom form is clearly of CNP ancestry and the C-terminal tail evolved soon after recruitment of this form to the venom system.

Recently, a proline-rich peptide was isolated from the venom of *Dendroaspis* *angusticeps* [54] which was described as being of an unknown peptide or protein type. The results of this study reveal that this proline-rich peptide is part of the propeptide region of the natriuretic peptide precursor, a fact missed by the aforementioned *Dendroaspis* study despite an *Oxyuranus* precursor being available that showed significant similarity to this peptide in an early stretch of the propeptide region. Such a region was preserved in the sequences obtained in this study. This region is posttranslationally cleaved from the precursor and undergoes dynamic evolution facilitating neofunctionalisation. It is where the bradykinin-potentiating peptides (of Captopril fame) evolved, and is also the source of the novel neurotoxic peptides from *Azemiops feae* and *Psammophis mossambicus* [32,55]. Thus, it represents an unexplored source of novel peptides with potentially useful activities for drug design and development.

### 2.4. PLA_2_

PLA_2_ toxins are an important component of the venom of many Australian elapid snakes and typically exhibit presynaptic neurotoxic activity, myotoxic activity, or both, although some forms exhibit antiplatelet activity (*c.f.* [14]). Almost all previous sequences have come from the well-studied larger species, despite toxic effects of venoms from the smaller snakes indicating they are rich in PLA_2_ toxins. For example *Furina* (*Glyphodon*) *tristis* venom has been demonstrated to be presynaptically and myotoxically active [56], indicating the presence of PLA_2_ toxins, a result consistent with components detected in the venom of this species by LC/MS (liquid chromatography mass spectrometry) that had PLA_2_ characteristic retention times and molecular weights [57]. In addition, the venoms of two species of small elapid snake from the genus *Suta* have been pharmacologically characterised as potently neurotoxic (*S. suta*) or myotoxic (*S. punctata*) [58]; mass spectrometry of these venoms detected components consistent with PLA_2_ as well as three-finger toxins [57]. The Kuruppu 2007 study could not rule out the presynaptic mode of action for the observed neurotoxicity, while the myotoxicity was almost certainly caused by the action of PLA_2_. These results demonstrate that the venoms of small Australian elapid snakes contain similar toxins to those of their larger cousins, and are potentially capable of delivering serious bites. This potential has been made alarmingly clear in the case of *Furina tristis* and *Suta suta*, both of which have been responsible for life-threatening envenomations in which the neurotoxic action of the respective venoms was poorly reversed by polyvalent antivenom ([10], BG Fry, personal observations). The results of the present study considerably augment the number of sequenced Type II PLA_2_ full length sequences from elapid snakes. The recovery of PLA_2_ sequences from 8 of the 11 libraries confirms that PLA_2_ is one of the most important and highly expressed toxin types in the venom of Australian elapid snakes. The new sequences recovered in the present study also provide further insight into the molecular evolutionary history of this clinically significant toxin type (Figure 11).

Several previously published PLA_2_ sequences are known to be components of multimeric neurotoxin complexes (e.g., taipoxin gamma chain [59]). These sequences feature a novel cysteine arrangement with the insertion of two additional cysteines between plesiotypic cysteines 1 and 2. Multimeric presynaptic PLA_2_ have long been known from the venoms of *Oxyuranus sp.* and *Pseudonaja* *sp* and are amongst the most potent neurotoxins known*.* Such complexes have recently been reported to be present in the venoms of *Acanthophis sp*. [60,61,62,63].

**Figure 10:**
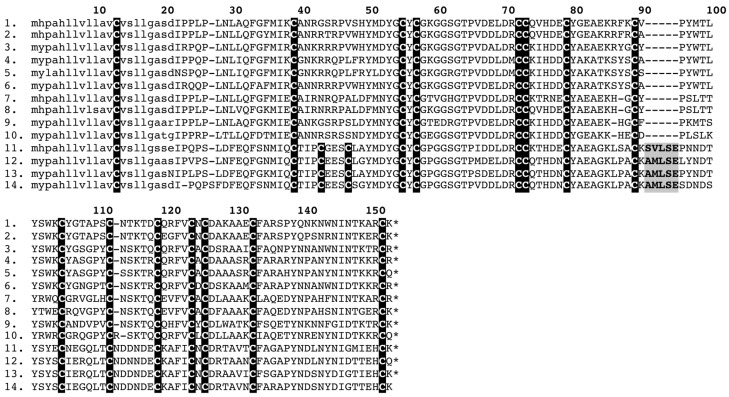
Sequence alignment of ‘taipoxin/paradoxin’-like presynaptic complex subunits: α-subunit (1). Q45Z43 *Oxyuranus microlepidotus*, (2). Q45Z48 *Oxyuranus scutellatus*, (3). GAGZ01000028 *Acanthophis wellsi*, (4). A6MFM9 *Rhinoplocephalus nigrescens*, (5). GAHI01000025 *Suta fasciata*, (6). B5G6G1 *Tropidechis carinatus*, β-subunit (7). Q45Z46 *Oxyuranus microlepidotus*, (8). Q45Z53 *Oxyuranus scutellatus*, (9). GAGZ01000024 *Acanthophis wellsi*, (10). GAHI01000027 *Suta fasciata* and γ-subunit (11). Q4VRI6 *Oxyuranus scutellatus*, (12). GAGZ01000027 *Acanthophis wellsi*, (13). Q9PUG7 *Austrelaps superbus*, (14). GAHI01000030 *Suta fasciata*.

**Figure 11:**
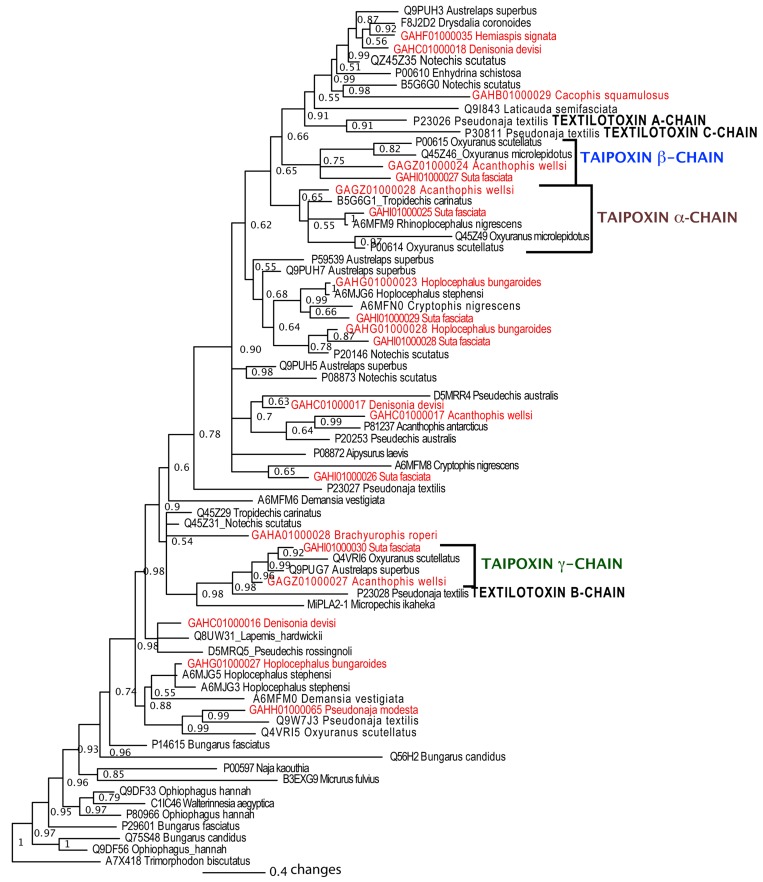
Phylogenetic reconstruction of the molecular evolutionary history of snake venom Type I phospholipase A_2_ toxins. Non-toxin outgroup sequences (Q8JFB2 and Q8JFG2) not shown. Representatives of sequences obtained in this study are shown in red. Node labels indicate posterior probabilities.

In the present study, sequences homologous with all three chains of taipoxin were recovered from *A. wellsi* and such transcripts were also present in the *S. fasciata* library (Figure 10). This supports a previous hypothesis that they were likely widespread in the venoms of Australian elapids [64]. Phylogenetic analyses showed that these *Acanthophis* and *Suta* sequences grouped into clades with each of the respective taipoxin subunits (α, β and γ) from *Oxyuranus scutellatus* (Figure 11). Intriguingly, the *P. modesta* library did not include any of the subunits of the potent presynaptic neurotoxic PLA_2_ complex ‘textilotoxin’ present in the venom of the congeneric *Pseudonaja textilis* (Figure 11).

### 2.5. Kunitz and Waprin

**Figure 12:**
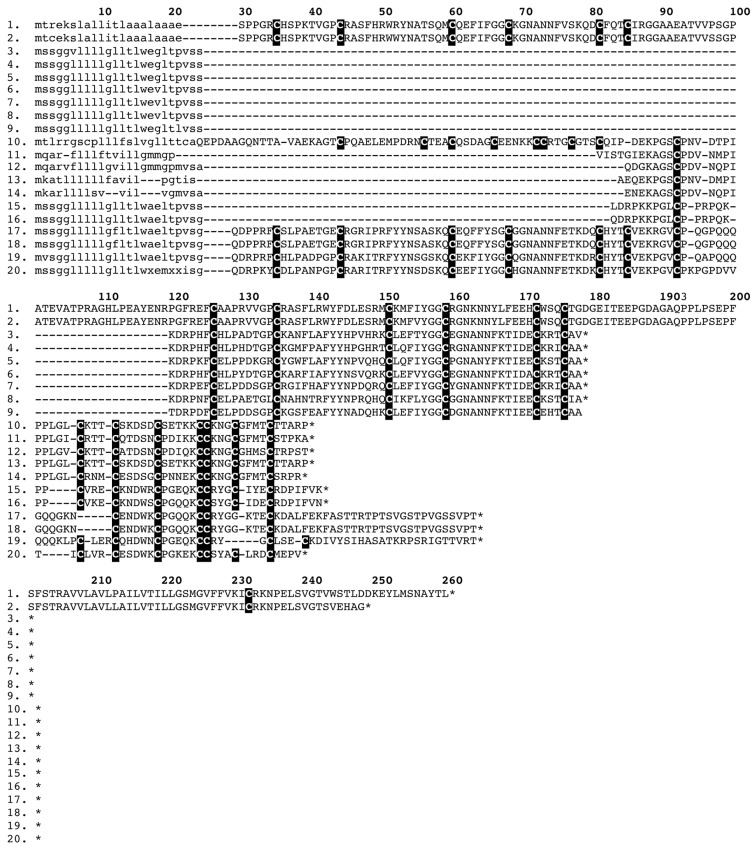
Sequence alignment of precursors encoding: dual-domain kunitz (1). B2BS84 *Austrelaps labialis*, (2). GAHG01000009 *Hoplocephalus bungaroides*; mono-domain kunitz (3). GAGZ01000019 *Acanthophis wellsi*, (4). GAGZ01000017 *Acanthophis wellsi*, (5). GAHB01000016 *Cacophis squamulosus*, (6). GAHD01000011 *Echiopsis curta*, (7). GAHG01000008 *Hoplocephalus bungaroides*, (8). GAHH01000051 *Pseudonaja modesta*, (9). GAHI01000010 *Suta fasciata;* dual-domain waprin (10). A7X4K1 *Philodryas olfersii*; mono-domain waprin (11). GAHC01000021 *Denisonia devisi*, (12). A7X4J4 *Rhabodophis tigrinus*, (13). A7X4K7 *Philodryas olfersii*, (14). A7X4I7 *Thrasops jacksonii*, (15). B5G6H4 *Notechis scutatus*, (16). B5G6G8 *Oxyuranus scutellatus*; kunitz-waprin fusion (17). D3U2B9 *Sistrurus catenatus edwardsii*, (18). D3U0D3 *Sistrurus catenatus tergeminus*, (19). GAHB01000034 *Cacophis squamulosus*, (20). GAHI01000009 *Suta fasciata.*

An interesting picture emerges from the results of this study in relation to the molecular evolutionary histories of the kunitz and waprin peptides. In addition to the single domain kunitz and waprin sequences, dual domain (kunitz-kunitz) and fused dual domain (kunitz-waprin) precursors were also recovered (the latter representing the first time fused kunitz-waprin precursors have been recovered from elapid snakes) (Figure 12). It is noteworthy that a mono-domain waprin sequence recovered from *Denisonia devisi* was highly similar to a previously published sequence from the natricid NFF advanced snake *Rhabdophis tigrinus* [19], and shared an identical signal peptide. Dual domain (kunitz-kunitz) kunitz peptide sequences were recovered from the venom gland transcriptomes of *Furina ornata* (fragment), *Hoplocephalus bungaroides* and *Hemiaspis signata* and fused dual domain kunitz-waprin toxin sequences were recovered from *Cacophis squamulosus* and *Suta fasciata*. Signal peptides differed considerably between the kunitz-kunitz dual domain precursors and the single domain kunitz precursors, the single domain waprin peptides and kunitz-waprin hybrid precursors (all of which share the same signal peptide in elapid snake forms [65] other than the novel waprin sequences from *D. devisi* mentioned above-Figure 12).

The fact that both dual-domain precursors of these toxin types are widespread and that the mammalian physiological homologues also have dual-domain precursors for both peptide types [66] strongly suggests that the plesiotypic form of each toxin type is a dual-domain precursor, consistent with phylogenetic analyses of the relative position of the dual-domain form [20,33]. Thus the mono-domain forms are secondary derivations resulting from the loss of a domain. In both toxin types it appears to be the first domain that has been deleted. It is worth noting, pending proteomic investigation, that it remains unknown whether or not the kunitz-waprin fused toxin is posttranslationally processed into two separate toxins or if it acts as a complex of hitherto uncharacterised activity.

The origin of the shared signal peptide of mono-domain kunitz and waprin peptides, as well as fused kunitz-waprin forms, is enigmatic. It has been suggested that kunitz and waprin arose from duplication of the same ancestral gene and that subsequent diversification occurred only in the toxin-encoding region, thus preserving the sequence homology of the signal peptides [67,68]. This hypothesis is in conflict with the fact that the ancestral dual-domain encoding toxin forms of each of these peptides types possess distinct signal peptides. Mono-domain waprin peptides sequenced from colubrid snakes also possess a different signal peptide to previously published mono-domain forms from elapid snakes, although in the present study a sequence with high degree of similarity (including the same signal peptide) to a sequence from the natricid snake *Rhabdophis tigrinus* [19] was recovered from the elapid snake *Denisonia devisi*. Only two (mono-domain) kunitz precursors have been recovered from colubrid snakes to date, one of which (*Telescopus dhara*) possesses the shared signal peptide, whilst the other (*Philodryas olfersii*) possesses yet another distinct signal peptide [19]. Mono-domain kunitz from viperid snakes possess the shared signal peptide (*c.f.* [69]). As yet, no mono-domain waprin peptide precursors have been sequenced from viperid snakes. It is apparent that a gene-splicing event took place at some point in the molecular evolutionary history of these toxins but the origin of the shared derived signal peptide is unclear. Phylogenetic analysis of kunitz sequences recovered in this study, along with previously published sequences, revealed that the dual kunitz-domain encoding precursors group together as a basally-divergent clade, further strengthening the hypothesis that the dual-domain precursor is the plesiotypic form of this toxin type.

The function of waprin in snake venom is almost entirely unknown. Only one component, from the venom of *O. microlepidotus*, has been tested and this toxin, named “omwaprin”, was found to exhibit weak antimicrobial activity [70]. However, phylogenetic analysis of sequences recovered in this study revealed an interesting level of sequence diversity. Although the majority of elapid sequences grouped together, a sequence recovered from *C.* *squamulosus* in this study was found to be basally-divergent from the main clade of elapid sequences while sequences recovered from *A. wellsi*, *D. devisi* and *V. annulata* displayed affinity to previously published sequences from colubrid snakes and African elapid snakes. The clustering of the fused kunitz-waprin precursors within the main clades of waprin or kunitz toxin sequences in the respective analyses suggests that the mono-domain toxin forms of these peptides had already been derived prior to their fusion. Nonetheless, the presence of the fused toxin in both viperid and elapid snakes, which are the most divergent pair of colubroid snake families, suggests that the kunitz-waprin splicing is relatively ancient, and occurred at the base of the Colubroidea radiation.

### 2.6. Procoagulant Toxins and Clinical Implications

As well as theoretical implications regarding molecular evolution, our discoveries may prove important in considerations of the potential clinical effects of bites from these species, such as our sequencing of fXaTx from *H. signata* and *H. bungaroides*, which is consistent with coagulopathic effects produced by the venom of these snakes [11,71,72]. Of interest is the inability in the current study to recover fXaTx sequences from the venom gland transcriptome of *P. modesta*; this is significant since high expression levels of fXaTx and fVaTx are characteristic of the venom of other members of the genus *Pseudonaja* [13,14]. Although it is possible that fXaTx is expressed in very low levels and that the sampling in this study simply failed to detect it, it is also possible, as previously suggested [73] that the venom of this species, unusually for a member of its genus, lacks any procoagulant component to its venom. The recovery of fVaTx from *P. modesta* in the present study, however, raises further interesting questions. A rate-limiting step in the action of fXaTx is that it must form a 1:1 complex with activated factor V in the bitten animal’s blood [74]. fVaTx is present in the venoms of species *Pseudonaja* and all studied species of *Oxyuranus* and, as it has not been detected in the venoms of any other species, it is believed to have been recruited into the venom system of the common ancestor of both genera, which form a monophyletic clade [14,75]. Not only is this toxin responsible for the potentially fatal venom-induced consumption coagulopathy (ViCC) often suffered by victims of bites from these snakes [76], it has also been hypothesised that this toxin played an important role in the evolutionary transition of snakes in this clade from a diet of reptiles and frogs (common to most Australian elapid snakes including the species included in this study) to a diet specialising in mammalian prey [19,77]. In the venom of these snakes, fVaTx forms a complex with fXaTx creating a “complete prothrombin activator” [78]. The function of the venom form of fVaTx is to act as a cofactor for fXaTx that doesn’t require proteolytic cleavage to function and is resistant to the regulatory activity of the anticoagulant protein C [79]. The venom form of fVaTx is therefore a cofactor for fXaTx that is faster acting and harder to stop than its endogenous counterpart. Why *P. modesta* might express this cofactor in the absence of fXaTx is a mystery. The absence of fXaTx may, however, relate to the diet of this species—*P. modesta*, uniquely for a member of its genus, appears to prey exclusively upon reptiles [80]. It is also worth noting that only very low expression levels of fVaTx were detected in the *P. modesta* library, which indicates that its expression is probably down-regulated in this species relative to other *Pseudonaja sp*. In order to absolutely confirm the absence of fXaTx in the venom system of *P. modesta* and in order to shed light on the recruitment date of this important toxin, it will be necessary in a future study to use PCR amplification with primers to facilitate the detection of sequences which may be expressed at very low levels. The radically different venom profile of *P. modesta* however is indicative of a potential failure of CSL Brown Snake antivenom to neutralise envenomation effects as the primary antibodies in the antivenom are selected towards the most dominant toxins in the immunising venom (*Pseudonaja textilis*), and thus would likely be directed predominantly against the fXaTx-fVaTx complex and not against 3FTx which is the dominant toxin type in *P. modesta* venom. This is crucially important as 3FTx been hypothesised to poorly immunogenic despite the high toxicity [81].

### 2.7. Influence of Prey Preference on Venom Gland Transcriptome

There is abundant evidence that prey preference drives the evolution of venom composition and toxicity in snakes [4,20,37,77,82,83,84]. It is perhaps surprising, therefore, that a reptile specialist like *S. fasciata* should have such a complex venom gland transcriptome, but it remains to be demonstrated whether or not the venom proteome is similarly complex or if it is complex but dominated by one particular toxin type or particular isoforms within a toxin type. Even the venom gland transcriptome of *V. annulata,* a species with venom that has been previously been inferred (via mass spectrometry analysis) to be dominated by a single toxin class (3FTx—[11]), contained a diversity of other toxin types. *V. annulata* is a burrowing species with a highly specialised diet; apparently feeding only on blind snakes [85]. One fairly intuitive hypothesis is that species with highly specialised diets are likely to have simpler venom compositions than those that feed on a wide range of prey. The results from the aforementioned study [11] supported this hypothesis, however the results of the present study, in which precursors for 12 distinct toxin types were recovered from the transcriptome of *V. annulata,* appear to contradict it. One possible explanation is that *V. annulata* is descended from a species with a broader diet and hence a more diverse venom composition. If that is the case, it is likely that microRNA is blocking the translation of these precursors into toxins [86], as the snake, with its derived diet of blind snakes, no longer requires them. Further proteomic investigation of the venom of this species will confirm whether or not its venom is indeed streamlined and subsequent pharmacological investigation of the dominant toxins will confirm whether or not they exhibit taxon-specific potency variation. Confirming these properties of the venom and toxins would strongly support the hypothesis that *V. annulata* and its prey are engaged in a co-evolutionary predator-prey chemical arms race. It should be noted that a complex toxin cocktail might also be helpful for defensive purposes.

### 2.8. Brachyurophis roperi: A Unique, Oophagous Burrowing Elapid Snake

The preponderance of unique sequences within the *B. roperi* venom gland transcriptome is something of a mystery. *B. roperi* are believed to be specialist egg-eaters and have the most specialised dentition for oophagy of any terrestrial Australian elapid snake (Scanlon and Shine, 1988). In oophagous marine elapid snakes, a degeneration of the venom system has been noted, along with deletions of functional residues in 3FTx and accumulation of deleterious mutations in PLA_2_ [87,88]. It is possible that a similar scenario is taking place within the venom system of *B. roperi*. Unlike the oophagous sea snakes *Aipysurus eydouxii*, *A. mosaicus* and *Emydocephalus annulatus*, however, *B. roperi* appears to retain fangs that are similar in size to those of non-specialist congeners that feed on both eggs and lizards [89]. In addition, a bite from *B. roperi* resulted in localised pain and inflammation, which lasted for several hours, suggesting that this species is still in possession of a functional venom system able to cause muscle pain radiating up an entire arm lasting 12 hours (BG Fry, personal observations).

Interestingly, Type I α-neurotoxins, which were found to be extremely well conserved in most Australian elapids, accumulated a large number of hypermutable sites in *B. roperi*; particularly in regions that harbour residues that confer α-neurotoxins in other species the ability to target muscular α1-nicotinic acetylcholine receptors (nAChRs). Similarly, Type II α-neurotoxins in this species appeared to have poorly conserved functionally important residues, which confer Type II α-neurotoxins in other species the ability to target muscular α1 and neuronal α7 nAChRs. Thus, *B. roperi* Type I and II α-neurotoxins have likely lost their ability to target nAChRs of the prey. Hence, the observed pathogenesis could have been the result of other venom-components, including the relatively well-conserved Type III α-neurotoxins, or non-specific immunogenic reactions. In contrast, α-neurotoxin genes in other Australian elapids tend to accumulate variations largely in structurally and functionally unimportant regions. Hence, these regions are likely involved in mounting immunogenic reactions in the prey.

## 3. Experimental Section

### 3.1. Study Species

The widest possible phylogenetic and ecological diversity amongst the smaller forms of Australian elapid snake were chosen for this study. Unlike the larger, more commonly studied species of Australian elapid snakes, which typically have generalized diets, the species chosen for this study feed predominantly on other reptiles (or their eggs, in the case of *Brachyurophis roperi*) except for *Denisonia devisi*, which feeds almost exclusively on frogs [12,80,85,90,91,92,93,94,95]. As well as reptile and frog specialists, this study also examines species with divergent ecologies including: burrowing species (*Brachyurophis* *roperi* and *Vermicella annulata)*; a semi-arboreal species (*Hoplocephalus* bungaroides); ambush hunters (*Acanthophis wellsi* and *Denisonia devisi*); nocturnal foragers (*Cacophis squamulosus* and *Furina ornata*); arid zone inhabitants (*Acanthophis wellsi* and *Suta fasciata*); and marsh dwellers (*Echiopsis* curta and *Hemiaspis signata*). Also examined were anomalous and unstudied members of otherwise well studied genera: *Pseudonaja modesta* (the smallest *Pseudonaja,* a reptile specialist in a genus of generalists) and *Acanthophis wellsi* (an arid zone death adder). To date, no toxin sequences have previously been retrieved/published from any of the species examined in the present study. 

*Species and collection localities: Acanthophis wellsi*—Millstream, Western Australia; *Brachyurophis roperi*—Kununurra, Western Australia; *Cacophis squamulosus*—Mt Glorious, Queensland; *Denisonia devisi*—Glenmorgan, Queensland; *Echiopsis curta*—Perth, Western Australia; *Furina ornata*—Kununurra, Western Australia; *Hemiaspis signata*—Mt Glorious, Queensland; *Hoplocephalus bungaroides*—Sydney, New South Wales; *Pseudonaja modesta*—Sandstone, Western Australia; *Suta fasciata*—Sandstone, Western Australia. *Vermicella annulata*—Mt. Glorious, Queensland. All specimens were adult males.

### 3.2. Transcriptome Sequencing

Total RNA was extracted from venom glands using the standard TRIzol Plus method (Invitrogen). Extracts were enriched for mRNA using standard RNeasy mRNA mini kit (Qiagen) protocol. mRNA was reverse transcribed, fragmented and ligated to a unique 10-base multiplex identifier (MID) tag prepared using standard protocols and applied to one PicoTitrePlate (PTP) for simultaneous amplification and sequencing on a Roche 454 GS FLX+ Titanium platform (Australian Genome Research Facility). As each plate contained mRNA samples from multiple species, automated grouping and analysis of sample-specific MID reads informatically separated sequences from the other transcriptomes on the plates, which were then post-processed to remove low quality sequences before *de novo* assembly into contiguous sequences (contigs) using v 3.4.0.1 of the MIRA software program. 

Full assembly parameters are available in Supplementary File 1, assembly details in Supplementary Tables 2 and 3 and nucleotide sequences are available in Supplementary File 2 as well from Genbank: *Acanthophis wellsi* (GAGZ01000001-GAGZ01000032), *Brachyurophis roperi* (GAHA01000001-GAHA01000031), *Cacophis squamulosus* (GAHB01000001-GAHB01000034), *Denisonia devisi* (GAHC01000001-GAHC01000021), *Echiopsis curta* (GAHD01000001-GAHD01000032), *Furina ornate* (GAHE01000001-GAHE01000038), *Hemiaspis signata* (GAHF01000001-GAHF01000041), *Hoplocephalus bungaroides* (GAHG01000001-GAHG01000046), *Pseudonaja modesta* (GAHH01000001-GAHH01000073), *Suta fasciata* (GAHI01000001-GAHI01000020), and *Vermicella annulata* (GAHJ01000001-GAHJ01000024). All raw reads have been deposited in the NCBI Sequence Read Archive (http://www.ncbi.nlm.nih.gov/sra/) with the accession numbers of: SRR768900 *Acanthophis wellsi*; SRR768902 *Brachyurophis roperi*; SRR768909 *Cacophis squamulosus*; SRR768910 *Denisonia devisii*; SRR768911 *Echiopsis curta*; SRR768912 *Furina ornata*; SRR768914 *Hoplocephalus bungaroides*; SRR768915 *Pseudonaja modesta*; SRR768916 *Suta fasciata*; SRR768917 *Vermicella annulata.* Assembled contigs were processed using CLC Main Work Bench (CLC-Bio) and Blast2GO bioinformatic suite to provide Gene Ontology, BLAST and domain/Interpro annotation. The above analyses assisted in the rationalisation of the large numbers of assembled contigs into phylogenetic ‘groups’ for detailed phylogenetic analyses outlined below.

### 3.3. Phylogenetics

Phylogenetic analyses were performed to allow reconstruction of the molecular evolutionary history of each toxin type for which transcripts were bioinformatically recovered. Toxin sequences were identified by comparison of the translated DNA sequences with previously characterised toxins using a BLAST search of the UniProtKB protein database. Molecular phylogenetic analyses of toxin transcripts were conducted using the translated amino acid sequences. Comparative sequences from other venomous reptiles and physiological gene homologs identified from non-venom gland transcriptomes were included in each dataset as outgroup sequences. To minimize confusion, all sequences obtained in this study are referred to by their Genbank accession numbers (http://www.ncbi.nlm.nih.gov/sites/entrez?db=Nucleotide) and sequences from previous studies are referred to by their UniProtKB accession numbers (www.uniprot.org). Resultant sequence sets were aligned using CLC Mainbench. When presented as sequence alignments, the leader sequence is shown in lowercase and cysteines are highlighted in black. > and < indicate incomplete N/5’ or C/3’ ends, respectively and * used to indicate the end of a sequence. Datasets were analysed using Bayesian inference implemented on MrBayes, version 3.2.1 using lset rates=invgamma with prset aamodelpr=mixed, which enables the program to optimize between nine different amino acid substitution matrices. The analysis was performed by running a minimum of 1 × 10^7^ generations in four chains, and saving every 100th tree. The log-likelihood score of each saved tree was plotted against the number of generations to establish the point at which the log likelihood scores reached their asymptote, and the posterior probabilities for clades established by constructing a majority-rule consensus tree for all trees generated after completion of the burn-in phase. 

### 3.4. Selection Analyses

We evaluated the influence of natural selection on various types of three-finger toxins using maximum-likelihood models [96,97] implemented in CODEML of the PAML package of programs [98]. We employed site-specific models that estimate positive selection statistically as a non-synonymous-to-synonymous nucleotide-substitution rate ratio (ω) significantly greater than 1. We compared likelihood values for three pairs of models with different assumed ω distributions as no *a priori* expectation exists for the same: M0 (constant ω rates across all sites) *versus* M3 (allows ω to vary across sites within ‘*n*’ discrete categories, *n* ≥ 3); M1a (a model of neutral evolution) where all sites are assumed to be either under negative (ω < 1) or neutral selection (ω = 1) *versus* M2a (a model of positive selection) which in addition to the site classes mentioned for M1a, assumes a third category of sites; sites with ω > 1 (positive selection) and M7 (Beta) *versus* M8 (Beta and ω), and models that mirror the evolutionary constraints of M1 and M2 but assume that ω values are drawn from a beta distribution [99]. Only if the alternative models (M3, M2a and M8: allow sites with ω > 1) show a better fit in Likelihood Ratio Test (LRT) relative to their null models (M0, M1a and M7: do not allow sites ω > 1), are their results considered significant. LRT is estimated as twice the difference in maximum likelihood values between nested models and compared with the χ^2^ distribution with the appropriate degree of freedom-the difference in the number of parameters between the two models. The Bayes empirical Bayes (BEB) approach [100] was used to identify amino acids under positive selection by calculating the posterior probabilities that a particular amino acid belongs to a given selection class (neutral, conserved or highly variable). Sites with greater posterior probability (*PP* ≥ 95%) of belonging to the ‘ω > 1 class’ were inferred to be positively selected.

Single Likelihood Ancestor Counting (SLAC), Fixed-Effects Likelihood (FEL) and Random Effects Likelihood (REL) implemented in HyPhy [101] were employed to detect sites evolving under the influence of positive and negative selection. Fast, Unconstrained Bayesian AppRoximation (FUBAR) [102] was also employed to detect sites evolving under the influence of pervasive diversifying and purifying selection. Mixed Effects Model Evolution (MEME) [103] was used to efficiently detect episodically diversifying sites. To clearly depict the proportion of sites under different regimes of selection, an evolutionary fingerprint analysis was carried out using the evolutionary selection distance (ESD) algorithm [104] implemented in datamonkey Branch-sire REL tests [105] were employed to identify lineages evolving under episodic bursts of adaptive selection pressures. For all the aforementioned selection analyses, phylogenetic trees were built using the maximum-likelihood methodology implemented in PhyML 3.0 [106]. Node support was evaluated with 1,000 bootstrapping replicates.

### 3.5. Structural Analyses

To depict the natural selection pressures influencing the evolution of various three-finger toxins, we mapped the sites under positive selection on the homology models created using Phyre 2 webserver [107]. Pymol 1.3 [108] was used to visualize and generate the images of homology models. Consurf webserver [109] was used for mapping the evolutionary selection pressures on the three-dimensional homology models.

## 4. Conclusion

Whilst the findings of the present study have significantly increased our knowledge of the molecular evolution of Australian elapid venom, little can be ascertained about the specific roles of these toxins in the lifestyles of these snakes without further work being conducted. Thus, future work must be undertaken to characterise the venom proteomes of the 11 species examined in this study. As well as shedding light on methodological questions (e.g., whether or not a complex transcriptome always equals a complex proteome), proteomic characterisation of these venoms will allow us to further investigate correlations between diet and venom composition (*c.f.* [82]), gauge their relative toxicity in different animal models, and more accurately assess the potential danger of these snakes to human bite victims. Alongside proteomic characterisation, it is important that the extent and basis of any cross-reactivity of these venoms with currently available antivenoms and the snake venom detection kit (SVDK) [110] be ascertained.

Additional questions regarding the molecular evolution of these toxins remain to be answered, including the timing of the recruitment event of the fXaTx into the venom arsenal of Australian elapids. This question may be answered through the use of primer-driven PCR amplification on both the venom gland cDNA of *C. squamulosus* and *F. ornata*, as well as that of key outgroup species such as *Micropechis ikaheka.*

This study reinforces how little we know about the venoms of Australian elapids. Our major findings are of interest to the fields of whole organism evolutionary biology and protein evolution, and also highlight the hitherto untapped bioresource for drug design and development that the venoms of these neglected snakes represent. These findings also have direct implications for treatment of envenomed patients. Collectively, these findings strongly support the notion that the venoms of elapid snakes underwent a punctuated molecular evolution paralleling the explosive radiation of the snakes themselves subsequent to the colonisation of the largely snake free Australian continent 25 million years ago. We still have much to learn about the proteomic composition of the venoms of these snakes, the role of individual venom components in prey capture and the *in vitro* activities of these venom components. Furthermore, the toxin types recovered in this study should not be considered as the full-suite as this sampling may have only recovered the dominant forms. This study therefore provides baseline data for continuing investigations. More extensive sampling is likely to recover novel isoforms of toxin types identified to date as well as entirely new toxin classes. In addition, investigation of the relationship of the venom gland transcriptomes to the venom proteomes of these snakes may reveal translational variances similar to those that have been previously documented for other snakes

## Supplementary Files

Supplementary File 1 Supplementary (ZIP, 287 KB)

## References

[1] Fry B.G. (2005). From genome to "venome": molecular origin and evolution of the snake venom proteome inferred from phylogenetic analysis of toxin sequences and related body proteins. Genome Res..

[2] Nisani Z., Boskovic D.S., Dunbar S.G., Kelln W., Hayes W.K. (2012). Investigating the chemical profile of regenerated scorpion (*Parabuthus transvaalicus*) venom in relation to metabolic cost and toxicity. Toxicon.

[3] Fry B.G., Roelants K., Champagne D.E., Scheib H., Tyndall J.D., King G.F., Nevalainen T.J., Norman J.A., Lewis R.J., Norton R.S., Renjifo C., de la Vega R.C. (2009). The toxicogenomic multiverse: convergent recruitment of proteins into animal venoms. Annu. Rev. Genomics Hum. Genet..

[4] Casewell N.R., Wuster W., Vonk F.J., Harrison R.A., Fry B.G. (2013). Complex cocktails: the evolutionary novelty of venoms. Trends Ecol. Evol..

[5] Earl S.T., Masci P.P., de Jersey J., Lavin M.F., Dixon J. (2012). Drug development from Australian elapid snake venoms and the Venomics pipeline of candidates for haemostasis: Textilinin-1 (Q8008), Haempatch (Q8009) and CoVase (V0801). Toxicon.

[6] Vetter I., Davis J.L., Rash L.D., Anangi R., Mobli M., Alewood P.F., Lewis R.J., King G.F. (2011). Venomics: a new paradigm for natural products-based drug discovery. Amino Acids.

[7] Vink S., Jin A.H., Poth K.J., Head G.A., Alewood P.F. (2012). Natriuretic peptide drug leads from snake venom. Toxicon.

[8] Vonk F.J., Admiraal J.F., Jackson K., Reshef R., de Bakker M.A., Vanderschoot K., van den Berge I., van Atten M., Burgerhout E., Beck A., Mirtschin P.J., Kochva E., Witte F., Fry B.G., Woods A.E., Richardson M.K. (2008). Evolutionary origin and development of snake fangs. Nature.

[9] Wilson S., Swan G. (2010). A Complete Guide to Reptiles of Australia.

[10] Sutherland S.K., Tibbals J. (2001). Australian Animal Toxins: The Creatures, Their Toxins and Care of the Poisoned Patient.

[11] Pycroft K., Fry B.G., Isbister G.K., Kuruppu S., Lawrence J., Ian Smith A., Hodgson W.C. (2012). Toxinology of venoms from five Australian lesser known elapid snakes. Basic Clin. Pharmacol. Toxicol..

[12] Shine R. (1983). Food-habits and reproductive-biology of Australian elapid snakes of the genus *Denisonia*. J. Herpetol..

[13] Birrell G.W., Earl S.T., Wallis T.P., Masci P.P., de Jersey J., Gorman J.J., Lavin M.F. (2007). The diversity of bioactive proteins in Australian snake venoms. Mol. Cell. Proteomics.

[14] Fry B.G. (1999). Structure-function properties of venom components from Australian elapids. Toxicon.

[15] Ching A.T., Rocha M.M., Paes Leme A.F., Pimenta D.C., de Fatima D.F.M., Serrano S.M., Ho P.L., Junqueira-de-Azevedo I.L. (2006). Some aspects of the venom proteome of the Colubridae snake *Philodryas olfersii* revealed from a Duvernoy's (venom) gland transcriptome. FEBS Lett..

[16] Fry B.G., Roelants K., Norman J.A. (2009). Tentacles of venom: toxic protein convergence in the Kingdom Animalia. J. Mol. Evol..

[17] Fry B.G., Roelants K., Winter K., Hodgson W.C., Griesman L., Kwok H.F., Scanlon D., Karas J., Shaw C., Wong L., Norman J.A. (2010). Novel venom proteins produced by differential domain-expression strategies in beaded lizards and gila monsters (genus *Heloderma*). Mol. Biol. Evol..

[18] Fry B.G., Scheib H., de L M Junqueira de Azevedo I., Silva D.A., Casewell N.R. (2012). Novel transcripts in the maxillary venom glands of advanced snakes. Toxicon.

[19] Fry B.G., Scheib H., van der Weerd L., Young B., McNaughtan J., Ramjan S.F., Vidal N., Poelmann R.E., Norman J.A. (2008). Evolution of an arsenal: structural and functional diversification of the venom system in the advanced snakes (Caenophidia). Mol. Cell. Proteomics.

[20] Fry B.G., Undheim E.A., Ali S.A., Jackson T.N., Debono J., Scheib H., Ruder T., Morgenstern D., Cadwallader L., Whitehead D., Nabuurs R., van der Weerd L., Vidal N., Roelants K., Hendrikx I., Gonzalez S.P., Koludarov I., Jones A., King G.F., Antunes A., Sunagar K. (2013). Squeezers and leaf-cutters: differential diversification and degeneration of the venom system in toxicoferan reptiles. Mol. Cell. Proteomics.

[21] Fry B.G., Vidal N., Norman J.A., Vonk F.J., Scheib H., Ramjan S.F., Kuruppu S., Fung K., Hedges S.B., Richardson M.K., Hodgson W.C., Ignjatovic V., Summerhayes R., Kochva E. (2006). Early evolution of the venom system in lizards and snakes. Nature.

[22] Fry B.G., Winter K., Norman J.A., Roelants K., Nabuurs R.J., van Osch M.J., Teeuwisse W.M., van der Weerd L., McNaughtan J.E., Kwok H.F., Scheib H., Greisman L., Kochva E., Miller L.J., Gao F., Karas J., Scanlon D., Lin F., Kuruppu S., Shaw C., Wong L., Hodgson W.C. (2010). Functional and structural diversification of the Anguimorpha lizard venom system. Mol. Cell. Proteomics.

[23] Fry B.G., Wroe S., Teeuwisse W., van Osch M.J., Moreno K., Ingle J., McHenry C., Ferrara T., Clausen P., Scheib H., Winter K.L., Greisman L., Roelants K., van der Weerd L., Clemente C.J., Giannakis E., Hodgson W.C., Luz S., Martelli P., Krishnasamy K., Kochva E., Kwok H.F., Scanlon D., Karas J., Citron D.M., Goldstein E.J., McNaughtan J.E., Norman J.A. (2009). A central role for venom in predation by *Varanus komodoensis* (Komodo Dragon) and the extinct giant *Varanus* (*Megalania*) *priscus*. Proc. Natl. Acad. Sci. USA.

[24] Junqueira-de-Azevedo I.L., Ching A.T., Carvalho E., Faria F., Nishiyama M.Y., Ho P.L., Diniz M.R. (2006). Lachesis muta (Viperidae) cDNAs reveal diverging pit viper molecules and scaffolds typical of cobra (Elapidae) venoms: implications for snake toxin repertoire evolution. Genetics.

[25] Terrat Y., Sunagar K., Fry B.G., Jackson T.N., Scheib H., Fourmy R., Verdenaud M., Blanchet G., Antunes A., Ducancel F. (2013). *Atractaspis aterrima* Toxins: The First Insight into the Molecular Evolution of Venom in Side-Stabbers. Toxins.

[26] Wagstaff S.C., Harrison R.A. (2006). Venom gland EST analysis of the saw-scaled viper, *Echis ocellatus*, reveals novel alpha9beta1 integrin-binding motifs in venom metalloproteinases and a new group of putative toxins, renin-like aspartic proteas. Gene.

[27] Fry B.G., Wuster W., Kini R.M., Brusic V., Khan A., Venkataraman D., Rooney A.P. (2003). Molecular evolution and phylogeny of elapid snake venom three-finger toxins. J. Mol. Evol..

[28] St Pierre L., Fischer H., Adams D.J., Schenning M., Lavidis N., de Jersey J., Masci P.P., Lavin M.F. (2007). Distinct activities of novel neurotoxins from Australian venomous snakes for nicotinic acetylcholine receptors. Cell. Mol. Life Sci..

[29] Sunagar K., Jackson T.N., Undheim E.A., Ali S.A., Antunes A., Fry B.G. (2013). Three-Fingered RAVERs: Rapid Accumulation of Variations in Exposed Residues of Snake Venom Toxins. Toxins.

[30] Gong N., Armugam A., Jeyaseelan K. (1999). Postsynaptic short-chain neurotoxins from Pseudonaja textilis. cDNA cloning, expression and protein characterization. Eur. J. Biochem..

[31] Gong N., Armugam A., Jeyaseelan K. (2000). Molecular cloning, characterization and evolution of the gene encoding a new group of short-chain alpha-neurotoxins in an Australian elapid, *Pseudonaja textilis*. FEBS Lett..

[32] Brust A., Sunagar K., Undheim E.A., Vetter I., Yang D.C., Casewell N.R., Jackson T.N., Koludarov I., Alewood P.F., Hodgson W.C., Lewis R.J., King G.F., Antunes A., Hendrikx I., Fry B.G. (2013). Differential evolution and neofunctionalization of snake venom metalloprotease domains. Mol. Cell. Proteomics.

[33] Koludarov I., Sunagar K., Undheim E.A., Jackson T.N., Ruder T., Whitehead D., Saucedo A.C., Mora G.R., Alagon A.C., King G., Antunes A., Fry B.G. (2012). Structural and molecular diversification of the Anguimorpha lizard mandibular venom gland system in the arboreal species *Abronia graminea*. J. Mol. Evol..

[34] Kozminsky-Atias A., Zilberberg N. (2012). Molding the business end of neurotoxins by diversifying evolution. FASEB J..

[35] Low D.H., Sunagar K., Undheim E.A., Ali S.A., Alagon A.C., Ruder T., Jackson T.N., Pineda Gonzalez S., King G.F., Jones A., Antunes A., Fry B.G. (2013). Dracula's children: Molecular evolution of vampire bat venom. J. Proteomics.

[36] Ruder T., Sunagar K., Undheim E.A., Ali S.A., Wai T.C., Low D.H., Jackson T.N., King G.F., Antunes A., Fry B.G. (2013). Molecular phylogeny and evolution of the proteins encoded by coleoid (cuttlefish, octopus, and squid) posterior venom glands. J. Mol. Evol..

[37] Sunagar K., Johnson W.E., O'Brien S.J., Vasconcelos V., Antunes A. (2012). Evolution of CRISPs associated with toxicoferan-reptilian venom and mammalian reproduction. Mol. Biol. Evol..

[38] Sunagar K., Fry B.G., Jackson T.N.W., Casewell N.R., Undheim E.A.B., Vidal N., Ali S.A., King G.F., Vasudevan K., Vasconcelos V., Antunes A. (2013). Molecular Evolution of Vertebrate Neurotrophins: Co-Option of the Highly Conserved Nerve Growth Factor Gene into the Advanced Snake Venom Arsenal. PLoS One.

[39] Tian C., Yuan Y., Zhu S. (2008). Positively selected sites of scorpion depressant toxins: possible roles in toxin functional divergence. Toxicon.

[40] Zhu S., Bosmans F., Tytgat J. (2004). Adaptive evolution of scorpion sodium channel toxins. J. Mol. Evol..

[41] Xu Q., Wu X.F., Xia Q.C., Wang K.Y. (1999). Cloning of a galactose-binding lectin from the venom of Trimeresurus stejnegeri. Biochem. J..

[42] Earl S.T., Robson J., Trabi M., de Jersey J., Masci P.P., Lavin M.F. (2011). Characterisation of a mannose-binding C-type lectin from *Oxyuranus scutellatus* snake venom. Biochimie.

[43] Fry B.G., Wickramaratana J.C., Lemme S., Beuve A., Garbers D., Hodgson W.C., Alewood P. (2005). Novel natriuretic peptides from the venom of the inland taipan (*Oxyuranus microlepidotus*): isolation, chemical and biological characterisation. Biochem. Biophys. Res. Commun..

[44] Schweitz H., Vigne P., Moinier D., Frelin C., Lazdunski M. (1992). A new member of the natriuretic peptide family is present in the venom of the green mamba (*Dendroaspis angusticeps*). J. Biol. Chem..

[45] Camargo A.C., Ianzer D., Guerreiro J.R., Serrano S.M. (2012). Bradykinin-potentiating peptides: beyond captopril. Toxicon.

[46] Vonk F.J., Jackson K., Doley R., Madaras F., Mirtschin P.J., Vidal N. (2011). Snake venom: From fieldwork to the clinic: Recent insights into snake biology, together with new technology allowing high-throughput screening of venom, bring new hope for drug discovery. Bioessays.

[47] St Pierre L., Flight S., Masci P.P., Hanchard K.J., Lewis R.J., Alewood P.F., de Jersey J., Lavin M.F. (2006). Cloning and characterisation of natriuretic peptides from the venom glands of Australian elapids. Biochimie.

[48] St Pierre L., Woods R., Earl S., Masci P.P., Lavin M.F. (2005). Identification and analysis of venom gland-specific genes from the coastal taipan (*Oxyuranus scutellatus*) and related species. Cell. Mol. Life Sci..

[49] Akashi Y.J., Springer J., Lainscak M., Anker S.D. (2007). Atrial natriuretic peptide and related peptides. Clin. Chem. Lab. Med..

[50] Amininasab M., Elmi M.M., Endlich N., Endlich K., Parekh N., Naderi-Manesh H., Schaller J., Mostafavi H., Sattler M., Sarbolouki M.N., Muhle-Goll C. (2004). Functional and structural characterization of a novel member of the natriuretic family of peptides from the venom of *Pseudocerastes persicus*. FEBS Lett..

[51] Fry B.G., Wuster W. (2004). Assembling an arsenal: origin and evolution of the snake venom proteome inferred from phylogenetic analysis of toxin sequences. Mol. Biol. Evol..

[52] Chen H.H., Lainchbury J.G., Burnett J.C. (2002). Natriuretic peptide receptors and neutral endopeptidase in mediating the renal actions of a new therapeutic synthetic natriuretic peptide dendroaspis natriuretic peptide. J. Am. Coll. Cardiol..

[53] Da Silva S.L., Dias-Junior C.A., Baldasso P.A., Damico D.C., Carvalho B.M., Garanto A., Acosta G., Oliveira E., Albericio F., Soares A.M., Marangoni S., Resende R.R. (2012). Vascular effects and electrolyte homeostasis of the natriuretic peptide isolated from *Crotalus oreganus abyssus* (North American Grand Canyon rattlesnake) venom. Peptides.

[54] Quinton L., Gilles N., Smargiasso N., Kiehne A., De Pauw E. (2011). An unusual family of glycosylated peptides isolated from *Dendroaspis angusticeps* venom and characterized by combination of collision induced and electron transfer dissociation. J. Am. Soc. Mass Spectrom..

[55] Utkin Y.N., Weise C., Kasheverov I.E., Andreeva T.V., Kryukova E.V., Zhmak M.N., Starkov V.G., Hoang N.A., Bertrand D., Ramerstorfer J., Sieghart W., Thompson A.J., Lummis S.C., Tsetlin V.I. (2012). Azemiopsin from *Azemiops feae* viper venom, a novel polypeptide ligand of nicotinic acetylcholine receptor. J. Biol. Chem..

[56] Kuruppu S., Fry B.G., Hodgson W.C. (2005). Presynaptic neuromuscular activity of venom from the brown-headed snake (*Glyphodon tristis*). Toxicon.

[57] Fry B.G., Wuster W., Ryan Ramjan S.F., Jackson T., Martelli P., Kini R.M. (2003). Analysis of Colubroidea snake venoms by liquid chromatography with mass spectrometry: evolutionary and toxinological implications. Rapid Commun. Mass Spectrom..

[58] Kuruppu S., Robinson S., Hodgson W.C., Fry B.G. (2007). The *in vitro* neurotoxic and myotoxic effects of the venom from the *Suta* genus (curl snakes) of elapid snakes. Basic Clin. Pharmacol. Toxicol..

[59] Fohlman J., Lind P., Eaker D. (1977). Taipoxin, an extremely potent presynaptic snake venom neurotoxin. Elucidation of the primary structure of the acidic carbohydrate-containing taipoxin-subunit, a prophospholipase homolog. FEBS Lett..

[60] Blacklow B., Escoubas P., Nicholson G.M. (2010). Characterisation of the heterotrimeric presynaptic phospholipase A(2) neurotoxin complex from the venom of the common death adder (*Acanthophis antarcticus*). Biochem. Pharmacol..

[61] Blacklow B., Konstantakopoulos N., Hodgson W.C., Nicholson G.M. (2010). Presence of presynaptic neurotoxin complexes in the venoms of Australo-Papuan death adders (*Acanthophis* spp.). Toxicon.

[62] Chaisakul J., Konstantakopoulos N., Smith A.I., Hodgson W.C. (2010). Isolation and characterisation of P-EPTX-Ap1a and P-EPTX-Ar1a: pre-synaptic neurotoxins from the venom of the northern (*Acanthophis praelongus*) and Irian Jayan (*Acanthophis rugosus*) death adders. Biochem. Pharmacol..

[63] Chaisakul J., Parkington H.C., Isbister G.K., Konstantakopoulos N., Hodgson W.C. (2013). Differential myotoxic and cytotoxic activities of pre-synaptic neurotoxins from Papuan taipan (*Oxyuranus scutellatus*) and Irian Jayan death adder (*Acanthophis rugosus*) venoms. Basic Clin. Pharmacol. Toxicol..

[64] Fry B.G., Wickramaratna J.C., Hodgson W.C., Alewood P.F., Kini R.M., Ho H., Wuster W. (2002). Electrospray liquid chromatography/mass spectrometry fingerprinting of *Acanthophis* (death adder) venoms: taxonomic and toxinological implications. Rapid Commun. Mass Spectrom..

[65] Doley R., Tram N.N., Reza M.A., Kini R.M. (2008). Unusual accelerated rate of deletions and insertions in toxin genes in the venom glands of the pygmy copperhead (*Austrelaps labialis*) from Kangaroo island. BMC Evol. Biol..

[66] Marlor C.W., Delaria K.A., Davis G., Muller D.K., Greve J.M., Tamburini P.P. (1997). Identification and cloning of human placental bikunin, a novel serine protease inhibitor containing two Kunitz domains. J. Biol. Chem..

[67] Doley R., Pahari S., Reza M.A., Mackessy S.P., Kini R.M. (2010). The Gene Structure and Evolution of ku-wap-fusin (Kunitz Waprin Fusion Protein), a Novel Evolutionary Intermediate of the Kunitz Serine Protease Inhibitors and Waprins from *Sistrurus catenatus* (Massasauga Rattlesnake) Venom Glands. Open Evol.J..

[68] St Pierre L., Earl S.T., Filippovich I., Sorokina N., Masci P.P., De Jersey J., Lavin M.F. (2008). Common evolution of waprin and kunitz-like toxin families in Australian venomous snakes. Cell. Mol. Life Sci..

[69] Francischetti I.M., My-Pham V., Harrison J., Garfield M.K., Ribeiro J.M. (2004). *Bitis. gabonica* (Gaboon viper) snake venom gland: toward a catalog for the full-length transcripts (cDNA) and proteins. Gene.

[70] Nair D.G., Fry B.G., Alewood P., Kumar P.P., Kini R.M. (2007). Antimicrobial activity of omwaprin, a new member of the waprin family of snake venom proteins. Biochem. J..

[71] Isbister G.K., Dawson A.H., Whyte I.M. (2002). Two cases of bites by the black-bellied swamp snake (*Hemiaspis signata*). Toxicon.

[72] Isbister G.K., White J., Currie B.J., O'Leary M.A., Brown S.G. (2011). Clinical effects and treatment of envenoming by *Hoplocephalus* spp. snakes in Australia: Australian Snakebite Project (ASP-12). Toxicon.

[73] White J., Williams V., Passehl J.H. (1987). The five-ringed brown snake, Pseudonaja modesta (Gunther): report of a bite and comments on its venom. Med. J. Aust..

[74] Reza M.A., Minh Le T.N., Swarup S., Manjunatha Kini R. (2006). Molecular evolution caught in action: gene duplication and evolution of molecular isoforms of prothrombin activators in *Pseudonaja textilis* (brown snake). J. Thromb. Haemost..

[75] Sanders K.L., Lee M.S., Leys R., Foster R., Keogh J.S. (2008). Molecular phylogeny and divergence dates for Australasian elapids and sea snakes (Hydrophiinae): evidence from seven genes for rapid evolutionary radiations. J. Evol. Biol..

[76] Isbister G.K., Scorgie F.E., O'Leary M.A., Seldon M., Brown S.G., Lincz L.F. (2010). Factor deficiencies in venom-induced consumption coagulopathy resulting from Australian elapid envenomation: Australian Snakebite Project (ASP-10). J. Thromb. Haemost..

[77] Fry B.G., Casewell N.R., Wuster W., Vidal N., Young B., Jackson T.N. (2012). The structural and functional diversification of the Toxicofera reptile venom system. Toxicon.

[78] Filippovich I., Sorokina N., St Pierre L., Flight S., de Jersey J., Perry N., Masci P.P., Lavin M.F. (2005). Cloning and functional expression of venom prothrombin activator protease from *Pseudonaja textilis* with whole blood procoagulant activity. Br. J. Haematol..

[79] Bos M.H., Boltz M., St Pierre L., Masci P.P., de Jersey J., Lavin M.F., Camire R.M. (2009). Venom factor V from the common brown snake escapes hemostatic regulation through procoagulant adaptations. Blood.

[80] Shine R. (1989). Constraints, allometry, and adaptation-food-habits and reproductive-biology of australian brownsnakes (*Pseudonaja*, Elapidae). Herpetologica.

[81] Kulkeaw K., Chaicumpa W., Sakolvaree Y., Tongtawe P., Tapchaisri P. (2007). Proteome and immunome of the venom of the Thai cobra, *Naja kaouthia*. Toxicon.

[82] Daltry J.C., Wuster W., Thorpe R.S. (1996). Diet and snake venom evolution. Nature.

[83] Gibbs H.L., Sanz L., Sovic M.G., Calvete J.J. (2013). Phylogeny-based comparative analysis of venom proteome variation in a clade of rattlesnakes (*Sistrurus* sp.). PLoS One.

[84] Pawlak J., Mackessy S.P., Fry B.G., Bhatia M., Mourier G., Fruchart-Gaillard C., Servent D., Menez R., Stura E., Menez A., Kini R.M. (2006). Denmotoxin, a three-finger toxin from the colubrid snake *Boiga dendrophila* (Mangrove Catsnake) with bird-specific activity. J. Biol. Chem..

[85] Shine R. (1980). Reproduction, feeding and growth in the australian burrowing snake *Vermicella annulata*. J. Herpetol..

[86] Durban J., Perez A., Sanz L., Gomez A., Bonilla F., Rodriguez S., Chacon D., Sasa M., Angulo Y., Gutierrez J.M., Calvete J.J. (2013). Integrated "omics" profiling indicates that miRNAs are modulators of the ontogenetic venom composition shift in the Central American rattlesnake, *Crotalus simus simus*. BMC Genomics.

[87] Li M., Fry B.G., Kini R.M. (2005). Eggs-only diet: its implications for the toxin profile changes and ecology of the marbled sea snake (*Aipysurus eydouxii*). J. Mol. Evol..

[88] Li M., Fry B.G., Kini R.M. (2005). Putting the brakes on snake venom evolution: the unique molecular evolutionary patterns of *Aipysurus eydouxii* (Marbled sea snake) phospholipase A2 toxins. Mol. Biol. Evol..

[89] Scanlon J.D., Shine R. (1988). Dentition and diet in snakes - adaptations to oophagy in the Australian elapid genus *Simoselaps*. J. Zool..

[90] Shine R. (1980). Comparative ecology of 3 australian snake species of the genus *Cacophis* (Serpentes, Elapidae). Copeia.

[91] Shine R. (1981). Ecology of Australian elapid snakes of the genera *Furina* and *Glyphodon*. J. Herpetol..

[92] Shine R. (1983). Arboreality in snakes: ecology of the Australian elapid genus *Hoplocephalus*. Copeia.

[93] Shine R. (1984). Reproductive-biology and food-habits of the Australian elapid snakes of the genus *Cryptophis*. J. Herpetol..

[94] Shine R. (1987). Food-habits and reproductive-biology of australian snakes of the genus *Hemiaspis* (Elapidae). J. Herpetol..

[95] Shine R. (1988). Food-habits and reproductive-biology of small australian snakes of the genera *Unechis* and *Suta* (elapidae). J. Herpetol..

[96] Goldman N., Yang Z. (1994). A codon-based model of nucleotide substitution for protein-coding DNA sequences. Mol. Biol. Evol..

[97] Yang Z. (1998). Likelihood ratio tests for detecting positive selection and application to primate lysozyme evolution. Mol. Biol. Evol..

[98] Yang Z. (2007). PAML 4: phylogenetic analysis by maximum likelihood. Mol. Biol. Evol..

[99] Nielsen R., Yang Z. (1998). Likelihood models for detecting positively selected amino acid sites and applications to the HIV-1 envelope gene. Genetics.

[100] Yang Z., Wong W.S., Nielsen R. (2005). Bayes empirical bayes inference of amino acid sites under positive selection. Mol. Biol. Evol..

[101] Pond S.L., Frost S.D., Muse S.V. (2005). HyPhy: hypothesis testing using phylogenies. Bioinformatics.

[102] Murrell B., Moola S., Mabona A., Weighill T., Sheward D., Kosakovsky Pond S.L., Scheffler K. (2013). FUBAR: a fast, unconstrained bayesian approximation for inferring selection. Mol. Biol. Evol..

[103] Murrell B., Wertheim J.O., Moola S., Weighill T., Scheffler K., Kosakovsky Pond S.L. (2012). Detecting individual sites subject to episodic diversifying selection. PLoS Genet..

[104] Pond S.L., Scheffler K., Gravenor M.B., Poon A.F., Frost S.D. (2010). Evolutionary fingerprinting of genes. Mol. Biol. Evol..

[105] Kosakovsky Pond S.L., Murrell B., Fourment M., Frost S.D., Delport W., Scheffler K. (2011). A random effects branch-site model for detecting episodic diversifying selection. Mol. Biol. Evol..

[106] Guindon S., Dufayard J.F., Lefort V., Anisimova M., Hordijk W., Gascuel O. (2010). New algorithms and methods to estimate maximum-likelihood phylogenies: assessing the performance of PhyML 3.0. Syst. Biol..

[107] Kelley L.A., Sternberg M.J. (2009). Protein structure prediction on the Web: a case study using the Phyre server. Nat. Protoc..

[108] DeLano W.L. (2002). The PyMOL Molecular Graphics System.

[109] Armon A., Graur D., Ben-Tal N. (2001). ConSurf: an algorithmic tool for the identification of functional regions in proteins by surface mapping of phylogenetic information. J. Mol. Biol..

[110] Steuten J., Winkel K., Carroll T., Williamson N.A., Ignjatovic V., Fung K., Purcell A.W., Fry B.G. (2007). The molecular basis of cross-reactivity in the Australian Snake Venom Detection Kit (SVDK). Toxicon.

