# Supplementary material for: Venom Down Under: Dynamic Evolution of Australian Elapid Snake Toxins
Source: Toxins (Basel). 2013 Dec 18;5(12):2621–55. doi: 10.3390/toxins5122621 (PMC3873703; doi:10.3390/toxins5122621)
Supplement: Supplementary File 1 — Supplementary (ZIP, 287 KB) [file toxins-05-02621-s001.zip › Supplementary File 2 nucleotides.pdf]

<u>TOXIN TYPES</u>
3FTX = Three finger toxin
ACN = acetylcholinesterase
cystatin
CVF = cobra venom factor
fV = factor V
fX = factor X
KP = kunitz peptide
LP = lectin protein
NGF = nerve growth factor
NP = natriuretic peptide
PLA2 = phospholipase A_2
SVMP = snake venom metalloprotease
VEGF = vascular endothelial growth factor
WP = waprin peptide

>GAGZ00000000.1|GAGZ01000000 TSA: Acanthophis wellsei, transcriptome shotgun assembly, transcriptome shotgun assembly

>GAGZ01000001 Acanthophis wellsi 3FTx-Aca-18 mRNA sequence
ATGAAAACTCTGCTGCTGACCTTGGTGGTGGTGACAATCCTGTGCCTGGACTTAGGATACACCATGACAT
GCTTCAAAACACCTTATGTTAAATCCGAGCCTTGTCCACCTGGGGAGAATGTATGCGTTACAAAGACTTG
GTGTGATGCCTTTTGTAGCATCAGAGGAAAGGTAATCGAACTGGGATGTGCTGCTACTTGCCCTCCAGCG
GAGCCAAAAAAGGATATTAAATGTTGCTCAACAGACAATTGCAACACACATCCGTAA

>GAGZ01000002 Acanthophis wellsi 3FTx-Aca-27 mRNA sequence
ATGAAAACTCTGCTGCTGACCTTGGTGGTGGTGACAATCCTGTGCCTGGACTTAGGTGACAGCGTGATAT
GCTACCTGGGATATAACAACCCTCAGACTTGTCCACCTGGGGAGAATGTATGCTTTACAAGGACTTGGTG
TGATGCCTTTTGTAGCAGCAGAGGAAAGGTAGTCGAACTGGGATGTGCTGCTACTTGCCCTATACCGAAG
TCCTATCAGGATGTTAAATGTTGCTCAACAGACAAGTGCAACCCATTTCCAGTACGTCCTCGACGGAGGC
CCTAA

>GAGZ01000003 Acanthophis wellsi 3FTx-Aca-30 mRNA sequence
ATGAAAACTCTGCTGCTGACCTTGGTGGTGGTGACAATCCTGTGCCTGGACTTAGGTGACAGCGTGATAT
GCTACCTGGGATATAACAACCCTCAGACTTGTCCACCTGGGGAGAATGTATGCTTTACAAGGACTTGGTG
TGATTCCTTTTGTAGCAGCAGAGGAAAGGTAGTCGAACTGGGATGTGCTGCTACTTGCCCTATACCGAAG
TCCTATCAGGATGTTAAATGTTGCTCAACAGACAAGTGCAACCCATTTCCAGTACGTCCTCGACGGAGGC
CCTAA

>GAGZ01000004 Acanthophis wellsi 3FTx-Aca-53 mRNA sequence
ATGAAAACTCTGCTGCTGACCTTGGTGGTGGTGACAATCGTGTGCCTGGACTTAGGATACACCAAGACAT
GCTTCAAAACACCTTATGTTAAATCCGAGCCTTGTCCACCTGGGCAGGACCTATGCTATACAAAGACTTG
GTGTGATGCCTTTTGTAGCATCAGAGGAAAGGTAATCGAACTGGGATGTGCTGCTACTTGCCCTCCAGCG
GAGCCAAAAAAGGATATTAAATGTTGCTCAACAGACAATTGCAACACACATCCGTAA

>GAGZ01000005 Acanthophis wellsi 3FTx-Aca-54 mRNA sequence
ATGAAAACTCTGCTGCTGACCTTGGTGGTGGTGACAATCGTGTGCCTGGACTTAGGATACACCAAGACAT
GCTTCAAAACACCTTATGTTAAATCCGAGCCTTGTCCACCTGGGCAGGACCTATGCTATACAAAGACTTG
GTGTGATGCCTTTTGTAGCATCAGAGGAAAGGTAATCGAACTGGGATGTGCTGCTACTTGCCCTCCAGCG
GAGCCAAAAAAGGATATTAAATGTTGCTCAACAGACAATTGCAACACACATCCGTAA

>GAGZ01000006 Acanthophis wellsi 3FTx-Aca-55 mRNA sequence
ATGAAAACTCTGCTGCTGACCTTGGTGGTGGTGACAATCCTGTGCCTGGACGTAGGTGACAGCGTGATAT
GCTACCTGGGATATAATTACGCTCAGCCTTGTCCACCTGGGGAGAATGTATGCTTTACAAAGACTTGGTG
TGATGGTCGATGTCCCCAACTAGGAAAGCGAGTCGAAATGGGATGTGCTGCTACTTGCCCCAAAGTGAAG
CGCGGTGTGGATATTAAATGTTGCTCAACAGACAAGTGCAACCCATCCATTAAGTCCTCGAGGGAAGC
CCTAA

>GAGZ01000007 Acanthophis wellsi 3FTx-Aca-57 mRNA sequence
ATGAAAACTCTGCTGCTGACCTTGGTGGTGGTGACAATCCTGTGCCTGGACGTAGGTGAAAGCGTGATAT
GCTACCTGGGATATAACAACCCTCAGACTTGTCCACCTGGGGAGAATGTATGCTTTACAAAGACTTGGTG
TGATGCTCGGTGTAGCCAACTAGGAAAGCGAGTCGAAATGGGATGTGCTGCTACTTGCCCCAAAGTGAAG
CCCGGTGTGGATATTAAATGTTGCTCAACAGACAAGTGCAACCCATTTCCGAAAACGACACCTCCTTGGG
AACGTCCTCGAGGGAAGCCCTAA

>GAGZ01000008 Acanthophis wellsi 3FTx-Aca-62 mRNA sequence
ATGAAAACTCTGCTGCTGACCTTGGTGGTGGTGACAATCGTGTGCCTGGACTTAGGTGAAAGCGTGATAT
GCTACCTGGGATATAATTATGCTCAGACTTGTCCACCTGGGGAGAATGTATGCTTTACAAAGACTTGGTG
TGATGCTCGGTGTGGCCTTCTAGGAAAGCGAGTTGAAATGGGATGTGCTGCTACTTGCCCCAAAGTGAAA
CCCGGTGTGGATATTAAATGTTGCGAAACAGACAAGTGCAACCCATTTCCGAAAACGACACCTCCTTGGG
AACGTCCTCGAGGGAAGCCCTAA

>GAGZ01000009 Acanthophis wellsi 3FTx-Aca-84 mRNA sequence
ATGAAAACTCTGCTGCTGACCTTGGTGGTGGTGACAATCCTGTGCCTGGACGTAGGTGACAGCGTGATAT
GCTACCTGGGATATAATTACGCTCAGCCTTGTCCACCTGGGGAGAATGTATGCTTTACAAAGACTTGGTG
TGATGGTCGATGTCCCCAACTAGGAAAGCGAGTCGAGATGGGATGTGCTGCTACTTGCCCCAAAGTGAAG
CGCGGTGTGGATATTAAATGTTGCTCAACAGACAAGTGCAACCCATTTCCATTAAGTCCTCGAGGGAAGC
CCTAA

>GAGZ01000010 Acanthophis wellsi 3FTx-Aca-85 mRNA sequence
ATGAAAACTCTGCTGCTGACCTTGGTGGTGGTGACAATCCTGTGCCTGGACGTAGGTGACAGCGTGATAT
GCTACCTGGGATATAATTACGCTCAGCCTTGTCCACCTGGGGAGAATGTATGCTTTACAAAGACTTGGTG
TGATGGTCGATGTCCCCAACTAGGAAAGCGAGTCGAAATGGGATGTGCTGCTACTTGCCCCAAAGTGAAG
CGCGGTGTGGATATTAAATGTTGCTCAACAGACAAGTGCAACCCATTTCCATTAAGTCCTCGAGGGAAGC
CCTAA

>GAGZ01000011 Acanthophis wellsi 3FTx-Aca-108 mRNA sequence
ATGAAAACTCTGCTGCTGACCTTGGTGGTGGTGACAATCGTGTGCCTGGACTTAGGATACACCATGCAAT
GTTACAACCACCCGTTATTGCAAGATAAAACCATTATAACTTGTGCTTTTGGGGTGAGCTCTTGCTTTAA
AAAGACTTGGAAAGTTCAACGTGAAACTATGATTGAAAGGGGATGTGGTTGCCCTAACGTGAAGCCTGGT
GTTAGACTTACATGTTGCAAAACAGACAAATGCAACAATTAG

>GAGZ01000012 Acanthophis wellsi 3FTx-Aca-112 mRNA sequence
ATGAAAACTCTGCTGCTGACCTTGGTGGTGGTGACAATCGTGTGCCTGGACTTAGTATACACCATGCAAT
GTTGCAACCAACAGTCATCGCAACCTAAAACCACTACAACTTGTCCAGGTGGGGTGAGCTCTTGCTATAA
AAAGACTTGGAGAGATCATCGTGGACTTAGAATTGAAAGGGGATGTGGTTGCCCTCACGTGAAGCCTGGT
ATTTGGCTTCTTTGTTGCAAAACAGACGAATGCAACAATTAG

>GAGZ01000013 Acanthophis wellsi 3FTx-Aca-118 mRNA sequence
ATGAAAACTCTGCTGCTGACCTTGGTGGTGGTGACAATCGTGTGCCTGGACTTAGTATACACCATGCAAT
GTTGCAACCAACAGTCATCGCAACCTAAAACCACTACAACTTGTCCAGGTGGGGTGAGCTCTTGCTATAA
AAAGACTTGGAGTGATCATCGTGGATCTAGAACTGAAAGGGGATGCGGTTGCCCTCACGTGAAGCCCGGT
ATTACACTTATTTGTTGCAAAACAGATGAATGCAACAATTAG

>GAGZ01000014 Acanthophis wellsi ACN-Aca-1 mRNA sequence
ATGAGCGTGCCTCACGCCAACGACATCGCCACCGAGGCCGTGGTGATGCAGTACACTGACTGGCAGGACC
AGGACAACGGGGAGAAGAACAGGGAGGCCCTGGACGACATCGTGGGGGACCACAACATCATCTGCCCGGT
TGTGCAGTTCGCCAATGACTACGCCAAGCGCAAGAACAAGGTGTACGCCTACCTCTTTGACCACCGGGCC
TCCAACCTGCTCTGGCCTCCCTGGATGGGCGTCCCCCACGGCTACGAGATCGAGTTTGTCTTTGGWATGC
CCCTCAACGACAGCCTGAACTACACGCCCMAMGAGAAGGAGCTGAGCCGCAGGATGATGCGCTACTGGGT
CGAACTTTGCCCGGACAGGGAACCCCGACCGACCCTGCCGACTAAGAACGGGGCCTGGCCCGACCTACAC
CGCCTCCCGGCCGCAGTACGTCCAGCTCAACACCCAGCCGCTGGCCACCCAACCCAG

>GAGZ01000015 Acanthophis wellsi CRiSP-Aca-1 mRNA sequence
ATGTTACAAATGGAATGGAATTCTCGTGCTGCTCAAAATGCAAAACGTTGGGCAGATAGATGCTTTTTTG
CTCACAGTCCACCACATACAAGAACTGTGGGAAAACTCCGTTGTGGTGAAAATATATTCATGTCAAGTCA
ACCTTTTCCATGGAGTGGAGTAGTTCAGGCTTGGTATGATGAAGTCAAAAAATTCGTCTATGGCATTGGA
GCAAAGCCACCAGGTTCTGTTATTGGCCATTATACCCAGGTAGTTTGGTACAAAAGTCACCTTCTTGGTT
GTGCTTCTGCCAAATGTTCTTCAACCAAATACCTCTACGTTTGTCAATACTGCCCAGCAGGGAACATCAG
AGGTTCAATTGCTACTCCATATAAATCAGGGCCATCTTGTGGGGACTGTCCTTCAGCTTGTGTCAACAGA
CTATGCACAAATCCTTGCAAATATAACAATGACTTCTCGAACTGCAAAGCTTTAGCGAAAAAAACTAAAT
GCAAGACTGGATGGATCAAGTCAAAATGCCCTGCTACTTGCTTCTGCCACACTGAAATAATATAG

>GAGZ01000016 Acanthophis wellsi KP-Aca-6 mRNA sequence
ATGTCTTCTGGAGGTCTTCTTCTCCTGCTGGGACTCCTCACCCTCTGGGAGGGGCTGACCCCCGTCTCCA
GCAAGGACCGTCCACACTTCTGTCATCTGCCTCATGACACCGGACCATGTAAAGGCATGTTCCCAGCCTT
CTACTACCACCCAGGTCATCGTACATGCCTACAGTTTATTTATGGTGGATGCCGAGGGAATCCTAACAAT
TTTAAGACCATAGATGAATGCAAACGCATCTGTGCTGCATGA

>GAGZ01000017 Acanthophis wellsi KP-Aca-8 mRNA sequence
ATGTCTTCTGGAGGTCTTCTTCTCCTGCTGGGACTCCTCACCCTCTGGGAGGGGCTGACCCCCGTCTCCA
GCAAGGACCGTCCACACTTCTGTCATCTGCCTCATGACACCGGACCATGTAAAGGCATGTTCCCAGCCTT
CTACTACCACCCAGGTCATCGTACATGCCTACAGTTTATTTATGGTGGATGCCGAGGGAATGCTAACAAT
TTTAAGACCATAGATGAATGCAAACGCATCTGTGCTGCATGA

>GAGZ01000018 Acanthophis wellsi KP-Aca-9 mRNA sequence
ATGTCTTCTGGAGGTCTTCTTCTCCTGCTGGGACTCCTCACCCTCTGGGAGGGGCTGACCCCCGTCTCCA
GCAAGGACCGTCCACACTTCTGTCATCTGCCTCATGACACCGGACCATGTAAAGGCATGTTCCCAGCCTT
CTACTACCACCCAGGTCATCGTACATGCCTACAGTTTATTTATGGTGGATGCCGAGGGAATGCTAACAAT
TTTAAGACCATAGAGGAATGCAAACGCATCTGTGCTGCATGA

>GAGZ01000019 Acanthophis wellsi KP-Aca-1 mRNA sequence
ATGTCTTCTGGAGGTGTTCTTCTCCTGCTGGGACTCCTCACCCTCTGGGAGGGGCTGACCCCCGTCTCCA
GCAAGGACCGTCCACACTTCTGTCATCTGCCTGCTGACACCGGACCATGTAAAGCCAACTTCCTAGCCTT
CTACTACCACCCAGTTCATCGTAAATGCCTAGAGTTTACTTATGGTGGATGTGAAGGGAATGCTAACAAT
TTTAAGACCATAGATGAATGCAAACGCACCTGTGCTGTATGA

>GAGZ01000020 Acanthophis wellsi LAO-Aca-1 mRNA sequence
GCCCTCTGCTACCCCTCAAAGATTAAAAAATGGAGCCTGGATAAATATGCTATGGGTGCTATAACCTCTT
TCACTCCTTACCAGTTTCAAGATTTTATTGAAACAGTCGCTGCACCTGTAGGCAGAATCTACTTTGCAGG
GGAGTACACTGCTAGCCTTCATGGTTGGTTGGACAGCACAATTAAGTCAGGGCTGACAGCAGCAAGAGAT
GTGAATCGTGCTTCTCAGAAGCCATCAAGAATCCAGCTGATCAGTGACAATCAACTTCAAGAAGGAGATC
AACACAAGGAGAATTATAAACAGCCATTCTGGCAACTTCCGGGTGTTTCAGACTAA

>GAGZ01000021 Acanthophis wellsi LP-Aca-2 mRNA sequence
ATGGGGCGATTCCTCTTGGTGGGCCTGTGCTTGCTGGTTGTGACTTTATCCCTAAATGGAATTGGAGCTG
ATCATCATTGCCTCTCGGGCTGGTTCCCATTTAAGACATTTTGCTACAAGGTCATCAGAGAATGGAAGAC
CTGGGAAGATGCCGAGGCATCCTGCGTGCAACTTCAGAACAGCAGCCATCTGGCCTCCATCCACAGCCAA
GCAGAGTCCTTTCACATCCAAAAGGTGATCTTCAGGGAACCGATCGTCCTCACTAATGTCTGGATTGGAC
TGAATGATCCAGAGRAAAATCGCACCTGGAAGTGGTCTGATGGCTCCAATTTTAATTATACATCATGGGG
AATGTTGAAACCAGGCATTTTTGATCATGGGAAGTACTGCGTTCAGTTGACCAGAATCTTAGGGTTTTAT
AGATGGGAAGCTGCCAAGTGTGAGTCCAAGAATTTTTTCATCTGCAAGATG

>GAGZ01000022 Acanthophis wellsi NP-Aca-6 mRNA sequence
ATGGTCTGCCTCTCACGTCTGGCGGGCGGCGGGCTGCTGCTGCTGCTGGTGCTGGCCCTGCTGCCTCTCG
CCCTCGACGGGAAGCCGACGGCGCCGCCTCAGGCGCTGCGCACGGCTCCGGCGGGCGGCATGATGGCGTC
GCGGCGGGAGCTGACGGAGCAGCCCCAGCAGCCGCTGATAAAGGACTCCTCGGGTCCCGCGGCTGAGCGC
AGCGGCTCCAAGACGGCCACGAAAGGGAACGGCTGCTTCGGCGTCAGGCTCGACCGCATCGGCAGCGTGA
GCGGCATGGGCTGCGGAGGCGTCCCGAAGCCGACACCTGGCGGATCCTAA

>GAGZ01000023 Acanthophis wellsi NP-Aca-7 mRNA sequence
ATGGTCGGCCTCTCACGTCTGGCGGGCGGCGGGCTGCTGCTGCTGCTGGTGCTGGCCCTGCTGCCTCTCG
CCCTCGACGGGAAGCCGACGGCGCCGCCTCAGGCGCTGCGCACGGCTCCGGCGGGCGGCATGATGGCGTC
GCGGCGGGAGCTGACGGAGCAGCCCCAGCAGCCGCTGATAAAGGACTCCTCGGGTCCCGCGGCTGAGCGC
AGCGGCTCCAAGACGGCCACGAAAGGGAACGGCTGCTTCGGCGTCAGGCTCGACCGCATCGGCAGCGTGA
GCGGCATGGGCTGCGGAGGCGTCCCGAAGCCGACACCTGGCGGATCCTAA

>GAGZ01000024 Acanthophis wellsi PLA2-Aca-2 mRNA sequence
ATGTATCCTGCTCACCTTCTGGTCCTGTTGGCAGTTTGTGTCTCCCTCTTAGGAGCCGCCAGAATTCCTC
CTCTGCCTCTCAACCTCGCACAATTCGGCAAAATGATTGAATGTGCCAACAAGGGCAGTCGACCTTCTTT
GGATTATATGAACTACGGTTGCTACTGTGGCACAGAAGATCGCGGGACACCGGTGGATGAGTTGGATAGG
TGCTGCAAAATACATGACGACTGCTATGCTGAAGCCGAAAAACATGGATGCTTCCCCAAGATGACGTCGT

ATAGTTGGAAATGTGCCAACGATGTACCCGTCTGCAATTCAAAAACGCAGTGTCAACATTTTGTGTGTTA
TTGTGACCTCTGGGCAACCAAGTGCTTTTCCCAAGAAACTTACAACAAGAACAACTTCGGTATCGACACC
AAGACACGTTGCAAATGA

>GAGZ01000025 Acanthophis wellsi PLA2-Aca-8 mRNA sequence
ATGTATCCTGCTCACCTTCTGGTCCTGTTGGCAGTTTGTGTCTCCCTCTTAGGAGCCTCCGACATTCCTC
CGCAGCCTCTCAGCCTCCTTCATTTCAAGAACATGATTAAATGTGCCAACAAGGGCACTCGAAGTTGGTT
GCGTTATGTGAACTACGGTTGCTACTGCGGCTGGGGAGGTAGCGGGAAACCGGTGGATGAGTTGGACAGG
TGCTGCAAAGCACATGACGACTGCTATGCTGAAGCCAGAAGAAAACGATGCTTCCCCAAGTTGACGTTGT
ATAGTTGGAAATGTAGCAGCCATGTGCCCGTCTGCAATTCAAAAAGGAAGTGTAAAGGTTTTGTGTGTGA
TTGTGACGCCACAGCAGCCAAGTGCTTTGCCAAAGCCCCTTACAACAAGACCAACTTCCGTATCAACACC
AAGAAACGTTGCCAATGA

>GAGZ01000026 Acanthophis wellsi PLA2-Aca-9 mRNA sequence
ATGTATCCTGCTCACCTTCTGGTCCTGTTGGCAGTTTGTGTCTCCCTCTTAGGAGCCTCCGACATTCCTC
CGCAGCCTCTCAGCCTCCTTCATTTCAAGAACATGATTAAATGTGCCAACAAGGGCACTCGAAGTTGGTT
GCGTTATGTGAACTACGGTTGCTACTGCGGCTGGGGAGGTAGCGGGAAACCGGTGGATGAGTTGGACAGG
TGCTGCAAAGCACATGACGACTGCTATGCTGAAGCCAGAAGAAAACGATGCTTCCCCAAGTTGACGTTGT
ATAGTTGGAAATGTGCCAAGGATGTGCCCGTCTGCAATTCAAAAAGGAAGTGTAAAGGTTTTGTGTGTGA
TTGTGACGCCACAGCAGCCAAGTGCTTTGCCAAAGCCCCTTACAACAAGACCAACTTCCGTATCAACACC
AAGACACGTTGCCAATGA

>GAGZ01000027 Acanthophis wellsi PLA2-Aca-34 mRNA sequence
ATGCGGTTGCCCTCACGTGAAAACCTGTATCCTGCTCACCTTCTGGTCCTGTTGGCAGTTTGTGTCTCCC
TCTTAGGAGCCGCCAGCATTCCTGTGCCATCTCTCAACTTCGAACAATTCGGCAACATGATTCAATGTAC
CATCCCCTGCGAAGAATCTTGCTTGGGTTATATGGACTACGGTTGCTACTGCGGCCCAGGAGGTAGCGGG
ACACCGATGGATGAGTTGGATAGGTGCTGCCAGACTCATGACAACTGCTATGCTGAAGCCGGAAAACTTC
CTGCATGTAAGGCCATGCTGAGTGAGCTGTACAACGACACCTATTCATACAGCTGTATTGAACGCCAACT
CACCTGCAACGATAACAACGATGAGTGTAAAGCCTTTATCTGTAATTGTGACCGCACAGCAGCCAACTGC
TTCGCCGGAGCCCCTTATAACGACTTGAACTACAATATCGACACCACTGAACATTGCCAATGA

>GAGZ01000028 Acanthophis wellsi PLA2-Aca-38 mRNA sequence
ATGTATCCTGCTCACCTTCTGGTCCTGTTGGCAGTTTGTGTCTCCCTCTTAGGAGCCTCCGACATTCGTC
CGCAGCCTCTCAACCTCCTTCAGTTCGGCTTCATGATTCGATGTGCCAACAAACGCAGACGACCTGTTTG
GCATTATATGGACTACGGTTGCTACTGCGGCAAAGGAGGTAGCGGGACACCGGTGGATGCGTTGGATAGG
TGCTGCAAAATACATGACGACTGCTATGCTGAAGCCGAAAAACGCTACGGATGCTACCCCTACTGGACGT
TGTATAGTTGGAAATGTTACGGAAGTGGACCGTACTGCAATTCAAAAACGCAGTGTCAACGTTTTGTGTG
TGCTTGTGACTCCAGAGCAGCCATCTGTTTCGCTCAAAACCCTTACAACAACGCGAACTGGAATATCAAC
ACCAAGACACGTTGCCGATGA

>GAGZ01000029 Acanthophis wellsi PLA2-Aca-43 mRNA sequence
ATGCTTATATTGTGTTGCCTTAATCCTCTTAAAACTGAAATAAAGTTGCAAATATATTCACTTCCGATTC
ATCTTGCTTGCAGCTTCACCACTGACAAAATGTATCCTGCTCACCTTCTGGTCCTGTTGGCAGTTTGTGT
CTCCCTCTTAGGAGCCTCCGACATTCCTCCGCAGCCTCTCAACCTCTATCAGTTCGGGGGAATGATTAAA
TGTGCCAACAAGGGCACTCGAAGTTGGTTGAGTTATGTGAACTACGGTTGCTACTGCGGCTGGGGAGGTA
GCGGGAAACCGGTGGATGAGTTGGATAGGTGCTGCCAGATTCATGACAACTGCTATGCTGAAGCCGGAAA
AAAACGATGCAAACCCAAGTTGACGTTGTATAGTTGGAAATGTGCCAAGGATGTACCCGTCTGCAATTCA
AAATCGGTGTGTGGAAGTTTTGTGTGTGATTGTGACGCCGCAGCAGCCAAGTGCTTTGCCAAAGCCCCTT
ACAACAAGAACAACATCGGTATTGGTTCCAAGAAACGTTGCCAATGA

>GAGZ01000030 Acanthophis wellsi PLA2-Aca-45 mRNA sequence
ATGTATCCTGCTCACCTTCTGGTCCTGTTGGCAGTTTGTGTCTCCCTCTTAGGAGCCTCCGACATTCCTC
CGCAGCCTCTCAACCTCTATCAGTTCGGGGGAATGATTAAATGTGCCAACAAGGGCACTCGAAGTTGGTT
GAGTTATGTGAACTACGGTTGCTACTGCGGCTGGGGAGGTAGCGGGAAACCGGTGGATGAGTTGGATAGG
TGCTGCCAGATTCATGACAACTGCTATGCTGAAGCCGGAAAAAAACGATGCAAACCCAAGTTGACGTTGT
ATAGTTGGAAATGTGCCAAGGATGTGCCCGTCTGCAATTCAAAATCGGTGTGTGGAAGTTTTGTGTGTGA
TTGTGACGCCGCAGCAGCCAAGTGCTTTGCCAAAGCCCCTTACAACAAGAACAACATCGGTATTGGTTCC
AAGACACGTTGCCAATGA

>GAGZ01000031 Acanthophis wellsi PLA2-Aca-97 mRNA sequence
ATGATTCGATGTGCCAACAAACGCAGACGACCTGTTTGGCATTATATGGACTACGGTTGCTACTGCGGCA
AAGGAGGTAGCGGGACACCGGTGGATGCGTTGGATAGGTGCTGCAAAATACATGACGACTGCTATGCTGA
AGCCGAAAAACGCTACGGATGCTACCCCTACTGGACGTTGTATAGTTGGAAATGTTACGGAAGTGGACCG
TACTGCAATTCAAAAACGCAGTGTCAACGTTTTGTGTGTGATTGTGACGCCGCAGCAGCCAAGTGCTTTG
CCAAAGCCCCTTACAACAAGAACAACATCGGTATTGGTTCCAAGAAACGTTGCCAATGA

>GAGZ01000032 Acanthophis wellsi SVMP-Aca-4 mRNA sequence
ATGATCCAAGCTCTCTTGGTAACTATATGCTTCATGGTTTTTCCATATCAAGGGAGCTCTATCATCCTGG
AATCCGGGAATGTTAATGATTATGAAGTAGTGTATCCACAAAAAGTGCCTGTATTGCCCAAAGGAGGAGT
TCAGAATCCTCAGCCAGAGACCAAGTATGAAGATACAATGCAATATGAATTTCACATGAATGGAGAGCCA
GTGGTCCTTCACTTAGAAAGAAATAAAGGACTTTTTTCAGAAGATTACACTGAAACTCATTATGCCCCTG
ATGGCAGAGAAATTACAACGAGCCCTCCGGTTCAGGATCACTGCTATTATCATGGCTACATTCAGAATGA
AGCTGATTCAAGTGCAGTCATCAGTGCATGCGACGGCTTGAAAGGACATTTCAAGCATCGAGGGGAGACA
TACTTTATTGAGCCCTTGAAGCTTTCCGACAGTGAATCCCATGCAATCTACAAAGATGAAAATGTAGAAA
AAGAGGATGAGATCCCCAAAATCTGTGGAGTAACCCAGACTACTTCGGAGTCAGATGAGCCCATCGAAAA
GATCTCTCAATTAACTAATACTCCTGAACAAGACAGGTACTTGAAGGTCAAAAAATACATTGAGTTTTAC
GTGGTTGTGGACAACAAAATGTACAAGAATTACAGCAGCAACAGAGATGCTATAAAAAGAAGAGTATATG
AAATGGTCAACACTTTAAATGTGATGTACAGACCTTTGAATATTTTCATTGCACTGATTGGCCTACAAAT
TTGGACCAACAAAGATAAGATTAACATTGAACCAGAGGTGGCTGTCACTTTGACATCATTTGGAAAATGG
AGAGAAACAGTTTTACTGCCACGCAGAAGGAATGATAATGCTCAGTTACTTACGGGCATTGACTTCAACG
GAGCTACTGTAGGACGTGCTTACGTTGGCACCCTCTGCAGTCCAGAGAAATCTGTAGCAGTTATTCAGGA
TTATAGCAAAAGAACAAGCATGGTGGCATCTACAATGGCCCATGAGTTGGGTCATAATCTGGGCATTGAT
CATGACAACGCTTCCTGTAATTGCAGTGCTGGACCATGCATTATGTCTGCAAAGATAAGTAATGAACCTC
TCTCTGAGTTCAGCAATTGTAGTATCCAGAAATATCAGAGGTATCTTCTTAGAGTCAGACCACAATGCAT
TCTCAACAAACCCTTGAGCACAGATATTGTTACACCTCCAGTTTGTGGAAATTACTTTGTGGAGCGGGGA
GAAGAATGTGACTGTGGCTCTCCTGTGGATTGTCAAAGTGCCTGCTGCAACGCCACAACTTGTAAACTGC
AACACGAGGCACAGTGTGACTCTGAAGAGTGTTGTGAGAAATGCAAATTTAAGAAAGCAGGAACAGAATG
CCGGGCAGCAAAGCATGACTGTGACTTGTCTGAAATCTGTACTGGCCAATCTGCTGAGTGTCCCACGGAC
AGCTTCCAGAGGAATGGACATCCATGCCAAAACAACCAGGGTTACTGCTACAATGGGAAATGCCCCCTCA
TGACAAACCAATGTATTGATCTCTGGGGTCCAGGTGTAAATGTGTCTCCAGATATATGTTTTACGTTGAA
CCAGGATGGGAAAGATTGTAGCTTCTGCAGAATGGAAAATGGTAGAAGGATTCCATGTGCAGCAAAGGAT

GAAAAGTGTGGCACGTTATACTGCAAAAGGGGAAACCGTCGATGCATCTGCGTTGATGGCCCAGATTATA
GAATGGTTGAACCTGGAACAAAATGTGGAGATGGAAAGGTGTGCATCAACAGGCAGTGTGTTGATGTGCA
GACAGCCTACTGA

>GAHA00000000.1|GAHA01000000 TSA: Brachyurophis [Simoselaps] roperi, transcriptome shotgun assembly, transcriptome shotgun assembly

>GAHA01000001 Brachyurophis [Simoselaps] roperi 3FTx-Bra-3 mRNA sequence
ATGAAAACTCTGCTGCTGACCTTGGTGGTGGTGACAATCGTGTGCCTGGACTTAGGTAATAGCTTCTCAT
GCTACAAAACACCTGATCTAAAATCTGAGCCTTGTGAACCTGGGGAGAACCAATGCTATACAAGGACTTG
GTGTGATGCTCGGTGTGGCCTCATGGGAAAGCGAATCGAACTGGGATGTGCTGCTACTTGCCCTACAGCG
AAGACCGGCATCGATATTAAGTGTTGCTCAACAGACAATTGCAACCCACATCCGAAACAGAAATCTCGTT
GA

>GAHA01000002 Brachyurophis [Simoselaps] roperi 3FTx-Bra-6 mRNA sequence
ATGAAAACTCTGCTGCTGACCTTGGTGGTGGTGACAATCGTGTGCCTGGACTTAGGTGACAGCTTGATAT
GCTTCGAGGGATATAAGGCCCCTAAGACTTGTCCACCTGGGCAGAACCTATGCTATACAAAGACTTTGTG
TGGTTTTCGTTGTGCCAGCAGAGGACCGCGAGTCGAACTGGGATGTGCTGCTACTTGCCCTATTCCAAAG
CCCCGTGAGGAGGTTACCTGTTGCTCAACAGACAATTGCAACCCATTTCCGAAATGGAATCCTTGGGGGT
TGCTCTCATCCATCATGGACCATCCTTGA

>GAHA01000003 Brachyurophis [Simoselaps] roperi 3FTx-Bra-8 mRNA sequence
ATGAAAACTCTGCTGCTGACCTTGGTGGTGGTGACAATCGTGTGCCTGGACTTAGGTGACAGCTTGATAT
GCAACGTGGATTATAAAAAGCATGAGACTTGTCCACCTGAGCGTAACCTATGCTATAAAATTACTTCGTT
TCTTGGTTGGTGTCCCAAGGGAGAACAGCTAATCAAAATGGGATGTGCTGCTACTTGCCCTAGACGGCTC
GGTGTGAAGATTACCTGTTGCTCAACAGACAATTGCAACCAACATAGGCCACGTTGTTGA

>GAHA01000004 Brachyurophis [Simoselaps] roperi 3FTx-Bra-9 mRNA sequence
ATGAAAACTCTGCTGCTGACCTTGGTGGTGGTGACAATCGTGTGCCTGGACTTAGGTGACAGCTTGATAT
GCAACGTGGATTATAAAAAGCATGAGACTTGTCCACCTGGGCGTATCCTATGCTATAAAATTACTTCGTT
TCTTGGTTGGTGTCCCGAGAGAGAAAGGCTAATCAAAATGGGATGTACTGCTACTTGCCCTAGACGGCCC
GGTGTGAAGGTTACCTGTTGCTCAACAGACAATTGCAACCAACATAGGCCACGTTGTTGA

>GAHA01000005 Brachyurophis [Simoselaps] roperi 3FTx-Bra-10 mRNA sequence
ATGAAAACTCTGCTGCTGACCTTGGTGGTGGTGACAATCGTGTGCCTGGACTTAGGTGACAGCTTGATGT
GCAACGTGGATTATAAAAAGCATGAGACTTGTCCACCTGAGCATATCCTATGCTATAAAATTACTTCGTT
TATTGGTTGGTGTCCCGAGGGAGAACAGCTAATCAAAATGGGATGTGCTGCTACTTGCCCTAGACGGCTC
GGTGTGAAGGTTACCTGTTGCTCAACAGACAATTGCAACCAACATAGGCCACGTTGTTGA

>GAHA01000006 Brachyurophis [Simoselaps] roperi 3FTx-Bra-12 mRNA sequence
ATGAAAACTCTGCTGCTGACCTTGGTGGTGGTGACAATCGTGTGCCTGGACTTAGGTGACAGCTTGATAT
GCAACGTGGATTATAAAAAAAATGAGACTTGTCCACCTGGGCATAACCTATGCTATAAAATTACTTCGTG
TGTTGGTTGGTGTCCCAAGGGAGAAAAGCTAATCAAAATGGGATGTGCTGCTACTTGCCCTAGACGGCTT
GGTGTGAAGGTTACCTGTTGCTCAACAGACAATTGCAACCAACGTAGGCCACCTCGTTGA

>GAHA01000007 Brachyurophis [Simoselaps] roperi 3FTx-Bra-13 mRNA sequence
ATGAAAACTCTGCTGCTGACCTTGGTGGTGGTGACAATCGTGTGCCTGGACTTAGGTGACAGCTTGATAT
GCAACGTGGATTATAAAAATGAGACTTGTCCACCTGGGCATAACCTATGCTATAAAATTACTTCGTGTGT
TGGTTGGTGTCCCAAGGGAGAAAAGCTAATCAAAATGGGATGTGCTGCTACTTGCCCTAGACGGCTTGGT
GTGAAGGTTACCTGTTGCTCAACAGACAATTGCAACCAACGTAGGCCACCTCGTTGA

>GAHA01000008 Brachyurophis [Simoselaps] roperi 3FTx-Bra-17 mRNA sequence
ATGAAAACTCTGCTGCTGACCTTGGTGGTGGTGACAATCGTGTGCCTGGACTTAGGATACACCATGACAT
GCTACAAAACAGCTCCTGTTAGATCTGAGCCTTGTCCACCTGGGGAGGACCTATGCTATACAAAGGCTTG
GTGTGATTTTCGGTGTGGCACCCATGGAAAGATACTCGAACTGGGATGTGCTGCTACTTGYCCTATACCG
AAGCACTATGAGGATGTTAAGTGTTGCTCAACAGACAATTGCAACCCATTTCCGAAACCGACAGGTGTTA
GACCAGGTCGACCAGGTGGTTGA

>GAHA01000009 Brachyurophis [Simoselaps] roperi 3FTx-Bra-25 mRNA sequence
ATGAAAACTCTGCTGCTGACCTTGGCGGTGGTGACAATCGTGTGCCTGGACTTAGGATACACCCTAAAAT
GTCGCAGTTCTAGGATTGCTAATCGTAATGTGGTTTGTAAAAAATACCAGACCATTTGCTTTCACTATCA
AGTTCGTCCATATCAAAATACTGTGTTAACCAGGATGGGATGTAGTGATTCTTGCCCTAGAAAGGATGCT
GTGTGTTGCAGTTCAGACTTGTGCAACTAG

>GAHA01000010 Brachyurophis [Simoselaps] roperi 3FTx-Bra-26 mRNA sequence
ATGAAAACTCTGCTGCTGACCTTGGTGGTGGTGACAATCGTGTGCCTGGACTTAGGATACACCCTAAAAT
GTCGCAGTTCTAGGATTGCTAATGGTAATGTGGTTTGTGAAAAATACCAGACCATTTGCTTTCGATATCA
AGTTCGTCCATATCAAAATATTGTGTTAACCAGGATGGGATGTAGTGATTCTTGCCCTAGAAAGGATGCT
GTGTGTTGCAGTTCAGACTTGTGCAACTAG

>GAHA01000011 Brachyurophis [Simoselaps] roperi 3FTx-Bra-27 mRNA sequence
ATGAAAACTCTGCTGCTGACCTTGGTGGTGGTGACAATCGTGTGCCTGGACTTAGGATACACCCTAAAAT
GTCGCAGTTCTAGGATTGCTAATCGTAATGTGGTTTGTAAAAAATACCAGACCATTTGCTTTCACTATCA
AGTTCGTCCATATCAAAATACTGTGTTAACCAGGATGGGATGTAGTGATTCTTGCCCTAGAAAGGATGCT
GTGTGTTGCAGTTCAGACTTGTGCAACTAG

>GAHA01000012 Brachyurophis [Simoselaps] roperi 3FTx-Bra-28 mRNA sequence
ATGAAAACTCTGCTGCTGACCTTGGTGGTGGTGACAATCGTGTGCCTGGACTTAGGATACACCAGGATAT
GTTTCAACCAACAGTCATGGCAACCTGAAACCACTACAACTTGTCCAGATGGGGAGACCTCTTGCTATAA
AAAGACTTTGATCAGTTTGTTTAGAATTCTAATTGAAAAGGGATGTGGTTGCCCTAAGGAGAGGAGCAAT
TTTAGACTTATGTGTTGCCAAAAAGACAAATGCAACAATTAG

>GAHA01000013 Brachyurophis [Simoselaps] roperi 3FTx-Bra-30 mRNA sequence
ATGAAAACTCTGCTGCTGACCTTGGTGGTGGTGACAATCGTGTGCCTGAACTTAGGATACACCAGGATAT
GTTACAACCAAAAGGCATCGCGACCTAAAACCACTATAACTTGTCCAGATGGGGAGACCTCTTGCTATAA
AAAGGATTTGATGAGTTCCTTTGGAATTCTAATTCTAAGGGGATGCGGTTGCCCTAAGGAGGTCCCCAAT
CTTTTACTTATGTGTTGCAAAAAAGACAAATGCAACAATTAG

>GAHA01000014 Brachyurophis [Simoselaps] roperi 3FTx-Bra-34 mRNA sequence
ATGAAAACTCTGCTGCTGACCTTGGTGGTGGTGACAATCGTGTGCCTGAACTTAGGATACACCAGGATAT
GTTTCAACCAACAGTCATGGCAACCTGAAACCACTACAACTTGTCCAGATGGGGAGACCTCTTGCTATAA

AAAGACTTTGATCAGTTTGTTTAGAATTCTAATTGAAAAGGGATGTGGTTGCCCTAAGGAGAGGAGCAAT
TTTATGTGTTGCCAAAAAGACAAATGCAACAATTAG

>GAHA01000015 Brachyurophis [Simoselaps] roperi 3FTx-Bra-36 mRNA sequence
ATGAAAACTCTGCTGCTGACCTTGGTGGTGGTGACAATCGTGTGCCTGGACTTAGGATACACCAGGATAT
GTTTCAACCAACAGTTATGGCAACCTGAAACCACTACAACTTGTCCAGATGGGGAGACCTCTTGCTATAA
AAAGAATTTGATCAATTTGTTTAGAATTCCAATTGAAAGGGGATGTGGTTGCCCTAAGGAGAGGGGCTAT
TTTAGACTTATGTGTTGCAAAAAAGACAAATGCAACAATTAG

>GAHA01000016 Brachyurophis [Simoselaps] roperi 3FTx-Bra-38 mRNA sequence
ATGAAAACTCTGCTGCTGACCTTGGTGGTGGTGACAATCGTGTGCCTGGACTTAGGATACACCAGGATAT
GTTTCAACCAACAGTCATGGCGACCTGAAACCACTACAACTTGTCCAGATGGGGAGAACACTTGCTATAA
AAAGGTTTTCATGACTCCCTTTAGAATTCCAATTGAAAGGGGATGTGGTTGCCCTAAGGAGAGGCGCAGT
TTTAGACTTATGTGTTGCCAAAAAGACAAATGCAACAATTAG

>GAHA01000017 Brachyurophis [Simoselaps] roperi CRiSP-Bra-2 mRNA sequence
ATGGGTTTAASCARGGGCTCATTCAAAACAATAGAAATGATTGCCTTCATTGTCTTGCTAAGTCTTGCTG
CAGTGCTGCAACAGTCTTCTGGAACTGTTGATTTTGCTTCTGAGTCAAGTAACAAAACAGGATACCAAAA
GCAAATTGTTGACAAGCACAATGCTTTAAGGAGATCAGTGAAACCAACTGCTAGGAACATGTTACAAATG
GAATGGAATTCTCGTGCTGCTCAAAATGCAAAACGTTGGGCAGATAAATGTTCTTTTGCTCACAGTCCAC
CACATAGAAGAACTGTGGGAAAACTCCGTTGTGGTGAAAATATATTCATGTCAAGTCAACCTTTTGCATG
GAGTAGAGTAATTCAGGCTTGGTATGATGAAAACAAAAAATTCGTCTATGGCACTGGAGCAAAGCCACCA
GGTTCGGTTATTGGCCATTATACCCAGATAGTTTGGTACAAAAGTCACCTTCTTGGTTGTGCTTCTGCCA
GATGTTCTTCATCCAAATACCTCTACGTTTGTCAATACTGCCCAGCAGGGAACATCAGAGGTTCAATTGC
TACTCCATATAAATCAGGGCCACCTTGTGGGGACTGTCCTTCGGCTTGTGTCAACGGACTATGCACAAAT
CCTTGCAAATATGAAAATGACTTCTCGAACTGCAAATCTTTAGCGAGAAAAACTAAATGCCAGACTGAAT
GGACCAAGTCAAAATGCCCTGCTTCTTGCTTCTGCCGCACTGAAATAATATAG

>GAHA01000018 Brachyurophis [Simoselaps] roperi LP-Bra-2 mRNA sequence
ATGGGGCGATTCCTCTTTGTGAGCCTCGGCTTGCTGGTCGTGGCTTTCTCCCTAAGTGGAATTGGAGCCA
ATCGCTATTGTCCCTTTGATTGGTTCTCCTACAATGTGTCTTGCTACAAGCTCTTCCACCTTCCGATGCC
CTGGGACCAAGCACAGAGGTCCTGTGTGGAACAGCAGGAAAACTCCCAACTCGCCTCCATCCATGACGTG
GGAGAGTCAGTGAAGTTGTCCAACTACATATCCCAAAGGTTGAAATTATCTGATGTTTGGATGGGACTGA
GGCTTTCAAAGAGAAAAGGCATCTGGGAGTGGAGTGATGGCTCCAACCTCACTTACACATCCTGGAAAGA
AGGTGAACCCAACAACTTTTTCAACATTGAATTCTGTGCTGTGCTGTCAGCCGGAACACGGTATCTCCAA
TGGAACGATAAGAGCTGCGTGCTCCCACATAAATTTATCTGCAAGTACCAGCCCCGGAGTGAGGCAGGCA
GCTAA

>GAHA01000019 Brachyurophis [Simoselaps] roperi LP-Bra-3 mRNA sequence
ATGGCGCAATTCATCGTTGTGAGCTTCAGCTTGCTGGTCGTGGCCCTCTCCTTAAGGGGAACTGGAGCTG
ATCACCATTGTCCTCGCGGTTGGTCCTCCTATAATGAATTTTGCTACATGGTCTACAACAGATCCGTGAC
CTGGAACAATGCAGAGAGATTCTGCCTGCAGCAAGAGCCAGACTGCCACCTCGCCTCCATCCATAGCAAG
CCAGAGGCAGCCTTCCTAGTCGGGCTGGCCTACCACAGGGCCAGCGCCGGGTCCAGTGTCTGGATCGGAT
TGAACGATCCAGAGAAAAGACGCGCTTGGCAATGGAGTGATGGCTCCAGCATAAACTACAGGTCCTGGAT
GCCAGGAGAGCCCAACAACCATGCAAACAAGGAGTATTGTGTTGTGCTGTCAGCCTGGTCACGTTATGTC
GGATGGAATGATCAGGACTGTGGGTCCAGGCATAATTTTGTCTGCAAGTTCCAGCCAAGGACCAAGAGGA
ACACCGTCGATGTTACAAACAGGAAGTAA

>GAHA01000020 Brachyurophis [Simoselaps] roperi LP-Bra-8 mRNA sequence
ATGGGGCGATTCCTCTTGGTGAGCCTGAGCTTGCTGGTTGTGGCTTTGTCCCTAAATGGAATTGGAGCTG
ATCATCATTGCCCCTCGGACTGGATCTCCCTGGGGAGGTATTGCTACAAGTTCATCAAAGAACGGAAGAC
CTGGGAAGATGCCGAGGAATCCTGCATGCAACTTCAGAACAGCAGCCATCTGGCCTCCATCCACAGCACA
GTAGAGTCCTTTTACACCCACAGGGTGATCTCCAGCAGAGGAGTCTTCTTCAATGATGTCTGGATTGGAC
TGAATGATCCAGGGAAAAATCGTATCTGGGAGTGGACTGATGGCTCCGATTTTGATTATACATCCTGGAG
AGTACGGGAACCCGGCAATGCTGATAGTGGGGAGTACTGTGTTAAGTTGACCAGATTCTCAAGGTTTTTT
AGTTGGAAAGCTGCCAACTGTGAATCCGAGAATTTTTTCATCTGCAAGATGTAG

>GAHA01000021 Brachyurophis [Simoselaps] roperi LP-Bra-11 mRNA sequence
ATGGGGCGATTCCTCTTGGTGAGCCTGAGCTTGCTGGTTGTGGCTTTGTCCCTAAATGGAATTGGAGCTG
ATCATCATTGCCCCTCGGACTGGATCTCCCTGGGGAAGTATTGCTACAAGTTCATCGAAGAACGGAAGAC
CTGGGAAGATGCCGAGGAATCCTGCGTGCAACTTCAGAACAGCAGCCATCTGGCCTCCATCCACAGCACA
GCAGAGTCCCTTGGCCTCAACACAATGATCTTCAAGAAAAGGCTCATAAGCACTAAAGTCTGGATTGGAC
TGAATGATCCAGGGAAAAATCGTATCTGGGAGTGGACTGATGGCTCCAATTTTGATTATACATCCTGGGG
AATATTTGAACCCGGCAATGCTGATCGTGGGGAGTACTGCGTTCAGTTTACCAGTTCGTTATGGTTTTCT
GGTTGGAAAGCTGACAACTGTGAATCCGAGAATTTTTTCGTCTGCAAGATGTAG

>GAHA01000022 Brachyurophis [Simoselaps] roperi LP-Bra-16 mRNA sequence
ATGAAGCGATTCATTCTTCTAAGTCTTGGCTTGCTGGTTGTGGCCCTCTCCTTAAGGGGAACTGGAGCCA
GTCCCCAATGTCCCTCCGAGTGGTCCTTCTTTAATGGGCATTGCTACAAGTTCTTCAAAACTCTCAAGAG
CTGGCGTGAGGCAGAGATGTCCTGCAGGCAGCAGGAGGAAGGCAGCCACCTCACCTCCATCCAAAGCTGG
GCAGAGTCAGATTATGTGGCCAAGTTGATCCCCCGCGATGTCTTCTTCGTCAATGTCTGGATCGGACTGA
GTGATCCAGAGAAACGACGCATCTGGAAGTGGAGTGATGGCTCCAAGTTCCTCTACAGATCCTGGAAACG
AGGAGAACCCAACAACTTTTTCTGGAATGAGTACTGCGTCGAACTGTGGAACTGGCCAGGGTACCTCAGG
TGGAATGATCAGAATTGCAGGTTCAAACGTTATTTCATCTGCAAGTTCCAGCCCCAGGGTGAGGGAAGCA
CCTGGTGA

>GAHA01000023 Brachyurophis [Simoselaps] roperi NP-Bra-2 mRNA sequence
ATGCTCGGCCTCGCCCGTCTGGCTGGCGGCGGGCTGCTGCTGCTGCTACTGCTGGCCCTGCTGCCTCTCG
CCCTCGACGGGAAGCCGGCGCCGCTGCCTCAGGCGCTGCCCAAGGCTCCGGCGGGCGGCACGACGGCGTC
GCGGCGGGAGCTGACGGAGCAGCAGCAGCCGCCGTCACCGGCGGAGGAGTCCTCGGGTCCCGCGGCTGGG
CGCAGCGGCTCCAAGGCGGCCAAGATAGAGCTGGACAACGGCTGCTTCGGCGTCCGGCTCGACCGCATCG
GCACCTCGAGCGGCCTGGGCTGCAAAAACCCCGTCGAAAACGCCCCGACGCCGACACCTGCCGGATCCTA
A

>GAHA01000024 Brachyurophis [Simoselaps] roperi NGF-Bra-3 mRNA sequence
ATGCATAGCGTAACATCCATGTTGTGCTACACTCTGATTATAGCATTTCTGATTGGCATATGGGCAGCAC
CAAAATCTGAAGATAATGTCCCTCTGGGCTCCCCTGCAACATCTGACCTTTCTGACACCAGCTGTGCTCA
AACTCACAAGGGTCTGAAAACATCTCTAAATACAGATCAGCACCATCCTGCTCCTAAGAAGGCAGAGGAT
CAAGAACTTGGATCAGTAGCAAACATCATTGTGGATCCCAAGCTTTTTCAGAAGAGGCGTTTCCAGTCAC
CTCGTGTTCTGTTCAGCACTCAGCCCCCACCGTTGTCAAGAGATGAGCAAAGTGTGGAGTTCCCCGACAA
TGAAGACGCTCTTAACATGAGTATCCGGGCCAAACGTGAAAATCATCCTGCGTATAATGTAGGGGAACAT

TCTTTGTGTGACAGTATCAATAACTGGGTTACCAAAACCACAGCAACGGACATCCACGACAATAGGGTGA
CTGTGACGGCGGAGATAAATATTAATAACCAATTCTTCAAGCAGTACTTTTTTGAAACCAAGTGCAGAAA
TCCAAATCCATTACCGAGTGGGTGCAGAGGCATTGATTCCAGGTATTGGAATTCTTATTGCACCACAACA
CAGTCATTTGTCATGGCATTAACAACGGAAGACAATCGGGCATCCTGGCGCTTCATTCGGATTGACACTG
CCTGTGTGTGCGTAATCAGTAGAAAATCTGACAGCTTCTGA

>GAHA01000025 Brachyurophis [Simoselaps] roperi NGF-Bra-4 mRNA sequence
ATGCATAGCGTAACGTCCATGTTGTGCTACACTCTGATTATAGCATTTCTGATTGGCATATGGGCAGCAC
CAAAATCTGAAGATAATGTCCCTCTGGGCTCCCCTGCAACATCTGACCTTTCTGACACCAGCTGTGCTCA
AACTCACAAGGGTCTGAAAACATCTCTAAATACAGATCAGCGCCATCCTGCTCCTAAGAAGGCAGAGGAT
CAAGAACTTGGATCAGCAGGCAACATCATTGTGGATCCCAAGCTTTTTCAGAAGAGGCGTTTCCAGTCAC
CTCGTGTTCTGTTCAGCACTCAGCCCCCACCGTTGTCAAGAGATGAGCAAAGTGTGGAGTTCCCCGACAA
TGAAGACGCTCTTAACATGAATATCCGGGCCAAACGTGAAAATCATCCTGCGTATAATGTAGGGGAACAT
TCTGTGTGTGACAGTATCAATAGCTGGGTTACCAAAACCACAGCAACGGACATCCACGACAATAGGGTGA
CTGTGACGGCGGAGATAAATCTTAATAACCATGTCTACAATCAGTACTTTTTTGAAACCAAGTGCATAAA
TCCAAATCCAGTACCGAGTGGGTGCAGGGGCATTGATTCCAGGTATTGGAATTCTTATTGCACCACAACA
CAGTCATTTGTCATGGCATTAACCATGGAAGGCAATCGGACATCCTGGCGCTTCATTCGGATTGACACTG
CCTGTGTGTGCGTAATCAGTAGAAAATCTGACAGCTTCTGA

>GAHA01000026 Brachyurophis [Simoselaps] roperi NGF-Bra-7 mRNA sequence
ATGCATAGCGTAACGTCCATGTTGTGCTACACTCTGATTATAGCATTTCTGATTGGTATATGGGCAGCAC
CAAAATCTGAAGATAATGTCCCTCTGGGCTTCCCTGCAACATCTGACCTTTATGACACCAGCTGTGCTCA
AACTCACAAGGGTCTGAAAACATCTCTAAATACAGATCAGCGCCATCCTGCTCCTAAGAAGGCAGAGGAT
CAAGAACTTGGATCAGCAGCCAACATCATTGTGGATCCCAAGCTTTTTCAGAAGAGGCGTTTCCAGTCAC
CTCGTGTTCTGTTCTGCACTCAGCCCCCACCGTTGTCAAGAGATGAGCAAAGTATGGAGCTCCCCGACAA
TGAAGATGCTCTTAACATGAGTATCTGGGCCCAACGTGAAAATCATCCTGCGTATAATGTAGGGGAACAT
TCTGTGTGTGACAGTATCAATAGCTGGGTTACCAAAACCACAGCAACGGACATCTACGACAATAGGGTGA
TTGTGACGGCGGGGATAAATCTTAATAACCATGTCTACAAGCAGTACTTTTTTGAAACCAAGTGCAGAAA
TCCAAATCCAGTACCGAGTGGGTGCAGGGGCATTGATTCCAGGTATTGGAATTCTTATTGCACCACAACA
CAGTCATTTGTCAAGGCATTAACCATGGAAGGCAATCAGGCATCCTGGCGCTTCATTCGGATTGACACTG
CCTGTGTGTGCGTAATCAGTAGAAAATCTGACAGCTTCTGA

>GAHA01000027 Brachyurophis [Simoselaps] roperi NGF-Bra-9 mRNA sequence
ATGCATAGCGTAACGTCCATGTTGTGCTACACTCTGATTATAGCATTTCTGATTGGCATATGGGCAGCAC
CAAAATCTGAAGATAATGTCCCTCTGGACTCCCCTGCAACATCTGACCTTTCTGACACCAGCTGTGCTCA
AACTCACGAGGGTCTGAAAACATCTCTAACTACAGATCAGCGCCATCCTGCTCCTAAGAAGGCAGAAGAT
CAAGAACTTGGATCAGCAGCCAACATCATTGTGGATCCCAAGCTTTTTCAGAAGAGGCGTTTCCAGTCAC
CTCGTGTTCTATTCAGCACTCAGCCCCCACCATTGTCAAGAGATGAGCAAAGTGTGGAGTTCCTGGACAA
TGAAGACACTCTTAACAGGAATATCCGGACCAAACGTGAAACTCATCCTGTGCATAACCGAGGGGAATAT
TCTGTGTGTGACAGTATCGATAGCTGGGTTACCAAAACCACAGCAACGGACATCCACGACAATAGGGTGA
CTGTGATGGTGGATGTAAATCTTAATAACCATTTCTACAAGCAGTACTTTTTTGAAACCAAGTGCACAAA
TCCAAATCCATTACCGAGTGGGTGCAGGGGCATTGATTCCAGGTATTGGAATTCTTATTGCACCACAACA
CAGTCATTTGTCATGGCATTAACCATGGAAGGCAATCGGACATCCTGGCGCTTCATTCGGATTGACACTG
CCTGTGTGTGCGTAATCAGTAGAAAATCTGACAGCTTCTGA

>GAHA01000028 Brachyurophis [Simoselaps] roperi PLA2-Bra-11 mRNA sequence
ATGTATCCTGCTCACCTTCTGGTCTTGTTGGCAGTTTGTGTCTCCCTCTTAGGAGCCTCCGACGTTCCTC
CGATGCCTCTCAACCTCCTACAATTCGGCAACATGATTAACTGTGCCAACCGTGGCCGTCGACCTGCTAG
GCATTATAGGGACTACGGTTGCTACTGCGGCAAAGGAGGTAGTGGGACACCGGTAGATGACTTGGATAGG
TGCTGCAAAACACATGACGACTGCTATGGTGAAGCCGAAAAATATCCTTCATGTAAGCCCTACTACAACT
CCTATTCATACAAATGTAATGGAGGCGAAGTCACCTGCACAGATGACAACGATGAGTGTAGAGCCTTTAT
TTGTAATTGTGACCGCACGGCAGCCATCTGCTTCGCCAGAGCCTATTACAACGAGGAGAACTGGGATATC
GACACCGCTGCACGTTGCCAATGA

>GAHA01000029 Brachyurophis [Simoselaps] roperi PLA2-Bra-12 mRNA sequence
ATGTATCCTGCTCACCTTCTGGTCTTGTTGGCAGTTTGTGTCTCCCTCTTAGGAGCCTCCGACGTTCCTC
CGATGCCTCTCAACCTCCTACAATTCGGCAACATGATTAACTGTGCCAACCGTGGCCGTCGACCTGCTAG
GCATTATAGGGACTACGGTTGCTACTGCGGCAAAGGAGGTAGTGGGACACCGGTAGATGACTTGGATAGG
TGCTGCCAGTCACATGACTACTGCTATGATGAAGCCGAAATACTTCCTACATGTAATTACATGCTGAGTG
GCCCCTACTACAACTCCTATTCATACGAATGTAATGAAGGCGAAGTCACCTGCACAGATGACAACGATGA
GTGTGAAGCCTTTATTTGTAATTGTGACCGCACGGCAGCCATCTGCTTCGCCAGAGCGTATTACAACAAG
ACGAACTGGAATATCGACATCGCTGCACGTTGCCAATGA

>GAHA01000030 Brachyurophis [Simoselaps] roperi PLA2-Bra-17 mRNA sequence
ATGTATCCTGCTAACCTTATGATCCTGTTGGCAGTTTGTGTCTCCCTCTTAGGAGCCTCCGACATTCCTC
CGATGCCTCTCAACCTCCTACAATTCGGCAACATGATTAACTGTGCCAACCGTGACAGTCGACCTTTGTT
GGATTATGCGGACTACGGTTGCTACTGCGGCAAAGGAGGTAGTGGGACACCGGTAGATGACTTGGATAGG
TGCTGCCAGTCACATGACTACTGCTATGATGAAGCCAAAATACTTCCTACATGTAATTACATGCTGAGTG
ACCCCTACTACAACTCCTATTCATACGAATGTAATGAAGGCGAACTCACCTGCAAAGATGACAACGATGG
GTGTGGAGCTTTTATTTGTAATTGTGACCGCACAGCAGCCATCTGCTTCGCCAGAGCCTATTACAACGAT
GCCAACTGGGATATCAACATCACTGCACGTTGCCAATGA

>GAHA01000031 Brachyurophis [Simoselaps] roperi VEGF-Bra-1 mRNA sequence
ATGAACTTTCTGCTCACTTGGATCCATTGCGGGTTGGCGGCACTGCTTTATTTCCACAACGCCAAGGTGT
TGCAGGCTGCTCCTGCCCAAGAGGATGGAGAGAAGCAACCAGATGAAGTTATCCCATTTATGACAGTCTT
TGAGCGGAGTGCCTGCAGGCCCATTGAGACCATGGTAGATATTTACCAGGAGTATCCTGATGAGGTGGAA
TACATGTTCAAACCATCCTGTGTGCTCCTGATGAAATGTGGAGGATTCTGTAATGACGAAGCGCTAGAAT
GTGTGCCCACAGAGGTGTACAATGTCAGCATGGAGATCATGAAACTCAGACACTTTCAGAGCCAGCATAT
ACAACTGATGAGCTTCCAGCAGCACAGTAAATGTCAA

>GAHB00000000.1|GAHB01000000 TSA: Cacophis squamulosus, transcriptome shotgun assembly, transcriptome shotgun assembly

>GAHB01000001 Cacophis squamulosus 3FTx-Cac-16 mRNA sequence
ATGAAAACTCTGCTGCTGACCTTGGTGGTGGTGACAATCGTGTGCCTGGACTTAGGTGACAGCTTGATAT
GCTACGTGGGATATAATGCCCCTCGGACTTGTCCAAATGGGCAGAAGCTATGCTATAAAATTACTTCGTG
TGTTGGTGTTTGTCCCAAGAGAGACCAGCCAATCGAAATGGGATGTGCTGCTACTTGCCCGATACGGAAC
CGCGGTGTGAAAGTTATCTGTTGCTCAACAGACAGGTGCAACAAATTATCGAATTGGGAACGTCCTTGA

>GAHB01000002 Cacophis squamulosus 3FTx-Cac-18 mRNA sequence

ATGAAAACTCTGCTGCTGACCTTGGTGGTGGTGACAATCGTGTGCCTGGACTTAGGTGACAGCTTGATAT
GCTACGTGGGATATAATGCCCCTCGGACTTGTCCAAATGGGCAGAAGCTATGCTATAAAATTACTTCGTG
TGTTGGTGTTTGTCCCAAGAGAGACCAGCCAATCGAAATGGGATGTGCTGCTACTTGCCCGATACGGAAC
CGCGGTGTGAAAGTTATCTGTTGCTCAACAGACAGATGCAACAAATTATCGAATTGGGAACGTCCTTGA

>GAHB01000003 Cacophis squamulosus 3FTx-Cac-81 mRNA sequence
ATGAAAACTCTGCTGCTGACCTTGGTGGTGGTGACAATCGTGTGCCTGGACTTAGTATACACCAGGACAT
GTTGCAACCAACAGTCATCACAACCTAAAACCACTATAGATTGTCCAAGTGGGGAGGACTCTTGCTATAA
AAGCTCTTGGAGAGGTCAACATGGTTCCTACGGTGGAATGAGAAGTGAAAGGGGATGTGGTTGCCCCCAC
GTGAAGAAAGGTATTACACTTACATGTTGCCAAACAGACGAATGCAACAATTAG

>GAHB01000004 Cacophis squamulosus 3FTx-Cac-139 mRNA sequence
ATGAAAACTCTGCTGCTGACCTTGGTGGTGGTGACAATCGTGTGCCTGGACTTAGTATACACCAGGACAT
GTTGCAACCAACAGTCATCACAACCTAAAACCACTATAGATTGTCCAAGTGGGGAGGACTCTTGCTATAA
AGGCTCTTGGAGAGGTCAACATGGTTCCTACGGTGGAATGAGAAGTGAAAGGGGATGTGGTTGCCCTCAC
GTGAAGAAAGGTATTACACTTACATGTTGCCAAACAGACGAATGCAACAATTAG

>GAHB01000005 Cacophis squamulosus 3FTx-Cac-152 mRNA sequence
ATGAAAACTCTGCTGCTGACCTTGGTGGTGGTGACAATCGTGTGCCTGGACTTAGTATACACCAGGACAT
GTTGCAACCAACAGTCATCACAACCTAAAACCACTATAGATTGTCCAAGTGGGGAGGACTCTTGCTATAA
AAGCTCTTGGAGAGGTCAACATGGTTCCTACGGTGGAATGAGAAGTGAAAGGGGATGTGGTTGCCCTCAC
GTGAAGAAAGGTATTACACTTACATGTTGCCAAACAGACGAATGCAACGATTAG

>GAHB01000006 Cacophis squamulosus 3FTx-Cac-180 mRNA sequence
ATGAAAACTCTGCTGCTGACCTTGGTGGTGGTGACAATCGTGTGCCTGGACTTAGTATACACCAGGTCAT
GTTGCAACCAACAGTCATCGCAACCTAAAACCACTAAATGTTGTGAGGAGAACTCTTGCTATAAAAAGAC
TTGGAGTGATCACCGTGGAACTATAATTGAAAGGGGATGTGGTTGCCCTCACGTGAAGAAAGGTATTAAA
CTTACATGTTGCCAAACAGATGAATGCAACAATTAG

>GAHB01000007 Cacophis squamulosus 3FTx-Cac-182 mRNA sequence
ATGAAAACTCTGCTGCTGACCTTGGTGGTGGTGACAATCGTGTGCCTGGACTTAGTATACACCAGGTCAT
GTTGCAACCAACAGTCATCGCAACCTAAAACCACTAAATGTTGTGAGGAGAACTCTTGCTATAAAAAGAC
TTGGAGTGATCACCGTGGAACTATAATTGAAAGGGGATGTGGTTGCCCTCACGTGAAGAAAGGTATTAAA
CTTACATGTTGCCAAACAGACGAATGCAACAATTAG

>GAHB01000008 Cacophis squamulosus 3FTx-Cac-203 mRNA sequence
ATGAAAACTCTGCTGCTGACCTTGGTGGTGGTGACAATCGTGTGCCTGGACTTAGTATACACCAGGTCAT
GTTGCAACCAACAGTCATCGCAACCTAAAACCACTAAATGTTGTGAGGAGAACTCTTGCTATAAAAAGAC
TTGGAGTGATCACCGTGGAACTATAATTGAAAGGGGATGTGGTTGCCCTCACGTGAAGAAAGGTATTGCA
CTTACATGTTGCCAAACAGACGAATGCAACAATTAG

>GAHB01000009 Cacophis squamulosus 3FTx-Cac-220 mRNA sequence
ATGAAAACTCTGCTGCTGACCTTGATGGTGGTGACAATCGTGTGCCTGGACTTAGGATACACCCTAGTAT
GTCTCGGAGGTTCGGGTCCTTATAAAGATGTGGTTTGTGCAAAAGACCAGACCATTTGCTATCGATATAT
TGTTTGGCCAATTAAAAGTCGTATGCTAATCATGAGGGGATGTACTTCTTCTTGCCCTGAAAGAAATAAT
AATTTGTGTTGCAGTACAGACCGGTGCAACAAGTAG

>GAHB01000010 Cacophis squamulosus 3FTx-Cac-225 mRNA sequence
ATGAAAACTCTGCTGCTGACCTTGATGGTGGTGACAATCGTGTGCCTGGACTTAGGATACACCCTAGTAT
GTCTCGGAGGTTCGGGTCCTTATAAAGATGTGGTTTGTGCAAAAGACCAGACCATTTGCTATCGATATAT
TGTTTGGCCATTTAAAAGTCGTGTTACAATCATGAGGGGATGTACTTCTTCTTGCCCTGAAAGAAATAAT
CATGTGTGTTGCAGTACAGACCGGTGCAACAAGTAG

>GAHB01000011 Cacophis squamulosus 3FTx-Cac-228 mRNA sequence
ATGAAAACTCTGCTGCTGACCTTGATGGTGGTGACAATCGTGTGCCTGGACTTAGGATACACCCTAGTAT
GTCTCGGAGGTTCGGGTCCTTATAAAGATGTGGTTTGTGCAAAAGACCAGACCATTTGCTATCGATATAT
TGTTTGGCCATTTAAAGGTCGTGTTACAATCATGAGGGGATGTACTTCTTCTTGCCCTGAAAGAAATAAT
AATGTGTGTTGCAGTACAGACCGGTGCAACAAGTAG

>GAHB01000012 Cacophis squamulosus 3FTx-Cac-236 mRNA sequence
ATGAAAACTCTGCTGCTGACCTTGATGGTGGTGACAATCGTGTGCCTGGACTTAGGATACACCCTAGTAT
GTGTCGGAGGTCCGGTTTCTTATGAAGATGTGGTTTGTGAAAAACACCAGACCATTTGCTATCGATATTC
TGTTTGTCCATTTAGAGGTCGTGTTGTAATCATGAGGGGATGTAGTGCTTCTTGCCCTGGAGGAAATAAT
CATGTGTGTTGCAGTACAGACCGGTGCAACAAGTAG

>GAHB01000013 Cacophis squamulosus 3FTx-Cac-241 mRNA sequence
ATGAAAACTCTGCTGCTGACCTTGATGGTGGTGACAATCGTGTGCCTGGACTTAGGATACACCCTAGTAT
GTGTCGGAGGTCCGGTTTCTTATGAAGATGTGGTTTGTGAAAAACACCAGACCATTTGCTATCGATATTC
TGTTTGTCCATTTAGAGGTCGTGTTGTAATCATGAGGGGATGTAGTGCTTCCTGCCCTGGAGGAAATAAT
CATGTGTGTTGCAGTACAGACCGGTGCAACAAGTAG

>GAHB01000014 Cacophis squamulosus CRiSP-Cac-1 mRNA sequence
ATGTTACAAATGGAATGGAATTCTCGTGCTGCTCAAAATGCAAAACGTTGGGCAGATAGATGTTCTTTTA
CTCACAGTCCATCACATTTAAGAACTGTGGGAAAATTCGGTTGTGGTGAAAATCTATTAATGTCAAGTCA
ACCTTTTGCATGGAGTAGAGTAATTCAGTCTTGGTATGATGAAAACAAAAAATTCGTCTATGGCGTTGGA
GCAAAGCCACCAGGTTCTGTTATTGGCCATTATACCCAGATAGTTTGGTACAAAAGTCACCTTCTTGGTT
GTGCTGCTGCCAGATGTTCTTCAACCAAATACATCTACGTTTGTCAATACTGCCCAGCAGGGAACATCAA
AGGTTCAATTGCTACTCCATATAAATCAGGGCCACCTTGTGGGGACTGTCCTTCGGCTTGTGTCAACGGA
CTATGCACAAATCCTTGCAAACATAACGATGACTTCTTGAACTGCAAATCTTTAGCGAAAGAATCTAAAT
GCCAGACTGCATGGATCAAGTCAAAATGCCCTGCTTCTTGCTTCTGCCGCACTGAAATAGTATAA

>GAHB01000015 Cacophis squamulosus CVF-Cac-3 mRNA sequence
ATGGTAGTGACTGAGCAAAGCGGCATTCATATTGTGATATCTCCCTATCAGATCTACTTCACAAAAACCC
CCAAATATTTCAAGCCAGGAATGCCATATGAACTGACGGTGTATGTTACCAACCCTGATGGCTCACCAGC
TGCCAATGTACCAGTGGTATCAGAGGCCATTCATTCTGAGGGAACCACTTTGAGCGATGGGACTGCTAAG
CTCATCCTGAACACACCACTGAACATTCAAAGCCTACCGATCACTGTTAGAACCAACCATGGAGACCTC

>GAHB01000016 Cacophis squamulosus KP-Cac-7 mRNA sequence
ATGTCTTCCGGAGGTCTTCTTCTCCTGCTGGGACTCCTCACCCTCTGGGACGGGCTGACCCCTGTCTCCA
GCAAGGACCGTCCAAAGTTCTGTGAACTGCCTCCTGACAAAGGACCATGTAAGGGCTCGTTCCTAGCCTT
CCACTACCACCCAGTTCAACAGCAGTGCCGACAGTTTACTTATGGTGGATGCCAAGGGAATCCTAACAAT

TTTAAGACCATAGAGGAATGCAAAAGCACCTGTGCTGCATGA

>GAHB01000017 Cacophis squamulosus KP-Cac-8 mRNA sequence
ATGTCTTCCGGAGGTCTTCTTCTCCTGCTGGGACTCCTCACCCTCTGGGACGGGCTGACCCCTGTCTCCA
GCAAGGACCGTCCAAAGTTCTGTGAACTGCCTCCTGACAAAGGACCATGTAAGGGCTCGTTCCTAGCCTT
CCACTACCACCCAGTTCAACAGCAGTGCCGACAGTTTACTTATGGTGGATGCCAAGGGAATCCTAACAAT
TTTAAGACCATAGAGGAATGCAAAAGCACCTGTGCTGCATGA

>GAHB01000018 Cacophis squamulosus KP-Cac-9 mRNA sequence
ATGTCTTCTGGAGGTCTTCTTCTCCTGCTGGGACTCCTCACCCTCTGGGAGGGGCTGACCCCCGTCTCCA
GCAAGGACCGTCCACACTTCTGTCATCTGCCTCATGACATCGGACCATGTAAGGCCAAGTTCCCAGCCTT
CTACTACGACCCAGTTTATCATATATGTGGAAAGTTTACTTATGGTGGATGCCAAGGGAATCCTAACAAT
TTTAAGACCATAGAGGAATGCAAACGCACCTGTGCTGCATGA

>GAHB01000019 Cacophis squamulosus KP-Cac-10 mRNA sequence
ATGTCTTCCGGAGGTCTTCTTCTCCTGCTGGGACTCCTCACCCTCTGGGAGGGGCTGACCCCTGTCTCCA
GCAAGGACCGTCCAAAGTTCTGTGAACTGCCTGCTGACAAAGGACCATGTAAGGGCTCGTTCCTAGCCTT
CCACTACCACCCAGTTCAACAGCAGTGCCTACAGTTTCTTTATGGTGGATGCCAAGGGAATCCTAACAAT
TTTGAGACCATAGAGGAATGCAAACGCACCTGTGCTGCATGA

>GAHB01000020 Cacophis squamulosus KP-Cac-14 mRNA sequence
ATGTCTTCTGGAGGTCTTCTTCTCCTGCTGGGACTCCTCACCCTCTGGGAGGGGCTGACCCCCGTCTCCA
GCAAGGACCGTCCACACTTCTGTCATCTGCCTCATGACATCGGACCATGTAAGGCCAAGTTCCCAGCCTT
CTACTACGACCCAGTTTATCATATATGTGGAAAGTTTACTTATGGTGGATGCCAAGGGAATCCTAACAAT
TTTAAGACCATAGAGGAATGCAAACGCACCTGTGCTGCATGA

>GAHB01000021 Cacophis squamulosus KP-Cac-20 mRNA sequence
ATGTCTTCTGGAGGTCTTCTTCTGCTGCTGGGACTTCTCACCCTCTGGGAGGGGCTGACTCCCGTCTCCA
GCAAGGACCGTCCAAAGTTCTGTGAACTGCCTCCTGACAAAGGAGGATGTTATGGCTGGCTCCTAGCCTT
CTACTACAATCCAGTTCAACATCAATGCCTACAGTTTATTTATGGTGGATGCCCAGGGAATGCTAACTAT
TTTAACACCATAGAGGAATGCAAAAGCACCTGTGCTGCATAA

>GAHB01000022 Cacophis squamulosus LP-Cac-4 mRNA sequence
ATGGGGCGATTCCTCTTGGTGACCCTGAGCTTGCTGGTTGTGACTTTGTCCCTAAATGGAATTGGAGCTG
ATCATCATTGCCCCTCGGGCTGGTCCTCCTTTGAGACGTTTTGCTACAAGTTCATCAGAGAAAAGAAGAG
CTGGAATGATGCCGAGGCCTCCTGCGTGCAACTTCAGAACAGCAGCCGCCTGGTCTCCATCCACAGCGGA
GCAGAGGCCCTTCACCTCAGAACGGTCATGCCCATTTGGCTCTTTGGTGATGTCTGGACTGGACTGAATG
ACCCAGGGAAAAATCGCACCTGGAAGTGGAGTGATGACTCCGATTATGATTATACATCCTGGGGATCATC
GCAACCCGGCAATACTGACCGTGCAGAGTTCTGCGTTAAGATGACCCGTAGCATATTGAAGCTTGAGTGG
AAAGCTACCACGTGTGAATCCAATCATTATTTCATCTGCAAGATGTAG

>GAHB01000023 Cacophis squamulosus NP-Cac-2 mRNA sequence
ATGCTCGCCCTCTCCCGTCTGGCGGGCGGCGGGCTGCTGGTGCTGGCCCTGCTGCCTCTCGCCCTCGACG
GGAAGCCGGCGCCGCCGCCTCAGGCGCTGCGCAAGGCTCCGGCGGGCGGCATGACGGCGTCGCGGCGGGA
GCTGACCGAGGAGCAGCAGCCGCCGCCGGCGGAGGAGTCGTCGGGTCCCGCGGCTGAGCGCAGCGGCTCC
AAGGCGGCCAAGATAGAGCTGGACAACGGCTGCTTCGGCGTCAGGCTCGACCGCATCGGCACCTCCAGCG
GCCTGGGCTGCAAAAACCCCGTCGGAAACGTCCCGACGCCGACACCTGAGGGATCCTAA

>GAHB01000024 Cacophis squamulosus NP-Cac-4 mRNA sequence
ATGCTCGCCCTCTCCCGTCTGGCGGGCGGCGGGCTGCTGCTGCTGGCCCTGCTGCCTCTCGCCCTCGACG
GGAAGCCGGCGCCGCCGCCTCAGGCGCTGCTTAAGGCTCCGGCGGGCGGCACGACGGCGTCGCGGCTGGA
GCTGACCGAGGAGCAGCAGCCGCCGCCGGCGGAGGAGTCGTCGGGTCCCGCGGCTGAGCGCAGCGGCTCC
AAGGCGGCCAAGATAGAGCTGGACAACGGCTGCTTCGGCGTCAGGCTCGACCGCATCGGTACCTCCAGCG
GCCTGGGCTGCAAAAACCCCGTCGGAAACGTCCCGACGCCGACACCTGAGGGATCCTAA

>GAHB01000025 Cacophis squamulosus NP-Cac-5 mRNA sequence
ATGGTCGCCCTCTCCCGTCTGGCGGGCGGCGGGCTGCTGGTGCTGGCCCTGCTGCCTCTCGCCCTCGACG
GGAAGCCGGCGCCGCCGCCTCAGGCGCTGCGCAAGGCTCCGGCGGGCGGCATGACGGCGTCGCGGCGGGA
GCTGACCGAGGAGCAGCAGCCGCCGCCGGCGGAGGAGTCGTCGGGTCCCGCGGCTGAGCGCAGCGGCTCC
AAGGCGGCCAAGATAGAGCTGGACAACGGCTGCTTCGGCGTCAGGCTCGACCGCATCGGCACCTCCAGCG
GCCTGGGCTGCAAAAACCCCGTCGGAAACGTCCCGACGCCGACACCTGAGGGATCCTAA

>GAHB01000026 Cacophis squamulosus NP-Cac-3 mRNA sequence
ATGCTCGCCCTCTCCCGTCTGGCGGGCGGCGGGCTTCTGCTGCTGGTGCTGGCCCTGCTGCCTCTCGCCC
TCGACGGGAAGCCGGCGCCGCCGCCTCAGGCGCTGCGCAAGGCTCCGGCGGGCGGCACGACGGCGTCGCG
GCTGGAGCTGACCGAGGAGCAGCAGCCGCCGCCGGCGGAGGAGTCGTCGGGTCCCGCGGCTGAGCGCAGC
GGCTCCAAGGCGGCCAAGATAGAGCTGGACAACGGCTGCTTCGGCGTCAGGCTCGACCGCATCGGCACCT
CCAGCGGCCTGGGCTGTAAAAACCCCGTCGGAAACGTCCCGACGCCGACACCTGAGGGATCCTAA

>GAHB01000027 Cacophis squamulosus NGF-Cac-1 mRNA sequence
ATGTCCATGCTGTGCTACACTCTGATTATAGCATTTCTGATTGGCATATGGGCAGCACCAAAATCTGAAG
ATAATGTCCCTCTGGGCTCCCCTGTAACATCTGACCTTTCTGACACCAGCTGTGCTCAAACTCACAAGGG
TCTGAAAACATCTCGAAATACAGATCAGCACCATCCTGCTCCTAAGAAGGCAGAGGATCAAGAACTTGGA
TCAGTAGCAAACATCATTGTGGATCCCAAGCTTTTTCAGAAGAGGCAGTTCCAGTCGTCTCGTGTTCTGT
TCAGCACTCAGCCCCCACGTTGTCAAGAGATGAGCAAAGTGTGGAGTTCCTGGACAATGAAGATGTTCTT
AAC

>GAHB01000028 Cacophis squamulosus PLA2-Cac-6 mRNA sequence
ATGTATCCTGCTCACCTTCTGGTCCTGTTGGCAGTTTGTGTCTCCATCTTAGGAGCCGTCACCATTCCTC
CCCTGCCTCTCAACGTCATTCAGTTCAAGAATATGATTCAATGTGCCAACCATGGCAGTCGACCTACACG
GCATTATATGAACTACGGTTGCTTCTGCGGCTCAGGAGGTCACGGGACACCGGTAGACGCCTTGGACATG
TGCTGCAAAACACATGACGACTGCTATGGTGAAGCCGAAAAACAGAGCTGCAACCCCAGGTTGACAAAGT
ATGATTACTACTGTGGCAAAGATGGACCCATCTGCAGTGTGAAAAAGAAGTGTCAACGTTTTGTGTGTGA
TTGTGACGTCACAGCAGCCAACTGCTTTGCCAGAGCCCCTTACAAAGATAGGAACTACAATATCAGCACC
AAGGCACGTTGCCAATGA

>GAHB01000029 Cacophis squamulosus PLA2-Cac-12 mRNA sequence
ATGTATCCTGCTCACCTTCTGGTCCTGTTGGCAGTTTGTGTCTCCCTCTTAGGAGCCGCCACCATTCCTC
CCCTGCCTCTCAACCTCGTACAATTCAGCTACTTGATTCAATGTGCCAACCATGGCAGTCGACCTACTTG
GCGTTATATGAACTACGGTTGCTACTGCGGCGCAGGAGGTTACGGGACACCGGTAGACGCCTTGGACATG
TGCTGCAAAGTACATGACGACTGCTATGGTGAAGCCGAAAAAAAAGGATGCGACCCCAAGTTGTTGGCGT

ATGATTACTACTGTGGCAGAGATGGACCCTACTGCAGAAATATCAAAAAGAAGTGTCTACGTGTTGTGTG
TGATTGTGACGTTGCAGCAGCCGAGTGCTTTGCCAGAACCCCTTACAACAGTAGGAACTTCGCTATCAAC
ATCAAATTACGTTGCATACCGATATATGAGAGGCTTCAGCGCTAG

>GAHB01000030 Cacophis squamulosus PLA2-Cac-16 mRNA sequence
ATGTATCCTGCTCACCTTCTGGTCCTGTTGGCAGTTTGTGTCTCCCTCTTAGGAGCCGCCACCATTCCTC
CCCTGCCTCTCAACCTCTATCAGTTCGGCCGCCTGATTCAATGTGCCATCCGCGGCAGTCGATATACTTG
GGATTATATGGACTACGGTTGCTTCTGCGGCTCAGGAGGTCACGGGACACCGGTAGACGCCTTGGACATG
TGCTGCAAAATACATGACGACTGCTATGGTGAAGCCGAAAAAAAAGGATGCGACCCCAAGTGGATGCCGT
ATACTTGGGAATGTGACAACGATGTACCCATCTGCAGTGTGAAAAAGAAGTGTCAACGTTTTGTGTGTGA
TTGTGACGCCAAAGCAGCCATCTGCTTCGCCGGAGCCCCTTACAACAAGGAGAACAACCATGTCGTCCTC
ACTGAACGTTGCCAATGA

>GAHB01000031 Cacophis squamulosus PLA2-Cac-18 mRNA sequence
ATGTATCCTGCTCACCTTCTGGTCCTGTTGGCAGTTTGTGTCTCCCTCTTAGGAGCCGCCACCATTCCTC
CCCTGCCTCTCAACCTCTATCAGTTCGGCCGCCTGATTCAATGTGCCATCCGCGGCAGTCGATATACTTG
GGATTATATGGACTACGGTTGCTTCTGCGGCTCAGGAGGTCACGGGACACCGGTAGACGCCTTGGACATG
TGCTGCAAAATACATGACGACTGCTATGGTGAAGCCGAAAAAAAAGGATGCGACCCCAAGTGGATGCCGT
ATACTTGGGAATGTGACAGCGATGTACCCATCTGCATTTCAAAAACGGGGTGTGAAAGTTCTGCGTGTGA
TTGTGACGCCAAAGCAGCCATCTGCTTCGCCGGAGCCCCTTACAACAAGGAGAACAACCATGTCGTCCTC
ACTGAACGTTGCCAATGA

>GAHB01000032 Cacophis squamulosus VEGF-Cac-1 mRNA sequence
ATGAACTTTCTGCTCAGTTGGATCCATTGCGGGTTGGCGGCGCTGCTTTATTTCCACAACGCCAAGGTGT
TGCAGGCTGCTCCTGCCCAAGAGGATGGAGAGAAGCAACCAGATGAAGTTATCCCATTTATGACGGTCTT
TGAGCGGAGTGCCTGCAGGCCCATTGAGACCATGGTAGATATTTACCAGGAGTATCCTGATGAGGTGGAA
TACATGTTCAAACCATCCTGTGTGCTCCTGATGAAATGTGGAGGATTCTGTAATGACGAAGCGCTAGAAT
GTGTGCCCACAGAGGTGTACAATGTCAGCATGGAGATCATGAAACTCAGACACTTTCAGAGCCAGCATAT
ACAACTGATGAGCTTCCAGCAGCACAGTAAATGTCAATGCAGACAAAAGAAACCAATGAGAATTAAACAA
GAAAATCACTGTGAACCTTGCTCAGAAAGGAGAAAGCACTTGTACAAACAAGATCCCTTGACCTGTAAAT
GTTCCTGCAAATTCACAGACTCACGTTGCAAGTCGAAGCAGCTTGAGTTAAACGAGCGCACTTGCAGATG
TGAAAAACCAAGGCGGTGA

>GAHB01000033 Cacophis squamulosus WP-Cac-1 mRNA sequence
ATGAAACCTTGGATTCTCCTGTTCCTAGCTGGTCTCCTCATCCTTTCAACTCAACTGGCAACAGCAAAGA
CCACCAAACGACGTTGTCCCCCAAAAGTCAAGCCGGGCGAGTGTCCCAAGGTCAAAATTGATCCTGACCA
TCCATGTAACCAAGACTGTGCCTGCGACTCTGGATGCCAAGGGAATAAGAAATGCTGCCCAGTCGGATGC
GCCAGAGAATGTTTTAGCCCAGGACCCCTCTGA

>GAHB01000033 Cacophis squamulosus KP-Cac-21 mRNA sequence
ATGGTGTCTGGAGGTCTTCTTCTCCTGCTGGGACTCCTCACCCTCTGGGCAGAGCTGACCCCCGTCTCCG
GCCAGGACCGTCCAAGGTTTTGTCATCTTCCTGCTGACCCCGGACCATGTAGAGCAAAAATAACTCGCTT
TTACTACAACTCGGGTTCAAAACAATGTGAAAAGTTTATTTATGGCGGATGCCAGGGCAATGCCAACAAT
TTTGAGACCAAAGATAGATGCCACTACACCTGTGTGGAAAAACCTGGAGTGTGTCCCCAGGCCCCTCAAC
AACAACAACAACAAAAACTTCCTTGTCTCGAGAGGTGTCAACATGACTGGAACTGTCCTGGGGAGCAGAA
GTGTTGCCGCTACGGTTGCCTGTCTGAGTGCAAGGACATTGTCTATTCGATCCATGCATCAGCAACGAAG
AGACCCTCCCGGATTGGAACAACTGTTCGCACTTGA

>GAHC00000000.1|GAHC01000000 TSA: Denisonia devisi, transcriptome shotgun assembly, transcriptome shotgun assembly

>GAHC01000001 Denisonia devisi 3FTx-Den-1 mRNA sequence
ATGAAAACTCTGCTGCTGACCTTGGTGGTGGTGACAATCGTGTGCCTGGACTTAGGTGACAGCTTCTCAT
GCTACAAAACACCTGGTCTGGAATCTGAGGCTTGTGCACCTGGGGAGAACCTATGCTATACAAGGACTTG
GTGTGATGCTCGGTGTGGCCTCATGGGAAAGCGAATCGAACTGGGATGTGCTGCTACTTGCCCTACAGCG
AAGACCGGCATCGATGTTATCTGTTGCTCAACAGACAATTGCAACCCACATCCGAAACGGAAACCTCATT
GA

>GAHC01000002 Denisonia devisi 3FTx-Den-5 mRNA sequence
ATGAAAACTCTGCTGCTGACCTTGGTGGTGGTGACAATCGTGTGCCTGGACTTAGGTGACAGCTTCTCAT
GCTACAAAACACCTGGTCTGGAATCTGAGGCTTGTGCACCTGGGGAGAACCTATGCTATACAAAGACTTG
GTGTGATCGTTTTTGTGGCGTCATGGCAAAGCGAATCGAACTGGGATGTGCTGCTACTTGCCCTGAAGGG
AGGCCCGGCATCGATGTTACCTGTTGCTCAACAGACAATTGCAACCCACATCCGAAACGGAAACCTCATT
GA

>GAHC01000003 Denisonia devisi 3FTx-Den-9 mRNA sequence
ATGAAAACTCTGCTGCTGACCTTGGTGGTGGTGACAATCGTGTGCCTGGACTTAGGTAACAGCTTCTCAT
GCTACAAAACACCTGATGTTAACTCTGAGCCTTGTGCACCTGGGGAGAACCTATGCTATACAAAGACTTG
GTGTGATCGTTTTTGTTCCAGCAGAGGAAAGGTAATCGAACTGGGATGTGCTGCTACTTGCCCTCCAGCG
GAGCCAAAAAAGGATATTACCTGTTGCTCAACAGACAATTGCAACCCACATCCGGCACACAAATCTCGTT
GA

>GAHC01000004 Denisonia devisi 3FTx-Den-15 mRNA sequence
ATGAAAACTCTGCTGCTGACCTTGGTGGTGGTGACAATCGTGTGCCTGGACTTAGGTGACAGCTTCTCAT
GCTACAAAACACCTGATGTTAACTCTGAGGTTTGTGCACCTGGGGAGAACCTATGCTATACAAGGACTTG
GTGTGATGCTTGGTGCGGCAGCAGAGGAAAGCGAATCGAACTGGGATGTGCTGCTACTTGCCCTCCAGCG
GAGCCAAAAAAGGATATTACCTGTTGCTCAACAGACAATTGCAACCCACATCCAGCACACAACTCTCGTT
GA

>GAHC01000005 Denisonia devisi ACN-Den-1 mRNA sequence
ATGCCTGCGCCGTGGCCTTGGTGGCTCCAGCTTGTCCTTTGCATCCTGTCCTCCGTAGCAGTCTTGCCTG
GCCGGGCAAGCGAGCTGAAAGTGTCCACGCAGATGGGCCTGGTGCGTGGCCTGAGCCTGCCGGTTCTCGA
CGGCCACGTCTCGGCCTTCCTGGGCATCCCCTTTGCGGAGCCTCCCGTGGGCAGGATGCGCTTCCTGCGC
CCTGAGCCCGTCAAGCCCTGGCAGCACATCCTGGACGCCACTTCCTACCAGCGCGCCTGTTACCAGGCGG
TGGACAACAGCTACCCAGGCTTCCAGGGCACGGAGATGTGGAACCCCAACCGGGGCATGAGCGAGGACTG
CCTCTACCTCAACATCTGGGTGCCCTCCCCGCGTCCCAGAGACGTCCCCGTCCTGGTCTGGATCTACGGA
GGGGGCTTCTACAGCGGCGCTGCCTCCCTGGACGTCTACGATGGCCGCTTCCTGACCTACACACAGAACG
TTATCGTGGTCTCCCTCAGCTACCGCGTGGGGGCCTTCGGCTTTTTGGGCCTCCCGGGCAGTCCGGAGGC
GCCGGGGAACATGGGGCTGCTGGACCAACGCTTGGCCCTGCAGTGGATCCAGAACAACATCCATCATTTT
GGGGGCAATCCCAGCGCGGTCACCATCTTTGGGGAGAGTGCCGGGGCCGCCTCGGTGGGCATGCACCTGC

TTTCCACCCAGAGCCGCGCCCTCTTCCAGCGGGCCATTCTGCAGAGCGGCGGCCCCAACGCACCGTGGGC
CACCGTCACGCCTGCCGAAAGCCGGCGCCGGGCGGCCCTTCTGGGAAAGCAGCTGGGCTGCCAGTTCAAC
AACGATTCCGAGCTGGTCAGTTGCTTGCGCTCGAAAACCCCGCAGGAGCTGATCGACGAGGAGTGGTCCG
TCCTGCCCTACAAGAGCATTTTCCGCTTCCCTTTCGTCCCGGTCATTGACGGGGACTTTTTTCCGGACAC
CCCGGAGGCCATGCTGAGTTCGGGCAACTTCAAAGAGACCCAGGTGTTGCTGGGCGTGGTGAAGGACGAG
GGCTCCTACTTTCTGATCTACGGGCTGCCCGGCTTCAGCAAGGACAACGAGAGCCTCATCAACCAGGCCG
ACTTCTTGGAAGGGGTGCGGATGAGCGTGCCTCACGCCAACGACATCGCCACCGAGGCCGTGGTGATGCA
GTACACTGACTGGCAGGACCAGGACAACGGGGAGAAGAACAGGGAGGCCCTGGACGACATCGTGGGAGAC
CACAACGTCATCTGCCCGGTCGTGCAGTTCGCCAACGACTACGCCAAGCGCAACAACAAGGTGTACGCCT
ACCTCTTTGACCACCGGGCCTCCAACCTGCTCTGGCCTCCCTGGATCGGGGGTCCCCCACGGCTACGAGA
TCGAGTTTGTCTTTGGATTGCCCCTCAACGACAGCCTGAACTACACACCCCAAGAGAAGGAGCTGAGCCG
CAGGATGATRCGCTACTGGGTCAACTTYGCCCGGACAGGGAACCCCACCGACCCTGCCGAGAAGGACGGG
GCCTGGCCCACCTACACCGCCTCCCGGCCGCAGTATGTCCAGCTCAACACCCAGCCGCTGGCCACCCAAC
CCAGCCTGCGGGCCCAGATCTGCGC

>GAHC01000006 Denisonia devisi CRiSP-Den-3 mRNA sequence
ATGATTGCCTTCATTGTCTTGCTAAGTCTTGCTGCAGTGCTGCAACAGTCTTCTGGAACTGTTGATTTTG
CTTCTGTGTCAAGTAACAAAAAGAATCACCGAAAGGAGATTGTTGACAAGCACAATGCTTTAAGGAGATC
AGTGAAACCAACTGCTAGGAACATGTTACAAATGAAATGGAATTCTCGTGCTGCTCAAAACGCAAAACGT
TGGGCAGATAGATGTACTTTTGCTCACAGTCCACCAGGTACAAGAACTGTGGGAAAACTCCGTTGTGGTG
AAAATATATTCATGTCAACTCAACCTTTTCCATGGAGTAGCGTAGTTCAGGCTTGGTATGATGAAGTCAA
AAATTTCGTCTATGGCATTGGAGCAAAGCCACCAGGTTCTGTTATTGGCCATTATACCCAGGTAGTTTGG
TACAAAAGTCACCTTCTTGGTTGTGCTTCTGCGAAATGTTCTTCAACCAAATACCTCTACGTTTGTCAAT
ACTGCCCAGCAGGGAACATCAGAGGTTCAATTGCTACTCCATATAAATCAGGGCCAGCTTGTGCGGACTG
TCCTTCAGCTTGTGTCAACGGACTATGCACAAATCCTTGCAAATATAAGGATGACTTCTCGAACTGCAAA
GCTTTAGCGAAAAAACTAAATGCCAGACTGCATGGATCAAGTCAAAATGCCCTGCTACTTGCTTCTGCCA
CAATAAAATAA

>GAHC01000007 Denisonia devisi cystatin-Den-1 mRNA sequence
ATCCCCGGGGGCCTTTCCCCGAGATCCGTCACGGATCCGGACGTGCAGGAAGCCGCCGAGTTCGCCGTGC
AAGAATACAACGCGCTCAGCGCAAACGCCTACTATTACAAGCAGTTGCGCATTGTGGAGGCGCAGTCCCA
GGTAGTCTCAGGGGAGAAATATTATCTGACGATGGAGCTGATGAAAAMCCAAGTGTGCAAAGACCACCGG
CAAGCCAAAGGTGTACAAAGAGATCCAGAACTGTGA

>GAHC01000008 Denisonia devisi KP-Den-3 mRNA sequence
ATGTGCAAGATGTTCATCTATGGGGGCTGTCGTGGCAACAAGAACAACTATCTCTTCGAAGAGCACTGCT
GGAGCCAGTGCACAGGTGATGGAGAGATAACCAAAGAGCCAGGAGACGCAGGCGCCCGCCCTTCCCTCCC
TCTGAACCCTTCAGGTTCTCCACCAGAGCTGTGGTCCTTGCCGTCCTTCTGGCCATCCTGGTGA

>GAHC01000009 Denisonia devisi LAO-Den-1 mRNA sequence
ATGAATGTCTTCATTATTTTCTCACTGCTGTTCTTGGCTGCCTTGGAAAGCTGTGCAGATGACAGAAGAA
GGCCCCTAGAAGAATGCTTCCGAGAAGCTGATTATGAAGAATTTCTAGAGATCGCCAGGAATGGTTTGAA
CAAGGCATCAAACCCAAAACATGTTGTGGTTGTAGGTGCAGGAATGGCCGGGCTTAGTGCAGCCTACGTT
CTTGCAGAGGCTGGACATAAGGTGACGCTTCTTGAAGCTAGTAAACGTGTGGGAGGGCGAGTGAACACTT
ATCGAAATGAAACGGAAGGCTGGTATGTCAATCTGGGACCCATGCGTCTCCCTGAGAGACACAGGATTAT
CCGGGAATATATCAAAAAATTTGGTCTCAAGTTGAATGAATTTCTTCAGGAAAATGAGAATGCCTGGTAT
TTTATTAAAAACATCAGGAAGAGAGTATGGGAAGTCAAGAAAGACCCCGGCGTCTTCAAATATCCTTTGG
AGCCTTCAGAAGAAGGCAAATCTGCTTCCCAGCTATATCGAGAATCCCTCGAAAAGGTTATAGAAGAATT
GAAAAGGACTAACTGCAGCTACATACTAAATAAATATGACACCTACTCAACAAAGGAGTATCTAATTAAA
GAAGGAAATCTGAGCCGCGGAGCTGTAGATATGATTGGAAAGTTACTGAATGAAGATTCTAGCTATTACC
TGTCTTTTATTGAGAGCCTGAAAGGTGATGATCTCTTCTCTTACGAAAAAAGATTTGATGAAATTGTTGG
TGGATTTGATCAATTGCCTAAATCCATGTATCAAGCCATTGCGAAAATGGTGCATTTAAATGCTCAAGTA
ATCAAGATACAACATAATGCTGAGGAAGTCAGAGTGGCATATCAAACTCCAGCAAAGACCTTGTCATCTG
TGACAGCCGATTATGTCATTGTATGCTCCACATCAAGGGCCGCCCGTCGCATCTATTTTGAACCTCCCCT
TCCAGCAAAGAAAGCACACGCTTTGCGGTCTATCCACTACAGAAGTGGCACCAAGATCTTCCTCACTTGC
TCTAAGAAGTTTTGGGAGGCTGATGGCATTCATGGTGGGAAGTCCACAACAGATCTTCCATCTCGATTCA
TCTACTACCCTAACCATAACTTTACTAGTGGCGTTGGAGTTATTGTGGCCTATACCCTTGCTGATGATGC
CGATTTCTTTCAAGCTCTTGATATCAAGACCAGTGCTGATATTGTCATTAATGACCTTTCACTCATCCAT
CAGCTGCCCAAGGAAGAGATCCAGGCCCTCTGCTACCCCTCAGTGATTAWWAAATGGAGCCTGGATAAAT
ATGCTATGGGTGCTATAACCTCTTTCACTCCTTACCAGTTTCAAGATTTTGTTGAAACAGTCGCTGTACC
TGTAGGCAGAATCTACTTTGCAGGGGAGTACACTGCCAGAGCTCATGGTTGGTTGGACAGCACAATTAAG
TCAGGGCTGACAGCAGCAAGAGATGTGAATCGTGCTTCTCAGAAGCCATCAAGAATCCAGCTGATCAGTG
ACAATCAACTTCAAGAAGGAGGTCAACACAAGGAGAATCCTAAACAGCCACTTTCCCCATTCTGGCAACT
TTCAGGTTTTTCAGACTAA

>GAHC01000010 Denisonia devisi LP-Den-2 mRNA sequence
ATGGGGCGATTCCTCTTTGCGAGCCTCGGCTTGCTGGTCGTGGCTTTCTCCCTAAGTGGAATTGGAGCCA
ATCGCTATTGTCCCTTTGATTGGTTCTCCTACAATGAGTTTTGCTACAAGCTCTTCTACTTTCCGAAGAC
CTGGGACGAAGCACAGAGGTTCTGTGTGGAACAGCAGGAAAACTCCCAACTTGCCTCCATCAATGACGTG
GGAGAATCAGTGAAGTTGTCCAACTACATCTCCCAAAGATTGAAATTCTTTGATGTTTGGATGGGACTGA
GGCTTTCAAAGAGAAAAGGCATCTGGGAGTGGAGTGATGGCTCCAACCTCACTCACACATCCTGGAAAGA
AGGTGAACCCAACAACCTTTTCAACAAGGAATTCTGTGCTGTGCTGTCAGCTGAAACATGGTATCTCCAA
TGGAACGATAAAAAGTGCACGCACAAGCATCCATTTGTCTGCAAGTTCCAGCCCCGGAGTGAGGCAAACG
GCTAA

>GAHC01000011 Denisonia devisi NP-Den-13 mRNA sequence
ATGCTCGGCCTCTCACGTCTGGCGGGCGGCGGGCTGCTGCTGCTGGTGCTGGCCCTGCTGCCTCTCGCCC
TCGACGGGAAGCCGGCGCCGCTGCCTGAGGCGCTGCCCACGGCTCCGGCGGGCGGCATGAAGGCGTCGCG
TCCGGTGCTGACGGAGCAGCAGAAGCAGCAGAACCAACAGCAGAACCAGCACCAGCATCCGCTGCCGCCG
ATGGAGGAGTCGTCGGGTCCCGCGGCTGAGCGCAGCGGCTCCGAGACGGCCAAGATAGGGGACGGCTGCT
TCGGCGTCCCGATCGACCACATCGGCAGCTCGAGCGGCATGGGCTGCGGAGGCGTCCCGAAACCTGGCGG
ATCCTAA

>GAHC01000012 Denisonia devisi NP-Den-10 mRNA sequence
ATGCTCGGCCTCTCACGTCTGGCGGGCGGCAGGCTGCTGCTGCTGGTGCTGGCCCTGCTGCCTCTCGCCC
TCGACGGGAAGCCGGCGCCGCTGCCTGAGGCGCTGCCCACGGCTCCGGCGGGCGGCATGAAGGCGTCGCG
TCCGGTGCTGACGGAGCAGCAGAAGCAGCAGAACCAACAGCAGAACCAGCACCAGCATCCGCTGCCGCCG
ATGGAGGAGTCGTCGGGTCCCGCGGCTGAGCGCAGCGGCTCCGAGACGGCCAAGATAGGGGACGGCTGCT
TCGGCGTCCCGATCGACCACATCGGCAGCTCGAGCGGCATGGGCTGCGGAGGCGTCCCGAAACCTGGCGG
ATCCTAA

>GAHC01000013 Denisonia devisi NP-Den-9 mRNA sequence
ATGCTCGGCCTCTCACGTCTGGCGGGCGGCGGGCTGCTGCTGCTGCTGCTGGCCCTGCTGCCTCTCGCCC
TCGACGGGAAGCCGGCGCCGCTGCCTGAGGCGCTGCCCACGGCTCCGGCGGGCGGCATGAAGGCGTCGCG
TCCTTTGCTGACGGAGCAGCAGAAGCAGCAGAACCAACAGCAGAACCAGCACCAGCATCCGCTGCCGCCG
CCGCCGATGGAGGAGTCGTCGGGTCCCGCGGCTGAGCGCAGCGGCTCCGAGACGGCCAAGATAGGGGACG
GCTGCTTCGGCGTCCCGATCGACCACATCGGCAGCTCGAGCGGCATGGGCTGCGGAGGCGTCCCGAAACC
TGGCGGATCCTAA

>GAHC01000014 Denisonia devisi NP-Den-19 mRNA sequence
ATGCTCGGCCTCTCACGTCTGGTGGGCGGCGGGCTGCTGCTGCTGGTCCTGCTGCCTCTCGCCCTCGATG
GGAAGCCGGCGCCGCTGCCTCAGGCGCTGCCCAAGGCTCCGGCGGGCGGCATAAAGGCGTCGCGTCCGGT
GCTGACGGAGCAGCAGAAGCAGCAGAACCAACAGCAGAACCAGCACCAGCATCCGCTGCCGCCGCCGCCG
ATGGAGGAGTCGTCGGGTCCCGCGGCTGAGCGCAGCGGCTCCGAGACGGCCAAGATAGGGGACGGCTGCT
TCGGCGTCCCGATCGACCACATCGGCAGCTCGAGCGGCATGGGCTGCGGAGGCGTCCCGAAACCTGGCGG
ATCCTAA

>GAHC01000015 Denisonia devisi NGF-Den-2 mRNA sequence
ATGATGTATAGCGTAATGTCCATGTTGTGCTACACTCTGATTATTGCATTTCTGATTGGCATATGGGCAG
CACCAAAATCTGAAGATAATGTCCCTCTGGGCTCCCCTGCAACCAGCTGTGCTCAAACTCACGAGGGTCT
GAAAACATCTCGAAATACAGATCAGCGCCCTCCTGCTCCTAAGAAGGCAGAGGATCAAGAACTTGGATCA
GTAGCAAACATCATTGTGGATCCCAAGCTTTTTCAGAAGAGGCGTTTCCAGTCACCTCGTGTTCTGTTCA
GCACTCAGCCCCCACCATTGTCAAGAGATGAGCAAAGTGTGGAGTTCCTCGACAATGAAGATGCTCTTAA
CAGGAATATCCGGGCCAAACGTGAAAATCATCCTGTGCATAACCTAGGGGAACATTCTGTGTGTGACAGT
ATCAGTGGCTGGGTTAACAAAACCACAGCAACGGACATGTACGGCAATATGGTGACTGTGATGGCGGAGA
TAAATCTTAATAATGAAGTCTATAAGCAGTACTTTTTTGAAACCAAGTGCAGAAATCCAAATCCAAATCC
ACTACTGAGTGAGTGCAGGGGCATTGATTCCAGGCTTTGGAATTCTTATTGCACCAGAACACAGACATTT
GTCAGGGCATTAACCATGGCAGGCAATGTGACATCCTGGCGCTTCATTCGGATTGACACTGCCTGTGTGT
GTGTAATCATTAGAAAAACTGACAACTTCTGA

>GAHC01000016 Denisonia devisi PLA2-Den-2 mRNA sequence
ATGTATCCTGTTCACCTTCTGGTTCTGTTGGCAGTTTGTGTCTCCCTCTTAGGAGCCGTCAGCATTCCTC
CGCGGCCTCTGAACCTCTTTCAGTTCAAGGACATGATTGAATGTGCCAACCATGGCAGTCGAGGTGCATT
GGATTATGCGGACTACGGTTGCTACTGCGGCAAAGGAGGTAGCGGGACACCGGTAGATGAGTTGGATAGG
TGCTGCCAAACACATGATGACTGCTATGGTGAAGCCGAAAAACTTCCTGCATGTAATTACATGTTGAGTG
GCCCCTACTACAACACCTTTAAATACGACTGTGTTGAACACCAACTCACCTGCAAAGATGACAAGGATGA
GTGTAGAGCCTTTATTTGTAATTGTGACCGCACAGCAGCCATCTGCTTCGCCGGAGCCCCTTACAACAAC
ACGAACTACAATATCGACACCAAGACACATTGCCAATGA

>GAHC01000017 Denisonia devisi PLA2-Den-9 mRNA sequence
ATGTATCCTGCTCACCTTCTGGTCCTGTTGGCAGTTTGTGTCTCCCTCTTAGGAGCCTCCGACATTCGTC
CGCGGCCTCTCAACCTCGTACAATTTGGCAACATGATTCAATGTGCCAACCATGGCAGTCGACCTACTCG
GCATTATATGGACTACGGGTGCTACTGCGGCTGGGGAGGTAGCGGGACACCGGTAGATGAGTTGGATGGG
TGCTGCAAAACACATGACGACTGCTATGGTGAAGTCGAAAAAAAAGGATGCTACCCCAAGTTGTCGTTCT
ATAGTTGGAAATGTACTGAAAACGTACCCACCTGCGATTCAGAAACGGGGTGTAAAGCTTCTGTGTGTGC
TTGTGACGCCGCAGCAGCCAAGTGCTTTGCCAAAGCCCCTTACAACAACGCAAACTGGAATATCAACACT
GACACACGTTGCTAA

>GAHC01000018 Denisonia devisi PLA2-Den-12 mRNA sequence
ATGTATCCTGCTCACCTTCTGGTCCTGTTGGCAGTTTGTGTCTCCCTCTTAGGAGCCGCCAACATTCCTC
CGCAGCCTCTGAACCTCGTACAATTCAGCTACTTGATTCAATGTGCCAACCATGGCAGTCGACCTACTCG
GCATTATCTGGACTACGGTTGCTACTGCGGCTCAGGAGGTAGCGGGACACCGGTAGATGAGTTGGATAGG
TGCTGCCAAACACATGACGACTGCTATACTCAAGCCGGAAACAAAGGATGCTACCCCAAGTTGTTGGCGT
ATGATTACTACTGTGGCGAAAATGGACCCTACTGCAGAAATATCAAAAAGGAGTGTCAACGTTTTGTGTG
TGATTGTGACGTTGAAGCAGCCAAGTGCTTTGCCAAAGCCCCTTACAACGACGCGAACTGGAATATCGAC
ACCACGAAACGTTGCAAATGA

>GAHC01000019 Denisonia devisi SVMP-Den-9 mRNA sequence
ATGATCCAAGCTCTCTTGGTAACTATATGCTTCACGGTTTTTCCATATCAAGGGAGCTCTATCATCCTGG
AATCCGGGAATGTTAATGATTATGAAGTAGTGTATCCACAAAAAGTGCCTGCATTGCCCAAAGGAGGAGT
TCAGAATCCTCAGCCAGAGACCAAGTATGAAGATACAATGCAATATGAATTTCACGTGAATGGAGAGCCA
GTGGTCCTTCACTTAGAAAGAAATAAAGGACTTTTTTCAGAAGATTACACTGAAACTCATTATGCCCCTG
ATGGCAGAGAAATTACAACAAGCCCTCCGGTTCAGGATCACTGCTATTACCATGGCTACATTCAGAATGA
AGCTGATTCAAGTGCAGCCATCAGTGCATGCAACGGCTTGAAAGGACATTTCAAGCATCGAGGGGAGACA
TACTTTATTGAGCCCTTGAAGCTTTCCGACAGTGAATCCCATGCAATCTACAAAGATGAAAATGTAGAAA
AAGAGGATAAGATCCCCAAAATCTGTGGGGTAACCCATACTACTTTGGAGTCGGAGGAGCCCATCGAAAA
GATCTCTCAATTAACTAATACTCCTGAACAAGACAGGTACTTGCAGGTCAAAAAATACATTGAGTTGTAT
ATAGTTGTGGACAAGGAAATGTACAAGAATTACAGCAGCAACAGAGATGCTACAAAAACTGGAGTATATG
CAATGGTCAACACTTTAAATGTGATGTACAGACCTTTGAATTTTTTCATTGCACTGATTGGCCTAGAAAT
TTGGACCAACAAAGATCAGATTAAGATTGAACAAGACGCGAATGTCACTTTGAAATCATTTGGAGAATGG
AGAGAGAAAAATTTGCTGCCACGCAAACGGAATGATAATGCTCAGTTACTTACGGGCATTGACTTCAATG
GAAGGACTGTAGGACTTGCTTACGTCGGCTCCCTCTGCATTCCGACGCAATCTGTAGCAGTTATTCAGAA
TTATAGCAGAGACACAAGAATAATGGCATCTATAATGGCCCATGAGATGGGTCATAATCTGGGCATTAAT
CATGACAGCGCTTCCTGTAATTGCAATGCTGGACCATGCATTATGTCTGACACAGTAAGTAGTCAACCTG
CCTATTGGTTCAGCTCTTGTAGTGTCCAGGAACATCAGAGGTATCTTCTTAGAGTCAAACCACAATGCAT
TCTCAACAAACCCTTGAGCACAGATATTGTTGCACGTCCAGTTTGTGGAAATTACTTTGTGGAGGTGGGA
GAAGAATGTGACTGTGGCTCTCCTCAGGATTGTCAAAGTGTCTGCTGCAATGCTGCAACTTGTAAACTGC
AACATGAGGCACAGTGTGACTCCGGAGAGTGTTGTGAGCAATGCAAATTTAAGAAAGCAGGAGCAGAATG
CCGGGCAGCAAAGGATGACTGTGACTTGCCTGAAAGCTGCACTGGCCAATCTGCTGAGTGTCCCACGGAC
CGCTTCCAGAGGAATGGACATCCATGCCAAAACAACAAGGGTTACTGCTACAATGGGAAATGCCCCATCA
TGACAAACCAGTGTATTGCTCTCGGGGGACCAGGTGTAAATGTGTCTCCAGATATATGTTTTCCGTTGAA
CAAGGATGGCCAAGGTTGTGGCTTCTGCAGAATAGAAAATGGTAGAAAGATTCCATGTGCAGCAAAGGAT
GTAAAGTGTGGCATGCTATACTGCGAAAGGGGAAAGGATACATGCACCTGCTTTATTTCAAGACATGACC
CAGATTCTGGAATGGTTGAACCTGGAACAAAATGTGGAGATGGAAAGGTGTGCATCAACAGGCAGTGTGT
TGATGTGCAGACAGCCTACTGA

>GAHC01000020 Denisonia devisi VEGF-Den-1 mRNA sequence
ATGAACTTTCTGCTCACTTGGATCCATTGCGGGTTGGCGGCGCTGCTTTATTTCCACAACGCCAAGGTGT
TGCAGGCTGCTCCTGCCCAAGAGGATGGAGAGAAGCAACCAGAAGAAGTTATCCCATTTATGACGGTCTT
TGAGCGGAGTGCCTGCAGGCCCATTGAGACCATGGTAGATATTTACCAGGAGTATCCCGATGAGGTGGAA
TACATGTTCAAACCATCCTGTGTGCTCCTGATGAAATGTGGAGGATTCTGTAATGACGAAGCGCTAGAAT
GTGTGCCCACAGAGGTGTACAATGTCAGCATGGAGATCATGAAACTCAGACACCTTCAGAGCCAGCATAT

ACAACTGATGAGCTTCCAGCAGCACAGTAAATGTCAATGCAGACAAAAGAAACCAATAAGAACTAAACAA
GAAAATCACTGTGAACCTTGCTCAGAAAGGAGAAAGCACTTGTACAAACAAGATCCCTTGACCTGTAAAT
GTTCCTGCAAATTCACAGACTCACGTTGCAAGTCGAAGCAGCTTGAGTTAAACGAGCGCACTTGCAGGTG
TGACAAACCGAGGCGGTGA

>GAHC01000021 Denisonia devisi WP-Den-30 mRNA sequence
ATGCAGGCCAGATTCCTCCTCCTGTTCACAGTGATCCTGCTGGGGATGATGGGGCCTGTGATCTCCACTG
GAATTGAGAAAGCCGGGTCCTGTCCAGATGTGAACATGCCCATCCCACCTCTGGGTATTTGCAGAACCAC
GTGTCAAACAGATTCGAACTGTCCAGACATCAAGAAGTGCTGCAAAAATGGCTGCGGGTTCATGACTTGT
TCAACGCCCAAAGCCTGA

>GAHD00000000.1|GAHD01000000 TSA: Echiopsis curta, transcriptome shotgun assembly, transcriptome shotgun assembly

>GAHD01000001 Echiopsis curta 3FTx-Ech-9 mRNA sequence
ATGAAAACTCTGCTGCTGACCTTGGTGGTGGTGACAATCCTGTGCCTGGACTTAGGTAACAGCATGTCAT
GCTACAAAACACCTGATGTTAAATCTGAGCCTTGTCCACCTGGGCAGAACCTATGCTATACAAAGACTTG
GTGTGATGCTTTTTGTTCCATCAGAGGAAAGGTAATCGAACTGGGATGTGCTGCTACTTGCCCTCCAGCG
GAGCCAAGAAAGGATATTACCTGTTGCTCAACAAACAATTGCAACCCACATCCGGCACACAACTCTCGTT
GA

>GAHD01000002 Echiopsis curta 3FTx-Ech-12 mRNA sequence
ATGAAAACTCTGCTGCTGACCTTGGTGGTGGTGACAATCCTGTGCCTGGACTTAGGTAACAGCATGTCAT
GCTACAAAACACCTGATGTTAAATCTGAGCCTTGTCCACCTGGGCAGAACCTATGCTATACAAAGACTTG
GTGTGATGCTTTTTGTTCCATCAGAGGAAAGGTAATCGAACTGGGATGTGCTGCTACTTGCCCTCCAGCG
GAGCCAAGAAAGGATATTACCTGTTGCTCAACAAACAATTGCAACCCACATCCGGCACACAACTCTCGTT
GA

>GAHD01000003 Echiopsis curta 3FTx-Ech-21 mRNA sequence
ATGAAAACTCTGCTACTGACCTTGGTGGTGGTGACAATCGTGTGCCTGGACTTAGGTGACAGCTTGATAT
GCTACAAGGGATATAATGCCCCTCAGACTTGTCCACCTGGGCAGAACCTATGCTATACAAAGACTTGGTG
TGATGCTTTTTGTTCCAGCAGAGGAAAGGTAGTCGAATTGGGATGTGCTGCTACTTGCCCTACAGTGAAG
CCCCATGTGGATATTAAGTGTTGCTCAACAGACAAGTGCAACCCATTTCCAGTACGTCCTAGATCACGTC
CTTGA

>GAHD01000004 Echiopsis curta 3FTx-Ech-25 mRNA sequence
ATGAAAACTCTGCTGCTGACCTTGGTGGTGGTGACAATCGTGTGCCTGGACTTAGGTGACAGCTTGATAT
GCTACAAGGGATATAATGCCCCTCAGACTTGTCCACCTGGGCAGAACCTATGCTATACAAAGACTTGGTG
TGATGCTTTTTGTACCAGCAGAGGAAAGGTAGTCGAATTGGGATGTGCTGCTACTTGCCCTACAGTGAAG
CCCCATGTGGATATTAAGTGTTGCTCAACAGACAAGTGCAACCCATTTCCAGTACGTCGTAGATCACGTC
CTTGA

>GAHD01000005 Echiopsis curta 3FTx-Ech-35 mRNA sequence
ATGAAAACTCTGCTACTGACCTTGGTGGTGGTGACAATCGTGTGCCTGGACTTAGGTAACAGCATGTCAT
GCTACAAAACACCTGGTGTTAAATCTGAGCCTTGTCCACCTGGGCAGAACCTATGCTATACAAAGACTTG
GTGTGATGCTTGGTGTACCAGCAGAGGAAAGGTAGTCGAATTGGGATGTGCTGCTACTTGCCCTATACCA
AAGTCCTATGAGGATATTAAGTGTTGCTCAACAGACAATTGCAACACATTTCCACTACGTCCTAGATCAC
GTCCTCGAGGGTTGCTCTCATCCATCATGGACCATCCTTGA

>GAHD01000006 Echiopsis curta 3FTx-Ech-37 mRNA sequence
ATGAAAACTCTGCTACTGACCTTGGTGGTGGTGACAATCGTGTGCCTGGACTTAGGTAACAGCATGTCAT
GCTACAAAACACCTGGTGTTAAATCTGAGCCTTGTCCACCTGGGCAGAACCTATGCTATACAAAGACTTG
GTGTGATGCTTGGTGTACCAGCAGAGGAAAGGTAGTCGAATTGGGATGTGCTGCTACTTGCCCTATACCA
AAGTCCTATGAGGATATTAAGTGTTGCTCAACAGACAATTGCAACACATTTCCACTACGTCCTAGATCAC
GTCCTCGAGGGTTGCTCTCATCCATCATGGACCATCCTTGA

>GAHD01000007 Echiopsis curta ACN-Ech-1 mRNA sequence
ATGCCCTCCTGCCAGCCTGGCAAGATGCCTGCGCCGTGGCCTTGGTGGCTCCAGCTTGTCCTTTGCATCC
TGTCCTCCATAGCAGTCTTGCCTGGGCGGGCAAGCGAGCTGAAAGTGTCCACGCAGACGGGCTGGGTGCG
TGGCCTGAGCCTGCCGGTTCTTGACGGCCACGTCTCGGCCTTCCTGGGCATCCCCTTTGCGGAGCCTCCC
GTGGGCAGGCTGCGCTTCCTGCGCCCTGAGCCTGTCAAGCCCTGGCAGCACGTCCTGGATGCTACTTCCT
ACCAGCGCGCCTGTTACCAGATGGTGGACAACAGCTACCCAGGTTTCCAGGGCACGGAGATGTGGAACCC
CAACCGAGGCATGAGCGAGGACTGCCTCTACCTCAACATCTGGGTGCCCTCCCCGCGTCCCAGAGACGTC
CCCGTCCTGGTCTGGATCTACGGAGGGGGATTCTACAGCGGCGCTGCCTCCCTGGACGTCTACGATGGCC
GTTTCCTGACCTACACACAGAACGTCATCTTGGTCTCCCTCAGCTACCGCGTGGGGGCCTTCGGCTTTTT
GGGCCTCCCGGGCAGTCCGGAGGCACCAGGGAACATGGGGCTGCTGGACCAGCGCTTGGCCCTGCAATGG
ATCCAGAACAACATCCATCATTTTGGAGGCAATCCCAGCGCGGTCACCATCKTTTGGGGAGAGTGCCGGG
GCCGCCTCGGTGGGCATGCACCTGCTTTCCACCCAGAGCCGCGCCCTCTTCCAGCGGGCCATTCTGCAGA
GCGGCGGCCCCAACACACCGTGGGCCACCGTCACACCTGCCGAAAGCCGGCGCCGGGCGGCTCTTCTGGG
AAAGCAGCTGGGCTGCCAGTTCAACAACGATTCCGAGCTGGTCAGTTGCTTGCGCTTGAAACCCCC

>GAHD01000008 Echiopsis curta CRiSP-Ech-1 mRNA sequence
ATGATTGCCTTCATTGTCTTGCTAAGTCTTGCTGCAGTGCTGCAACAGTCTTCTGGCACTGTTGATTTTG
CTTCTGAGTCAAGTAACAGAAAAGATTACCGAAAGGAGATTGTTGACAAGCACAATGCTTTAAGGAGATC
AGTGAAACCAACTGCTAGGAACATGTTACAAATGAAATGGAATTCTCGTGCTGCTCAAAATGCAAAACGT
TGGGCAGATAGATGTACTTTTGCTCACAGTCCATCACATACAAGAACTGTGGGAAAACTCCGTTGTGGTG
AAAATATATTCATGTCAAGTCAACCTTTTGCATGGAGTGGAGTAGTTCAGGCTTGGTATGATGAAGTCAA
AAAATTCGTCTATGGCATTGGAGCAAAGCCACGAGGTTCTGTTATTGGCCATTATACCCAGGTAGTTTGG
TACAAAAGTCACCTTCTTGGTTGTGCTTCTGCCAAATGTTCTTCAACCAAATACCTCTACGTTTGTCAAT
ACTGCCCAGCAGGGAACATCAGAGGTTCAATTGCTACTCCATATAAATCAGGGCCACCTTGTGGGGACTG
TCCTTCGGCTTGTGTCAACGGACTATGCACAAATCCTTGCAAATATGAAGATGATTTCTCGAACTGCAAA
GCTTTAGCGAAAAACTCTAAATGCCAGACTGCATGGATCAAGTCAAAATGCCCTTCTGCTTGCTTCTGCC
ACAATAAAATAATATAG

>GAHD01000009 Echiopsis curta KP-Ech-8 mRNA sequence
ATGATTGCCTTCATTGTCTTGCTAAGTCTTGCTGCAGTGCTGCAACAGTCTTCTGGCACTGTTGATTTTG
CTTCTGAGTCAAGTAACAGAAAAGATTACCGAAAGGAGATTGTTGACAAGCACAATGCTTTAAGGAGATC
AGTGAAACCAACTGCTAGGAACATGTTACAAATGAAATGGAATTCTCGTGCTGCTCAAAATGCAAAACGT
TGGGCAGATAGATGTACTTTTGCTCACAGTCCATCACATACAAGAACTGTGGGAAAACTCCGTTGTGGTG
AAAATATATTCATGTCAAGTCAACCTTTTGCATGGAGTGGAGTAGTTCAGGCTTGGTATGATGAAGTCAA

AAAATTCGTCTATGGCATTGGAGCAAAGCCACGAGGTTCTGTTATTGGCCATTATACCCAGGTAGTTTGG
TACAAAAGTCACCTTCTTGGTTGTGCTTCTGCCAAATGTTCTTCAACCAAATACCTCTACGTTTGTCAAT
ACTGCCCAGCAGGGAACATCAGAGGTTCAATTGCTACTCCATATAAATCAGGGCCACCTTGTGGGGACTG
TCCTTCGGCTTGTGTCAACGGACTATGCACAAATCCTTGCAAATATGAAGATGATTTCTCGAACTGCAAA
GCTTTAGCGAAAAACTCTAAATGCCAGACTGCATGGATCAAGTCAAAATGCCCTTCTGCTTGCTTCTGCC
ACAATAAAATAATATAG

>GAHD01000010 Echiopsis curta KP-Ech-3 mRNA sequence
ATGTGCCAGGAATTCATTTTTGGAGGTTGCAAAGGCAATGCCAACAACTTCGTCTCCAAACAGGACTGCT
TCCAGACGTGTATCAGAGGAGGGGCTGCAGAGGCAACGGTGGTACCTAGCGGGCCAGCCACTGAAGTGGC
TACACTAAGAGCTGGACATCTCCCAGAGGCCTACGAAAACAGGCCTGGCTTCAGAGAATTCTGTGCTGCG
CCCCGGGTGGTAGGTCCCTGCCGGGCCTCCTTCCTCCGTTGGTACTTCGACCTAGAGAGCCGGACGTGCA
AGATGTTCGTCTATGGGGGCTGTCGTGGCAACAAGAACAACTATCTCTTCGAAGAGCACTGCTGGAGCCA
GTGCACAGGTGATGGAGAGATAACTGAAGAGCCAGGAGATGCAGGCGCCCAGCCTCCCCTCCCCTCCGAA
CCCTTCAGCTTCTCCACCAGAGCTGTGGTCCTTGCTGTCCTTCTGGCCATCCTGGTGACTATCCTGCTGG
GCTCGATGGGGGTTTTCTTGTTAAGATCTGCCGGAAGAACCCGNGTTGTCAGTGGGAACAGTGTGGAGCA
CGCTGGATGA

>GAHD01000011 Echiopsis curta KP-Ech-4 mRNA sequence
ATGTCTTCTGGAGGTCTTCTTCTCCTGCTGGGACTCCTCACCCTCTGGGAGGTGCTGACCCCCGTCTCCA
GCAAGGACCGTCCACACTTCTGTCATCTGCCTTATGATACCGGACCATGTAAAGCCAGGTTCATAGCCTT
CTACTACAACTCAGTTCAACGTAAATGCCTAGAGTTTGTTTATGGTGGATGCGAAGGGAATGCTAACAAT
TTTAAGACCATAGATGCATGCAAACGCACCTGTGCTGCATGA

>GAHD01000012 Echiopsis curta LAO-Ech-1 mRNA sequence
CCCCTAGAAGAATGCTTCCAAGAAGCTGATTATGAAGAATTTCTAGAGATCGCCAGGAATGGTTTGAACA
AAACATCAAACCCAAAACATGTTGTGGTTGTAGGTGCAGGAATGGCTGGGCTTAGTGCAGCCTATGTTCT
TGCAGGAGCTAGACATAAGGTGACGCTTCTTGAAGCTAGTGAACGTGTGGGAGGACGAGTGAACACTTAT
CGAAATGAAACGGAAGGCTGGTATGTCAATCTGGGACCCATGCGTCTCCCTGAGAGACACAGGATTATCC
GGGAATATATCAGAAAATTTGGTCTCAAGTTGAATGAATTTCTTCAGGAAAATGAGAATGCCTGGTATTT
TATTAAAAACATCAGGAAGAGAGTATGGGAAGTCAAGAAAGACCCCGGCGTCTTCAAATATCCTTTGAAG
CCCTCAGAAGAAGGCAAATCTGCTTCACAGCTATATCGAGAATCCCTCGAAAAGGTTATGGAAGAATTGA
AAAGGACTAGCTGCAGCTATATACTAAATAAATATGACACCTACTCAACAAAGGAGTATCTAATTAAAGA
AGGAAATCTGAGCCGCGGAGCTGTAGATATGATTGGAAAGTTACTGAATGAAGATTCTAGCTATTACCTG
TCTTTTATTGAGAGCCTGAAAAGTGATGATCTCTTCTCTTATGAAAAAAGATTTGATGAAATTGTTGGTG
GATTTGATCAATTGCCTATATCCATGTATCAAGCCATTGCGAAAATGGTGCATTTAAATGCTCAAGTAAT
CAAGATACAACACAATGCTGAGGAAGTCAGAGTGACATATCAAACTCCAGAAAATACCTTGTCATATGTG
ACAGCCGATTATGTCATTGTATGCTCCACATCAAGGGCCACCCGTCGCATCTATTTTGAACCTCCCCTTC
CACCAAAGAAAGCACACGCTTTGCGGTCTATCCACTACAGAAGTGGCACCAAGATCTTCCTCACTTGCAC
TAGGAAGTTTTGGGAGGCTGATGGCATTCATGGTGGGAAGTCCACAACTGATCTTCCATCTCGATTCATC
TACTACCCTAACCATAACTTTACTAGTGGCGTTGGAGTTATTGTGGCCTATACCCTTGCTGATGATGCCG
ATTTCTTTCAAGCTCTTGATATCAAGACCAGTGCTGATATTGTCATTAATGACCTTTCACTCATCCATCA
GCTGCCCAAGGAAGAGATCCAGGCCCTCTGCTACCCCTCAATGATTAAAAAATGGAGCCTGGATAAATAT
GCTATGGGTGCTATAACCTCTTTCACTCCTTACCAGTTTCAAGATTTTATTGAAACAGTCGCTGCACCTG
TAGGCAGAATCTACTTTGCAGGGGAGTACACTGCCAGAGTTCATGGTTGGTTGGACAGCACAATTAAGTC
AGGGCTGACAGCAGCAAGAGATGTGAATCGTGCTTCTCGGAAGCCATCAAGAATCCAGCTGATCAGTGAC
AATCAACTTCAAGAAGGAGGTCAACACAAGGAGAATCCTAAACAGCCACTTTCCCCATTCTGGCAACTTC
TAGGTTTTTCAGACTAA

>GAHD01000013 Echiopsis curta LP-Ech-1 mRNA sequence
ATGGCGCAATTCATCGTCGTGAGCTTCACCTTGCTGGTCGTGGCCCTCTCCTTAAGGGGAACTGGAGCTG
ATCACCATTGTCCTAGCGGTTGGGCCTCCTATAATGAGTTTTGCTACATGGTCCACAACAGATCCGTCAC
CTGGAACAATGCCGAGAGATTCTGCCTGCAGCAAGAGACAAACTGCCACCTCGCCTCCATCCATAGCAAG
CCAGAGGCAGCCTTCATCGTCAGGCTGGTCTACCACAGGGCCAGCGCCGGGTCCAGTGTCTGGATCGGAT
TGAACGATCCAGAGAAAAGACGCACCTGGCAATGGAGTGATGGCTCTAGCATAAAGTACAGATCCTGGAT
GCCAGGAGAGCCCAACAACCATGCAAACAACGAGTATTGTGTTGTGCTGTCAGCCTGGTCACGTTATGTC
GGATGGAACGATCAGGACTGTGGGTCCAGGCATAACTTTGTCTGCAAGTTCCAGCCAAGGACCGAGACGA
ACACCGTCAATGTTGCAAACAGGAAGTAA

>GAHD01000014 Echiopsis curta NP-Ech-33 mRNA sequence
ATGCTCGGCCTCTCACGTCTGGCGGGCGGCGGGCTGCTATTAGTGGTGCTGGCCCTGCTGCCTCTCGCCC
TCGACGGGAAGCCGACGGCGCCGCCTCAGGCGCTGCCCACGGCTCCGGCGGGCGGCATGATGGCGTCGCG
GCCGATGCTGACGGAGAAGCAGAGGCAGCAGCACCAGCAGCCGCTGCCGCCGACGATGGAGGAGTCCTCG
GGTCCCGCGGCTGAGCGCAGCGGCTCCAAGACGGCCAAGATAGGGAACGGCTGCTTCAGCTTCCCGCTCG
ACCGCATCGGCAGCGTGAGCGGCATGGGCTGCAGAACCTTCCCGAAGCCGACACCTGGCGGGTCCTAA

>GAHD01000015 Echiopsis curta NP-Ech-34 mRNA sequence
ATGCTCGGCCTCTCACGTCTGGCGGGCGGCGGGCTGCTATTAGTGGTGCTGGCCCTGCTGCCTCTCGCCC
TCGACGGGAAGCCGACGGCGCCGCCTCAGGCGCTGCCCACGGCTCCGGCGGGCGGCATGATGGCGTCGCG
GCCGATGCTGACGGAGAAGCAGAGGCAGCAGCACCAGCAGCCGCTGCCGCCGACGATGGAGGAGTCCTCG
GGTCCCGCGGCTGAGCGCAGCGGCTCCAAGACGGCCAAGATAGGGAACGGCTGCTTCAGCTTCCCGCTCG
ACCGCATCGGCAGCGTGAGCGGCATGGGCTGCAGAACCTTCCCGAAGCCGACACCTGGCGGGTCCTAA

>GAHD01000016 Echiopsis curta NP-Ech-35 mRNA sequence
ATGCTCGGCCTCTCACGTCTGGCGGGCGGCGGGCTGCTATTAGTGGTGCTGGCCCTGCTGCCTCTCGCCC
TCGACGGGAAGCCGACGGCGCCGCCTCAGGCGCTGCCCACGGCTCCGGCGGGCGGCATGATGGCGTCGCG
GCCGATGCTGACGGAGAAGCAGAGGCAGCAGCACCAGCAGCCGCTGCCGCCGACGATGGAGGAGTCCTCG
GGTCCCGCGGCTGAGCGCAGCGGCTCCAAGACGGCCAAGATAGGGAACGGCTGCTTCGGCTTCCCGCTCG
ACCGCATCGGCAGCGTGAGCGGCATGGGCTGCAGAACCTTCCCGAAGCCGACACCTGGCGGGTCCTAA

>GAHD01000017 Echiopsis curta NP-Ech-36 mRNA sequence
ATGCTCGGCCTCTCACGTCTGGCGGGCGGCGGGCTGCTATTGGTGGTGCTGGCCCTGCTGCCTCTCGCCC
TCGACGGGAAGCCGACGGCGCCGCCTCAGGCGCTGCCCACGGCTCCGGCGGGCGGCATGATGGCGTCGCG
GCCGATGCTGACGGAGCAGCAGAGGCAGCAGCACCAGCACCAGCAGCCGCCGACGATGGAGGAGTCCTCG
GGTCCCGCGGCTGAGCGCAGCGGCTCCAAGACGGCCAAGATCGGGGACGGCTGCTTCGGCCTCCCGCTCG
ACCGCATCGGCAGCACGAGCGGCATGGGCTGCGGAAGCGTCCCGAAGCCGACACCTGGCGGGTCCTAA

>GAHD01000018 Echiopsis curta NP-Ech-37 mRNA sequence
ATGCTCGGCCTCTCACGTCTGGCGGGCGGCGGGCTGCTATTGGTGGTGCTGGCCCTGCTGCCTCTCGCCC
TCGACGGGAAGCCGACGGCGCCGCCTCAGGCGCTGCCCACGGCTCCGGCGGGCGGCATGATGGCGTCGCG
GCCGATGCTGACGGAGAAGCAGAGGCAGCAGCATCAGCACCAGCAGCCGCCGACGATGGAGGAGTCCTCG

GGTCCCGCGGCTGAGCGCAGCGGCTCCAAGACGGCCAAGATCGGGGACGGCTGCTTCGGCCTCCCGCTCG
ACCGCATCGGCAGCACGAGCGGCATGGGCTGCGGAAGCGTCCCGAAGCCGACACCTGGCGGGTCCTAA

>GAHD01000019 Echiopsis curta NP-Ech-38 mRNA sequence
ATGCTCGGCCTCTCACGTCTGGCGGGCGGCGGGCTGCTATTGGTGGTGCTGGCCCTGCTGCCTCTCGCCC
TCGACGGGAAGCCGACGGCGCCGCCTCAGGCGCTGCCCACGGCTCCGGCGGGCGGCATGATGGCGTCGCG
GCCGATGCTGACGGAGCAGCAGAGGCAGCAGCACCAGCACCAGCAGCCGCCGACGATGGAGGAGTCCTCG
GGTCCCGCGGCTGAGCGCAGCGGCTCCAAGACGGCCAAGATCGCGGACGGCTGCTTCGGCCTCCCGCTCG
ACCGCATCGGCAGCACGAGCGGCATGGGCTGCGGAAGCGTCCCGAAGCCGACACCTGGCGGGTCCTAA

>GAHD01000020 Echiopsis curta NP-Ech-39 mRNA sequence
ATGCTCGGCCTCTCACGTCTGGCGGGCGGCGGGCTGCTATTGGTGGTGCTGGCCCTGCTGCCTCTCGCCC
TCGACGGGAAGCCGACGGCGCCGCCTCAGGCGCTGCCCACGGCTCCGGCGGGCGGCATGATGGCGTCGCG
GCCGATGCTGACGGAGAAGCAGAGGCAGCAGCATCAGCACCAGCAGCCGCCGACGATGGAGGAGTCCTCG
GGTCCCGCGGCTGAGCGCAGCGGCTCCAAGACGGCCAAGATCGGGGACGGCTGCTTCGGCCTCCCGCTCG
ACCGCATCGGCAGCACGAGCGGCATGGGCTGCGGAAGCGTCCCGAAGCCGACACCTGGCGGGTCCTAA

>GAHD01000021 Echiopsis curta NP-Ech-46 mRNA sequence
ATGCTCGGCCTCTCACGTCTGGCGGGCGGCGGGCTGCTATTGGTGGTGCTGGCCCTGCTGCCTCTCGCCC
TCGACGGGAAGCCGACGGCGCCGCCTCAGGCGCTGCCCACGGCTCCGGCGGGCGGCATGATGGCGTCGCG
GCCGATGCTGACGGAGCAGCAGAGGCAGCAGCACCAGCACCAGCAGCCGCCGACGATGGAGGAGTCCTCG
GGTCCCGCGGCTGAGCGCAGCGGCTCCAAGACGGCCAAGATCGGGGACGGCTGCTTCGGCCTCCCGCTCG
ACCGCATCGGCAGCACGAGCGGCATGGGCTGCGGAAGCGTCCCGAAGCCGACACCTGGCGGGTCCTAA

>GAHD01000022 Echiopsis curta NP-Ech-47 mRNA sequence
ATGCTCGGCCTCTCACGTCTGGCGGGCGGCGGGCTGCTATTGGTGGTGCTGGCCCTGCTGCCTCTCGCCC
TCGACGGGAAGCCGACGGCGCCGCCTCAGGCGCTGCCCACGGCTCCGGCGGGCGGCATGATGGCGTCGCG
GCCGATGCTGACGGAGCAGCAGAGGCAGCAGCACCAGCACCAGCAGCCGCCGACGATGGAGGAGTCCTCG
GGTCCCGCGGCTGAGCGCAGCGGCTCCAAGACGGCCAAGATCGGGGACGGCTGCTTCGGCCTCCCGCTCG
ACCGCATCGGCAGCACGAGCGGCATGGGCTGCGGAAGCGTCCCGAAGCCGACACCTGGCGGGTCCTAA

>GAHD01000023 Echiopsis curta NP-Ech-48 mRNA sequence
ATGCTCGGCCTCTCACGTCTGGCGGGCGGCGGGCTGCTATTGGTGGTGCTGGCCCTGCTGCCTCTCGCCC
TCGACGGGAAGCCGACGGCGCCGCCTCAGGCGCTGCCCACGGCTCCGGCGGGCGGCATGATGGCGTCGCG
GCCGATGCTGACGGAGCAGCAGAGGCAGCAGCACCAGCACCAGCAGCCGCCGACGATGGAGGAGTCCTCG
GGTCCCGCGGCTGAGCGCAGCGGCTCCAAGACGGCCAAGATCGGGGACGGCTGCTTCGGCCTCCCGCTCG
ACCGCATCGGCAGCACGAGCGGCATGGGCTGCGGAAGCGTCCCGAAGCCGACACCTGGCGGGTCCTAA

>GAHD01000024 Echiopsis curta NP-Ech-50 mRNA sequence
ATGCTCGGCCTCTCACGTCTGGCGGGCGGCGGGCTGCTATTGGTGGTGCTGGCCCTGCTGCCTCTCGCCC
TCGACGGGAAGCCGACGGCGCCGCCTCAGGCGCTGCCCACGGCTCCGGCGGGCGGCATGATGGCGTCGCG
GCCGATGCTGACGGAGAAGCAGAGGCAGCAGCACCAGCAGCCGCCGACGATGGAGGAGTCCTCGGGTCCC
GCGGCTGAGCGCAGCGGCTCCAAGACGGCCAAGATCGGGGACGGCTGCTTCGGCCTCCCGCTCGACCGCA
TCGGCAGCACGAGCGGCATGGGCTGCGGAAGCGTCCCGAAGCCGACACCTGGCGGGTCCTAA

>GAHD01000025 Echiopsis curta NP-Ech-51 mRNA sequence
ATGCTCGGCCTCTCACGTCTGGCGGGCGGCGGGCTGCTATTGGTGGTGCTGGCCCTGCTGCCTCTCGCCC
TCGACGGGAAGCCGACGGCGCCGCCTCAGGCGCTGCCCACGGCTCCGGCGGGCGGCATGATGGCGTCGCG
GCCGATGCTGACGGAGCAGCAGAGGCAGCAGCACCAGCACCAGCAGCCGCCGACGATGGAGGAGTCCTCG
GGTCCCGCGGCTGAGCGCAGCGGCTCCAAGACGGCCAAGATCGGGGACGGCTGCTTCGGCCTCCCGCTCG
ACCGCATCGGCAGCACGAGCGGCATGGGCTGCGGAAGCGTCCCGAAGCCGACACCTGGCGGGTCCTAA

>GAHD01000026 Echiopsis curta NP-Ech-52 mRNA sequence
ATGCTCGGCCTCTCACGTCTGGCGGGCGGCGGGCTGCTATTAGTGGTGCTGGCCCTGCTGCCTCTCGCCC
TCGACGGGAAGCCGACGGCGCCGCCTCAGGCGCTGCCCACGGCTCCGGCGGGCGGCATGATGGCGTCGCG
GCCGATGCTGACGGAGAAGCAGAGGCAGCAGCACCAGCAGCCGCTGCCGCCGACGATGGAGGAGTCCTCG
GGTCCCGCGGCTGAGCGCAGCGGCTCCAAGACGGCCAAGATAGGGAACGGCTGCTTCGGCTTCCCGCTCG
ACCGCATCGGCAGCGTGAGCGGCATGGGCTGCAGAACCTTCCCGAAGCCGACACCTGGCGGGTCCTAA

>GAHD01000027 Echiopsis curta NP-Ech-56 mRNA sequence
ATGCTCGGCCTCTCACGTCTGGCGGGCGGCGGGCTGCTATTGGTGGTGCTGGCCCTGCTGCCTCTCGCCC
TCGACGGGAAGCCGACGGCGCCGCCTCAGGCGCTGCCCACGGCTCCGGCGGGCGGCATGATGGCGTCGCG
GCCGATGCTGACGGAGCAGCAGAGGCAGCAGCACCAGCACCAGCAGCCGCCGACGATGGAGGAGTCCTCG
GGTCCCGCGGCTGAGCGCAGCGGCTCCAAGACGGCCAAGATCGGGGACGGCTGCTTCGGCCTCCCGCTCG
ACCGCATCGGCAGCACGAGCGGCATGGGCTGCGGAAGCGTCCCGAAGCCGACACCTGGCGGGTCCTAA

>GAHD01000028 Echiopsis curta NP-Ech-61 mRNA sequence
ATGCTCGGCCTCTCACGTCTGGCGGGCGGCGGGCTGCTATTGGTGGTGCTGGCCCTGCTGCCTCTCGCCC
TCGACGGGAAGCCGACGGCGCCGCCTCAGGCGCTGCCCACGGCTCCGGCGGGCGGCATGATGGCGTCGCG
GCCGATGCTGACGGAGCAGCAGAGGCAGCAGCACCAGCACCAGCAGCCGCCGACGATGGAGGAGTCCTCG
GGTCCCGCGGCTGAGCGCAGCGGCTCCAAGACGGCCAAGATCGGGGACGGCTGCTTCGGCCTCCCGCTCG
ACCGCATCGGCAGCACGAGCGGCATGGGCTGCGGAAGCGTCCCGAAGCCGACACCTGGCGGGTCCTAA

>GAHD01000029 Echiopsis curta NP-Ech-64 mRNA sequence
ATGCTCGGCCTCTCACGTCTGGCGGGCGGCGGGCTGCTATTGGTGGTGCTGGCCCTGCTGCCTCTCGCCC
TCGACGGGAAGCCGACGGCGCCGCCTCAGGCGCTGCCCACGGCTCCGGCGGGCGGCATGATGGCGTCGCG
GCCGATGCTGACGGAGCAGCAGAGGCAGCAGCACCAGCACCAGCAGCCGCCGACGATGGAGGAGTCCTGG
GTCCCCGCGGCTGAGCGCAGCGGCTCCAAGACGGCCAAGATCGGGGACGGCTGCTTCGGCCTCCCGCTCG
ACCGCATCGGCAGCACGAGCGGCATGGGCTGCGGAAGCGTCCCGAAGCCGACACCTGGCGGGTCCTAA

>GAHD01000030 Echiopsis curta NP-Ech-70 mRNA sequence
ATGCTCGGCCTCTCACGTCTGGCGGGCGGCGGGCTGCTATTGGTGGTGCTGGCCCTGCTGCCTCTCGCCC
TCGACGGGAAGCCGACGGCGCCGCCTCAGGCGCTGCCCACGGCTCCGGCGGGCGGCATGATGGCGTCGCG
GCCGATGCTGACGGAGCAGCAGAGGCAGCAGCACCAGCACCAGCAGCCGCCGACGATGGAGGAGTCCTCG
GGTCCCGCGGCTGAGCGCAGCGGCTCCAAGACGGCCAAGATCGGGGACGGCTGCTTCGGCCTCCCGCTCG
ACCGCATCGGCAGCACGAGCGGCATGGGCTGCGGAAGCGTCCCGAAGCCGACACCTGGCGGGTCCTAA

>GAHD01000031 Echiopsis curta NGF-Ech-1 mRNA sequence
ATGATGCATAGCGTAATGTCCATGCTGTGCTACACTCTGATTATTGCATTTCTGATTGGCATATCGGCAG
CACCAAAATCTGAAGATAATGTCCCTCTGGGCTCCCCTGCAACCAGCTTTGCTCAAACTCATGAGGGTCT
GAAAACATCTCGAAATACAGATCAGCACCATCCTGCTCCTAAGAAGGCAGAGGATCAAGAACTTGGATCA

GTAGCAAACATCATTGTGGATCCCAAGCTTTTTCAGAAGAGGCATTTCCAGTCACTTCGTGTTCTGTTCA
GCACTCAGCCCCCACCATTGTCAAGAGATGAGCAAAGTGTGGAGTTCCTGGACAATGAAGATGCTCTTAA
CAGGAATATCCGGACCAAACGTGAAAATCATCCTGTGCATAACCTAGGGGAACATTCTGTGTGTGACAGT
ATCAGTGTCTGGGTTAGCAACAAAACCAACGCAACGGACATCAAAGGAAATATGGTGACTGTGATGGTAG
ACATAAATCTTAATAATGAAGTCTACAAGCAGTACTTTTTTGAAACCAAGTGCAGAAATCCAAATCCAGT
ACCGAGTGGGTGCAGGGGCACTGATTCCAGGCATTGGAATTCTTATTGCACCACAACACAGACATTTGTC
AGGGCATTAACCATGGAAGGCAATCGGGCATCCTGGCGCTTCATTCGGATTGACACTGCCTGTGTGTGCG
TAATCAGTAGAAAAACTGTCAACTTCTGA

>GAHD01000032 Echiopsis curta SVMP-Ech-32 mRNA sequence
ATGATCCAAGCTCTCTTGGTAACTATATGCTTCACGGTTTTTCCATATCAAGGGAGCTCTATCATCCTGG
AATCCGGGAATGTTAATGATTATGAAGTAGTGTATCCACAAAAAGTGCCTGCGTTGCCCAAAGGAGGAGT
TCAGAATCCTCAGCCAGAGACCAAGTATGAAGATACAATGCAATATGAATTTCAAGTAAATGGAGAGCCA
GTGGTCCTTCACTTAGAAAGAAATAAAGGACTTTTTTCAGAAGATTACACTGAAACTCATTATGCCCCTG
ATGGCAGAGAAATTACAACAAGCCCTCCGGTTCAGGATCACTGCTATTATCATGGCTACATTCAGAATGA
AGCTGATTCAAGTGCAGTCATCAGTGCATGCGACGGCTTGAAAGGACATTTCAAGCATCGAGGGGAGACA
TACTTTATTGAGCCCTTGAAGCTTTCCGACAGTGAATCCCATGCAATCTACAAAGATGAAAATGTAGAAA
AAGAGGATGAGATCCCCAAAATCTGTGGGGTAACCCATACTACTTCGGAGTCAGATGAGCCCATCGAAAA
GATCTCTCAATTAACTTATACTCCTGAACAAAACAGGTACTTGCAGATCAAAAAATACATTGAGTTCTAT
GTAGTTGTGGACAACAGATTGTACAAGAATTACAGCAGCAACAGAGATGCTATAAAAACAGGAGTATATG
AAATGGTCAACATTTTAAATGTGATGTACAGACCTTTGAATGTTCTCATTGCATTGATTGGCCTGGAAAT
TTGGACCAACCAAGATGAGATTAAGATTGAACCAGATGTGGAGGTCACTTTGGAATCATTTGGAGAATGG
AGAGAAACAGTTTTACTGCTACGCAAAAGGAATGATAATGCTCAGTTACTTACGGGCATTGACTTCAACG
GAGATACTGTAGGACTTGCTTACTTGGGCACCCTCTGCAATCCGGAGGAATCTGTAGCAGTTATTCAGGA
ACATAGCAGCAGAACAAGCATAGTGGCATCTACAATGGCCCATGAGTTGGGTCATAATCTGGGCATTAAT
CATGACAGCGCTTCCTGTAATTGCAATGCTGGACCATGCATTATGTCTGCGGCAATAAATGATGAACCTT
TCTATGAGTTCAGCAATTGTAGTGTCCAGGAACATCAAATTTATCTTCTTAACTTCAGACCACAATGCAT
TCTCAACAAACCCTTGAGCACAGATATTGTTACACCTCCAGTTTGTGGAAATTACTTAGTGGAGGTGGGA
GAAGAATGTGACTGTGGCTCTCCTCAGGATTGTCAAGATGCCTGCTGCAACGCTGCAACTTGTAAACTGC
AACATGACTGTGACTCCGGAGAGTGTTGTGAGCAATGCAAATTTAAGAAAGCAGGAGCAGAATGCCGGGC
AGCAAAGGATGACTGTGACTTGCCTGAAAGCTGCACTGGCCAATCTGCTGAGTGTCCCACGGACAGCTTC
CAGAGGAATGGACATCCATGCCAAAACAACCAGGGTTACTGCTACAATGGGACATGCCCCCTCATGACAA
ACCAGTGTATTGCTCTAATGGGGCCAGGTGTAACTGTGTCTCCAGATGAATGTTTTACGTTGAAACAGAA
TGACCCAAATTGTGGCTTCTGCAGAATAGAAAATGGTAGAAAGATTCCATGTGCAGAAAAGGATAAAATG
TGTGGCATGTTAGTATGCGAAAAGAGAAACTCGAAATGCACCTGCTTTACTACAACAGATGACCCAGATT
ATGGAATGGTTGATCCTGGAACAAAATGTGAAGATGGAAAGGTGTGCATCAACAGGCAGTGTGTTGATGT
GCAGACAGCCTACTGA

>GAHE00000000.1|GAHE01000000 TSA: Furina ornata, transcriptome shotgun assembly, transcriptome shotgun assembly

>GAHE01000001 Furina ornata 3FTx-Fur-10 mRNA sequence
ATGAAAACTCTGCTGCTGACCTTGGTGGTGGTGACAATCGTGTGCCTGGACTTGGGTGACAGCGTGATAT
GCTACTTTGGTTATAATTACTTTCGGACTTGTCCAGATGGGCACCAATGCTTTACAAAGACTTGGTGTGA
TGCTTGGTGTAGCGTCAGAGGACCACGGGTCGAAATGGGATGTGAAGCTACTTGCCCTATAGTGAAGCCC
GGTATAAAGATGTTCTGTTGCTCAACAGACAAATGCAACCCATTTCCGAAATGGGAACGTCCTACACGAA
GTCCTTGA

>GAHE01000002 Furina ornata 3FTx-Fur-11 mRNA sequence
ATGAAAACTCTGCTGCTGACCTTGGTGGTGGTGACAATCGTGTGCCTGGACTTGGGTGACAGCGTGATAT
GCTACTTTGGTTATAATTACTCTCGGACTTGTCCAGATGGGCACCAATGCTTTACAAAGACTTGGTGTGA
TGCTTGGTGTGCTGAAAGAGGACCGCGAGTCGAAATGGGATGTGAAGCTACTTGCCCTACAGTGAAGCCC
GGTGTAAAGATTTTCTGTTGCTCAACAGACAAGTGCAACCCATTTCCGAAAGTTGAACGTCCTGGATGA

>GAHE01000003 Furina ornata 3FTx-Fur-12 mRNA sequence
ATGAAAACTCTGCTGCTGACCTTGGTGGTGGTGACAATCGTGTGCCTGGACTTGGGTGACAGCGTGATAT
GCTACTTTGGTGAAAATTACTCTCGGACTTGTCCAGATGGGCACCAATGCTTTACAAGGACTTGGTGTGA
TCCTTGGTGTAGCGTCAGAGGACCACGGGTCGAAATGGGATGTGAAGCTACTTGCCCTACAGTGAAGAAG
GGTATAAAGATATTCTGTTGCTCAACAGACAAGTGCAACCCATTTCCGAAATGGGAACGTCCTACACGAA
GTCCTTGA

>GAHE01000004 Furina ornata 3FTx-Fur-13 mRNA sequence
ATGAAAACTCTGCTGCTGACCTTGGTGGTGGTGACAATCGTGTGCCTGGACTTGGGTGACAGCTTGATAT
GCTACTTTGGTGAAAATTACTCTCGGACTTGTCCAGATGGGCACAAATGCTTTACAAGGACTTGGTGTGA
TCCTTGGTGTTCCATCAGAGGACCGCGGGTCGAAATGGGATGTGAAGCTACTTGCCCTATAGTGAAGCCC
GGTATAAAGATATTCTGTTGCTCAACAGACAAATGCAACCCATTTCCGAAATGGGAACGTCCTACACGAC
GTCCTTGA

>GAHE01000005 Furina ornata 3FTx-Fur-14 mRNA sequence
ATGAAAACTCTGCTGCTGACCTTGGTGGTGGTGACAATCGTGTGCCTGGACTTGGGTGACAGCGTGATAT
GCTACTTTGGTGAAAATTACTCTCGGACTTGTCCAGATGGGCACCAATGCTTTACAAGGACTTGGTGTGA
TCCTTGGTGTAGCGTCAGAGGACCACGGGTCGAAATGGGATGTGAAGCTACTTGCCCTACAGTGAAGAAG
GGTATAAAGATATTCTGTTGCTCAACAGACAAGTGCAACCCATTTCCGAAATGGGAACGTCCTACACGAA
GTACTTGA

>GAHE01000006 Furina ornata 3FTx-Fur-16 mRNA sequence
ATGAAAACTCTGCTGCTGACCTTGGTGGTGGTGACAATCGTGTGCCTGGACTTGGGTGACAGCGTGATAT
GCTACTTTGGTGAAAATTACTCTCGGACTTGTCCAGATGGGCACCAATGCTTTACAAGGACTTGGTGTGA
TCCTTGGTGTAGCGTCAGAGGACCACGGGTCGAAATGGGATGTGAAGCTACTTGCCCTACAGTGAAGAAG
GGTATAAAGATATTCTGTTGCTCAACAGACAAGTGCAACCCATTTCCGAAATGGGAACGTCCTACACGAA
GCCCTTGA

>GAHE01000007 Furina ornata 3FTx-Fur-17 mRNA sequence
ATGAAAACTCTGCTGCTGACCTTGGCGGTGGTGACAATCGTGTGCCTGGACTTGGGTGACAGCGTGATAT
GCTACTTTGGTTATAATTACTCTCGGACTTGTCCAGATGGGCACCAATGCTTTACAAAGACTTGGTGTGA
TGCTTGGTGTGCTGAAAGAGGACCGCGAGTCGAAATGGGATGTGAAGCTACTTGCCCTACAGTGAAGCCC
GGTGTAAAGATTTTCTGTTGCTCAACAGACAAGTGCAACCCATTTCCGAAAGTTGAACGTCCTGGATGA

>GAHE01000008 Furina ornata 3FTx-Fur-23 mRNA sequence
ATGAAAACTCTGCTGCTGACCTTGGTGGTGGTGACAATCGTGTGCCTGGACTTGGGTGACAGCGTGATAT

GCTACTTTGGTTATAATTACTTTCGGACTTGTCCAGATGGGCACCAATGCTTTACAAAGACTTGGTGTGA
TGCTTGGTGTAGCGTCAGAGGACCACGGGTCGAAATGGATGTGAAGCTACTTGCCCTATAGTGAAGCCT
GGTATAAAGATGTTCTGTTGCTCAACAGACAAATGCAACCCATTTCCGAAATGGGAACGTCCTACACGAA
GTCCTTGA

>GAHE01000009 Furina ornata 3FTx-Fur-24 mRNA sequence
ATGAAAACTCTGCTGCTGACCTTGGTGGTGGTGACAATTGTGTGCCTGGACTTGGGTGACAGCGTGATAT
GCTACTTTGGTTATAATTACTCTCGGACTTGTCCAGATGGGCACCAATGCTTTACAAAGACTTGGTGTGA
TGCTTGGTGTGCTGAAAGAGGACCGCGAGTCGAAATGGGATGTGAAGCTACTTGCCCTACAGTGAAGCCC
GGTGTAAAGATTTTCTGTTGCTCAACAGACAAGTGCAACCCATTTCCGAAAGTGGAACGTCCTGGATGA

>GAHE01000010 Furina ornata 3FTx-Fur-26 mRNA sequence
ATGAAAACTCTGCTGCTGACCTTGGTGGTGGTGACAATCGTGTGCCTGGACTTGGGTGACAGCTTGATAT
GCTACTTTGGTTATAATTACTCTCAGACTTGTCCAGATGGGCACCAATGCTTTACAAAGACTTGGTGTGA
TGCTTGGTGTTCCGTCAGAGGACCGCGGGTCGAAATGGGATGTGAAGCTACTTGCCCTATAGTGAAGCCC
GGTATAAAGATATTCTGTTGCTCAACAGACAAATGCAACCCATTTCCGAAATGGGAACGTCCTACACGAC
GTCCTTGA

>GAHE01000011 Furina ornata 3FTx-Fur-27 mRNA sequence
ATGAAAACTCTGCTGCTGACCTTGGTGGTGGTGACAATCGTGTGCCTGGACTTGGGTGACAGCTTGATAT
GCTACTTTGGTTATAATTACTCTCAGACTTGTCCAGATGGGCACCAATGCTTTACAAAGACTTGGTGTGA
TGCTTGGTGTTCCATCAGAGGACCGCGGGTCGAAATGGGATGTGAAGCTACTTGCCCTATAGTGAAGCCC
GGTATAAAGATGTTCTGTTGCTCAACAGACAAATGCAACCCATTTCCGAAATGGGAACGTCCTACACGAC
GTCCTTGA

>GAHE01000012 Furina ornata 3FTx-Fur-28 mRNA sequence
ATGAAAACTCTGCTGCTGACCTTGGTGGTGGTGACAATCGTGTGCCTGGACTTGGGTGACAGCGTGATAT
GCTACTTTGGTGAAAATTATTCTCAGACTTGTCCAGATGGGCACCAATGCTTTACAAGGACTTGGTGTGA
TCCTTGGTGTAGCGTCAGAGGACCACGGGTCGAAATGGGATGTGAAGCTACTTGCCCTACAGTGAAGAAG
GGTATAAAGATATTCTGTTGCTCAACAGACAAGTGCAACCCATTTCCGAAATGGGAACGTCCTACACGAC
GTCCTTGA

>GAHE01000013 Furina ornata 3FTx-Fur-31 mRNA sequence
ATGAAAACTCTGCTGCTGACCTTGGTGGTGGTGACAATCGTGTGCCTGGACTTGGGTGACAGCTTGATAT
GCTACTTTGGTTATAATTACTCTCAGACTTGTCCAGATGGGCACCAATGCTTTACAAAGACTTGGTGTGA
TGCTTGGTGTAGCATCAGAGGACCGCGGGTCGAAATGGGATGTGAAGCTACTTGCCCTACAGTGAAGCCC
GGTATAAAGATATTCTGTTGCTCAACAGACAAATGCAACCCATTTCCGAAATGGGAACGTCCTACACGAC
ATCCTTGA

>GAHE01000014 Furina ornata 3FTx-Fur-33 mRNA sequence
ATGAAAACTCTGCTGCTGACCTTGGTGGTGGTGACAATCGTGTGCCTGGACTTGGGTGACAGCGTGATAT
GCTACTTTGGTTATAATTACTCTCGGACTTGTCCAGATGGGCACCAATGCTTTACAAAGACTTGGTGTGA
TGCTTGGTGTCCTGAAAGAGGACCGCGAGTCGAAATGGGATGTGAAGCTACTTGCCCTACAGTGAAGCCC
GGTGTAAAGATTTTCTGTTGCTCAACAGACAAGTGCAACCCATTTCCGAAATGGGAACGTCCTGGATGA

>GAHE01000015 Furina ornata 3FTx-Fur-44 mRNA sequence
ATGAAAACTCTGCTGCTGACCTTGGTGGTGGTGACAATCGTGTGCCTGGACTTGGGATACACCAGGACAT
GCTGGTTAACACCTGAAGTTAAACCTCAGACTTGTCCACCTGGGCAGGACCAATGCTTTACAAAGACTTG
GTGTGATGCTTGGTGTCCCATCAGAGGAGAGCGAGTTGAATTGGGATGTGCTGCTACTTGCCCTACAGTG
AAGCCTGGTGTAAAGATTGTCTGTTGCTCAACAGACAATTGCAACCCATTTCCGACAAAACGTCGTTGA

>GAHE01000016 Furina ornata 3FTx-Fur-46 mRNA sequence
ATGAAAACTCTGCTGCTGACCTTGGTGGTGGTGACAATCGTGTGCCTGGACTTGGGATACACCAGGACAT
GCTGGTTAACACCTGAAGTTAAACCTCAGACTTGTCCACCTGGGCAGGACCAATGCTTTAAAAAGACTTG
GTGTGATGCTTGGTGTCCCATCAGAGGAGAGCGAGTCGAATTGGGATGTGCTGCTACTTGCCCTACAGTG
AAGCCTGGTGTAAAGATTGTCTGTTGCTCAACAGACAATTGCAACCCATTTCCGACAAAACGTCGTTGA

>GAHE01000017 Furina ornata 3FTx-Fur-55 mRNA sequence
ATGAAAACTCTACTGCTGACCTTGGTAGTGGTGACAATCGTGTGCCTGGACTTGGGATACCCCCTAGCAT
GTCTCCACTCTCCGGGTTCTCCTAGTTATTCGGTTTGTCCAGGGTACATGACCATTTGCTATCGATATGA
TTTTTCACCAATTAAAGATAAAGTCGTAACCGTCAGGGGATGTAGTGCTTCTTGCCCTGGAGGAAGAAAT
CATGTGTGTTGCAGTACAGACATGTGCAACCGGTAG

>GAHE01000018 Furina ornata 3FTx-Fur-57 mRNA sequence
ATGAAAACTCTGCTGCTGACCTTGGTAGTGGTGACAATCGTGTGCCTGGACTTAGGATACCCCCTAGTAT
GTCTCCACTCTCCGGGTTCTCCTAGTTATTCGGTTTGTGCAGGGTACATGACCATTTGCTATCGATATGA
TTTTTCACCAATTAAAGATAAAGTCGTAACCGTTAGGGGATGTAGTGCTTCTTGCCCTGGAGGAAGAAAT
CATGTGTGTTGCAGTACAGACATGTGCAACGGGTAG

>GAHE01000019 Furina ornata 3FTx-Fur-58 mRNA sequence
ATGAAAACTCTGTTGCTGACCTTGGTAGTGGTGACAATTGTGTGCCTGGACTTAGGATACCCCCTAGTAT
GTCTCCACTCTCCGGGTTCTCCTAGTTATTCGGTTTGTGCAGGGTACATGACCATTTGCTATCGATATGA
TTTTTCACCAATTAAAGATAAAGTCGTAACCGTTAGGGGATGTAGTGCTTCTTGCCCTGGAGGAAGAAAT
CATGTGTGTTGCAGTACAGACATGTGCAACGAGTAG

>GAHE01000020 Furina ornata 3FTx-Fur-61 mRNA sequence
ATGAAAACTCTGCTGCTGACCTTGGTAGTGGTGACAATCGCATGCCTGGACTTAGGATACCCCCTAGTAT
GTCTCCACTCTCCGGGTTCTCCTAGTTATTCGGTTTGTCCAGGGTACATGACCATTTGCTATCGATATGA
TTTTTCACCAATTAAAGATAAAGTCGTAACCGTCAGGGGATGTAGTGCTTCTTGCCCTGGAGGAAGAAAT
CATGTGTGTTGCAGTACAGACATGTGCAACGGGTAG

>GAHE01000021 Furina ornata 3FTx-Fur-62 mRNA sequence
ATGAAAACTCTGCTGCTGACTTTGGTAGTGGTGACAATCGCATGCCTGGACTTAGGATACCCCCTAGTAT
GTCTCCACTCTCCGGGTTCTCCTAGTTATTCGGTTTGTGCAGGGTACATGACCATTTGCTATCGATATGA
TTTTTCACCAATTAAAGATAAAGTCGTAACCGTTAGGGGATGTAGTGCTTCTTGCCCTGGAGGAAGAAAT
CATGTGTGTTGCAGTACAGACATGTGCAACGAGTAG

>GAHE01000022 Furina ornata 3FTx-Fur-63 mRNA sequence
ATGAAAACTCTACTGCTGACCTTGGTAGTGGTGACAATCATGTGCCTGGACTTAGGATACACCCTGAGAT
GTCACAACACTTGGAATTATGGTTATATAAGTTGTGAAGCACACGCGCCCTTTTGCTATAAATCTCGTCA
TTATGGACGTACAGAGAAACCTACAATCGCCCAGGGTTGTACTGCTAAATGCTCTGAAAAAAGTGAAGTG

TGTTGCAGTACAGACATGTGCAACAAGTAG

>GAHE01000023 Furina ornata 3FTx-Fur-64 mRNA sequence
ATGAAAACTCTGCTGCTGACCTTGGTAGTGGTGACAATCGTGTGCCTGGACTTGGGATACACCCTAATAT
GTCACAACACTTGGAATTATGGTTATATAACTTGTGAAGAACACCAGACCTTTTGCTATAAAGCTTTTTA
TAATGGACGTACAGAGCGTCCTCTCATCGCCCGGGGTTGTATTGCTACCTGCCCTGAAAAATATGAAGCG
TGTTGCAGTACAGACATGTGCAACGAGTAG

>GAHE01000024 Furina ornata 3FTx-Fur-67 mRNA sequence
ATGAAAACTCTGCTGCTGACCTTGGTAGTGGTGACAATCGTGTGCCTGGACTTGGGATACACCCTGAAAT
GTCACAACACTTGGAATTATGGTTATATAAGTTGTGAAGCACACGAGACCTTTTGCTATAAATCTCTTCA
TTATGGACGTACAGAGAAACCTACGATCGCCCGGGGTTGTACTGCTAAATGCTCTGAAAAAAGTTTAGTG
TGTTGCAGTACAGACATGTGCAACGAGTAG

>GAHE01000025 Furina ornata 3FTx-Fur-74 mRNA sequence
ATGAAAACTCTGCTGCTGACCTTGGTAGTGGTGACAATCGTGTGCCTGGACTTAGGATACACCCTGAAAT
GTCACAACACTTGGAATTATGGTTATATAAGTTGTGAAGCACACGAGACCTTTTGCTATAAATCTCTTCA
TTATGGACGTACAGAGGAACCTACGATCGCCCGGGGTTGTACTGCTAAATGCTCTGAAAAAAGTTTAGTG
TGTTGCAGTACAGACATGTGCAACGAGTAG

>GAHE01000026 Furina ornata KP-Fur-10 mRNA sequence
ATGCGACCCCCGAGCGAGTTTCATCATGTCTTCTGGAGGTCTTCTTCTCCTGCTAGGACTCCTCACCCTC
TGGGCGGAGCTGACCCCCGTCTCCAGCAAGGGCCGGTCCAAAGTTAYTGTGAACTGCCTGCTGACCGCGG
ACCATGTAAAAGTTACTCTGAAGCCTTCTACTACAGCCCAGTTTATCATAAATGCCTAAGGTTTATGTAT
GGTGGATGCCCAGGGAATGCTAACAATTTTAAGACCATAGATGAATGCCAACGCACCTGTGCTGCATGA

>GAHE01000027 Furina ornata KP-Fur-17 mRNA sequence
ATGTCTTCTGGAGGTCTTCTTCTCCTGCTAGGACTCCTCACCCTCTGGGCCGAGCTGACCCCCGTCTCCA
GCAAGGACCGTCCAAAGTTCTGTGAACTGCCTCCTGACTCTGGACCATGTAAACAAAACATCAAAGCCTT
CTACTACAGTCATCATGCATGCCTACCGTTTATTTATGGTGGATGCCAAGGGAATGCTAACAATTTTAAG
ACCATAGATGAATGCCAACGCACCTGTGCTGCATGA

>GAHE01000028 Furina ornata KP-Fur-8 mRNA sequence
ATGTYTTCTGGAGGTCTTCTTCTCCTGCTAGGACTCCTCACCCTCTGGGCGGAGCTGACCCCCGTCTCCA
GCAAGGACCGTCCAAAGTTCTGTGAACTACCTGCTGACACCGGACTATGTAAAAGATACATCAAAGCCTT
CTACTACAGCCCAGGTCATCATGCATGCCTACCATTTGTTTATGGTGGCTGCCCAGGGAATGCTAACAAT
TTTAAGACCATAGATGAATGCAAACGCACCTGTGCTGCATGA

>GAHE01000029 Furina ornata KP-Fur-18 mRNA sequence
ATGCCAGCCATACAGGAGAGCTTCATCATGTCTTCTGGAGGTCTTCTTCTCCTACTGGGACTCCTCACCC
TCTGGGCAGAGCTGACCCCCGTCTTCAGCAAGGACCGTCCAAAGATCTGTGAACTGCCTGCTGACCGCGG
ACTATGTAAAAGATACATCAAAGCCTTCTACTACAGCCCAGGTCATCATGCATGCCTACCATTTATTTAT
GGTGGATGCCCAGGGAATGCTAACAATTTTAAGACCATAGATGAATGCAAACGCACCTGTGCTGCATGA

>GAHE01000030 Furina ornata LP-Fur-1 mRNA sequence
ATGGGGCGATTYCTCTTGGTGAGCCTCAGCTTGCTGGTCGTGGCTTTCTCCCTAAGTGGAATCGAAGCCA
ATCGCTATTGTCCCTTTGATTGGCTCTCCTACAATGTGTCTTGCTACAAGTTCTTCTTCCAAAGGATGAC
CTGGGACGAAGCACAGAGGTTCTGTGTGGAACAGCAGGAAAACTCCCAACTCGCCTCCATCCATGACATG
GGAGAGTCAGTCAAGTTGTCCAACTACATGTCCCAAAGATGGAATTTCTTTGATGTTTGGATGGGACTGA
GGCTTTCAAAGAGAAAAGGCATCTGGGAGTGGAGTGACGGCTCCAATCTCACTTACACATCCTGGGAAGA
TGGTGAACCCAACAACTTTTTCAACAGGGAATTCTGTGCTGTGCTATCAATCGGATCACGGTATCTCCAA
TGGAACGATAAGAACTGCATGCACAAGCATCCATTTGTCTGCAAGTTCCAGCCCCGGAGTGAGGCAGGCG
CGTTGGAGAAGCAGTGA

>GAHE01000031 Furina ornata LP-Fur-2 mRNA sequence
ATGGGGCGATTCGTCTTGGTGAGCCTGAGCTTGCTGCTTGTGACTTTATCCCTAAATGGAATTGGAGCTG
ATCATCACTGCCCCTCGGACTGGTACTCCTATGAGGCGTTTTGCTACAAGGTCGTCAAAGAATGGAAGAC
CTGGAAAGATGCCGAGGCATCCTGTGAACAACTCCAGGACAAAAGCCACCTGGCCTCCGTCCACAGCCTG
GCAGAGACCATTAACATCCGAAAGGCGATCTTCGGCAGTTCGATCCGCATCAGTGATATCTGGCTTGGAC
TGAGTAACTCAGAGGGAAATCGCAACTGGGAGTGGAGTGATGGCTCCAATTTTAATTATACAGCCTGGGA
ACTATTTGAACCCGATGATGATGAGGATGAAAACTGCGTTAAGTTGACCAGTATGTCAAGGTATTCTAAA
TGGAAAATTTCCAAGTGTGAATCCAAGAATTTTTTCATCTGCAAGATGTAG

>GAHE01000032 Furina ornata LP-Fur-3 mRNA sequence
ATGGGGCGATTCGTCTTGGTGAGCCTGAGCTTGCTGCTTGTGACTTTATCCCTAAATGGAATTGGAGCTG
ATCATCACTGCCCCTCGGATTGGTACTCCTATGAGGCGTTTTGCTACAAGGTCGTCAAAGAATGGAAGAC
CTGGAAAGATGCCGAGGCATCCTGTGAACAACTCCAGGACAAAAGCCACCTGGCCTCCGTCCACAGCCGA
GCAGAGACCATTAACATCCAAAAGGCAATCTTCGGCAGTTCGATCTTCGGCAGTGGTATCTGGATTGGAC
TGAGTAACTCAGAGGGAAATCGCAACTGGGMGTGGAGTGATGACTCCAATTATAATTATACAGCCTGGGA
ACCATTTGAACCCGATGATGATGAGGAGGAAAACTGCGTTAAGTTGACCAGTATGTCAATGTATTCTAAT
TGGAAAGTTTCCAAGTGTGAATCCAAGAATTTTTTCATCTGCAAGATGTAG

>GAHE01000033 Furina ornata LP-Fur-5 mRNA sequence
ATGGCACGATTCATTTCTCTAAGTCTTGGCTTGCTGGTTGTGGCCCTCTCCTTAAGGGGAACTGGAGCCA
ATCCCCAATGTCCCAACGAGTGGTCCTTCTTTAATGGATATTGCTACAAGGTCTTCAAACATCTCAAGAG
CTGGCGTGATGCAGAGATGTCCTGCAGGCGTCAGGAGGAAGGCAGCCACCTCGCCTCCATCCAAAGCTCG
GCAGAGTCATCTTACGTGGCCAGGTTGATCTCCCGCAATGTCTTCTTCATCAACGTCTGGATCGGACTGA
GTGATCCAGAGAGACGAGGCATCTGGAAGTGGAGCGATGGCTCCAGGTTCCTCTACAAATCCTGGAAAAG
AGGAGAACCCAACAACTTTTTCGGGAACGAGTACTGCGTCGAACTGTGGAGATTGTCAGGGTATCTCTCA
TGGAACGATAAGAATTGCTGGTCCAAGCTTTATTTCATCTGCAAGTTCCAGCCACAGGACGAGGGAAGCA
CCTGGTGA

>GAHE01000034 Furina ornata LP-Fur-6 mRNA sequence
ATGGCACGATTCATTTCTCTAAGTCTTGGCTTGCTGGTTGTGGCCCTCTCCTTAAGGGGAACTGTAGCCA
ATCCCCAATGTCCCTACGAGTGGTCCTTCTTTAATGGATATTGCTACAAGGTCTTCAAACAACTCAAGAG
CTGGCGTGATGCAGAGATGTCCTGCAGGCGTCAGGAGGAAGGCAGCCACCTCGCCTCCATCCAAAGCTCG
GCAGAGTCATCTTACGTGGCCAGGTTGATCTCCCGCAATGTCTTCTTCATCAACGTCTGGATCGGACTGA
GTGATCCAGAGAGACGAGGCATCTGGAAGTGGAGCGATGGCTCCAGGTTCCTCTACAAATCCTGGAAAAA
AGGAGAACCCAACAACTTTTTAGGGAACCAGTACTGCGTCGAACTGTGGAGCTTGTCAGGGTATCTCTCA
TGGAACGATAAGAATTGCTGGTCCAAGCTTTATTTCATCTGCAAGTTCCAGCCACAGGATGAGGGAAGCA
CCTGGTGA

>GAHE01000035 Furina ornata LP-Fur-7 mRNA sequence
ATGGCACGATTCATTTCTCTAAGTCTTGGCTTGCTGGTTGTGGCCCTCTCCTTAAGGGGCACTGGAGCCA
ATCCCCAATGTCCCCAAGAGTGGTCCTTCTTTAATGGATATTGCTACAAGGTCTTCAAACATCTCAAGAG
CTGGCGTGATGCAGAGATGTCCTGCAGGCGTCAGGAGGAAGGCAGCCACCTCGCCTCCATCCAAAGCTCG
GCAGAGTCATCTTACGTGGCCAAGCTGATCTCCCGCGATGTCTTCYTCATCAACGTCTGGATCGGACTGA
GTGATCCAGAGAAACGAGGCATCTGGAAGTGGAGCGATGGCTCCAGGTTCCTCTACAAATCCTGGAAAAG
AGGAGAACCCAACAACTTTTTAKGGAACGAGTACTGCGTCGAACTGTGGAGCTTGTCAGGGTATCTCTCA
TGGAACGATAAGAATTGCTGGTCCAAGCTTTATTTCATCTGCAAGTTCCAGCCACAGGATGAGGGAAGCA
CCTGGTGA

>GAHE01000036 Furina ornata NGF-Fur-1 mRNA sequence
ATGTCCATGCTGTGCTACACTCTGATTATAGCATTTCTGATTGGCACATGGGCAGCACCAAAATCTGAAG
ATAATGTCCCTCTGGGCTCCCCTGCAACATCTGACCTTTCTGACACCGGCTGTGCTAAAACTCATGAGGG
TCTGAAAACATCTCGAAATACAGATCAGCGCCATCCTGCTCCTAAGAAGGCAGAAGATCAAGAACTTGGA
TCAGCAGCCAACATCATTGTGGATCCCAAGCTTTTTCAGAAGTGGCGTTTCCAGTCACCTCGTGTTCTGT
TCAGCACTCAGCCCCCACCATTGTCAAGAGATGAGCAAAGTGTGGAGTTCCTGGACAATGAAGACACTCT
TAACAGGAATATCCGGACCAAACGTGAACATCATCCTGTGCATGACCAAGGAGAACATTCTGTGTGTGAC
AGTATCAGTGTCTGGGTTGCCAACAAAACTATAGCAACAGACATCAAAGGCAATGTGGTGACTGTGATAG
TGGACATAAATTTTAATAATGAAGTCTACAAGCAGTACTTTTTTGAAACCAAGTGCAGAAATCCAAATCC
AGTATCGAGTGGGTGCAGGGGCATTGATTCCAGGCATTGGAATTCTTATTGCACCACAAAACAAACATTT
GTCAAGGCATTAACCATGGAAGACAATCTGACATTCTGGCGCTTCATTCGGATTGACACTGCCTGTGTGT
GCGTAATCAGTAGAAAAGCTGACAACTTCTGA

>GAHE01000037 Furina ornata SVMP-Fur-1 mRNA sequence
ATGCAGCCAGAGGGCGAAGTAACTTTGGAATCATTTGGAGAATGGAGAGAGAAAAAATTGCTGCCACGCA
AAAGGAATGATAATGCTCAGTTACTCACGCGCATTGACTTCAGCGAAAATGTTGTAGGACTTGGTTATGT
GGGCACCCTCTGCAGTCCGAAGTCATCTGTAGCAGTTATTCAGGATTATAGCAGAAGAGCAAGTGTCATG
GCATCTGTAATGGCCCATGAGTTGGGTCATAATCTGGGCATTAATCATGACAGCGCTTCCTGTAATTGTA
ATGCTGGACGATGCATTATGTCTGCGATAATAAGTGATGAACCTCTCTATGAGTTCAGCTCTTGTAGTGT
CCAGGAACATCAGAGGTATCTTCTTAGACACAGACCACAATGCATTCTCAACAAACCCTTGAGCACAGAT
ATTGTTACACCTGCAGTTTGTGGAAATTACTTTGTGGAGTGGGAGAAGAATGTGATGTGGTTCTCCTCAG
GATTGTCAAAAGTGCCTGCTGCAAATGCTGCAAACTTGTAA

>GAHE01000038 Furina ornata VEGF-Fur-1 mRNA sequence
ATGAACTTTCTGCTCACTTGGATCCACTGCGGATTGGCGGCGCTGCTTTATTTCCACAACGCCAAGGTGT
TGCAGGCTGCTCCTGCCCAAGAGGATGGAGAGAAGCAACCAGAAGAAGTTATCCCATTTATGACGGTCTT
TGAGCGGAGTGCCTGCAGRCCCATTGAGACCATGGTAGATATTTACCAGGAGTATCCTGATGAGGTGGAA
TACATGTTCAAACCATCCTGTGTGCTCCTGATGAAATGTGGAGGATTCTGTAATGACGAAGCGCTAGAAT
GTGTGCCCACAGAGGTGTACAATGTCAGCATGGAGATCATGAAACTCAGACACTTTCAGAGCCAGCATAT
ACAACTGATGAGCTTCCAGCAGCACAGTAAATGTCAATGCAGACAAAAGAAACCACTGATAATTAAACAA
GAAAAATGTGACAAACCAAGGCGGTGA

>GAHF00000000.1|GAHF01000000 TSA: Hemiaspis signata, transcriptome shotgun assembly, transcriptome shotgun assembly

>GAHF01000001 Hemiaspis signata 3FTx-Hem-2 mRNA sequence
ATGAAAACTCTGCTGCTGACCTTGGTGGTGGTGACAATCGTGTGCCTGGACTTAGGTAACAGCTTCTCAT
GCTACAAAACACCTGATGTTAAATCTGAGGCTTGTGCAGCTGGGGAGAACCTATGCTATACAAAGACTTG
GTGTGGTTCTATGTGTTCCATCAGAGGAAGTTTAATCGAACTGGGATGTGCTGCTACTTGCCCTAAAGCG
AAGCCCGGTGTGGATATTACCTGTTGCTCAACAGACAATTGCAACCCACATCCGGCACACAACTCTCGTT
GA

>GAHF01000002 Hemiaspis signata 3FTx-Hem-5 mRNA sequence
ATGAAAACTCTGCTGCTGACCTTGGTGGTGGTGACAATCGTGTGCCTGGACTTAGGTAACAGCTTCTCAT
GCTACAAAACACCTGATGTTAAATCTGAGCCTTGTGCAGCTGGGGAGAACCTATGCTATACAAAGACTTG
GTGTGGTTCTATGTGTTCCATCAGAGGAAGTTTAATCGAACTGGGATGTGCTGCTACTTGCCCTAAAGCG
AAGCCCGGTGTGGATATTACCTGTTGCTCAACAGACAATTGCAACCCACATCCGGCACACAACTCTCGTT
GA

>GAHF01000003 Hemiaspis signata 3FTx-Hem-6 mRNA sequence
ATGAAAACTCTGCTGCTGACCTTAGTGGTGGTGACAATCGTGTGCCTGGACTTAGGTAACAGCTTCTCAT
GCTACAAAACACCTGATGTTAAATCTGAGGCTTGTGCAGCTGGGGAGAACCAATGCTATACAAAGACTTG
GTGTGGTTCTATGTGTTCCATCAGAGGAAGTTTAATCGAACTGGGATGTGCTGCTACTTGCCCTACAGCG
AAGCCCGGGGTGACTGTTAAGTGTTGCTCAACAGACAATTGCAACCCACATCCGGCACACAACTCTCGTT
GA

>GAHF01000004 Hemiaspis signata 3FTx-Hem-7 mRNA sequence
ATGAAAACTCTGCTGCTGACCTTGGTGGTGGTGACAATCGTGTGCCTGGACTTAGGTAACAGCTTCTCAT
GCTACAAAACACCTGATGTTAAATCTGAGGCTTGTGCAGCTGGGGAGAACCTATGCTATACAAAGACTTG
GTGTGGTTCTATGTGTTCCATCAGAGGAAGTTTAATCGAACTGGGATGTGCTGCTACTTGCCCTAAAGCG
AAGCCCGGTGTGGATATTACCTGTTGCTCAACAGACAATTGCAACCCACATCCGGCACACAACTCTCGTT
GA

>GAHF01000005 Hemiaspis signata 3FTx-Hem-9 mRNA sequence
ATGAAAACTCTGCTGCTGACCTTGGTGGTGGTGACAATCGTGTGCCTGGACTTAGGTGACAGCTTGAAAT
GCTACCTGAGCTCTACTCACTCTAAGGATTGTGCAGCTGGGGAGGACCTATGTTATACAAAGACTTGGTG
TGATGCTTGGTGTAGCCAGAGAGGACAGCGAGTCGAATTGGGATGTGCTGCTACTTGCCCTACAGCGAAG
CCCAGGGTGACTGTTAAGTGTTGCTCAACAGACAATTGCAACACATTTCCAGTACAACCTAAACCAAATC
CTCGAGGGTTGCTCTCATCCATCATGGACCATCCTTGA

>GAHF01000006 Hemiaspis signata 3FTx-Hem-11 mRNA sequence
ATGAAAACTCTGCTGCTGACCTTGGTGGTGGTGACAATCGTGTGCCTGGACTTAGGTGACAGCTTGTCAT
GCTACAAAACACCTCATGTTAAATCTGAGCCTTGTGCAGTTGGGGAGAACCAATGCTATACAAAGACTTG
GTGTGATACTTTTTGTAGCAGCAGAGGAAAGGTAGTCGAACTGGGATGTGCTGCTACTTGCCCTGTACCG
GAGTCCTACGAGGAAGTTACCTGTTGCTCAACAGACATTTGCAACAAATTTCCAGTACAATCTAAACCAT
ATCCTAGACCAGGGTTGCTCTCATCCATCATGGACCATCCTTGA

>GAHF01000007 Hemiaspis signata 3FTx-Hem-12 mRNA sequence
ATGAAAACTCTGCTGCTGACCTTGGTGGTGGTGACAATCGTGTGCCTGGACTTAGGTGACAGCTTGAAAT

GCTACCTGAGCTCTACTCACTCTAAGGATTGTGCAGTTGGGGAGGACCTATGCTATACAGAGACTTGGTG
TGATGCTTGGTGTGGCGTGAGAGGACAGCGAGTCAATTTGGGATGTGCTGCTACTTGCCCTACAGCGAAG
CCCAGGGTGACTGTTAAGTGTTGCTCAACAGACAATTGCAACACATTTCCAGTACAACCTAAACCAAATC
CTCGAGGGTTGCTCTCATCCATCATGGACCATCCTTGA

>GAHF01000008 Hemiaspis signata 3FTx-Hem-14 mRNA sequence
ATGAAAACTCTGCTGCTGACCTTGGTGGTGGTGACAATCGTGTGCCTGGACTTAGGTGACAGCTTGAAAT
GCTACCTGAGCTCTACTCACTCTAAGGATTGTGCAGTTGGGGAGGACCTATGCTATACAGAGACTTGGTG
TGATGCTTGGTGTGGCGTGAGAGGACAGCGAGTCGAATTGGGATGTGCTGCTACTTGCCCTACAGCGAAG
CCCAGGGTGACTGTTAAGTGTTGCTCAACAGACAATTGCAACACATTTCCAGTACAATCTAAACCAAATC
CTCGAGGGTTGCTCTCATCCATCATGGACCATCCTTGA

>GAHF01000009 Hemiaspis signata 3FTx-Hem-16 mRNA sequence
ATGAAAACTCTGCTGCTGACCCTGGTGGTGGTGACAATCGTGTGCCTGGACTTAGGATACACCATGACAT
GTTGCAACCAACAGTCATCGCAACCTAAAACCACTAAAACTTGTGAAGAGAGCACTTGCTATAAAAAGAC
TTGGAGAGATCACCGTGGAACTATCATTGAAAGGGGATGTGGTTGCCCTAAGGTGAAGCCCGGTATTCAA
CTTGAATGTTGCAGAACAGACGAATGCAACAATTAG

>GAHF01000010 Hemiaspis signata 3FTx-Hem-18 mRNA sequence
ATGAAAACTCTGCTGCTGACCCTGGTGGTGGTGACAATCGTGTGCCTGGACTTAGGATACGCCATAACAT
GTTGCGACCAACAGTCATCGCAACCTAAAACCACTAAAACTTGTGAAGAGAGCACTTGCTATAAAAAGAC
TTGGAGAGATCACCGTGGAACTATAATTGAAAGGGGATGTGGTTGCCCTAAGGTGAAGCCCGGTATTCAA
CTTGAATGTTGCGGAAAAGACGAATGCAACAATTAG

>GAHF01000011 Hemiaspis signata 3FTx-Hem-19 mRNA sequence
ATGAAAACTCTGCTGCTGACCCTGGTGGTGGTGACAATCGTGTGCCTGGACTTAGGATACACCATGACAT
GTTGCAACCAACAGTCATCGCAACCTAAAACCACTAAAACTTGTGAAGAGAGCACTTGCTATAAAAAGAC
TTGGAGAGATCACCGTGGAACTATCATTGAAAGGGGATGTGGTTGCCCTAAGGTGAAGCCCGGTATTCAA
CTTGAATGTTGCAGAACAGATGAATGCAACAATTAG

>GAHF01000012 Hemiaspis signata 3FTx-Hem-21 mRNA sequence
ATGAAAACTCTGCTGCTGACCCTGGTGGTGGTGACAATCGTGTGCCTGGACTTAGGATACACCATGACAT
GTTGCAACCAACAGTCATCGCAACCTAAAACCACTAAAACTTGTGAAGAGAGCACTTGCTATAAAAAGAC
TTGGAGAGATCACCGTGGAACTATAATTGAAAGGGGATGTGGTTGCCCTAAGGTGAAGCCCGGTATTCAA
CTTGAATGTTGCAGAACAGACGAATGCAATAATTAG

>GAHF01000013 Hemiaspis signata 3FTx-Hem-24 mRNA sequence
ATGAAAACTCTGCTGCTGACCCTGGTGGTGGTGACAATCGTGTGCCTGGACTTAGGATACACCATGACAT
GTTGCAACCAACAGTCATCGCAACCTAAAACCACTAAAACTTGTGAAGAGAGCACTTGCTATAAAAAGAC
TTGGAGAGATCACCGTGGAACTATCATTGAAAGGGGATGTGGTTGCCCTAAGGTGAAGCCCGGTATTCAA
CTTGAATGTTGCAGAACAGACGAATGCAACAATTAG

>GAHF01000014 Hemiaspis signata 3FTx-Hem-29 mRNA sequence
ATGAAAACTCTGCTGCTGACCTTGGTGGTGGTGACAATCGTGTGCCTGGACTTAGGATACGCCATAACAT
GTTGCAACCAACAGTCATCGCAACCTAAAACCACTACAACTTGTACAGAGAGCTCTTGCTATAAAAAGAC
TTGGAGAGATCACCGTGGAAGTATAACTGAAAGGGGATGTGGTTGCCCTGAGGTAAAGACCGGTGTTGGA
ATTAAATGTTGTGAAACAGATGAATGCAACAATTAG

>GAHF01000015 Hemiaspis signata CRiSP-Hem-1 mRNA sequence
ATGTTACAAATGGAATGGAATTCTCGTGCTGCTCAGAATGCAAAACGTTGGGCAGATAAATGTACTTTTG
CTCACAGTCCACGACATACAAGAACTGTGGGAAAACTCAGTTGTGGTGAAAATATATTCATGTCAAGTCA
ACCTTTTGCATGGAGTGGAGTAGTTCAGGCTTGGTATGATGAAGTTAAAAAATTCGTCTATGGCATTGGA
GCAAAGCCACCAGGTTCTGTTATTGGCCATTATACCCAGGTAGTTTGGTACAAAAGTCACCTTCTTGGTT
GTGCTTCTGCCAAATGTTCTTCAACCAAATACCTCTACGTTTGTCAATACTGCCCAGCAGGGAACATCAT
AGGTTCAATTGCTACTCCATATAAATCAGGGCCACCTTGTGGGGACTGTCCTTCGGCTTGTGTTAACGGA
CTATGCACAAATCCTTGCAAATATAATGATGACTTCTCGAACTGCAAAGCTTTAGCGAAAAAAACTAAAT
GCAAGACTGAATGGATCAAGTCAAAATGCCCTGCTGCTTGCTCCTGCCACAATAAAATAATATAG

>GAHF01000016 Hemiaspis signata KP-Hem-5 mRNA sequence
ATGTGCCAGGAATTCATTTTTGGAGGTTGCAAAGGCAATGCCAACAACTTCGTCTCCAAACAGGACTGCT
TCCAGGCGTGTATCAGAGGAGGGGCTGCAGAGGCAACAGTGGTACCCAGCGGGCCAGCCACTGAAGTGGC
TACACCAAGAGCTGGACATCTCCCAGAGGCCTACGAAAGCAGGCCTGGCTTCAGAGAATTCTGTGCTGCG
CCCCGGGTGGTAGGTCCCTGCCGGGCCTCCTTCCTCCGTTGGTACTTTGACCTAGAGAGCCGGATGTGCA
AGATGTTCATCTATGGGGGCTGTCGTGGCAACAAGAACAACTATCTCTTCGAAGAGCACTGCTGGAGCCA
GTGCACAGGTGATGGAGAGATAACTGAAGAGCCAGGAGATGCAGGCGCCCACCCTCCCCTCCCCTCCAAA
CCCTTCAGGTTCTCCACCAGAGCTGTGGTCCTTGCCGTCCTTCTGGCCATCCTGGTGACTATCCTGCTGG
GCTCGATGGGGGTTTTCTTTGTTAAGATCTGCCGGAAGAACCCGGAGTTGTCAGTGGGAACAGTGTGGAG
CACGCTGGATGACAAAGAGTACCTGATGAGCAATGCCTACACCCTCTGA

>GAHF01000017 Hemiaspis signata KP-Hem-8 mRNA sequence
ATGTCTTCTGGAGGTCTTCTTCTCCTGCTGGGACTCCTCACGCTCTGGGCGGAGCTGACCCCCGTCTCTG
GCCAGGACCGTCCAAAGTTCTGTGAACTGCCTGCTGACTCCGGATCATGTAAAGGTGACTTCCTAGCCTT
CTACTACAACCCAGATAAACATCAATGCCTAGAGTTTAGTTATGGTGGATGCGACGGGAATGATAACAAT
TTTAAGACCATAGAGGAATGCAAACGCACCTGTGCTGCATGA

>GAHF01000018 Hemiaspis signata KP-Hem-9 mRNA sequence
ATGTCTTCTGGAGGTCTTCTTCTCCTGCTGGGACTCCTCACGCTCTGGGCGGAGCTGACCCCCGTCTCTG
GCCAGGACCGTCCAAAGTTCTGTGAACTGCCTGCTGACTCCGGATCATGTAAAGGTGACTTCCTAGCCTT
CTACTACAACCCAAATCAACATCAATGCCTAGAGTTTAGTTATGGTGGATGCGACGGGAATGATAACAAT
TTTAAGACCATAGAGGAATGCAAACGCACCTGTGCTGCATGA

>GAHF01000019 Hemiaspis signata KP-Hem-12 mRNA sequence
ATGTCTTCTGGAGGTCTTCTTCTCCTGCTGGGACTCCTCACCCTCTGGGAGGTGCTGACCCCCGTCTCCA
GCAGGGACCGTCCACATTTCTGTCATCTGCCTCATGAAACCGGACTATGTAAACGCAATATCCAAGCCTT
CTACTACGACCCAGTTTATCATACATGCCTAAAGTTTATTTATGGTGGATGCGAAGGGAATGCTAACAAT
TTTAAGACCATAGATGAATGCAAACGCACCTGTGCTGCATGA

>GAHF01000020 Hemiaspis signata LAO-Hem-2 mRNA sequence
ATGGCTGGGCTTAGTGCAGCCTACGTTCTTGCAGGAGCTGGACATAAGGTGACGCTTCTTGAAGCTAGTG
AACGTGTGGGAGGGCGAGTGAACACTTATCGAAATGAAACGGAAGGCTGGTATGTCAATCTGGGACCCAT
GCGTCTCCCTGAGAGACACAGGATTATCCGGGAATATATCAGAAAATTTGGTCTCAAGTTGAACGAATTT

CTTCAGGAAAATGAGAATGCCTGGTATTTTATTAAAAACATCAGGAAGAGAGTATGGGAAGTCAAGAAAG
ACCCCGGYGTCTTCAAATATCCTTTGGAGCCCTCAGAAGAAGGCAAATCTGCTTCMCAGCTATATCGAGA
ATCCCTCGAAAAGGTTATAGAAGAATTGAATACA

>GAHF01000021 Hemiaspis signata NP-Hem-1 mRNA sequence
ATGGTCGGCCTCTCACGTCTGGCGGGCGGCGGGCTGCTGCTGCTGGTGCTGGCCCTGCTGCCTCTCGCCC
TCGACGGGAAGCCGGCGCCGCTGCCTCAGGCGCTGCTCAAGGCTCTGGCGGGCGGCATGATGGCGTCGGA
GCGGGAGCTGACGGAGAGGCAGAAGCTGCAACAGGAGCACCAGCCATCGCAGACGGAGGAGTCCTTGGGT
CCCGCGGCTGAGCGCAGCGGCTCCGAGACGGCCAAGCTAGGGGACGGCTGCTTCGGTGTCCCGATTGACC
ACATCGGCAGCTCAAGCGGCATGGGCTGCGGAGGCGTCCCGAAGCCGACACCTGGCGGATCCTAA

>GAHF01000022 Hemiaspis signata NP-Hem-2 mRNA sequence
ATGCTCTCACGTCTGGCGGGCGGCGGGCTGCTGCTGCTGGCCCTGCTGCCTCTCGCCCTCGACGGGAAGC
CGGCGCCGCTGCCTCAGGCGCTGCTCAAGGCTCCGGCGGGCGGCATGATGGCGTCGGGGCGGGAGCTGAC
GGAGCGGCAGAAGCTGCAACAGGAGCACCAGCCGCCGCAGACGGAGGAGTCCTCGGGTCCCGCGGCTGAG
CGCAGCGGCTCCGAGACGGCCAAGCTCGGGGACGGCTGCTTCGGCCTCCCGATCGACCGCATCGGCAGCA
CGAGCGGCATGGGCTGCGGAGGCGTCCCGAACCCAACACCTGGCGGATCCTAA

>GAHF01000023 Hemiaspis signata NP-Hem-3 mRNA sequence
ATGGTCGGCCTCTCACGTCTGGCGGGCGGCGGGCTGCTGTTGCTGGTGCTGGCCCTGCTGCCTCTCGCCC
TCGACGGGAAGCCAGCGCCGCTGCCTCAGGCGATGTTCAAGGCTCCGGCGGGCGGCATGATGGCGTCGGG
GCGGGAGCTGACGGAGAGGCAGAAGCTGCAACAGGAGCACCAGCCGTCGCTGACGGAGGAGTCCTTGGGT
CCCGCGGCTGAGCGCAGCGGCTCCGAGACAGCCAAGCTAGGGGACGGCTGCTTCGGTGTCCCGATTGACC
ACATCGGCAGCTCAAGCGGCATGGGCTGCGGAGGCGTCCCGAAGCCGACACCTGGCGGATCCTAA

>GAHF01000024 Hemiaspis signata NP-Hem-4 mRNA sequence
ATGGTCGGCCTCTCACGTCTGGCGGGCGGCGGGCTGCTGCTGGTGGTGCTGGCCCTGCTGCCTCTCGCCC
TCGACGGGAAGCCGGCGCCGCTGCCTCAGGCGCTGCTCAAGGCTCCGGCGGGCGGCATGATGGCGTCGGG
GCGGGAGCTGACGGAGCGGCAGAAGCTGCAACAGGAGCACCAGCCATCGCAGACGGAGGAGTCCTTGGGT
CCCGCGGCTGAGCGCAGCGGCTCCGAGACGGCCAAGCTCGGGGACGGCTGCTTCGGCGTCCGGCTCGACC
GCGTCGGCAGCACGAGCGGCATGGGCTGCGGAGGCGTCCCGAACCCAACACCTGGCGGATCCTAA

>GAHF01000025 Hemiaspis signata NP-Hem-5 mRNA sequence
ATGGTCGGCCTCTCACGTCTGGCGGGCGGCGGGCTGCTGTTGCTGGTGCTGGCCCTGCTGCCTCTCGCCC
TCGACGGGAAGCCAGCACCGCTGCCTCAGGCACTGCTCAAGGCTCCAGCGGGCGGCATGATGGCGTCGGG
GCGGGAGCTGACGGAGAGGCAGAAGCTGCAACAGGAGCACCAGCCGCCGCAGACGGAAGAGTCCTCGGGT
CCCGCGGCTGAGCGCAGCGGCTCCGAGACGGCCAAGCTAGGGGACGGCTGCTTCGGTGTCCCGATTGACC
ACATCGGCAGCTCAAGCGGCATGGGCTGCGGAGGCGTGCCGAAGCCGACACCTGGCGGATCCTAA

>GAHF01000026 Hemiaspis signata NP-Hem-7 mRNA sequence
ATGGTCGGCCTCTCACGTCTGGCGGGCGGCGGGCTGCTGCTGGTGGTGCTGGCCCTGCTGCCTCTCGCCC
TCTACGGGAAGCCGGCGCCGCTGCCTCAGGCGCTGCTCAAGGCTCCGGCGGGCGGCATGATGGCGTCGCG
GCGGGAGCTGACGGAGCGGCAGAAGCTGCAACAGGAGCACCAGCCGCCGCAGAGGGAAGAGTCCTCGGGT
CCCGCGGCTGAGCGCAGCGGCTCCGAGACGGCCAAGCTCGGGGACGGCTGCTTCGGCCTCCCGATCGACC
GCATCGGCAGCACGAGCGGCATGGGCTGCGGAGGCGTCCCGAACCCGACACCTGGCGGATCCTAA

>GAHF01000027 Hemiaspis signata NP-Hem-9 mRNA sequence
ATGGTCGGCCTCTCACGTCTGGCGGGCGGCGGGCTGCTGTTGCTGGTGCTGGCCCTGCTGCCTCTCGCCC
TCGACGGGAAGCCGGCGCCGCTGCCTCAGGCGATGTTCAAGGCTCCGGCGGGTGGCATGATGGCGTCGGG
GCGGGCGCTGACGGAGAGGCAGAAGCTGCAACAGGAGCACCAGCCATCGCAGACGGAGGAGTCCTCGGGT
CCCGCGGCTGAGCGCAGCGGCTCCGAGACGGCCAAGCTCGGGGACGGCTGCTTCGGCGTCCCGATTGACC
ACATCGGCAGCTCAAGCGGCATGGGCTGCGGAGGCGTCCCGAAGCCGACACCTGGCGGATCCTAA

>GAHF01000028 Hemiaspis signata NP-Hem-11 mRNA sequence
ATGGTCGGCCTCTCACGTCTGGCGGGCGGCGGGCTGCTGTTGCTGGTGCTGGCCCTGCTGCCTCTCGCCC
TCGACGGGAAGCCAGCACCGCTGCCTCAGGCACTGCTCAAGGCTCCAGCGGGCGGCATGATGGCGTCGGG
GCGGGAGCTGACGGAGAGGCAGAAGCTGCAACAGGAGCACCAGCCGCCGCAGACGGAAGAGTCCTCGGGT
CCCGCGGCTGAGCGCAGCGGCTCCGAGACGGCCAAGCTAGGGGACGGCTGCTTCGGTGTCCCGATTGACC
ACATCGGCAGCTCAAGCGGCATGGGCTGCGGAGGCGTGCCGAAGCCGACACCTGGCGGA

>GAHF01000029 Hemiaspis signata NP-Hem-12 mRNA sequence
ATGGTCGGCCTCTCACGTCTGGCGGGCGGCGGGCTGCTGCTGCTGCTGCTGGCCCTGCTGCCTCTCGCCC
TCGACGGGAAGCCAGCACCGCTGCCTCAGGCGCTGCTCAAGGCTCCGGCGGGCGGCATGATGGCGTCGGG
GCGGGAGCTGACGGAGCGGCAGAAGCTGCAACAGGAGCACCAGCCGCCGCAGACGGAGGAGTCCTCGGGT
CCCGCGGCTGAGCGCAGCGGCTCCGAGACGGCCAAGCTCGGGGACGGCTGCTTCGGCCTCCCGATCGACC
GCATCGGCAGCACGAGCGGCATGGGCTGCGGAGGCGTCCCGAACCCAACACCTGGCGGATCCTAA

>GAHF01000030 Hemiaspis signata NP-Hem-13 mRNA sequence
ATGGTCGGCCTCTCACGTCTGGCGGGCGGCGGGCTGCTGTTGCTGGTGCTGGCCCTGCTGCCTCTCGCCC
TCGACGGGAAGCCAGCACCGCTGCCTCAGGCACTGCTCAAGGCTCCAGCGGGCGGCATGATGGCGTCGGG
GCGGGAGCTGACGGAGAGGCAGAAGCTGCAACAGGAGCACCAGCCGCCGCAGACGGAAGAGTCCTCGGGT
CCCGCGGCTGAGCGCAGCGGCTCCGAGACGGCCAAGCTAGGGGACGGCTGCTTCGGTGTCCCGATTGACC
ACATCGGCAGCTCAAGCGGCATGGGCTGCGGAGGCGTCCCGAACCCGACACCTGGCGGATCCTAA

>GAHF01000031 Hemiaspis signata NP-Hem-14 mRNA sequence
ATGATGGCGTCGGAGCGGGAGCTGACGGAGAGGCAGAAGCTGCAACAGGAGCACCAGCCATCGCAGACGG
AGGAGTCCTTGGGTCCCGCGGCTGAGCGCAGCGGCTCCGAGACGGCCAAGCTAGGGGACGGCTGCTTCGG
TGTCCCGATTGACCACATCGGCAGCTCAAGCGGCATGGGCTGCGGAGGCGTCCCGAAGCCGACACCTGGC
GGATCCTAA

>GAHF01000032 Hemiaspis signata PLA2-Hem-3 mRNA sequence
ATGTCTCCTGCTCACCTTTTGGTCCTGTTGGCAGTTTGTGTCTCCCTCTTAGGAGCTGCCAACATTCGTC
CACAGCCTCTCAACCTCTATCAGTTCAAGAACATGATTGAATGTGCCAACCGTGGCAGTCGAAGTTGGTT
GGCTTATGCAAACTACGGTTGCTACTGCGGCTGGGGAGGTAGCGGGACACCTGTAGATGAGTTGGATACG
TGCTGCAAAATACATGACGACTGCTATGGTGAAGCCGAAAAAAAAGGATGCTACCCCAAGTTGACGTTGT
ATACTTGGCAATGTACTGAAAATGTACCCACCTGCGATTCAAAAACGGGGTGTGAAGGTTCTGTGTGTGA
TTGTGATGCCAAAGCAGCCATCTGCTTCGCCGGAGCCCCTTACAACTCTGCGAACATAAAAATCAACACC
AAGACACGTTGCCAATGA

>GAHF01000033 Hemiaspis signata PLA2-Hem-7 mRNA sequence
ATGTATCCTGCTCACCTTCTGGTCCTGTTGGCAGTTTGTGTCTCCCTCTTAGGAGCCTCCGACATTCGTC

CACAGCCTCTCAACCTCTATCAGTTCGGCAACATGATTGGATGTGCCAACCATAACACTCGAGGTTGGTC
GGATTATGCAAACTACGGTTGCTACTGCGGCTGGGGAGGTAGCGGGACACCGGTGGATGAGTTGGATAGG
TGCTGCAAAATACATGACGACTGCTATGGTGAAGCCGAAAAAAATTGCTTCCCCAAGTTGACGTTGTATA
GTTGGAAATGTACTGAAAATGTACCCACCTGCGATTCAAAAAAGGAGTGTGAAAGTTTTGTGTGTGATTG
TGATGCCAAAGCGGCCATCTGCTTCGCCGGAGCCCCTTACAACTCTGCGAACATAAAAATCGACACCAAG
ACACGTTGCCAATGA

>GAHF01000034 Hemiaspis signata PLA2-Hem-9 mRNA sequence
ATGTCTCCTGCTCACCTTCTGGTCCTGTTGGCAGTTTGTGTCTCCCTCTTAGGAGCCGCCAACATTCCTC
CGCAGCCTCTCAACCTCGTACAATTCAGCTACTTGATTCAATGTGCCAACCATGGCAGTCGTGCTACTTG
GCATTATATGGACTACGGTTGCTACTGCGGCTCAGGAGGTAGCGGGACACCGGTAGATGAGTTGGATACG
TGCTGCAAAATACATGACGACTGCTATGGTGAAGCCGAAAAACAAGGATGCTCCCCCAAGATGTTGGCGT
ATGATTACTACTGTGGCGAAAATGGACCCTACTGCAGAAATATCGAAAAGAAGTGTCAACGTATTGTGTG
TGATTGTGACGTCAAAGCAGCCTTCTGCTTTGCCAAAGCCCCTTACAACAACGCGAACTGGAATATCGAC
ACCACTACACGTTGCCAATGA

>GAHF01000035 Hemiaspis signata PLA2-Hem-10 mRNA sequence
ATGTCTCCTGCTCACCTTCTGGTCCTGTTGGCAGTTTGTGTCTCCCTCTTAGGAGCCGCCAACATTCCTC
CGCAGCCTCTCAACCTCGTACAATTCAGCTACTTGATTCAATGTGCCAACCATGGCAGTCGTGCTACTTG
GCATTATATGGACTACGGTTGCTACTGCGGCTCAGGAGGTAGCGGGACACCGGTAGATGAGTTGGATACG
TGCTGCAAAATACATGACGACTGCTATGGTGAAGCCGAAAAACAAGGATGCTCCCCCAAGATGTCGGCGT
ATGATTACTACTGTGGCGAAAATGGACCCTACTGCAGAAATATCGAAAAGAAGTGTCAACGTATTGTGTG
TGATTGTGACGTCAAAGCAGCCTTCTGCTTTGCCAAAGCCCCTTACAACAACGCGAACTGGAATATCGAC
ACCACTACACGTTGCCAATGA

>GAHF01000036 Hemiaspis signata PLA2-Hem-11 mRNA sequence
ATGTCTCCTGCTCACCTTCTGGTCCTGTTGGCAGTTTGTGTCTCCCTCTTAGGAGCCGCCAACATTCCTC
CGCAGCCTCTCAACCTCATACAATTCAGCTACTTGATTCAATGTGCCAACCATGGCAGTCGACCTACTTT
GCATTATATGGACTACGGTTGCTACTGCGGCTCAGGAGGTAGCGGGACACCAGTAGATGAGTTGGATACG
TGCTGCAAAATACATGACGACTGCTATGGTGAAGCCGAAAAAAAAGGATGCTCCCCCAAGATGTTGGCGT
ATGATTACTACTGTGGCGAAGATGGACCCTACTGCAGAAATATCGAAAAGAAGTGTCAACGTATTGTGTG
TGATTGTGACGTCAAAGCAGCCAAGTGCTTTGCCAAAGCCCCTTACAACAACGCGAACTGGAATATCGAC
ACCACTACACGTTGCCAATGA

>GAHF01000037 Hemiaspis signata SVMP-Hem-2 mRNA sequence
ATGCAATATGAATTTCACGTGAATGGAGAGCGAGTGGTCCTTCACTTAGAAAGAAATAAAGGACTTTTTT
CAGAAGATTACACTGAAACTCATTATGCCCCTGATGGCAGAGAAATTACAACAAGCCCTTCGGTTCAGGA
TCACTGCTATTATCATGGCTACATTCAGAATGAAGCTGATTCAAGTGCAGTCATCAGTGCATGCGACGGC
TTGAAAGGACATTTCAAGCATCGAGGGGAGACATACTTTATTGAGCCCTTGAAGCTTTCCGACAGTGAAT
CCCATGCAATCTACAAAGATGAAAATGTAGAAAAAGAGGATGAGATCCCCAAAATCTGTGGGGTAACCCA
TACTACTTCGGAGTCAGATGAGCCCATCGAAAAGATCTCTCAATTAACGAATGCTTCTCAACAAGACAGG
TACTTGCAGGTCAAAAAATACATTGAGTTGTATGTAGTTGTGGACAACAGAATGTACAGGAATTACAACA
GCAACAGAGATGCTATAAATGAAAGAGTATATGAAATGGTCAACACTTTAAATGTGATGTACAGACCTTT
GAATTTTTTCACTGCACTGATTGGCCTAGAAATTTGGACCAACCAAGATGAGATTAACGTTGAACCAGAT
GCGTCTGTCACTTTGAAATCATTTGGAGATTGGAGAGAAACAGATTTACTGCCACGCAGAAGGAATGATA
ATGCTCAGTTACTTACGAGCATTGACTTCAACGGAGCTACTGTAGGACTTGCTTACGTCGGCTCCCTCTG
CAATCCGAAGTACTCTGTAGCAGTTATTCAGGATCATAGCAATAGAACAAGCATAGTGGCATCTACAATG
GCCCATGAGTTGGGTCATAATCTGGGCATTCATCATGACAGGGATTTCTGTAATTGCAGTGCTATAGGGT
GCATTATGTCTGCGACAATAAGTAATGAACCTTTCTATGAGTTCAGCAATTGTAGTATCCAGGAACATCA
GAGCTATCTTCTTAGAATCAGACCACAATGCATTCTCAACAAACCCTTGAGCACAGATATTGTTACACCT
CCAGTTTGTGGAAATTACTTAGTGGATGTGGGAGAAGAATGTGACTGTGGCTCTCCTGTCGATTGTCAAA
GTGCCTGCTGCAATGCTACAACTTGTAAACTGCAACATGAGGCACAGTGTGACTCTGGAGAGTGTTGTGA
GCAATGCCAATTTACGGAAGCAGGAAGAGAATGCCGGGCAGCAAAGGATGACTGTGACTTGCCTGAAAGC
TGCACTGGCCAATCTGCTGAGTGTCCCACGGACAGCTTCCAGAGGAATGGACATCCATGCCAACACAACC
AAGGTTACTGCTACAATGGGACATGCCCCCTCATGACAAACCAGTGTATTGCTCTTGGGGGACCAGGTGT
AAATGTGTCTCCAGATGGATGTTTTATGTCCAACCGGTATGGCCAAGGTTGTGGCTTCTGCAGAATAGAA
AATCGTAGAAAGATTCCATGTGCAGCAAAGGATGTAAAGTGTGGCAAGTTATACTGCCAACAGGGAAACA
ATGCATGCCTCTGCTATAATTCATTACATGACCCAGATTCTGGAATGGTTAAACCTGGAACAAAATGTGG
AGATGGAAAGGTGTGCATCAACAGGCAGTGTGTTGATGTGCAGACAGCCTACTGA

>GAHF01000038 Hemiaspis signata SVMP-Hem-3 mRNA sequence
ATGATCCAAGCTCTCTTGGTAACTATATGCTTTGCGGTTTTTCCATATCAAGGGAGCTCTATCATCCTGG
AATCCGGGAATGTTAATGATTATGAAGTAGTGTATCCACAAAAAGTGCCTGCATTGCCCAAAGGAGGAGT
TCAGAATCCTCAGCCAGAGACCAAGTATGAAGATACAATGCAATATGAATTTCACGTGAATGGAGAGCGA
GTGGTCCTTCACTTAGAAAGAAATAAAGGACTTTTTTCAGAAGATTACACTGAAACTCATTATGCCCCTG
ATGGCAGAGAAATTACAACAAGCCCTTCGGTTCAGGATCACTGCTATTATCATGGCTACATTCAGAATGA
AGCTGATTCAAGTGCAGTCATCAGTGCATGCAACGGCTTGAAAGGACATTTCAAGCATCGAGGGGAGACA
TACTTTATTGAGCCCTTGAAGCTTTCCGACAGTGAATCCCATGCAATCTACAAAGATGAAAATGTAGAAA
AAGAGGATGAGATCCCCAAAATCTGTGGGGTAACCCATACTACTTCGGAGTCAGATGAGCCCATCGAAAA
GATCTCTCAATTAACGAATGCTTCTCAACAAGACAGGTACTTGCAGGTCAAAAAATACATTGAGTTGTAT
GTAGTTGTGGACAACAGAATGTACARSAATTACAACAGCAACAGAGATGCTATAAATGAAAGAGTATATG
AAATGGCCACTATGCTTGTTCTATTGCTA

>GAHF01000039 Hemiaspis signata SVMP-Hem-5 mRNA sequence
ATGATCCAAGCTGTCTTGGTTACTATATGCTTAGTGGTTTTTCCATATCAGGTGAGCTCTACAATCCTGG
AATCTGGGAAAGGTAAAGATTATGAAGTAGTGTATCCACAAAAAATTCCTTCATTGCCCAAAGGAGGACT
TCAGAAGCTTGAGGAAAAGACCAAATATGAAGATACCATGAAATATGAATTTAAAGTGAATGGAGAGCCA
GTGGTCCTTAACCTAGAAAAAAATAAACGACTTTTTTCAAAAGATTATACTGAGACTCATTATTCCCCTG
ATGGCAGAGAAATTACAACAAGCCCTCCAGTTCAGGACCACTGCTATTATCATGGACACATCCAGAATGA
TGCTGACTCAAGTGCAGTCATCAGGGCTTGTGATGGCTTGAATGGGTATTTCAAGAATAACGGTGAGATG
TACATTATTGAACCTTTGAAGCATCCTGACGAT

>GAHF01000040 Hemiaspis signata SVMP-Hem-11 mRNA sequence
ATGTGCAATAATGACATATCTGTAGGAGTTATTCAGGATAACAGCTTCCAGGCAATTACAACTGCAGCTG
TAATGACCCACGAGCTGGGTCATAATCTTGGTATGGATCATGATACAGATTCCTGTACTTGCAACATAGG
ACCATGCATTATGGAACCCTCAATAAATTTTAACCCTCCTCGGGAGTTCAGCTCTTGCAGTTTCCGGGAT
TATCAGAATTATATCATGACTGAAACTGCACAATGCATTCTCAATGACCCCTTGACGACAGATATTGTTC
CAATTGCAATTTGTGGAAATGGTTTTGTGGAAGAGGGAGAAGAATGTGACTGTGGCCTTCCAGAGATATG
TAAAAATGAGTGCTGTGAAGCTGCAACTTGTAAACTGAAACCTGAGGCAGAGTGTGCAAGTGGAGCATGT
TGTGAGAAATGCCAGTTTAGGAGAGCTGGAGAAGTATGCCGGGCAGCAAAGGATGACTGTGACTTTGATG
AACTCTGCACTGGCCAATCTGCTGAGTGTCCCATGAATCTCTTCCATATGGATGGACACCCATGCCAAAA

CAACCAAGGTTACTGCTTCAGGGGGACATGTCCCACCTTAAAAAAGCAATGCATTGCTCTCTGGGGGCCA
GATGCAGAAGTGGCTCCAGATGGTTGTTTTATGAATAACCAGAAAGGGAAAGATTATGGCTACTGTAAAA
AGGAAAACGGTACAAACATTCCATGTGAACCAAAGGATGTTAAATGTGGCAGGTTATACTGCATAGATGA
TTCAACTGAAGAGAAAATCATGACTAAATTTTACTTTTACAAAACGAAAATGCCAATTTCGGAATGGTTG
AACCTGGAACAAAGTGTAGGAGAAGGAATGGTCTGTGGCTCCGGGCAGTGTATTAACCTAGAAACAGCAC
GATC

>GAHF01000041 Hemiaspis signata SVMP-Hem-7 mRNA sequence
ATGTACAGGAATTACAACAACAACAGAGATGCTATAAATGAAAGAGTATATGTAATGGTCAACACTTTAA
ATGTGATGTACAGACCTTTGAATTTTATCATTGCACTGATTGGCCTAGAAATTTGGACCAACCAAGATGA
GATTAACATTGAACCAGATGTGTCTGTCACTTTGAGATCATTTGGAGATTGGAGAGAAACAGATTTACAG
CCACGCAGAAGGAATGATAATGCTCAGTTACTTACGAGCATTGACTTCAACGGAGCTACTGTAGGACTTG
CTTACGTGGGCACCGGTGCAGAATGA

>GAHG00000000.1|GAHG01000000 TSA: Hoplocephalus bungaroides, transcriptome shotgun assembly, transcriptome shotgun assembly

>GAHG01000001 Hoplocephalus bungaroides 3FTx-Hop-7 mRNA sequence
ATGAAAACTCTGCTGCTGACCTTCGTGGTGGTGACAATCGTGTGCCTGGACTTAGGTAACAGCTTCTCAT
GCTACAAAACACCTGGTGTTAAATCTGAGCCTTGTGCACCTGGGGAGAACCTATGCTTTACAAAGACTTG
GTGTGATGCTTTTTGTGGCCACAGAGGAAAGCGAGTCGAAATGGGATGTGCTGCTACTTGCCCTACAGTG
AAGCCCGGTGTGGATATTATCTGTTGCTCAACAGACAATTGCAACCCATTTCCAACACACAACTCTCGTT
GA

>GAHG01000002 Hoplocephalus bungaroides 3FTx-Hop-9 mRNA sequence
ATGAAAACTCTGCTGCTGACCTTGGTGGTGGTGACAATCGTGTGCCTGGACTTAGGTGACAGCTTGATAT
GCTACATGGGATATAATGCTCCTCAGACTTGTCCACCTGGGGAGAACCTATGCTTTACAAAGAGTTGGTG
TGATGCTCACTGTGGCCAGAGAGGAAAGCGAATCGATTTGGGATGTGCTGCTACTTGCCCTACAGTGAAG
CCCGGTGTGGATATTAGCTGTTGCTCAACAGACAAGTGCAACCCACATCCAAAACAGCAACGTCGTTGA

>GAHG01000003 Hoplocephalus bungaroides 3FTx-Hop-14 mRNA sequence
ATGAAAACTCTGCTGTTGACCTTGGTGGTGGTGACAATCGTGTGCCTGGACTTAGGATACACCATGACAT
GTTGCAACCAACAGTCATCGCAACCTAAAACCACTAAAACGTGTGCAGAGAGCTCTTGCTATAAAAAGAC
TTGGAGAGATCACCGTGGAACTATAATTGAAAGGGGATGTGGTTGCCCTCACGTGAAGCCCGGTATTCAA
CTTGTATGTTGCAAAACAGACGAATGCAACAATTAG

>GAHG01000004 Hoplocephalus bungaroides 3FTx-Hop-18 mRNA sequence
ATGAAAACTCTGCTGTTGACCTTGGTGGTGGTGACAATCGTGTGCCTGGACTTAGGATACACCATGACAT
GTTGCAACCAACAGTCATCGCAACCTAAAACCACTAAAACTTGTGCAGAGAGCTCTTGCTATAAAAAGAC
TTGGAGAGATCACCGTGGAACTATAATTGAAAGGGGATGTGGTTGCCCTCACGTGAAGCCCGGTATTCAA
CTTGTATGTTGCAAAACAGACGAATGCAACAATTAG

>GAHG01000005 Hoplocephalus bungaroides 3FTx-Hop-22 mRNA sequence
ATGAAAACTCTGCTGCTGACCTTGGTGGTGGTGACAATCGTGTGCCTGGACTTAGGATACACCATGACAT
GTTGCAACCAACAGTCATCGCAACCTAAAACCACTAAAACTTGTGCAGAGAGCTCTTGCTATAAAAAGAC
TTGGAGAGATCACCGTGGAACTATAATTGAAAGGGGATGTGGTTGCCCTCACGTGAAGCCCGGTATTCAA
CTTGTATGTTGCAAAACAGACGAATGCAACAATTAG

>GAHG01000006 Hoplocephalus bungaroides 3FTx-Hop-25 mRNA sequence
ATGAAAACTCTGCTGTTGACCTTGGTGGTGGTGACAATCGTGTGCCTGGACTTAGGATATACCATGACAT
GTTGCAACCAACAGTCATCGCAACCTAAAACCACTATACCTTGTCCAATGATGTTTTGCTATAAAATGAC
TTGGAGAGATCACCGTGGAACTATAATTAAAAGGGGATGTGGTTGCCCTCACGTGAAGCCCGGTATTCAA
ATTGAATGTTGCAAAACAAACGAATGCAACAATTAG

>GAHG01000007 Hoplocephalus bungaroides fX-Hop-1 mRNA sequence
ATGGCTCATCAGCTACTCCTCTGTCTGATCCTCACTTTTCTGTGGAGTGTCCCAGAGGCTGAAAGTAATG
TATTCTTAAAAAGCAAAGTGGCAAATAGATTTTTGCAAAGAACAAAACGATCTAATTCACTGTTTGAGGA
AATTAGACCTGGAAACATTGAAAGGGAATGCATTGAGGAGAAATGTTCAAAAGAAGAAGCCAGGGAGGTA
TTTGAAGATAACGAGAAAACTGAGACCTTCTGGAATGTTTATGTAGATGGGGATCAGTGTTCATCAAACC
CCTGTCATTATCACGGGACATGCAAAGATGGCATTGGTAGCTATACCTGTACCTGCTTGCCTAACTATGA
AGGGAAAAACTGTGAAAAAGTCTTATTTAAGTCCTGCAGAGCGTTCAATGGTAACTGTTGGCACTTCTGC
AAACGTGTTCAAAGTGAAACTCAGTGTTCATGTGCTGAAAGTTACCGTTTGGGAGTTGATGGGCACTCTT
GTGTTGCTGAAGGTGACTTTTCATGTGGTAGAAATATAAAAGCAAGGAACAAGAGGGAAGCAAGTCTGCC
TGACTTTGTGCAGTCCCAGAAGGCAACTTTGCTGAAAAAATCTGATAATCCAAGCCCTGATATCAGAATT
GTTAATGGAATGGACTCCAAACTGGGTGAATGTCCATGGCAGGCAGTTCTGATAAATGAAAAAGGAGAAG
TGTTTTGTGGAGGAACAATTTTGAGTCCCATCCATGTGCTTACTGCAGCCCACTGCATTAACCAGACCAA
GAGCGTTTCAGTTATTGTAGGGGAAAATAGACATATCAAGAAAAGAAACCAGACGTCTTCTTTCTGTGGA

>GAHG01000008 Hoplocephalus bungaroides KP-Hop-3 mRNA sequence
ATGTCTTCTGGAGGTCTTCTTCTCCTGCTGGGACTCCTCACCCTCTGGGAGGTGCTGACCCCCGTCTCCA
GCAAGGACCGTCCAGAGTTCTGTGAACTGCCTGATGACTCCGGACCATGTAGAGGCATCTTCCATGCCTT
CTACTACAATCCAGATCAACGTCAATGCCTAGAGTTTATTTATGGTGGATGCTATGGGAATGCTAACAAT
TTTAAGACCATAGATGAATGCAAACGCATCTGTGCTGCATGA

>GAHG01000009 Hoplocephalus bungaroides KP-Hop-7 mRNA sequence
ATGACGTGCGAGAAAAGTCTGGCGCTGTTGATCACGTTGGCGGCTGCGCTGGCGGCCGCAGAGTCGCCGC
CAGGCCGATGCCACTCGCCCAAGACAGTGGGGCCTTGTCGGGCATCCTTCCATCGGTGGTGGTACAATGC
CACGAGTCAGATGTGCCAGGAATTCATTTTTGGAGGTTGCAAAGGCAATGCCAACAACTTTGTCTCCAAA
CAGGACTGCTTCCAGACGTGTATCAGAGGAGGGGCTGCAGAGGCAACAGTGGTATCTAGCGGGCCAGCCA
CTGAAGTGGCTACACCAAGAGCTGGACATCTCCCAGAGGCCTACGAAAACAGGCCTGGCTTCAGAGAATT
CTGTGCTGCGCCCCGGGTGGTAGGTCCCTGCCGGGCCTCCTTCCTCCGTTGGTACTTTGACCTAGAGAGC
CGGATGTGCAAGATGTTCGTCTATGGGGGCTGTCGTGGCAACAAGAACAACTATCTCTTCGAAGAGCACT
GCTGGAGCCAGTGCACAGGTGATGGAGAGATAACTGAAGAGCCAGGAGATGCAGGCGCCCAGCCTCCCCT
CCCCTCCGAACCCTTCAGCTTCTCCACCAGAGCTGTGGTCCTTGCCGTCCTTCTGGCCATCCTGGTGACT
ATCCTGCTGGGCTCGATGGGGGTTTTCTTTGTTAAGATCTGCCGGAAGAACCCGGAGTTGTCAGTGGGAA
CGAGTGTGGAGCACGCTGGATGA

>GAHG01000010 Hoplocephalus bungaroides KP-Hop-9 mRNA sequence
ATGTCTTCTGGAGGTCTTCTYCTCCTGCTGGGACTCCTCACCCTCTGGGAGGTGCTGACCCCCGTCTCCA
GCAAGGACCGTCCAAAGTTCTGTGAACTGCCTGCTGACTCCGGATCATGTAAAGGCAACTTCCAAGCCTT

CTACTACAACCCAGTTCAACATCAATGCCTAGAGTTTATTTATGGTGGATGTGACGGGAATGCTAACAAT
TTTAAGACCATAGATGAATGCAAACGCACCTGTGCTGCATGA

>GAHG01000011 Hoplocephalus bungaroides KP-Hop-11 mRNA sequence
ATGTCTTCTGGAGGTCTTCTTCTCCTGCTGGGACTCCTCACCCTCTGGGAGGTGTTGACCCCCGTTTCCA
GCAAGGACCGTCCACACTTCTGTCATCTGCCTCATGAAACCGGACCATGTAAACGCAACACCCAAGCCTT
CTACTACAACCCAGTTTATCATACATGCCTAAAGTTTATTTATGGTGGATGCGAAGGGAATTCTAACAAT
TTTAAGACCATAGATGAATGCAAACGCACCTGTGCTGCATGA

>GAHG01000012 Hoplocephalus bungaroides LAO-Hop-2 mRNA sequence
ATGATTGGAAAGTTACTGAATGAAGATTCTAGCTATTACCTGTCTTTTATTGAGAGCCTGAAAAGTGATG
ATATCTTCTCTTATGAAAAAAGATTTGATGAAATTGTTGGTGGATTTGATCAATTGCCTATATCCATGTA
TCAAGCCATTGCAGAAATGGTGCATTTAAATGCTCAAGTAATCAAGATACAACATAATGCTGAGGAAGTC
AGAGTGGCATATCAAACTCCAGCAAAGACCTTGTCATATGTGACAGCCGATTATGTCATTGTATGCTCCA
CATCAAGGGCCACCCGTCGCATCTATTTTGAACCTCCCCTTCCACCAAAGAAAGCACACGCTTTGCGGTC
TATCCACTACAGAAGTGGCACCAAGATCTTCCTCACTTGCACTAGGAAGTTTTGGGAGGCTGATGGCATT
CATGGTGGGAAGTCCCACAACTGA

>GAHG01000013 Hoplocephalus bungaroides LP-Hop-1 mRNA sequence
ATGGGGCGATTCCTCTTGGTGAGCCTGAGCTTGCTGGTCGTGACTTTATCCCTAAATGGAATTGGAGCTG
ATCATCATTGTCCCTCGGACTGGATCTCCCTTGGGAAGTTTTGCTACAAGGTCATCAGAGAATGGAAGAC
CTGGGAAGATGCCGAGGCATCCTGCGTGCAGCTTCAGAACAGCAGCCATCTGGCCTCCATCCACAGCCTA
GCAGAGTCCCTTTACATCCAAACGGCGATCTCCAGGAGACTGGTCTTCACTGATGTCTGGATTGGACTGA
ATGATCCAGGGAAAAATCGCACCTGGGAGTGGACTGATGGCTCCGATTTTGATTATACATCCTGGGGAAT
ATTTGAACCCGGCAATACTGATCGTGAGGAGTACTGCGCTAAGTTGAGAAGTGTCTCAAGGCATTCTAGA
TGGGAAGCTGCCAAGTGTGAATCCAAGAATCGTTTCATCTGCAAGATGTAG

>GAHG01000014 Hoplocephalus bungaroides LP-Hop-2 mRNA sequence
ATGCTTCTCATCACTTGCTTCATCTTTGGCCTCTTGGGCAGCCTTTCCTGGGCAGGTCCACATGCTCGGA
GTGTCTGTCCTTCTGGCACCTTTGCCAACAGAGACAGAAGCCAGTGGTATTGCTACAAGTTCTATGAAGA
TATGGCCACCTTTCAAGAAGCTGAGGAAGAGTGTCAGTACAAATGGAGAGGTCATTTAGCATCTTTCACA
AGAGACAAGCAGGCAGAAACCGTTGGTGCCTATATCAATAAAGAAAATACAGAGAATAACCACGTCTGGA
TTGGGCTCCAACGAGAGAATCTGGCTAGTGCATGGCGCTGGACTGATGGATCACGATCCAAGTACAGAAA
GTTCAACTGGGCAAAAACTCAAGTGGAATCTTACAATGAACATTGCGCTGCCTTCTCTCCTGTATATGGG
CGCCTGAGTTGGTCTGAGCAGGACTGTAGCAGCAAATTTCCTTTTCTCTGCAAATGGAAGCCCTCTTAA

>GAHG01000015 Hoplocephalus bungaroides NP-Hop-00039 mRNA sequence
ATGCTCGGCCTCTCACGTCTGGCGGGCGGCGGGCTGCTATTGGTGGTGCTGGCCCTACTGCCTCTTGCCC
TCGACGGGAAGCCGGCGCCACCGCCTCAGGCGCTGCCCACGGCTCCGGCGGGCGGCATGATGGCGTCGCG
GCCGGTGCTGACGGAGCAGCAGAAGCAGCAGCAGCACCAGCAGCAGCAGCACCAGCAGCCGCTGCTCCCG
CTGCCGCCGCCGACGATGGAGGATTCCTCGGGTCCCGCGGCTGAGCGCAGCGGCTCCAAGACGGCCAAGA
TCGGGGACGGCTGCTTCGGCCTCCCGATCGACCGCATCGGCAGCACGAGCGGCATGGGCTGCGGAAGCGT
CCCGAAGCCGACACCTGGCGGATCCTAA

>GAHG01000016 Hoplocephalus bungaroides NP-Hop-00040 mRNA sequence
ATGGTCGGCCTCTCACGTCTGGCGGGCGGCGGGTTGCTGCTGCTGCTGGCCCTGCTGCCTCTCGCCCTCG
ACGGGAAACCGGCGCCGCCGCCTCAGGCGCTGCCCACGGCTCCAGCGGGCGGCATGATGGCGTCGCGGCC
GGTGCTGACGGAGCAGCACCAGCAGCAGCAGCACCACCAGCAGCAGCACCAGCAGCCGCTGCTGCCGCTG
CCGCCGCCGACGATGGAGGATTCCTCGGGTCCCGCGGCTGAGCGCAGCGGCTCCAAGACGGCCAAGATCG
GGGACGGCTGCTTCGGTGTCAGGCTCGACCGCATCGGCAGCACGAGCGGCATGGGCTGCGGAGGCGTCCC
GAAGCCGACACCTGGCGGATCCTAA

>GAHG01000017 Hoplocephalus bungaroides NP-Hop-00061 mRNA sequence
ATGGTCGGCCTCTCACGTCTGGCGGGCGGCGGGTTGTTGCTGCTGCTGCTGGCCCTGCTGCCTATCGCCC
TCGACGGGAAACCGGCGCCGCCGCCTCAGGCGCTGCCCACGGCTCCGGCGGGCGGCATGATGGCGTCGCG
GCCGGTGCTGACGGAGCAGCAGAAGCAGCAGCAGCACCACCAGCAGCAGCAGCCGCTGCTGCCGCTGCCG
CCGCCGACGATGGAGGATTCCTCGGGTCCCGCGGCTGAGCGCAGCGGCTCCAAGACGGCCAAGATAGGGG
ACGGCTGCTTCGGCCTCCCGATCGACCGCATCGGCAGCACGAGCGGCATGGGCTGCGGAGGCGTCCCGAA
GCCGACACCTGGC

>GAHG01000018 Hoplocephalus bungaroides NP-Hop-00064 mRNA sequence
ATGGTCGGCCTCTCACGTCTGGCGGGCGGCGGGTTGTTGCTGCTGCTGCTGGCCCTGCTGCCTATCGCCC
TCGACGGGAAACCGGCGCCGCCGCCTCAGGCGCTGCCCACGGCTCCGGCGGGCGGCATGATGGCGTCGCG
GCCGGTGCTGACGGAGCAGCAGAAGCAGCAGCAGCACCACCAGCAGCAGCAGCCGCTGCTGCCGCTGCCG
CCGCCGACGATGGAGGATTCCTCGGGTCCCGCGGCTGAGCGCAGCGGCTCCAAGACGGCCAAGATAGGGG
ACGGCTGCTTCGGCCTCCCGATCGACCGCATCGGCAGCACGAGCGGCATGGGCTGCGGAGGCGTCCCGAA
GCCGACACCTGGCGGATCCTAA

>GAHG01000019 Hoplocephalus bungaroides NGF-Hop-1 mRNA sequence
ATGTTGTGCTACACTCTGATTATTGCATTTCTGATTGGCATATGGGCAGCACCAAAATCTGAGGATAATG
TCCCTCTGGGCTCCCCTGCAACATCTGACCTTTCTGACACCAGCTGTGCTCAAACTCATGAGGGTCTGAA
AACATCTCGAAATACAGATCAGCGCCATCCTGCTCCTAAGAAGGCAGAGGATCAAGAACTTGGATCAGCA
GCCAACATCATTGTGAATCCCAAGCTTTTTCAGAAGAGGCGTTTCCAGTCACCTCGTGTTCTGTTCAGTA
CTCAGCCCCCACCGTTGTCAAGAGATGAGCAAAGTGTGGAGTTCCTGGACAATGAAGACGCTCTTAACAG
GAATATCCGGGCCAAACGTGAAAATCATCCTGTGCATAACAGAGGGGAACATTCTGTGTGTGACAGTATC
AGTGTCTGGGTTACCAACAAAACCAAAGCAACGGACATCAAAGGCAATATGGTGACTGTGATGGTGGATG
TAAATCTTAATAATGAAGTCTACAAGCAGTACTTTTTTGAAACCAAGTGCAGAAATCCAAATCCAGTACC
GAGTGGGTGCAGGGGCACTGATTCCAGGCATTGGAATTCTTATTGCACCACAACACAGACATTTGTCAGG
GCATTAACCATGGAAGGCAATCGGGCATCCTGGCGCTTCATTCGGATTGACACTGCCTGTGTGTGCGTAA
TAATTAGAAAAACTGACAACTTCTGA

>GAHG01000020 Hoplocephalus bungaroides NGF-Hop-2 mRNA sequence
ATGTTGTGCTACACTCTGATTATTGCATTTCTGATTGGCATATGGGCAGCACCAAAATCTGAAGATAATG
TCCCTCTGGGCTCCCCTGCAACATCTGACCTTTCTGACACCAGCTGTGCTCAAACTCATGAGGGTCTGAA
AACATCTCGAAATACAGATCAGCACCATCCTGCTCCTAAGAAGGCAGAGGATCAAGAACTTGGATCAGCA
GCCAACATCATTGTGGATCCCAAGCTTTTTCAGAAGAGGCGTTTCCAGTCACCTCGTGTTCTGTTCAGTA
CTCAGCCCCCACCGTTGTCAAGAGATGAGCAAAGTGTGGAGTTCCTGGACAATGAAGACGCTCTTAACAG
GAATATCCGGGCCAAACGTGAAAATCATCCTGTGCATAACAGAGGGGAACATTCTGTGTGTGACAGTATC
AGTGTCTGGGTTACCAACAAAACCAAAGCAACGGACATCAAAGGCAATATGGTGACTGTGATGGTGGATG
TAAATCTTAATAATGAAGTCTACAAGCAGTACTTTTTTGAAACCAAGTGCAGAAATCCAAATCCAGTACC
GAGTGGGTGCAGGGGCACTGATTCCAGGCATTGGAATTCTTATTGCACCACAACACAGACATTTGTCAGG

GCATTAACCATGGAAGGCAATCGGGCATCCTGGCGCTTCATTCGGATTGACACTGCCTGTGTGTGCGTAA
TCATTAGAAAAACTGACAACTTCTGA

>GAHG01000021 Hoplocephalus bungaroides NGF-Hop-3 mRNA sequence
ATGTTGTGCTACACTCTGATTATTGCATTTCTGATTGGCATATGGGCAGCACCAAAATCTGAGGATAATG
TCCCTCTGGGCTCCCCTGCAACATCTGACCTTTCTGACACCAGCTGTGCTCAAATTCACGAGGGTCTGAA
AACATCTCGAAATACAGATCAGCGCCATCCTGCTCCTAAGAAGGCAGAGGATCAAGAACTTGGATCAGCA
GCCAACATCATTGTGGATCCCAAGCTTTTTCAGAAGAGGCGTTTCCAGTCACCCCGTGTTCTGTTCAGCA
CTCAGCCCCCACCGTTGTCAAGAGATGAGCAAAGTGTGGAGTTCCTCGACAATGAAGACGCTCTTAACAG
GAATATCCGGGCCAAACGTGAAAATCATCCTGTGCATAACCAAGGGGAACATTCTGTGTGTGACAGTGTC
AGTGACTGGGTTATCAAAACCACAGCAACGGACATCCACGGCAATATGGTGACTGTGATGGAGGACATAA
CTCTTAATAATGAGGTCTACAAGCAGTACTTTTTTGAAACCAAGTGCAGAAATCCAAATCCAAATCCACT
ACAGAGTGAGTGCAGGGGCATTGATTCCGGGCTTTGGAATTCTTATTGCACCAGAACACAGACATTTGTC
AAGGCATTAACCATGGTAGGCAATCAGGCATCCTGGCGCTTCATTCGTATTGACACTGCCTGTGTGTGCG
TAATCATTAGAAAAACTGACAACTTCTGA

>GAHG01000022 Hoplocephalus bungaroides NGF-Hop-4 mRNA sequence
ATGTTGTGTTACACTCTGATTATTGCATTTCTGATTGGCATATGGGCAGCACCAAAATCTGAGGATAATG
TCCCTCTGGGCTCCCCTGCAACATCTGACCTTTCTGACACCAGCTGTGCTCAAACTCATGAGGGTCTGAA
AACATCTCGAAATACAGATCAGCGCCATCCTGCTCCTAAGAAGGCAGAGGATCAAGAACTTGGATCAGTA
GCAAACATCATTGTGGATCCCAAGCTTTTTCAGAAGAGGCGTTTCCAGTCACCTCGTGTTCTTTTCAGCA
CTCAGCCCCCACCGTTGTCAAGAGATGAGCAAAGTGTGGAGTTCCTCGACAATGAAGACGCTCTTAACAG
GAATATCCGGGCCAAACGTGAAAATCATCCTGTGCATAACCAAGGGGAACATTCTGTGTGTGACAGTGTC
AGTGATTGGGTTATCAAAACCACAGCAACGGACATCCACGGCAATATGGTGAATGTGATGGAGGACATAA
ATCTTAATAATGAGGTCTACAAGCAGTACTTTTTTGAAACCAAGTGCAGAAATCCAAATCCAAATCCACT
ACAGAGTGAGTGCAGGGGCATTGATTCCAGGCTTTGGAATTCTTATTGCACCAGAACACAGACATTTGTC
AAGGCATTAACCATGGCAGGCAATCAGGCATCCTGGCGCTTCATTCGTATTGACACTGCCTGTGTGTGCG
TAATCATTAGAAAAACTGACAACTTCTGA

>GAHG01000023 Hoplocephalus bungaroides PLA2-Hop-6 mRNA sequence
ATGTATCCTGTTCACCTTCTGGTCCTGTTGGCAGTTTGTGTCTCCCTCTTAGGAGCCTCCAACATTCCTC
CGCAGCCTCTCAACCTCTATCAGTTCAAGAACATGATTCCATGTGCCAACCATGGCCGAAGACCTACTTG
GCATTATATGGACTACGGTTGCTACTGCAGCTCAGGAGGTAGCGGGACACCGGTAGATGAGTTGGATAGG
TGCTGCAAAACACATGAGGACTGCTATACTAACATCGGAACATCCAAATGCAACCCCAAGTCGCAGATTT
ATACTTGGCAATGTGGCAGCGATGGACCACACTGCGACGATTCAAAAACAGGATGTGAACGTTCTGTGTG
TGAATGTGATGCCGCAGCAGCCAAGTGCTTTGCCAAAGCCCCTTACAACCAGGAAAACTGGAATATCGAC
ACCAAGAAACGTTGCCAATGA

>GAHG01000024 Hoplocephalus bungaroides PLA2-Hop-7 mRNA sequence
ATGTATCCTGTTCACCTTCTGGTCCTGTTGGCAGTTTGTGTCTCCCTCTTAGGAGCCTCCAACATTCCTC
CGCGGCCTCTCAACCTCTATCAGTTCAAGAACATGATTCCATGTGCCAACCATGGCAGAAGACCTACTTG
GCATTATATGGACTACGGTTGCTACTGCAGCTCAGGAGGTAGCGGGACACCAGTAGATGAGTTGGATAGG
TGCTGCAAAACACATGAGGACTGCTATACTAACATCGGAACATCCAAATGCAACCCCAAGTCGCAGATTT
ATACTTGGCAATGTGGCAGCGATGGACCACACTGCGACGATTCAAAAACAGGATGTGAACGTTCTGTGTG
TGAATGTGATGCCGCAGCAGCCAAGTGCTTTGCCAAAGCCCCTTACAACCAGGAAAACTGGAATATCGAC
ACCAAGAAACGTTGCCAATGA

>GAHG01000025 Hoplocephalus bungaroides PLA2-Hop-8 mRNA sequence
ATGTATCCTGCTCACCTTCTGGTCCTGTTGGCAGTTTGTGTCTCCCTCTTAGGAGCCTCCAACATTCCTC
TGCGGCCTCGCAACCTCATGCAATTCAGCAACATGATTCAATGTGCCAACCATGGCAGTCGACCTACTTC
GCATTATGCGGACTACGGTTGCTACTGCGGCAAAGGAGGCAGCGGGACACCCGTAGATGAGTTGGATAGG
TGCTGCAAAACACATGATGACTGCTATGCTAAAGCCAAAAAATGCTCCCCCAAGCTGTTGTTGTATAGTT
GGCGATGTAGCCGAAATGTACCCATCTGCAAAGATTCAAAATCGGGATGTAAAGGTGTTGTGTGTGATTG
TGACGCCAAAGCAGCCAAGTGCTTTGCCAAAGCCCCTTACAACGACGCGAACTGGGATATCGACACCAAG
ACACGTTGCTAA

>GAHG01000026 Hoplocephalus bungaroides PLA2-Hop-9 mRNA sequence
ATGTATCCTGCTCACCTTCTGGTCCTGTTGGCAGTTTGTGTCTCCCTCTTAGGAGCCTCCAACATTCCTC
TGCGGCCTCGCAACCTCATGCAATTCAGCAACATGATTCAATGTGCCAACCATCGCAGTCGACCTACTTC
GCATTATGCGGACTACGGTTGCTACTGCGGCAAAGGAGGCAGCGGGACACCCGTAGATGAGTTGGATAGG
TGCTGCAAAACACATGATGACTGCTATGCTAAAGCCAAAAAATGCTCCCCCAAGCTGTTGTTGTATAGTT
GGCGATGTAGCCGAAATGTACCCATCTGCAAAGATTCAAAATCGGGATGTAAAGGTGTTGTGTGTGATTG
TGACGCCAAAGCAGCCAAGTGCTTTGCCAAAGCCCCTTACAACGACGCGAACTGGGATATCGACACCAAG
ACACGTTGCTAA

>GAHG01000027 Hoplocephalus bungaroides PLA2-Hop-15 mRNA sequence
ATGTATCCTGCTCACCTTCTGGTCCTGTTGGCAGTTTGTGTCTCCCTCTTAGGAGCCTCCAACATTCCTC
CGCGGCCTCTCAACCTCTATCAGTTCAAGAACATGATTCAATGTGCCAACCGTGGCAGTCGAACTTGGTG
GGCTTATGCGCACTACGGTTGCTACTGCGGCAAAGGAGGCAGCGGGACACCCGTAGATGAGTTGGATAGG
TGCTGCAAAACACATGACGACTGCTATGGTGAAGCCGAAAATCTTCCTGCATGTAATTACGTGATGAGTG
GCTCCTACTACAACATCTATAAATACAAATGTAATGAAGGCGAACTCACCTGCCAAGCGGACAACGATGA
GTGTAAAGCCTTTATCTGTAATTGTGACCGCACAGCAGCCATCTGCTTCGCCGGAGCCCCTTACAACCAG
GAGAAGTTCATGATCTCCAGCAAGACACATTGCCAATGA

>GAHG01000028 Hoplocephalus bungaroides PLA2-Hop-18 mRNA sequence
ATGTATCCTGTTCACCTTCTGGTCCTGTTGGCAGTTTGTGTCTCCCTCTTAGGAGCCTCCAACATTCCTC
CGCAGCCTCTCAACCTTGAACAATTCGACAACATGATTGAATGTGCCAACTATGGCAGTCGACCTTCTTG
GCATTATATGGACTACGGTTGCTACTGCGGCAAAGGAGGTAGCGGGACACCCGTAGATGAGTTGGATAGG
TGCTGCAAAACACATGAGGACTGCTATACTGAAGCCGAAAAACGCGGATGCCACCCCAAGTTCTCGGCAT
ATAGTTGGAAATGTGGCAGCGATGGACCCACTTGCGATGGCAACAATGAGTGTAAAGCCTTTATCTGTAA
TTGTGACCGCACAGCAGCCAAGTGCTTTGCCAAAGCCCCTTTCAACCAGGAAAACTGGGATATCGACACC
GAGACACATTGCCAATGA

>GAHG01000029 Hoplocephalus bungaroides SP-Hop-3 mRNA sequence
ATGTTGATCAGGCTGAACAGTTCTGTTAACTATAGTGAACATATCGCACCTCTTAGCTTGCCTTCCAACC
CTCCCAGGATGGGCTCAGTTTGCCGTGTTATGGGCTGGGGCTCAATCACAACTTCTAATGTGACTTATCC
CGAAGTCCCTCATTGTGTTGACATTAACATACTCCATAATCCGGTGTGTCAAGCAGCTTACCCATCAATG
TCAGGGAAGAACACATTGTGTGCGGGTATCCTGGAAGGAGGCAAAGATTCATGTAAGGGCGATTCTGGGG
GACCCCTCATCTGTAATGGACAAATCCAGGGCATTGTATCTTGGGGGCACTTTCCTTGTGCCCTACTTCT
TGAACCTGGCGTCTACAGCAAGGTCTTCGATTATAAGGACTGGATTGAGGGTATTATTGCAGGAAATTCA
AACGCGATCTGCCCCTAG

>GAHG01000030 Hoplocephalus bungaroides SVMP-Hop-13 mRNA sequence
ATGTCTGCGATAATAAGTAATGAACCTTTTTCTAAGTTCAGCAATTGTAGTGTCCAGGAACATCGGGAGT
ATCTTCTTAGAGACAGACCACAATGCATTCTCAATAATCCCTTGAGCACAGATATTGTTACACCTCCAGT
TTGTGGAAATTACTTAGTGGAGGTGGGAGAAGAATGTGACTGTGGCTCTCCTCAGGATTGTCAAGATGCC
TGCTGCAACGCTGCAACTTGTAAACTGCAACACGACTGTGACTCCGGAGAGTGTTGTGAGAAATGCAAAT
TTAAGAAAGCAGGAGCACAATGCCGGGCAGCAAAGGATGACTGTGACTTGCCTGAAAGCTGCACTGGCCA
ATCTGCTGAGTGTCCCACGGACAGCTTCCAGAGGAATGGACATCCATGCCAAAACAACCAGGGTTACTGC
TACAATGGGAAATGCCCCATCATGACAAACCAGTGTATTGCTCTAAAGGGGCCAGGTGTAAATGTGTCTC
CAGATGAATGTTTTACGTCGAAACAGAATGACCCACGTTGTGGCTTCTGCAGAATAGAAAATGGTAGAAA
GATTCCATGTGCAGAAAAGGATAAAATGTGTGGCAAGTTATTATGCCAAAAGGGAAACGCGATATGCATC
TGCTTTCCTACAACAGATGACCCAGATTATGGAATGGTTGAACCTGGAACAAAATGTGGAGATGGAAAGG
TGTGCATCAACAGGCAGTGTGTTGATGTGCAGACAGCCTACTGA

>GAHG01000031 Hoplocephalus bungaroides SVMP-Hop-14 mRNA sequence
ATGGTCAACACTTTAACTGTGATGTGCAGACCTTTGAATTTTTTCATTGCACTGATTGGCCTAGAAATTT
GGACCAACCAAGATGAGATTGAGATTAAACCAGAGGTGGCTGTCACTTTGAAATCATTTGGAAAATGGAG
AGAAACAGTTTTACTGCCACGCAAAAGGAATGATAATGCTCAGTTACTTACGGGCATTGACTTCAATGGA
ACTACTGTAGGACGTGCTCACGTCGGCTCCCTCTGCAGTCCGAAGAAATCTGTAGCAGTTATTCAGGATC
ATAGCAAAAGAACAAGCATGGTGGCATCTACAATGGCCCATGAGTTGGGTCATAATCTGGGCATTCATCA
TGACAACGCTTCCTGTAATTGCAGTGCTGGACCATGCATTATGTCTGCGAGAGCAAGTCATGAACCTGCC
TATGAGTTCAGCAATTGTAGTGTCCAGGAACATCGGGAGTATCTTCTTAGAGACAGACCACAATGCATTC
TCAACAAACCCTTGAGAAGGGATATTGTTACACCTCCAGTTTGTGGAAATTACTTAGTGGAGCGGGGAGA
AGAATGTGACTGTGGCTCTCCGCAGGATTGTCAAAATGCCTGCTGTAATGCTGCAACTTGTAAACTGCAA
CACGAGGCACAGTGTGAGTCTGGAGTGTGTTGTGAGAAATGCAAATTTAAGAAAGCAGGAGCAGAATGCC
GGGCAGCAAAGGACGACTGTGACTTGCCTGAAAGCTGCACTGGCCAATCTGCTAAGTGTCCCACGGACAG
CTTCCAGAGGAATGGACATCCATGCCAAAACAACCAGGGTTACTGCTACAATCGGAAATGCCCCCTCATG
ACAAACCAGTGTATTGCTCTCGGGGGACCAGGTGTAAATGTGTCTCCAGATAGATGTTTTACGATCAACC
AGAGGGGCAGAGGTTGTGGCTTCTGCAGAATAGAAAATGGTACAAAGATTCCATGTGCAGCAAAGGATAA
AATGTGTGGCATGTTATACTGCGAAAAGGGAAACACGACATGCACCTGCTTTACTACAACAGATGACCCA
GATTATGGAATGGTTGATCCTGGAACAAAATGTGGAAATGGAAAGGTGTGCATCAACAGGCAGTGTGTTG
ATGTGCAGACAGCCTACTGA

>GAHG01000032 Hoplocephalus bungaroides SVMP-Hop-15 mRNA sequence
ATGAGTTGGGTCGATAATCTGGGCATTCATCATGACAACGCTTCCTGTAATTGCAGTACTGGACCATGCA
TTATGTCTGCGATAATAAGTGATGAACCTCTCTATGAGTTCAGCTCCTGTAGTGTCCAGGAACATCAGAG
GTATCTTCTTAGACACAGACCACAATGCAGTCTCAACAAACCCTTGAGCACAGATATTGTTGCACCTCCA
GTTTGTGGAAATTACTTTGTGGAGGTGGGAGAAGAATGTGATTGCGGCTCTCCTGCGGATTGTCAAAGTG
CCTGCTGCAATGCTGCAACTTGTAAACTGCAACATGAGGCACAGTGTGACTCCGGAGAGTGTTGTGAGAA
ATGCAAATTTAAGAAAGCAGGAGCAGAATGCCGGGCAGCAAAGGATGACTGTGACTCACCTGAAAGCTGC
ACTGGCCAATCTGCTGAGTGTCCCACGGACAGCTTCCAGAGGAATGGACATCCATGCCAAAACAACCAAG
GTTACTGCTACAATGGGAAATGTCCCATCATGACAAACCAATGTATTGATCTCTGGGGGCCAGGTGTAAA
AGTGTCTTCAGGTATATGTTTTACATTGAACCAGAATGGCCGAAGTTGTGGCTTCTGCAGAATAGAACAT
GGTACAAAGATTCCATGTGCAGCAAAGGATGTAAAGTGTGGCACGTTATTTTGCAAAAAGAGAAACTCGA
TGATATGCAGCTGCAAAGTTTCATCACATGACCCAAGTYRTGGAATGGTTGAACCTGG

>GAHG01000033 Hoplocephalus bungaroides SVMP-Hop-18 mRNA sequence
ATGATCAACACTTTAAATATGATGTACAGATCTTTGAATTTTTTCATTGCACTGATTGGCCTAGAAATTT
GGACCAACAAAGATAAGATTAAGATTGAACAAGAGGCGAATGTCACTTTGAAATCATTTGGAGAATGGAG
AGAAACAGTTTTGCTGCCACGCAAAAGGAATGATAATGCTCAGTTACTTACGGGCATTGACTTCAACGGA
AGGACTCTAGGACTTGCTTACGTCGGCTCCCTCTGCCTTCCGATGCACTCTGTAGCAGTTATTCAGAATT
ATAGCAGAGACACAAGAATAATGGCATCTATAATGGCCCATGAGTTGGGTCATAATCTGGGCATTAATCA
TGACAACTCTTCCTGTAATTGCAGTGCTAGACCATGCATTATGTCTAAGACAGTAAGTCATGAACCTCTC
TATGACTTCAGCAATTGTAGTGTCCAGGAACATCAGAGGTATCTTCTTAGAGTCAGACCACAATGCATTC
TCAACAAACCCTTGAGCACAGATATTGTTACACCTCCAGTTTGTGGAAATTACTTAGTGGAGAGGGGAGA
AGAATGTGATTGTGGCTCTCCTCAGGATTGTCAAGATGCCTGCTGCAACGCTGCAACTTGTAAACTGCAA
CACGACTGTGACTCCGGAGAGTGTTGTGAGCAATGCAAATTTAAGAAAGCAGGAGCACAATGCCGGGCAG
CAAAGGACGACTGTGACTTGCCTGAAAGCTGCACTGGCCAATCTGCTGAGTGTCCCACGGACAGCTTCCA
GAGGAATGGACATCCATGCCAAAGCAACCAGGGTTACTGCTACAATGGGAAATGCCCCATCATGACAAAC
CAGTGTATTGCTCTAATGGGGCCAGGTGTAAATGTGTCTCCAGATGATTGTTTTACGTGGAAACAGAATG
ACCCAGATTGTGGCTTCTGCAGAATAGAAAATGGTAGAAAGATTCCATGTGCAGAAAAGGATAAAATGTG
TGGCATGTTAGTATGCGAAAAGAGAAACTCGAAATGCACCTGCTTTACTACAACAGATGACCCAGATTAT
GCAATGGTTGATCCWGGGCTTGATCAG

>GAHG01000034 Hoplocephalus bungaroides SVMP-Hop-23 mRNA sequence
ATGGCATCTGAAATGGCCCATGAGTTGGGTCATAATCTGGGCATTAGTCATGACAACGCTTCCTGTAATT
GCAATGCTGGACCATGCATTATGTCTGCGATAATAAGTTTTGAACCTCTCTCTGAGTTCAGCTCTTGTAG
TATCCAGGAACATCAGAGGTATCTTCTTAGAGACAGACCACAATGCATTCTCAACAAACCCTTGAGCACA
GATATTGTTACACCTCCAGTTTGTGGAAATTACTTAGTGGAGGTGGGAGAAGAATGTGACTGTGGTTCTC
CTGTGGATTGTCAAAGTGCCTGCTGCAATGCTACAACTTGTAAACTGCAACATGAGGCACAGTGTGACTC
CGGAGAGTGTTGTGAGCAATGCAAATTTAAGAAAGCAGGAGCAGAATGCCGGGCAGCAAAGGATGACTGT
GACTTGCCTGAAAGCTGCACTGGCCAATCTGCTGAGTGTCCCACAGACAGCTTCCAGAGGAATGGACATC
CATGCCAAAACAACCAGGGTTACTGCTACAATGGGAAATGCCCCATCATGACAAACCAATGTATTGATCT
CTGGGGGCCAGGTGTAAATGTGTCTCCAGATGAATGTTTTACGTGGAACCAGAATGGCCAAGGTTGTGGC
TTCTGCAGAATGGAAAATGGTGCAAAGATTCCATGTGCAGCAAAGGATGTAAAGTGTGGCAAKTTACACT
GCGAAAAGGGACACATGATATGCATCTGCTTTGTTTCACCAGATGACCCAGATTATGGAATGGCTGAACC
TGG

>GAHG01000035 Hoplocephalus bungaroides SVMP-Hop-30 mRNA sequence
ATGTCTAAGACAGTAAGTCATGAACCTGCCTATGAGTTCAGCAATTGTAGTGTCCAGGAACATCAGAGGT
ATCTTCTTAGTAACAGACCACAATGCATTCTCAACAAACCCTTGAGCACAGATATTGTTACACCTCCAGT
TTGTGGAAATTACTTAGTGGAGAGGGGAGAAGAATGTGATTGTGGCTCTCCTCAGGATTGTCAAGATGCC
TGCTGCAATGCGGCAACTTGTAAACTGCAACACGACTGTGACTCCGGAGAGTGTTGTGAGCAATGCAAAT
TTAAGAAAGCAGGAGCAGAATGCCGGGCAGCAAAGGATGACTGTGACTTGCCTGAAAGCTGCACTGGCCA
ATCTGCTAAGTGTCCCACGAACCGCTTCCAGAGGAATGGACATCCATGCCAAAACAACCAGGGTTACTGC
TACAATGGGAAATGCCCCATCATGACAAACCAGTGTATTGCTCTCAGGGGACCAGGTGTAAATGTGTCTC
CAGATGGATGTTTTAAGTTCAACCAGGCTGGCCAAAGTTGTGGCTTCTGCAGAATAGAAAATGGTAGAAA
GATTCCATGTGCAGCAAAGGATGTAAAGTGTGGCACGTTATACTGCAAAGAGGGAAACGCGACATGCCGC
TGCTTTCCTACAACACATGACCCATATTATAGAATGGTTGAACCTGGAACAAAATGTGGAGATGGAAAGG
TGTGCATCAACAGGCAGTGTGTTGATGTGCAGACAGCCTACTGA

>GAHG01000036 Hoplocephalus bungaroides SVMP-Hop-36 mRNA sequence
ATGCACTCTGTAGCAGTTATTCAGAATTATAGCAGAGACACAAGAATAATGGCATCTATAATGGCCCATG
AGTTGGGTCATAATCTGGGCATTAATCATGACAACTCTTCCTGTAATTGCAGTGCTAGACCATGCATTAT
GTCTAAGACAGTAAGTGATGAACCTGCCTATGAGTTCAGCAATTGTAGTGTCCAGGAACATCAGAGGTAT
CTTCTTAGTAACAGACCACAATGCATTCTCAACAAACCCTTGAGCACAGATATTGTTACACCTCCAGTTT
GTGGAAATTACTTAGTGGAGAGGGGAGAAGAATGTGATTGTGGCTCTCCTCAGGATTGTCAAGATGCCTG
CTGCAATGCTACAACTTGTAAACTGCAACACGACTGTGACTCCGGAGAGTGTTGTGAGCAATGCAAATTT
AAGAAAGCAGGAGCAGAATGCCGGGCAGCAAAGGATGACTGTGACTTGCCTGAAAGCTGCACTGGCCAAT
CTGCTGAGTGTCCCACGGACAGCTTCCAGAGGAATGGACATCCATGCCAAAACAACCAGGGTTACTGTTA
CAATCGGACATGCCCCCTCATGACAAACCAGTGTATTGCTCTCGGGGGACCAGGTGTAAATGTGTCTCCA
GATGAATGTTTTATGATCAACAAGAAGGGCAAAGGTTGTGGCTTCTGCAGAATAGAAAATGGTAGAAAGA
TTCCATGTGCAGCAAAGGATGTAAAGTGTGGCAAGTTATACTGCAAAGAGGGAAACAATAAATGCCTCTG
CTATGAATCACAAGATGACCCAGATACTGGAATGGTTGAACCTGGAACAAAATGTGGAGATGGAAAGGTG
TGCATCAACAGGCAGTGTGTTGATGTGCAGACAGCCTACTGA

>GAHG01000037 Hoplocephalus bungaroides SVMP-Hop-39 mRNA sequence
ATGCATTATGTCTGCGACATAAGTCATGAACCTCTCTATGACTTCAGCAATTGTAGTGTCCAGGAACATC
AGAGGTATCTTCTTAGAGTCAGACCACAATGCATTCTCAACAAACCCTTGAGCACAGATATTGTTACACC
TCCAGTTTGTGGAAATTACTTAGTGGAGGTGGGAGAAGAATGTGACTGTGGCTCTCCTCAGGATTGTCAA
GATGCCTGCTGCAACGCTGCAACTTGTAAACTGCAACACGACTGTGACTCCGGAGAGTGTTGTGAGCAAT
GCAAATTTAAGAAAGCAGGAGCAGAATGCCGGGCAGCAAAGGATGACTGTGACTTGCCTGAAAGCTGCAC
TGGCCAATCTGCTGAGTGTCCCACGGACAGCTTCCAGAGGAATGGACATCCATGCCAAAACAACCAGGGT
TACTGCTACAATGGGAAATGCCCCATCATGACAAACCAATGTATTGATCTCTGGGGGCCAGGTATAAATG
TGTCTCCAGATGAATGTTTTACGTGGAACCAGAATGGCCAAGGTTGTGGCTTCTGCAGAATGGAACATGG
TAGAAAGATTCCATGTGCAGCAAAGGATATAAAGTGTGGCAAGTTACACTGCAAAAAGGGAAACGCGACA
TGCATCTGCTTTGTTTCACCAGATGACCCAGATTATGGAATGGCTGAACCTGGAACAAAATGTGGAGATG
GAATGGTGTGCATCAACAGGCAGTGTGTTGATGTGCAGACAGCCTACTGA

>GAHG01000038 Hoplocephalus bungaroides SVMP-Hop-45 mRNA sequence
ATGGCCCCCATGAGTTGGGTCATAATCTGGGCATTCATCATGACAACGCTTCCCTGTAATTGCAGTGCTG
GACAATGCATTATGTCTGAGACAATAAGTGATGAACCTTTCTATGAGTTCAGCAATTGTAGTGTCCAGGA
ACATCAGAGGTATCTTCTTAGTAACAGACCACAATGCATTCTCAACAAACCCATGAGAAAGAATATTGTT
ACACCTCCAGTTTGTGGAAATTACTTAGTGGAGCGGGGAGAAGAATGTGACTGTGGCTCTCCTCAGGATT
GTCAAAGTGCCTGCTGCAATGCTACAACTTGTAAACTGCAACACGAGGCACAGTGTGACTCCGGAGAGTG
TTGTGAGAAATGCAAATTTAAGAAAGCAGGAACAGAATGCCGGGCAGCAAAGGATGACTGTGACTTGCCT
GAAAGCTGCACTGGCCAATCTGCTAAGTGTCCCACGAACCGCTTCCAGAGGAATGGACATCCATGCCAAA
ACAATGAGGGTTACTGCTACAATGGGAAATGCCCCATCATGACAAACCAGTGTATTGCTCTCGGGGGACC
AGGTGTAAATGTGTCTCCAGATGGATGTTTTAAGTTCAACCAGGCTGGCCAAGATTGTGGCTTCTGCAGA
ATGGAAAATGGTAGAATGATTCCGTGTGCAGCAAACGATGAAAAGTGTGGCAAGTTATACTGCATAGTCG
GAAACAATACATGCCTCTGCTATGGTTCACGAGATGACCCAGATACTGGAATGGTTGAACCTGGAACAAA
ATGTGGAGATGGAAAGGTGTGCATCAACAGGCAGTGTGTTGATGTGCAGACAGCCTACTGA

>GAHG01000039 Hoplocephalus bungaroides SVMP-Hop-46 mRNA sequence
ATGCCCCCTGATGGCAGAGAAATTACAACAAGCCCTCCGGTTCAGGATCACTGCTATTATCATGGCTACA
TTCAGAATGAAGCTGATTCACGTGCAGTCATCAGTGCATGCAACGGCTTGAAAGGACATTTCAAGCATCG
AGGGGAGACATACTTTATTGAGCCCTTGAAGCTTTCCGACAGTGAATCCCATGCAATCTACAAAGATGAA
AATGTAGAAAAAGAGGATGAGATCCCCAAAATCTGTGGGGTAACCCATACTACTTCGGAGTCAGATGAGC
CCATCGAAAAGATCTCTCAATTAGCTAATACTCCTGAACAAGGCAGGTACTTGCAGGTCAAAAAAATATAT
TGAGTTTTACGTGGTTTTGGACAACAAAATGTACAAGAATTACAACAGCAACAGAGGTGCTATAAAAACA
AGAGTATATGAAATGATCAACACTTTAAATATGATGTACAGATCTTTGAATTTTTTCATTGCACTGATTG
GCCTAGAAATTTGGACCAACAAAGATAAGATTAAGATTGAACAAGAGGCGAATGTCACTTTGAAATCATT
TGGAGAATGGAGAGAAACAGTTTTGCTGCCACGCAAAAGGAATGATAATGCTCAGTTACTTACGGGCATT
GACTTCAACGGAGAAACTGTAGGACTTGCTTACGTGGGCACCCCTCTGCAGTCCGAAGTTCTCTGTAGCGG
TTATTCAGAATTATAGCAGAGACACAAGAATGATGGCATCTATAATGGCCCATGAGTTGGGTCATAATCT
GGGCATTAATCATGACAACTCTTCCTGTAATTGCAGTGCTAGACCATGCATTATGTCTGAAAGATTAAGT
CATGAACCTGCCTATGAGTTCAGCAATTGTAGTGTCCAGGAACATCGGAAGTATCTTCTTAGAGACAGAC
CACAATGCATTCTCAACAAACCCTTGAGCACAGATATTGTTACACCTCCAGCTTGTGGAAATTACTTAGT
GGAGAGGGGAGAAGAATGTGATTGTGGCTCTCCTCAGGATTGTCAAGATGCCTGCTGCAACGCTGCAACT
TGTAAACTGCAACACGACTGTGACTCCGGAGAGTGTTGTGAGAAATGCAAATTTAAGAAAGCAGGAGCAG
AATGCCGGGCAGCAAAGGATGACTGTGACTTGCCTGAAAGCTGCACTGGCCAATCTGCTAAGTGTCCCAC
GAACCGCTTCCAGAGGAATGGACATCCATGCCAAAACAACCAGGGTTACTGCTACAATGGGAAATGCCCC
ATCATGACAAACCAGTGTATTGCTCTCAGGGGACCAGGTGTAAATGTGTCTCCAGATGGATGTTTTAAGT
TCAACCAGGCTGGCCAAAGTTGTGGCTTCTGCAGAATAGAAAATGGTAGAAAGATTCCATGTGCAGCAAA
GGATGTAAAGTGTGGCACGTTATACTGCAAA

>GAHG01000040 Hoplocephalus bungaroides SVMP-Hop-50 mRNA sequence
ATGCACTCTGTAGCAGTTATTCAGAATTATAGCAGAGACACAAGAATAATGGCATCTATAATGGCCCATG
AGTTGGGTCATAATCTGGGCATTAATCATGACAACTCTTCCTGTAATTGCAGTGCTAGACCATGCATTAT
GTCTAAGACAGTAAGTGATGAACCTGCCTATGAGTTCAGCAATTGTAGTGTCCAGGAACATCAGAGGTAT
CTTCTTAGTAACAGACCACAATGCATTCTCAACAAACCCTTGAGCACAGATATTGTTACACCTCCAGTTT
GTGGAAATTACTTAGTGGAGAGGGGAGAAGAATGTGATTGTGGCTCTCCTCAGGATTGTCAAGATGCCTG
CTGCAACGCTACAACTTGTAAACTGCAACACGACTGTGACTCCGGAGAGTGTTGTGAGCAATGCAAATTT
AAGAAAGCAGGAGCACAATGCCGGGCAGCAAAGGATGACTGTGACTTGCCTGAAAGCTGCACTGGCCAAT
CTGCAGAGTGTCCCACGGACAGCTTCCAGAGGAATGGACATCCATGCCAAAACAACCAGGGTTACTGCTA
CAATCGGAAATGCCCCATCATGACAAACCAGTGTATTGCTCTAGGGGGGCCAGGTGTAAATGTGTCCCCA
GATGGATGTTTTAAGTTCAACCAGGATGGCCAAGATTGTGGCTTCTGCAGAATGGAAAATGGTAGAATGA
TTCCGTGTGCAGCAAAGGATGTAAAGTGTGGCAAGATATTCTGCAAAGAGGGAAACGATACATGCATCTG
CTATGGTTCACCAGGTGACCCAGATAGAGGAATGGTTGAACCTGGAACAAAATGTGGAGATGGAAAGGTG
TGCATCAACAGGCAGTGTGTTGATGTGCAGACAGCCTACTGA

>GAHG01000041 Hoplocephalus bungaroides SVMP-Hop-62 mRNA sequence
ATGATGGCATCTATAATGGCCCATGAGTTGGGTCATAATCTGGGCATTAAACATGACAACTCTTCCTGTA
ATTGCAGTGCTAGACCATGCATTATGTCTGAAAGATTAAGTCATGAACCTGCCTATGAGTTCAGCAATTG
TAGTGTCCAGGAACATCGGGAGTATCTTCTTAGAGACAGACCACAATGCATTCTCAACAAACCCTTGAGC
ACAGATATTGTTACACCTCCAGTTTGTGGAAATTACTTAGTGGAGAGGGGAGAAGAATGTGATTGTGGCT
CTCCTCAGGATTGTCAAGATGCCTGCTGCAACGCTGCAACTTGTAAACTGCAACACGACTGTGACTCCGG
AGAGTGTTGTGAGAAATGCAAATTTAAGAAAGCAGGAGCAGAATGCCGGGCAGCAAAGGATGACTGTGAC
TTGCCTGAAAGCTGCACTGGCCAATCTGCTGAGTGTCCCACGGACAGCTTCCAGAGGAATGGACATCCAT
GCCAAAACAACCAGGGTTACTGCTACAATCGGAAATGCCCCATCATGACAAACCAGTGTATTGCTCTAGG
GGGGCCAGGTGTAAATGTGTCCCCAGATGGATGTTTTAAGTTCAACCAGGATGGCCAAGATTGTGGCTTC
TGCAGAATGAAAAATGGTAGAATGATTCCGTGTGCAGCAAAGGATGTAAAGTGTGGCAAGATATTCTGCA

AAGAGGGAAACGATACATGCAACTGCTATGGTTCACCAGATGACCCAGATTATGGAATGGTTGAACCTGG
AACAAAATGTGGAGATGGAAAGGTGTGCATCAACAGGCAGTGTGTTGATGTGCAGACAGCGTACTGA

>GAHG01000042 Hoplocephalus bungaroides SVMP-Hop-63 mRNA sequence
ATGGCATCTATAATGGCCCATGAGTTGGGTCATAATCTGGGCATTAATCATGACAACTCTTCCTGTAATT
GCAGTGCTAGACCATGCATTATGTCTAAGACAGTAAGTGATGAACCTGCCTATGAGTTCAGCAATTGTAG
TGTCCAGGAACATCAGAGGTATCTTCTTAGTAACAGACCACAATGCATTCTCAACAAACCCTTGAGCACA
GATATTGTTACACCTCCAGTTTGTGGAAATTACTTAGTGGAGAGGGGAGAAGAATGTGATTGTGGCTCTC
CTCAAGATTGTCAAGATGCCTGCTGCAACGCTACAACTTGTAAACTGCAACACGACTGTGACTCCGGAGA
GTGTTGTGAGCAATGCAAATTTAAGAAAGCAGGAGCAGAATGCCGGGCAGCAAAGGATGACTGTGACTTG
CCTGAAAGCTGCACTGGCCAATCTGCTGAGTGTCCCACGGACAGCTTCCAGAGGAATGGACATCCATGCC
AAAACAACCAGGGTTACTGCTACAATCGGAAATGCCCCATCATGACAAACCAGTGTATTGCTCTAGGGGG
GCCAGGTGTAAATGTGTCCCCAGATGGATGTTTTAAGTTCAACCAGGATGGCCAAGATTGTGGCTTCTGC
AGAATGAAAAATGGTAGAATGATTCCGTGTGCAGCAAAGGATGTAAAGTGTGGCAAGATATTCTGCAAAG
AGGGAAACGATACATGCATCTGCTATGGTTCACCAGGTGACCCAGATAGAGGAATGGTTGAACCTGGAAC
AAAATGTGGAAATGGAAAGGTGTGCATCAACAGGCAGTGTGTTGATGTGCAGACAGCCTACTGA

>GAHG01000043 Hoplocephalus bungaroides SVMP-Hop-65 mRNA sequence
ATGTACAGGAATTACAACAGCACGAAACATGCTATAAAAGAAAAGTATATGAAATAGTCAACCATTTAA
ACATGATGTACAGACCTTTGAATTTTCTCATTGCACTGATTGGCCTAGAAATTTGGTCCAACCGAGATAA
GATTAACATTGAACCAGAGATGGCTGTCACTTTGAAATCATTTGGAAAACGGAGAGAAACAGTTTTACTG
CCACGCAAAAGGAATGATAATGCTCAGTTACTTACGGGCATTGAGTTCAGCGGAACTACTGTAGGACTTG
GTTACGTGGGCACCCTCTGCAGTCCGGAGGAATCTGTAGCAGTTATTGAGGTTTATAGCAGAAGAACAAG
TATAATGGCATCTGAAATGGCCCATGAGTTGGGTCATAATCTGGGCATTAGTCATGACAACGCTTCCTGT
AATTGCAATGCTGGACCATGCATTATGTCTGCGATAATAAGTTTTGAACCTCTCTCTGAGTTCAGCTCTT
GTAGTATCCAGGAACATCAGAGGTATCTTCTTAGAGACAGACCACAATGCATTCTCAACAAACCCTTGAG
CACAGATATTGTTACACCTCCAGTTTGTGGAAATTACTTAGTGGAGGTGGGAGAAGAATGTGACTGTGGT
TCTCCTGAGGATTGTCAAAGTGCCTGCTGCAATGCTACAACTTGTAAACTGCAACATGAGGCACAGTGTG
ACTCCGGAGAGTGTTGTGAGCAATGCAAA

>GAHG01000044 Hoplocephalus bungaroides VEGF-Hop-4 mRNA sequence
ATGGTGCATAGCGTAATGTCCATGTTGTGCTACACTCTGATTATTGCATTTCTGATTGGCATATGGGCAG
CACCAAAATCTGAAGATAATGTCCCTCTGGGCTCCCCTGCAACATCTGACCTTTCTGACACCAGCTGTGC
TCAAACTCATGAGGGTCTGAAAACATCTCGAAATACAGATCAGCACCATCCTGCTCCTAAGAAGGCAGAG
GATCAAGAACTTGGATCAGCAGCCAACATCATTGTGGATCCCAAGCTTTTTCAGAAGAGGCGTTTCCAGT
CACCTCGTGTTCTGTTCAGTACTCAGCCCCCACCGTTGTCAAGAGATGAGCAAAGTGTGGAGTTCCTGGA
CAATGAAGACGCTCTTAACAGGAATATCCGGGCCAAACGTGAAAATCATCCTGTGCATAACAGAGGGGAA
CATTCTGTGTGTGACAGTATCAGTGTCTGGGTTACCAACAAAACCAAAGCAACGGACATCAAAGGCAATA
TGGTGACTGTGATGGTGGATGTAAATCTTAATAATGAAGTCTACAAGCAGTACTTTTTTGAAACCAAGTG
CAGAAATCCAAATCCAGTACCGAGTGGGTGCAGGGGCACTGATTCCAGGCATTGGAATTCTTATTGCACC
ACAACACAGACATTTGTCAGGGCATTAACCATGGAAGGCAATCGGGCATCCTGGCGCTTCATTCGGATTG
ACACTGCCTGTGTGTGCGTAATCATTAGAAAAACTGACAACTTCTGA

>GAHG01000045 Hoplocephalus bungaroides VEGF-Hop-5 mRNA sequence
ATGATGCATAGCGTAATGTCCATGTTGTGCTACACTCTGATTATTGCATTTCTGATTGGCATATGGGCAG
CACCAAAATCTGAGGATAATGTCCCTCTGGGCTCCCCTGCAACATCTGACCTTTCTGACACCAGCTGTGC
TCAAATTCACGAGGGTCTGAAAACATCTCGAAATACAGATCAGCGCCATCCTGCTCCTAAGAAGGCAGAG
GATCAAGAACTTGGATCAGCAGCCAACATCATTGTGGATCCCAAGCTTTTTCAGAAGAGGCGTTTCCAGT
CACCCCGTGTTCTGTTCAGCACTCAGCCCCCACCGTTGTCAAGAGATGAGCAAAGTGTGGAGTTCCTCGA
CAATGAAGACGCTCTTAACAGGAATATCCGGGCCAAACGTGAAAATCATCCTGTGCATAACCAAGGGGAA
CATTCTGTGTGTGACAGTGTCAGTGACTGGGTTATCAAAACCACAGCAACGGACATCCACGGCAATATGG
TGACTGTGATGGAGGACATAACTCTTAATAATGAGGTCTACAAGCAGTACTTTTTTGAAACCAAGTGCAG
AAATCCAAATCCAAATCCACTACAGAGTGAGTGCAGGGGCATTGATTCCGGGCTTTGGAATTCTTATTGC
ACCAGAACACAGACATTTGTCAAGGCATTAACCATGGTAGGCAATCAGGCATCCTGGCGCTTCATTCGTA
TTGACACTGCCTGTGTGTGCGTAATCATTAGAAAAACTGACAACTTCTGA

>GAHG01000046 Hoplocephalus bungaroides VEGF-Hop-6 mRNA sequence
ATGATGCATAGTGTAATGTCCATGTTGTGTTACACTCTGATTATTGCATTTCTGATTGGCATATGGGCAG
CACCAAAATCTGAGGATAATGTCCCTCTGGGCTCCCCTGCAACATCTGACCTTTCTGACACCAGCTGTGC
TCAAACTCATGAGGGTCTGAAAACATCTCGAAATACAGATCAGCGCCATCCTGCTCCTAAGAAGGCAGAG
GATCAAGAACTTGGATCAGTAGCAAACATCATTGTGGATCCCAAGCTTTTTCAGAAGAGGCGTTTCCAGT
CACCTCGTGTTCTTTTCAGCACTCAGCCCCCACCGTTGTCAAGAGATGAGCAAAGTGTGGAGTTCCTCGA
CAATGAAGACGCTCTTAACAGGAATATCCGGGCCAAACGTGAAAATCATCCTGTGCATAACCAAGGGGAA
CATTCTGTGTGTGACAGTGTCAGTGATTGGGTTATCAAAACCACAGCAACGGACATCCACGGCAATATGG
TGAATGTGATGGAGGACATAAATCTTAATAATGAGGTCTACAAGCAGTACTTTTTTGAAACCAAGTGCAG
AAATCCAAATCCAAATCCACTACAGAGTGAGTGCAGGGGCATTGATTCCAGGCTTTGGAATTCTTATTGC
ACCAGAACACAGACATTTGTCAAGGCATTAACCATGGCAGGCAATCAGGCATCCTGGCGCTTCATTCGTA
TTGACACTGCCTGTGTGTGCGTAATCATTAGAAAAACTGACAACTTCTGA

>GAHH00000000.1|GAHH01000000 TSA: Pseudonaja modesta, transcriptome shotgun assembly, transcriptome shotgun assembly

>GAHH01000001 Pseudonaja modesta 3FTx-Pse-229 mRNA sequence
ATGAAAACTCTACTGCTGACCTTGGTGATGGTGACAATCGTGTGCCTGGACTTAGGATACACCCTAACAT
GTTACAAATCTCTGAGTGGTACTGTGGTTTGTAAACCACACGAGACGAAATGCTATCGATATTCTCTTCC
TGCAACTCATGGTAATGCGGTAACCATCAAGGGATGTGGTACTTCTTGCCCTAGTGGAATCCGCCCTGTG
TGTTGCAGTACGGACCTATGCAACAAGTAG

>GAHH01000002 Pseudonaja modesta 3FTx-Pse-243 mRNA sequence
ATGAAAACTCTACTGCTGACCTTGGTGATGGTGACAATCATGTGCCTGGACTTAGGATACACCCTAACAT
GTTACAAATCTCTGAGTGGTACTGTGGTTTGTAAACCACACGAGACGAAATGCTATCGATATTCTCTTCC
TGCAACTCATGGTAATGCGGTAACCATCAAGGGATGTGGTACTTCTTGCCCTAGTGGAATCCGCCCTGTG
TGTTGCAGTACGGACCTATGCAACAAGTAG

>GAHH01000003 Pseudonaja modesta 3FTx-Pse-242 mRNA sequence
ATGAAAACTCTACTGCTGACCTTGGTGATGGTGACAATCATGTGCCTGGACTTAGGATACACCCTAACAT
GTTACAAATCTCTGAGTGGTACTGTGGTTTGTAAACCACACGAGACGAAATGCTATCGATATTCTCTTCC
TGCAACTCATGGTAATGCGGTAACCATCAAGGGATGTGGTACTTCTTGCCCTACTGGAATCCGCCCTGTG
TGTTGCAGTACGGACCTATGCAACAAGTAG

>GAHH01000004 Pseudonaja modesta 3FTx-Pse-241 mRNA sequence
ATGAAAACTCTACTGCTGACCTTAGTGATGGTGACAATCATGTGCCTGGACTTAGGATACACCCTAACAT
GTTACAAATCTCTGAGTGGTACTGTGGTTTGTAAACCACACGAGACGAAATGCTTTCGATATTCTGTTCC
TGCAAGTCATGGTAATGCAATGATCATCAAGGGATGTGGTACTTCTTGCCCTAGTGGAATCCGCCCTGTG
TGTTGCAGTACGGACCTATGCAACAAGTAG

>GAHH01000005 Pseudonaja modesta 3FTx-Pse-245 mRNA sequence
ATGAAAACTCTACTGCTGACCTTGGTGATGGTGACAATCATGTGCCTGGACTTAGGATACACCCTAACAT
GTTACAAATCTCTGAGTGGTACTGTGGTTTGTAAACCACACGAGACGAAATGCTTTCGATATTCTGTTCC
TGCAAGTCATGGTAATGCAATGATCATCAAGGGATGTGGTACTTCTTGCCCTAGTGGAATCCGCCCTGTG
TGTTGCAGTACGGACCTATGCAACAAGTAG

>GAHH01000006 Pseudonaja modesta 3FTx-Pse-215 mRNA sequence
ATGAAAACTCTACTGCTGACCTTGGTGATGGTGACAATCATGTGCCTGGACTTAGGATACACCCTAACAT
GTTACAAGTTTCTGAGGGGTACTGTGGTTTGTAAACCACACGAGACGAAATGCTATCGATATTCTGTTCC
TGCAACTCATGGTAATGCGGTAACCTTCAGGGGATGTGGTACTTCTTGCCATAGTGGAATCCACCCTGTC
TGTTGCAGTACGGACCTATGCAACAAGTAG

>GAHH01000007 Pseudonaja modesta 3FTx-Pse-178 mRNA sequence
ATGAAAACTCTACTGCTGACCTTGGTGATGGTGACAATCATGTGCCTGGACTTAGGATACACCCTAACAT
GTTACAAGAGTTTTTCTTATACTGTGGTTTGTAAACCACACGAGACGAAATGCTATCGATATTCTCTTCC
TGCAAGTCATGGTAATGCGGTAACCATCTGGGGATGTGGTACTTCTTGCCCTACTGGAATCCGCCCTGTG
TGTTGCAGTACGGACCTATGCAACAAGTACTCTACAAGTGGCTAA

>GAHH01000008 Pseudonaja modesta 3FTx-Pse-188 mRNA sequence
ATGAAAACTCTACTGCTGACCTTGGTGATGGTGACAATCATGTGCCTGGACTTAGGATACACCCTAACAT
GTTACAAGAGTTTTTCTTATACTGTGGTTTGTAAACCACACGAGACGAAATGCTATCGATATTCTCTTCC
TGCAAGTCATGGTAATGCGGTAACCATCATGGGATGTGGTACTTCTTGCCCTAGTGGAATCCGCCCTGTG
TGTTGCAGTACGGACCTATGCAACAGGTAG

>GAHH01000009 Pseudonaja modesta 3FTx-Pse-211 mRNA sequence
ATGAAAACTCTACTGCTGACCTTGGTGATGGTGACAATCATGTGCCTGGACTTAGGATACACCCTAAAAT
GTTACAAGAGTTATCGTGATACTGTGGTTTGTCAACCACACGAGACGAAATGCTATCGATATTCTGTTCC
TGCAACTCATGGTAATGCGGTAACCATCAGGGGATGTGGTACTTCTTGCCATAGTGGAATCCACCCTGTC
TGTTGCAGTACGGACCTATGCAACAATTAG

>GAHH01000010 Pseudonaja modesta 3FTx-Pse-217 mRNA sequence
ATGAAAACTCTACTGCTGACCTTGGTGATGGTGACAATCATGTGCCTGGACTTAGGATACACCCTAAAAT
GTTACGAGAGTTTTTTTCATACTGTGGTTTGTAAACCACAGGAGACGAAATGCTATCGATATTCTGTTCC
TGCAAGTCATGGTAATGCGGTAACCATCAGGGGATGTGGTACTTCTTGCCCTGGGGGAATCCACCCTGTG
TGTTGCAGTACGGACCTGTGCAACCGGTAG

>GAHH01000011 Pseudonaja modesta 3FTx-Pse-271 mRNA sequence
ATGAAAACTCTGCTGCTGACCTTGGTGGTGGTGACAAGCCTGTGCCTGGACTTAGGATACACCCTAAAAT
GTTACGAGAGTTTTTTTCATACTGTGGTTTGTAAACCACAGGAGACGAAATGCTATCGATATTCTGTTCC
TGCAAGTCATGGTAATGCGGTAACCATCAGGGGATGTGGTACTTCTTGCCCTGGGGGAATCCGCCCTGTG
TGTTGCAGTACGGACCTGTGCAACCGGTAG

>GAHH01000012 Pseudonaja modesta 3FTx-Pse-256 mRNA sequence
ATGAAAACTCTACTGCTGACCTTGGTGATGGTGACAATCATGTGCCTGGACTTAGGATACACCCTAACAT
GTTACGAGAGTTTTTTTCATACTGTGGTTTGTAAACCACAGGAGACGAAATGCTATCGATATTCTGTTCC
TGCAAGTCATGGTAATGCGGTAACCATCAGGGGATGTGGTACTTCTTGCCCTGGGGGAATCCGCCCTGTG
TGTTGCAGTACGGACCTGTGCAACCGGTAG

>GAHH01000013 Pseudonaja modesta 3FTx-Pse-267 mRNA sequence
ATGAAAACTCTACTGCTGACCTTAGTGATGGTGACAATCATGTGCCTGGACTTAGGATACACCCTAACAT
GTTACGAGAGTTTTTTCTATACTGTGGTTTGTAAACCACAGGAGACGAAATGCTATCGATATTCTGTTCC
TGCAAGTCATGGTAATGCGGTAACCATCAGGGGATGTGGTACTTCTTGCCCTGGGGGAATCCGCCCTGTG
TGTTGCAGTACGGACCTGTGCAACCGGTAG

>GAHH01000014 Pseudonaja modesta 3FTx-Pse-268 mRNA sequence
ATGAAAACTCTACTGCTGACCTTGGTGATGGTGACAATCATGTGCCTGGACTTAGGATACACCCTAAAAT
GTTACGAGAGTTTTTTCTATACTGTGGTTTGTAAACCACAGGAGACGAAATGCTATCGATATTCTGTTCC
TGCAAGTCATGGTAATGCGGTAACCATCAGGGGATGTGGTACTTCTTGCCCTGGGGGAATCCGCCCTGTG
TGTTGCAGTACGGACCTGTGCAACCGGTAG

>GAHH01000015 Pseudonaja modesta 3FTx-Pse-273 mRNA sequence
ATGAAAACTCTACTGCTGACCTTGGTGATGGTGACAATCATGTGCCTGGACTTAGGATACACCCTAACAT
GTTACACGAGTTTTCATGGTACTGTGGTTTGTCAACCACACGAGACGAAATGCTATCGATATATTGCTCC
TGGAACTCATGGTCATGCGTTAACCATCAGGGGATGTGATACTTATTGCCCTGGGGGAATGCGCCCTGTG
TGTTGCAGTACGGACCTGTGCAACTGGTAG

>GAHH01000016 Pseudonaja modesta 3FTx-Pse-179 mRNA sequence
ATGAAAACTCTACTGCTGACCTTGGTGATGGTGACAATCATGTGCCTGGACTTAGGATACACCCTAAAAT
GTTACAAGGGTTATTATGATACTGTGGTTTGTAAACCAAACGAGACCATTTGCTATGAATATCATATTTC
TGCAACTCATGGTAATTCGATACTCTACAGGGGATGTAGTACTTCTTGCCCTGGGGGAATGCGCTCTGTG
TGTTGCAGTACGGACCTGTGCAACAAGTAG

>GAHH01000017 Pseudonaja modesta 3FTx-Pse-180 mRNA sequence
ATGAAAACTCTACTGCTGACCTTGGTGATGGTGACAATCATGTGCCTGGACTTAGGATACACCCTAAAAT
GTTACAAGGGTTATCATGATACTGTGGTTTGTAAACCAGACGAGACCATTTGCTATGAATATCATATTTC
TGCAACTCATGGTAATTCGATACTCTACAGGGGATGTAGTACTTCTTGCCCTGGGGGAATGCGCTCTGTG
TGTTGCAGTACGGACCTGTGCAACAAGTAG

>GAHH01000018 Pseudonaja modesta 3FTx-Pse-191 mRNA sequence
ATGAAAACTCTACTGCTGACCTTGGTGATGGTGACAATCATGTGCCTGGACTTAGGATACACCCTAAAAT
GTTACAAGGGTTATCATGATACTGTGGTTTGTAAACCACACCAGACCATTTGCTATGAATATCGTATTGC
TGCAACTCATGGTAATTCGATACTCTACAGGGGATGTAGTGCTTCTTGCCGTGGGGGAATGCGCTCTGTG
TGTTGCAGTACGGACCTGTGCAACAAGTAG

>GAHH01000019 Pseudonaja modesta 3FTx-Pse-183 mRNA sequence

ATGAAAACTCTACTGCTGACCTTGGTGATGGTGACAATCATGTGCCTGGACTTAGGATACACCCTAACAT
GTTACAAGGGTTATCATGGTACTGTGGTTTGTAAACCACACGAGACCATTTGCTATCGATATCTTATTCC
TGCATTTCATAGTAATGCGATACTCGCCAGAGGATGTAGTACTTCTTGCCCTGGGGGAATCCGCCCTGTG
TGTTGCAGTACGGACCTGTGCAACAAGTAG

>GAHH01000020 Pseudonaja modesta 3FTx-Pse-196 mRNA sequence
ATGAAAACACTACTGCTGACCTTGGTGATGGTGACAATCATGTGCCTGGACTTAGGATACACCCTAACAT
GTTACAAGGGTTATCATGGTACTGTGGTTTGTAAACCACACGAGACCATTTGCTATCGATATCTTATTCC
TGCATTTCATAGTAATGCGATACTCGCCAGAGGATGTAGTACTTCTTGCCCTGGGGGAATCCGCCCTGTG
TGTTGCAGTACGGACCTGTGCAACAAGTAG

>GAHH01000021 Pseudonaja modesta 3FTx-Pse-200 mRNA sequence
ATGAAAACTCTACTGCTGACCTTGGTGATGGTGACAATCATGTGCCTGGACTTAGGATACACCCTAAAAT
GTTACAAGAGTTATCATGGTACTGTGGTTTGTCAAACACACGAGACCATTTGCTATCGATATGTTATTCC
TACAAGTCATGGTAATGCGAACATTTACAAAGGATGTAGTGCTTCTTGCCCTGGGGGAATCGGCTCTGTG
TGTTGCAGTACGGACCTGTGCAACAAGTAG

>GAHH01000022 Pseudonaja modesta 3FTx-Pse-263 mRNA sequence
ATGAAAACTCTACTGCTGACCTTGGTGATGGTGACAATCATGTGCCTGGACTTAGGATACACCCTAACAT
GTTACAAGGATTATCGTGATACTGTGGTTTGTAAATCACACGAGACCATTTGCTATCGATATAATATTCC
TACAAGTCATGGTAATGCGATAACCTACAGGGGATGTAGTGCTTCTTGCCCTGGGGGAATCCGCTCTGTG
TGTTGCAGTACGGACCTGTGCAACAAGTAG

>GAHH01000023 Pseudonaja modesta 3FTx-Pse-275 mRNA sequence
ATGAAAACTCTACTGCTGACCTTGGTGATGGTGACAATCATGTGCCTGGACTTAGGATACACCCTAACAT
GTTACAAGGATTATCATAATACTGTGGTTTGTAAACCACACGAGACCATTTGCTATGTATATACTCTTCC
TGCAACTCATGGTAATGTGATAACCTCCATGGGATGTGGTACTTCTTGCCCTGGGGGAATCCGCCCTGTG
TGTTGCAGTAAGGACCTGTGCAACAAGTAG

>GAHH01000024 Pseudonaja modesta 3FTx-Pse-272 mRNA sequence
ATGAAAACTCTACTGCTGACCTTGGTGATGGTGACAATCATGTGCCTGGACTTAGGATACACCCTAACAT
GTTACAAGGATTATCGTAATACTGTGGTTTGTAAACCACACGAGACCATTTGCTATGAATATACTCTTCC
TGCAACTCATGGTAATGTGATGAACTCCAGGGGATGTGGTACTTCTTGCCCTGGGGGAATCCGCCCTGTG
TGTTGCAGTACGGACCTGTGCAACAAGTAG

>GAHH01000025 Pseudonaja modesta 3FTx-Pse-204 mRNA sequence
ATGAAAACTTTGCTGCTGACCTTGGTGGTGGTGACAATCATGTGCCTGGACTTAGGATACACCCTAACAT
GTTACAAGAGTTTTTCTGATACTGTGGTTTGTAAACCACACGAGACGAAATGCTTTCAATATTCTTTTTG
TCCATTTCGAAATCGTGTGATATACGTCAGGGGATGTAGTACTTCTTGCCGTGGGGGAAACAATGCTGTG
TGTTGCAGTACGGACCTGTGCAACAAGTAG

>GAHH01000026 Pseudonaja modesta 3FTx-Pse-206 mRNA sequence
ATGAAAACTTTGCTGCTGACCTTGGTGGTGGTGACAATCGTGTGCCTGGACTTTGGATACACCCTAACAT
GTTACAAGAGTTTTTCTGATACTGTGGTTTGTAAACCACACGAGACGAAATGCTTTCAATATTCTTTTTG
TCCATTTCGAAATCGTGTGATATACGTCAGGGGATGTAGTACTTCTTGCCCTGGGGGAAACAATGCTGTG
TGTTGCAGTACGGACCTGTGCAACAAGTAG

>GAHH01000027 Pseudonaja modesta 3FTx-Pse-207 mRNA sequence
ATGAAAACTTTGCTGCTGACCTTGGTGGTGGTGACAATCGTGTGCCTGGACTTAGGATACACCCTAACAT
GTTACAAGAGTTTTTCTGATACTGTGGTTTGTAAACCACACGAGACGAAATGCTTTCAATATTCTTTTTG
TCCATTTCGAAATCGTGTGATATACGTCAGGGGATGTAGTACTTCTTGCCCTGGGGGAAACAATGCTGTG
TGTTGCAGTACGGACCTGTGCAACAAGTAG

>GAHH01000028 Pseudonaja modesta 3FTx-Pse-239 mRNA sequence
ATGAAAACTTTGCTGCTGACCTTGGTGGTGGTGACAATCGTGTGCCTGGACTTAGGATACACCCTAACAT
GTTACAAGTCTCTGAGTGGTACTGTGGTTTGTAAACCACACGAGACGAAATGCTATCGATATTCTTTTTG
TCCATTTCCAAATCGTGTGATATACAGAAGGGGATGTGGTACTTCTTGCCCTGGGGGAAACAATCCTGTG
TGTTGCAGTACGGACCTGTGCAACAAGTAG

>GAHH01000029 Pseudonaja modesta 3FTx-Pse-240 mRNA sequence
ATGAAAACTTTGCTGCTGACCTTGGTGGTGGTGACAATCGTGTGCCTGGACTTAGGATACACCCTAACAT
GTTACAAGTCTCTGAGTGGTACTGTGGTTTGTAAACCACACGAGACAAAATGCTATCGATATTCTTTTTG
TCCATTTCCAAATCGTGTGATATACAGAAGGGGATGTGGTACTTCTTGCCCTGGGGGAAACAATCCTGTG
TGTTGCAGTACAGACCTGTGCAACAAGTAG

>GAHH01000030 Pseudonaja modesta 3FTx-Pse-288 mRNA sequence
ATGAAAACTTTGCTGCTGACCTTGGTGGTGGTGACAATCGTGTGCCTGGACTTAGGATACACCCTAACAT
GTTACAAGTCTCCGTTTGGTACTGTGGCTTGCAACAAATATGAGACCTTCTGCTATCGATTTACTCATTG
TCCATTTCCACATCATGTGGTAGTCATCAGGGGATGTATTACTTCTTGCCCTACGCAAAACAATTATGTG
TGTTGCAGTACGGACCTGTGCAACAAGTAG

>GAHH01000031 Pseudonaja modesta 3FTx-Pse-290 mRNA sequence
ATGAAAACTCTACTGCTGACCTTGGTGATGGTGACAATCATGTGCCTGGACTTAGGATACACCCTAACAT
GTTACAAGTCTCCGTTTGGTACTGTGGCTTGCAACAAATATGAGACCTTCTGCTATCGATTTACTCATTG
TCCATTTCCACATCATGTGGTAGTCATCAGGGGATGTATTACTTCTTGCCCTACGCAAAACAATTATGTG
TGTTGCAGTACGGACCTGTGCAACAAGTAG

>GAHH01000032 Pseudonaja modesta 3FTx-Pse-287 mRNA sequence
ATGAAAACTCTACTGCTGACCTTGGTGATGGTGACAATCATGTGCCTGGACTTAGGATACACCCTAACAT
GTTACAAGTCTCCGTTTGGTACTGTGGCTTGCAACAAATATGAGACCTTCTGCTATCGATTTACTCATTG
TCCATTTCCACATCATGTGGTAGTCATCAGGGGATGTAGAGTTTCTTGCCCTACGCAAAACAATTATGTG
TGTTGCAGTACGGACCTGTGCAACAAGTAG

>GAHH01000033 Pseudonaja modesta 3FTx-Pse-197 mRNA sequence
ATGAAAACTCTACTGCTGACCTTGGTGATGGTGACAATCATGTGCCTGGACTTAGGATACACCCTAACAT
GTTACACGAGTTATTATAATACTAAGGTTTGTCCACGACACGAGACCAGTTGCTATCGACGTGTTTTTCA
TGCAGGTCATGGTAATGTGATAGACCTCTGGGGATGTCGTACTTCTTGCCCTTGGGAAAACCCCCCTGAA
TGTTGCAGTACGGACCTGTGCAACCGGTAG

>GAHH01000034 Pseudonaja modesta 3FTx-Pse-23 mRNA sequence
ATGAAAACTCTGCTGCTGACCTTGGTGGTGGTGACAATCGTGTGCCTGGACTTAGGTGACAGCTTGATAT

GCTACCTGGATTTTAGTGTACCTCACACTTGTGCACCTGGGGAGGAGCTATGCTATACAAGGACTTGGAA
TGATGGCCGTGGAACGCGAATCGAACGGGGATGTGCTGCTACTTGCCCTATTCCAAAGAACCCCGAGATA
CATGTTACCTGTTGCTCAACAGACAGGTGCAACCCACATCCAAAAGAGAAACCTCGTTGA

>GAHH01000035 Pseudonaja modesta 3FTx-Pse-38 mRNA sequence
ATGAAAACTCTGCTGCTGACCTTGGTGGTGGTGACAATCGTGTGCCTGGACTTAGGTGACAGCTTGATAT
GCTACCTGGATTTTAGTGTACCTCACACTTGTGCACCTGGGGAGGAGCTATGCTATACAAGGACTTGGAA
TGATGGCCGTGGAACGCGAATCGAACGGGGATGTGCTGCTACTTGCCCTATTCCAAAGAGGCCCGGTATA
CATGTTACCTGTTGCTCAACAGACAGGTGCAACCCACATCCAAAAGAGAAACCTCGTTGA

>GAHH01000036 Pseudonaja modesta 3FTx-Pse-15 mRNA sequence
ATGAAAACTCTGCTGCTGACCTTGGTGGTGGTGACAATCGTGTGCCTGGACTTAGGTGACAGCTTGATAT
GCTACCTGGATTTTAGTGTACCTCACACTTGTGCACCTGGGGAGGAGCTATGCTATACAAAGACTTGGAG
TGATGGCCGTGGAACGCGAATCGAACGGGGATGTGCTGCTACTTGCCCTATTCCAAAGAGGCCCGGTATA
CATGTTACCTGTTGCTCAACAGACAGGTGCAACCCACATCCGAAACAGAAACCTCATTGA

>GAHH01000037 Pseudonaja modesta 3FTx-Pse-24 mRNA sequence
ATGAAAACTCTGCTGCTGACCTTGGTGGTGGTGACAATCGTGTGCCTGGACTTAGGTGACAGCTTGATAT
GCTACCTGGATTTTAGTGTACCTCACACTTGTGCACCTGGGGAGGAGCTATGCTATACAAAGACTTGGTA
TGATGGCCGTGGAACGCGAATCGAACGGGGATGTGCTGCTACTTGCCCTATTCCAAAGAGGCCCGGTATA
CATGTTACCTGTTGCTCAACAGACAGGTGCAACCCACATCCGAAACAGAAACCTCATTGA

>GAHH01000038 Pseudonaja modesta 3FTx-Pse-39 mRNA sequence
ATGAAAACTCTGCTGCTGACCTTGGTGGTGGTGACAATCGTGTGCCTGGACTTAGGTGACAGCTTGATAT
GCTACCTGGATTTTAGTGTACCTCACACTTGTGCACCTGGGGAGGAGCTATGCTATACAGAGACTTGGTA
TGATGGCCGTGGAACGCGAATCGAACGGGGATGTGCTGCTACTTGCCCTATTCCAAAGAGGCCCGGTATA
CATGTTACCTGTTGCTCAACAGACAGGTGCAACCCACATCCGAAACAGAAACCTCATTGA

>GAHH01000039 Pseudonaja modesta 3FTx-Pse-89 mRNA sequence
ATGAAAACTCTGCTGCTGACCTTGGTGGTGGTGACAATCGTGTGCCTGGACTTAGGATACACCAGGACAT
GCTACAAAACACCTGCACCTGTTAGACCTGGGCCTTGTCCACCTGGGCAGGAGATATGCTACACAAAGAC
ATGGTGTGATGAGTGGTGTGGCATCAGAGGAAAGGTAATCGAACTGGGATGTGCTGCTACTTGCCCTACT
CCAGAGAAGACCAGTATACAAGTTACCTGTTGCTCAACAGACAGGTGCAACCCACATCCGAAACAGAAAC
CTCATTGA

>GAHH01000040 Pseudonaja modesta 3FTx-Pse-110 mRNA sequence
ATGAAAACTCTGCTGCTGACCTTGGTGGTGGTGACAATCATGTGCCTGGACTTAGGATACACCAGGACAT
GCTTGACAACACCTGGTTTTTATCTAAGCCTTGTCCATCTGGGCAGGAGGTATGCTACACAAAGGCATG
GTGTAATGAGTGGTGTCCAGACAGAGGAAAGCAAGTCGAAATGGGATGTGCTGCTATTTGCCCTTCAGTG
AAGCCCAAAGAGGATATTACCTGTTGCTCAGAAGACAATTGCAACCCACGTCCATAA

>GAHH01000041 Pseudonaja modesta 3FTx-Pse-99 mRNA sequence
ATGAAAACTCTGCTGCTGACCTTGGTGGTGGTGACAATCGTGTGCCTGGACTTAGGATACACCAGGACAT
GCTTCAAAACACCTGCACCTGTTAGATCTGAGCCTTGTCCACCTGGGGAGGACCTATGCTTTACAAGGAC
TTGGAGAGATGCCCATAGTGGAAACAGAGGACCGCGAGTCGATTTGGGATGTGCTGCTACTTGCCCCACT
ACAGATAAGACCGAGATACGTATTTCCTGTTGCTCAGAAGACGATTGCAACACATTTCCAAAATGGGAAC
GTCCTAAACCACGTCCTTGA

>GAHH01000042 Pseudonaja modesta 3FTx-Pse-116 mRNA sequence
ATGAAAACTCTGCTGCTGACCTTGGTGGTGGTGACAATCGTGTGCCTGGACTTAGGATACACCAGGACAT
GCTTCAAAACACCTGCACCTGTTAGATCTGGGCCTTGTCCACCTGGGGAGGACCTATGCTTTACAAGGAC
TTGGAGAGATGGCCATAGTGGAAACAGAGGACCGCGAGTCGATTTGGGATGTGCTGCTACTTGCCCCACT
ACAGATAAGACCGAGATACATATTTCCTGTTGCTCAGAAGACGATTGCAACACATTTCCGCAATGGGAAC
GTCCTAAACCACGTCCTCGAGGGAGGCTCTCATCCATCAAGGACCATGCTTGA

>GAHH01000043 Pseudonaja modesta 3FTx-Pse-121 mRNA sequence
ATGAAAACTCTGCTGCTGACCTTGGTGGTGGTGACAATCGTGTGCCTGGACTTAGGATACACCAGGACAT
GCTTCAAAACACCTGCACCTGTTAGATCTGAGCCTTGTCCACCTGGGGAGGACCTATGCTTTACAAGGAC
TTGGAGAGATGGCCATGGTGGAAGAGGACCGCGAGTCGATTTGGGATGTGCTGCTACTTGCCCCACTACA
GATAAGACCGGGATACATATTTCCTGTTGCTCAACAGACGATTGCAACACATTTCCGAAATGGGAACGTC
CTAAATAA

>GAHH01000044 Pseudonaja modesta 3FTx-Pse-93 mRNA sequence
ATGAAAACTCTGCTGCTGACCTTGGTGGTGGTGACAATCGTGTGCCTGGACTTAGGATACACCAGGACAT
GCTTCAAAACACCTGCACCTGTTAGATCTGAGCCTTGTCCACCTGGGGAGGAGGTATGCTATACAAGGAC
TTGGAGAGATGGCCATGGTGGAAGAGGACCGCGAGTCGATTTGGGATGTGCTGCTACTTGCCCCACTACA
GATAAAACCGGGATACATATTTCCTGTTGCTCAACAGACGATTGCAACACATTTCCGAAATGGGAACGTC
CTAAATAA

>GAHH01000045 Pseudonaja modesta 3FTx-Pse-105 mRNA sequence
ATGAAAACTCTGCTGCTGACCTTGGTGGTGGTGACAATCGTGTGCCTGGACTTAGGATACACCAGGACAT
GCTTCAAAACACCTGATGTTAGATCTGAGCCTTGTCCACCTGGGGAGGACCTATGCTATACAAAGAGTTG
GTGTGGTGAGTTGTGTTACAGCAGAAGAAATCGAGTCGATTTGGGATGTGCTGCTACTTGCCCTACTCCA
GAGAATACCGGTATAGAGATTATCTGTTGCTCAACAGACAATTGCAACCCATTTCCGAAATGGGAACATC
CTAAACCACGTTCTCGAGGGAGGCTCTCATCCATCAAGGACCATCCTTGA

>GAHH01000046 Pseudonaja modesta 3FTx-Pse-78 mRNA sequence
ATGAAAACTCTGCTGCTGACCTTGGTGGTGGTGACAATCATGTGCCTGGACTTAGGATACACCAGGACAT
GCTTCAAAACACCTTATGTTAAATCTGAGCCTTGTCCACCTGGGCAGGAGGTATGCTACACAAAGACTTG
GTGTGATTCTTTTTGTTCCCTCAGAGGAAAGGTAGTCGAACTGGGATGTGCTGCTACTTGCCCTCCAGCG
GGGCCAAAAGAGGATGTTACCTGTTGCTCAACAGACAATTGCAACACACATCCGTAA

>GAHH01000047 Pseudonaja modesta CRiSP-Pse-4 mRNA sequence
ATGATTGCCTTCATTGTCTTGCTGAGTCTTGCTGCAGTGCTGCAACAGTCTTCTGGAACTGTTGATTTTG
CTTCTGAGTCAAGTAACGAAAATGATTACCAAAGGGAGATTGTTGACAAGCACAATGATTTAAGAAGATC
AGTGAAACCAACTGCTAGGAACATGTTACAAATGAAATGGAATTCTCTTGCTGCTCAAAATGCAAAACGT
TGGGCAAATAGATGTACTTATGCTCACAGTCCACCACACACAAGAACTGTGGGAAAACTCCGTTGTGGTG
AAAATATATTCATGTCAAGTCAACCTTTTCCATGGAGTGGAATAGTTCAGGCTTGGTATGATGAAGTCAA
AAAATTTGTCTATGGCATTGGACCAAACCCACCAAATTCTGTTACTGGCCATTATACCCAGGTAGTTTGG
TACAAAAGTCACCTTCTTGGTTGTGCTTCTGCCAAATGTTCTCCAACCAAATACCTCTACGTTTGTCAAT
ACTGCCCAGCAGGGAACATCGAAGGTTCAATTGCTACTCCATATAAATCAGGACCACCTTGTGGGGACTG

TCCTTCGGCTTGTGACAACGGACTATGCACAAATCCTTGCAAACATAACGATGACTTTTCGAACTGCAAA
ACTTTAGTGCAACAACATAAATGCCAGAGTGAATGGATCAAGTCAAAATGCCCTGCTACTTGCTTCTGCC
ACACTGAAATAATATAG

>GAHH01000048 Pseudonaja modesta CRiSP-Pse-11 mRNA sequence
ATGATTGCCTTCATTGTCTTGCTGAGTCTTGCTGCAGTGCTGCAACAGTCTTCTGGAACTGTTGATTTTG
CTTCTGAGTCAAGTAACAAAACTAATTACCAAAAGGAGATTGTTGACAAGCACAATGATTTAAGGAGATC
AGTGAAACCAACTGCTAGGAACATGTTACAAATGAAATGGAATTCTCTTGCTGCTAAAAATGCAAAACGT
TGGGCAAATAGATGTACTTTTGCTCACAGTCCACCATACACAAGAACTGTGGGAAAACTCCGTTGTGGTG
AAAATATATTCATGTCAAGTCAACCTTTTGCATGGAGTGGAGTAGTTCAGGCTTGGTATGATGAAGTCAA
AAAATTTGTCTATGGCATTGGAGCAAAGCCACCAAGTTCTGTTACTGGCCATTATACCCAGGTAGTTTGG
TACAAAAGTCACCTTCTTGGTTGTGCTTCTGCCAAATGTTCTCCAACCAAATACCTCTACGTTTGTCAAT
ACTGCCCAGCAGGGAACATCAAAGGTTCAATTGCTACTCCATATAAATCAGGACCACCTTGTGGGGACTG
TCCTTCGGCTTGTGACAACGGACTATGCACAAATCCTTGCAAACATAACGATGACTTATCGAACTGCAAA
ACTTTAGTGAAAAAACATAAATGCCAGACTGAATGGATCAAGTCAAAATGCCCTGCTACTTGCTTCTGCC
GCACTGAAATAATATAG

>GAHH01000049 Pseudonaja modesta CRiSP-Pse-17 mRNA sequence
ATGATTGCCTTCATTGTCTTGCTGAGTCTTGCTGCAGTGCTGCAACAGTCTTCTGGAACTGTTGATTTTG
CTTCTGAGTCAAGTAACGAAAATGATTACCAAAGGGAGATTGTTGACAAGCACAATGATTTAAGAAGATC
AGTGAAACCAACTGCTAGGAACATGTTACAAATGAAATGGAATTCTCTTGCTGCTCAAAATGCAAAACGT
TGGGCAAATAGATGTACTTATGCTCACAGTCCACCACACACAAGAACTGTGGGAAAACTCCGTTGTGGTG
AAAATATATTCATGTCAAGTCAACCTTTTCCATGGAGTGGAATAGTTCAGGCTTGGTATGATGAAGTCAA
AAAATTTGTCTATGGCATTGGACCAAACCCACCAAGTTCTGTTACTGGCCATTATACCCAGGTAGTTTGG
TACAAAAGTCACCTTCTTGGTTGTGCTTCTGCCAAATGTTCTCCAACCAAATACCTCTACGTTTGTCAAT
ACTGCCCAGCAGGGAACATCGAAGGTTCAATTGCTACTCCATATAAATCAGGACCACCTTGTGGGGACTG
TCCTTCGGCTTGTGACAACGGACTATGCACAAATCCTTGCAAACATAACGATGACTTTTCGAACTGCAAA
ACTTTAGTGCAACAACATAAATGCCAGAGTGAATGGATCAAGTCAAAATGCCCTGCTACTTGCTTCTGCC
ACACTGAAATAATATAG

>GAHH01000050 Pseudonaja modesta KP-Pse-5 mRNA sequence
ATGTCTTCTGGAGGTCTTCTTCTCCTGCTGGGACTCCTCACCCTCTGGGAGGTGCTGACCCCCGTCTCCA
GCAAGGACCGTCCAAATTTCTGTGAACTGCCTGCTGAAACCGGACGATGTAATGCCCATATCACACGCTT
CTACTACAACCCACGTCAACATCAATGCATAAAGTTTCTTTATGGTGGATGCGGAGGGAATGCTAACAAT
TTTAAGACCATTAAGGAATGCAAAAGCACCTGTATTGCCTGA

>GAHH01000051 Pseudonaja modesta KP-Pse-7 mRNA sequence
ATGTCTTCTGGAGGTCTTCTTCTCCTGCTGGGACTCCTCACCCTCTGGGAAGTGCTGACCCCCGTCTCCA
GCAAGGACCGTCCAAATTTCTGTGAACTGCCTGCTGAAACCGGACTATGTAATGCCCATAACACACGCTT
CTACTACAACCCACGTCAACATCAATGCATAAAGTTTCTTTATGGTGGATGCGGAGGGAATGCTAACAAT
TTTAAGACCATAGAGGAATGCAAAAGCACCTGTATTGCCTGA

>GAHH01000052 Pseudonaja modesta LP-Pse-1 mRNA sequence
ATGGGGCGATTCCTCTTGGTGACCCTCAGCTTGCTGGTCGGGGCTTTCTCCCTAAATGGAGCCAACAGCT
GCTGTTGTCCCCAAGATTGGCTCCCCATGAATGGGTTTTGCTACAAGGTCTTCAATGATCCCAAGAACTG
GGACGACGCTGAGATGTTCTGCAGGAAATTAAAGCCAGGTTGCCACCTCGCATCCATCCATAGTAATGCA
GATGCAGTTGAGCTTTCTGAATACATCACCGACTATCTCATAGGTCATGACCACGTCTGGATCGGACTGC
GGGATACAAACAAGAAGTACATGTGGGAGTGGACAGACAGATCTAGAACTGATTTCCTGCTCTGGAGGAA
AGATCAGCCCGATCATTCCACTAACAATGAGTTCTGTGTTGAGATTGTGAGCTTTACAGGGTATCGCGAG
TGGAATGACGACAGCTGCACAGCCTTGCGTCCTTTTCTGTGCCAGTGCAAACACTAG

>GAHH01000053 Pseudonaja modesta LP-Pse-2 mRNA sequence
ATGGGGCGATTCCTCTTGGTGACCCTCAGCTTGCTGGTCGGGGCTTTCTTCCTAAATGAAGCCAACAGCT
GCTGTTGCCCCCAAGATTGGCTCCCCATGAATGGGCTTTGCTACAAGGTCTTCAATGACCACAAGAACTG
GAACGATGCTGAGATGTTCTGCAGGAAATTAAAGCCAGGTTGCCACCTCGCCTCCATCCATAGTAATGCA
GATGCAGTTGAGCTTTCTGAATACATCACCGACTATCTCATAGGTCATGACCACGTCTGGATCGGACTGC
GGGATACAAACAAGAAGTACATGTGGGAGTGGACAGACAGATCTAGAACTGATTTCCTGCTCTGGAGGAA
AGATCAGCCCGATCATTCCAGTAACAATGAGTTCTGTGTCGAGATTGTGAGCTTTACAGGGTATCGCGAG
TGGAATGACGACAGCTGCACAGCCTTGCGTCCTTTTCTGTGCCAGTGCAAACACTAG

>GAHH01000054 Pseudonaja modesta LP-Pse-3 mRNA sequence
ATGGGGCGATTCCTCTTGGCGACCCTCAGCTTGCTGGTCGGGGCTTTCTTCCTAAATGGAGCCAACAGCT
GCTGTTGTCCCCAAGATTGGCTCCCCATGAATGGGCTTTGCTACAAGGTCTTCAATGACCACAAGAACTG
GGACGACGCTGAGATGTTCTGCAGGAAATTAAAGCCAGGTTGCCACCTCGCCTCCATCCATAGTAATGCA
GACTCAGCTGACTTGGCTGAATACGTCTCCGACTATCTCATAGGTCATGACCACGTCTGGATCGGACTGC
GGGATACAAGGAAGAAGTACATCTGGGAGTGGACAGACAGATCTAGAACTGATTTCCTGCTCTGGAGGAA
AGATCAGCCCGATCATTATAAAAACAATGAATTCTGTGTCGAGATTGTGAGCTTTACAGGGTATCGCGAG
TGGAATGACGACAGCTGCACAGCCTTGCGTCCTTTTCTGTGCCAGTGCAAACACTAG

>GAHH01000055 Pseudonaja modesta LP-Pse-5 mRNA sequence
ATGGGGCGATTCCTCTTGGTGACCCTCAGCTTGCTGGTCGGGGCTTTCTCCCTAAATKGAGCCAACAGCT
GCTGTTGTCCCCAAGATTGGCTCCCCATGAATGGGTTTTGCTACAAGGTCTTCAATGACCACAAGAACTG
GAACGATTCTGAGATGTTCTGCAGGAAATTAAAGCCAGGTTGCCACCTCGCATCCATCCATAGTAATGCA
GATGCAGTTGAGCTTTCTGAATACATCACCGACTATCTCATAGGTCATGACCACGTCTGGATCGGACTGC
GGGATACAAACAAGAAGTACATGTGGGAGTGGACAGACAGATCTAGAACTGATTTCCTGCTCTGGAGGAA
AGATCAGCCCGATCATTCCACTAACAATGAGTTCTGTGTTGAGATTGTGAGCTTTACAGGGTATCGCGAG
TGGAATGACGACAGCTGCACAGCCTTGCGTCCTTTTCTGTGCCAGTGCAAACACTAG

>GAHH01000056 Pseudonaja modesta LP-Pse-6 mRNA sequence
ATGGGGCGATTCCTCTTGGCGACCCTCAGCTTGCTGGTCGGGGCTTTCTCCCTATGCGGAGCCACCAGCT
CCTGTTGTCCCCAAGATTGGCTCCCCAGGAATGGGCTTTGCTACAAGGTCTTCAATGACCACAAGAACTG
GAACGACGCTGAGATGTTCTGCAGGAAATTAAAGCCAGGTTGCCACCTCGCCTCCATCCATAGTAATGCA
GACTCAGCTGACTTGGCCGAATACGTCTCCGACTATCTCATAGGTCATGACCACGTCTGGATCGGACTGC
GGGATACAAGGAAGAAGTACATCTGGGAGTGGACAGACAGATCTAGAACTGATTTCCTGCTCTGGAGGAA
AGATCAGCCCGATCATTATAAAAACAATGAATTCTGTGTCGAGATTGTGAGCTTTACAGGGTATCGCGAG
TGGAATGACGACAGCTGCACAGCCTTGCGTCCTTTTCTGTGCCAGTGCAAACACTAG

>GAHH01000057 Pseudonaja modesta LP-Pse-7 mRNA sequence
ATGGGGCGATTCCTCTTTGCGAGCCTCGGCTTGCTGGTCCTGGCTTTCTCCCTCAGTGGAATTAGAGCCA
ATCGCTATTGTCCCTTTGATTGGTTCTCCTACAATATGTCTTGCTACAAGCTCTTCTACTCTCCGATGTC
CTGGGACCAAGCACAGAGGTTCTGTGTGGAACAGCAGGAAAACTCCCAACTCGCCTCCATCAACGACGTG

GCAGAGTCGGTGAAGTTGTCCAACTACATCTCCCAAAGATTGACATTCCTTGATGTTTGGATGGGACTGA
GGCTTTCCAAGAGAAAAGGCATCTGGGAGTGGAGTGATGGCTCCAACCTCACTCACACATCCTGGAAAGA
AGGTGAACCCAACAACCTTTTCAACAAGGAATTCTGTGCTGTGCTGTCAGCCGGAACACGGTATCTCCAA
TGGAACGATAAGAAGTGCACACACCTGCATCCATTTGTCTGCAAGTTCCAGCCCCGGAGTGAGGCAGGAG
GCTAA

>GAHH01000058 Pseudonaja modesta LP-Pse-9 mRNA sequence
ATGGGGCGATTCCTCTTGGTGAGCCTGAGCTTGCTGGTTGTGGCTTTRTCCYTAAATGGAATTGGAGCTG
ACCATCATTGCCCGTCGGACTGGACCTCCCAGGGGGAGTTTTGCTACAAGTTGATCAGAGAATTGAAGAC
CTGGGAAGACGCCGAGGCATCCTGCGTGCAACGTCAGAACAGCAGCCACCTGGCCTCCATCCACAACCCA
GAAGAGTCCGGTTACATCCAAACGGTGATCTTCAGGAGTATTGTCTACAGTAATGTCTGGATTGGACTGA
ATGATCCAGGGAAAAATCGCATGTGGGAATGGAGTGATGGCTCCAATGTGGATTATACGTCCTGGAAACG
ATCGGAACCCGGCAACGCTGATRGTGGGGAGTACTGCGTTCAGGTGACCGCTATCACAGGTCATTATAGA
TGGCAAGCTGACAAGTGTGAATCCAAGAATTTTTTCGTCTGCAAGATGTAG

>GAHH01000059 Pseudonaja modesta NP-Pse-1 mRNA sequence
ATGGTCGGCCTCTCCCGTCTGGCGGGCGGCGGGCTGCTGCTGCTGGCCCTGCTGCCTCTCGCCCTCGACG
GGAAGCCGGCGCCGCTGCCTCAGGCGCTGCCCGAGGCTCCGGCGGGCGGCGTGATGGCATCGGTGACGGA
GGAGCAGCAGCGGCTGCCGGTGGCGGCGGAGGAGTCCCCGGATCCAGCGGCTGGGCGCAGCGGCTCCAAA
GTGGCCAAGCTGGGCAGCGGTTGCTTCGGCAACCGGCTCGACCACATCGGCACCTCGAGTGGCATGGGCT
GCAATTGGCCAAACCAAAACCGCCCGAATCCGACACCTGCCGGATCCTAA

>GAHH01000060 Pseudonaja modesta NP-Pse-2 mRNA sequence
ATGGGCGGCCTCTCCCGTCTGGCAGGCGGCGGGCTGCTGCTGCTGCTGCTGGCCCTGCTGCCTGTCGCCC
TCGACGGGAAGCCGGCGCCGCTGCCTCAGGCGCTGCCCGAGGCTCCGGAGGGCAGCGTGATGGCGTCGCG
GCAGGAGCTGACGGAGGAGCAGCAGCAGCTGCCGGCAGAGGTGTCCTCGGGTCACGCGGCTGGGCGCAGC
GGCTCTAAGATAGGGAACGGTTGCTTCGGCGTCCGGCTCGACCGCATCAGCAACACGAGTGGCATGGGCT
GCAGAAACCCCATCCAAAACCGCCCGAAATCGACACCTGGCGGATCCTAA

>GAHH01000061 Pseudonaja modesta NP-Pse-3 mRNA sequence
ATGGTCGGCCTCTCCCGTCTGGCGGGCGGCGGGCTGCTGCTGCTGCTGCTGGCCCTGCTGCCTCTCGCCC
TCGACGGGAAGCCGGCGCCGCTGCCTCAGGCGCTGCCCGAGGCTCCGGCGGGCGGCGTGATGGCATCGGT
GACGGAGGAGCAGCAGCGGCTGCCGGTGTCGGCGGAGGAGTCCCCGGATCCAGCGGCTGGGCGCAGCGGC
TCCAAAGTGGCCAAGCTGGGCAGCGGTTGCTTTGGCAACCGGCTCGACCACATCGGCACCGCGAGTGGCA
TGGGCTGCAATTGGCCAAACCAAAACCGCCCGAATCCGACACCTGCCGGATCCTAA

>GAHH01000062 Pseudonaja modesta NP-Pse-5 mRNA sequence
ATGGTCGGCCTCTCCCGTCTGGCGGGCGGCGGGCTGCTGCTGCTGCTGCTGCTGGCCCTGCTGCCTCTCG
CCCTCGACGGGAAGCCGGCGCCGCTGCCTCAGGCGCTGCCCGAGGCTCCGGCGGGCAGCGTGATGGCATC
GGTGACGGAGGAGCAGCAGCGGCTGCTGGTGGCGGCGGAGGAGTCCCCGGATCCAGCGGCTGGGCGCAGC
GGCTCCAAAGTGGCCAAGCTGGGCAGCGGTTGCTTCGGCAACCGGCTCGACCACATCGGCACCTCGAGTG
GCATGGGCTGCAATTGGCCAAACCAAAACCGCCCGAATCCGACACCTGCCGGATCCTAA

>GAHH01000063 Pseudonaja modesta NP-Pse-6 mRNA sequence
ATGGTCGGCCTCTCCCGTCTGGCAAGGGGCGGGCTGCTGCTGCTGCTGCTGGCCCTGCTGCCTCTCGCCC
TCAACGGGAAGCCGGCGCCGCTGCCTCAGGCGCTGCCCGAGGCTCCGGAGGGCAGCGTGATGGCGTCGCG
GCAGGAGCTGACGGAGGAGCAGCAGCAGCTGCCGGCAGAGGTGTCCTCGGGTCACGCGGCTGGGCGCAGC
GGCTCTAAGATAGGGAACGGTTGCTTCGGCGTCCGGCTCGACCGCATCAGCAACACGAGTGGCATGGGCT
GCAATTGGCCAAACCAAAACCGCCCGAATCCGACACCTGCCGGATCCTAA

>GAHH01000064 Pseudonaja modesta NGF-Pse-1 mRNA sequence
ATGGTGCATAGCGTAATGTCCATGTTGTGCTACACTCTGATTATAGCATTTCTGATTGGCATATGGGCAG
CACCAAAATCTGAAGATAATGTCCCTCTGGGCTCCCCTGCAACATCTGACCTTTCTGACACCAGCTGTGC
TCAAACTCACAAGGCTCTGAAAACATCTCGAAATACAGATCAGCGCCATCCTGCTCCTAAGAAGGCAGAA
GATCAAGAACTTGGATCAGCAGCCAACATCATTGTGGATCCCAAGCTTTTTCAGAAGAGGCGTTTCCAGT
CACCTCGTGTTCTGTTCAGCACTCAGCCCCCACCATTGTCAAGAGATGAGCAAAGTGTGGAGTTCCTGGA
CAATGAAGACGCTCTTAACAGGAATTTCCGGGCCAAACGTGAAACTCATCCTGTGCATAACCAAGGGGAA
TATTCTGTGTGTGACAGTATCAGTGTCTGGGTTGGCAACAAAACCAAAGCAATAGACATCAAAGACAAAC
CGGTGACTGTGATGGTCGATGTAAATCTTAATAACCATGTCTTCAAGCAGTACTTTTTKGAAACCAAGTG
CAGAAATCCAAATCCAGTACCAAGTGGGTGCAGGGGCATTGATTCCAGGCATTGGAATTCTTATTGCACC
ACAACACAGACATATGTCAGGGCATTAACCATGGAAGGCAATCAGGCATCCTGGCGCTTCATTCGGATTG
ACGCTGCCTGTGTGTGCGTAATCAGTAGAAAACTGACAACTTCTGATGGGCCATTGATTGCTCCAATACT
CACTTTC

>GAHH01000065 Pseudonaja modesta PLA2-Pse-2 mRNA sequence
ATGCATCCTGCTCACCTTCTGGTCCTGTTGGGAGTTTGTGTCTCCCTCTTAGGAGCCGCCAGAATTCCTC
CGCTGCCTCTCGACCTCATAGATTTCTCCAACCTGATTACATGTGCTAACCGTGGCAGTCGAAGTTGGTT
GGATTATGCGCACTACGGTTGCTTCTGCGGCTCAGGAGGTAGCGGGACACCGGTAGACGACTTGGATAGG
TGCTGCCAGGTTCATGACAACTGTTATGGTGACGCCGAAAAACTTCCTGCATGTAATTACCAGTTCAGTA
GCCCCTACTGGAACCCCTATTCATACAAATGTAATGAAGGCGAAATCACCTGCACAGATGACAAGGATGA
GTGTAAAGCCTTTATTTGTAATTGCGACCGCACAGCAGCCATCTGTTTCGCCAGAGCCACTTACAAGGAC
GAAAACTTCATGATCTCCACCAAGAAAATAATATTTGCCAATGATATTTGA

>GAHH01000066 Pseudonaja modesta PLA2-Pse-3 mRNA sequence
ATGCATCCTGCTCACCTTCTGGTCCTGTTGGGAGTTTGTGTCTCCCTCTTAGGAGCCGCCAGAATTCCTC
CGCTGCCTCTCGACCTCATAGATTTCTCCAACCTGATTACATGTGCTAACCGTGGCAGTCGAAGTTGGTT
GGATTATGCGCACTACGGTTGCTTCTGCGGCTCAGGAGGTAGCGGGACACCGGTAGACGACTTGGATAGG
TGCTGCCAGGTTCATGACAACTGTTATGGTGACGCCGAAAAACTTCCTGCATGTAATTACCAGTTCAGTA
GCGCCTACTGGAACCCCTATTCATACAAATGTAATGAAGGCGAAATCACCTGCACAGATGACAAGGATGA
GTGTAAAGCCTTTATTTGTAATTGCGACCGCACAGCAGCCATCTGTTTCGCCAGAGCCACTTACAAGGAC
GAAAACTTCATGATCTCCACCAAGAAAAATAATATTTGCCAATGA

>GAHH01000067 Pseudonaja modesta PLA2-Pse-4 mRNA sequence
ATGCATCCTGCTCACCTTCTGGTCCTGTTGGGAGTTTGTGTCTCCCTCTTAGGAGCCGCCAGAATTCCTC
CGCTGCCTCTCGACCTCATAGATTTCTCCAACCTGATTACATGTGCTAACCGTGGCAGTCGAAGTTGGTT
GGATTATGCGCACTACGGTTGCTTCTGCGGCTCAGGAGGTAGCGGGACACCGGTAGACGACTTGGATAGG
TGCTGCCAGGTTCATGACAACTGTTATGGTGACGCCGAAAAACTTCCTGCATGTAATTACCAGTTCAGTA
GCGCCTACTGGAACCCCTATTCATACAAATGTAATGAAGGCGAAATCACCTGCACAGATGACAACGATGA
GTGTAAAGCCTTTATTTGTAATTGCGACCGCACAGCAGCCATCTGTTTCGCCAGAGCCACTTACAAGGAC
GAAAACTTCATGATCTCCACCAAGAAAAATAATATTTGCCAATGA

>GAHH01000068 Pseudonaja modesta PLA2-Pse-5 mRNA sequence
ATGCATCCTGCTCACCTTCTGGTCCTGTTGGGAGTTTGTGTCTCCCTCTTAGGAGCCGCCAGAATTCCTC
CGCTGCCTCTCGACCTCATAGATTTCTCCAACCTGATTACATGTGCTAACCGTGGCAGTCGAAGTTGGTT
GGATTATGCGCACTACGGTTGCTTCTGCGGCTCAGGAGGTAGCGGGACACCGGTAGACGACTTGGATAGG
TGCTGCCAGGTTCATGACAACTGTTATGGTGACGCCGAAAAACTTCCTGCATGTAATTACCAGTTCAGTA
GCGCCTACTGGAACCCCTATTCATACAAATGTAATGAAGGCGAAATCACCTGCACAGATGACAAGGATGA
GTGTAAAGCCTTTATTTGTAATTGCGACCGCACAGCAGCCATCTGTTTCGCCAGAGCCACTTACAAGGAC
GAAAACTTCATGATCTCCACCAAGAAAATAATATTTGCCAATGATATTTGA

>GAHH01000069 Pseudonaja modesta PLA2-Pse-8 mRNA sequence
ATGAATCCTGCTCACCTTCTGGTCCTGTTGGGAGTTTGTGTCTCCCTCTTAGGAGCCGCCAGAATTCCTC
CGCTGCCTCTCAGCCTCGTAGAATTCAGAAGCTTGATTAAATGTGCCAACCATAACAGTCGAAATGTGTA
CGATTATGTGGACTACGGTTGCTACTGTGGCAAAGGAGGTAGCGGGACACCGGTAGACGAGTTGGACAGG
TGCTGCCAGGCTCATGACTACTGCTATGATGATGCCGAAAAACTTCCTGCATGTAATTACTGGTTCAGTG
GCGCCTACTACAACTCCTATTCATACAAATGTAATGAAGGCGAAATCACCTGCACAGATGACAACGATGA
GTGTCAAGCCTTTATTTGTAATTGCGACCGCACAGCAGCCATCTGTTTCGCCAGAGCCCCTTATGACAAG
GACAACTGGAATATCAACACCGAGACAGATTGCTAA

>GAHH01000070 Pseudonaja modesta SVMP-Pse-2 mRNA sequence
ATGGTTGCAGACAATTTAGTTTACAGGAAGTACAGCGGCAACATCACTGATGTAAGGATGAGAATATTTG
AAATTCTCAACTATGTAAATTTGTATTAYAAAGTTTTCAATATTCATGTAATATTGATTGGCTTAGAAGT
TTGGTCTGATGAAGATAAGATTCTGATCAATGGATCATCAGAACCCACTGTGAAATCCTTTGCAGCATGG
AGACATTCAGATCTACTGAAGCGCAAAAGGAATGACAATGCTCAGTTACTCACGGGTATTCACTTCGATG
AAGGAGTCTTAGGAGTAGCTTTCATAGGCGGCATGTGCAATAATTTCACATCTGTAGGAGTTATTCAGGA
TAACAGCATCCAGGCAGTTTTAATTGCAGCTGT

>GAHH01000071 Pseudonaja modesta SVMP-Pse-4 mRNA sequence
ATGGACAGCTTCCAGAGGAATGGACATCCATGCCAAAACAACCAAGGTTACTGCTACAATGGGAAATGCC
CCATCATGACAAACCAATGTATCGATCTAGGGGGGCCAGGTGTAAATGTGTCTCCAGATATATGTTTTAC
ATTGAACCAGTATAGCCAAGGTTGTGGCTTCTGCAGAATGGAAAATGGTACAAAGATTCCGTGTGCAGCA
AAGAATAAAATGTGTGGCAAGTTAATATGCGAAAAGGGAAACTCGACATGCACCTGCTTTTCTGCAACAG
ATGACCCAGATTATGGAATGGTTGAACCTGGAACAAAATGTGGAGATGGAATGGTGTGCATCAACAGGCA
GTGTGTTGATGTTCAGACAGCCTACTGA

>GAHH01000072 Pseudonaja modesta SVMP-Pse-3 mRNA sequence
ATGTACAAGAATTATGACAGCAACAGACATGCTATAAAAAGAAAAGTATATGAAACAATCAACCTTTTAA
ACATGATGTACAGACCTTTGAATTTTCTCATTGCACTGATTGGCCTAGAAATTTGGTCCAACCGAGATAA
GATTAACATTGAACCAGAGGTGGCTGTCACTTTGAAATCATTTGGAAAATGGAGAGAAACAGTTTTACTG
CCACGCAAAAGGAATGATAATGCTCAGTTACTTACGCAGATTGAGTTTAACGGAACTACTGTAGGACTTG
CTTATGTGGGCAGCATCTGCAGTCCGAAGGAATCTGTAGCAGTTATAGAGGTTTATAGCAGAAGAACAGA
TATAATTGCATCTGAAATGGCCCATGAGTTGGGTCATAATCTGGGCATTACTCATGACCACGCTTCCTGT
AATTGCAGTGCTGAACTATGTATTATGTCTGCGATAATAAGTTTTGAACCTCTCTCTGAGTTCAGCTCTT
GTAGTATCCAGGAACATCAGAGGTATCTTCTTAGAGAGAGACCACAATGCATTCTCAACAGACCCTTGAG
CACAGATATTGTTACACCTCCAGTTTGTGGAAATTACTTAGTGGAGGTGGGAGAAGAATGTGACTGTGGC
TTTCCTGTGGATTGTCAAAGTGCCTGCTGCAACGCTACAACTTGTAAACTGCAACATGAGGCACAGTGTG
ACTCTGAAGAGTGTTGTGAGAAATGCAAACTTAAGAAAGCAGGAGCAGAATGCCGGGCAGCAAAGGATGA
CTGTGACTTGCCTGAAATCTGCACTGGCCAATCTGCCGAGTGTCCCATGGACAGCTTCCAGAGGAATGGA
CATCCATGCCAAAACAACCAAGGTTACTGCTACAATGGGAAATGCCCCATCATGACAAACCAATGTATCG
ATCTAGGGGGGCCAGGTGTAAATGTGTCTCCAGATATATGTTTTACATTGAACCAGTATAGCCAAGGTTG
TGGCTTCTGCAGAATGGAAAATGGTACAAAGATTCCGTGTGCAGCAAAGAATAAAATGTGTGGCAAGTTA
ATATGCGAAAATGGATGGTCGAGTATAAGAATAATAAGAAATATTTTAGGGTCTCCACTGTGGGAATATG
ATTCGGTCATCTGGCTAAAACAG

>GAHH01000073 Pseudonaja modesta VEGF-Pse-3 mRNA sequence
ATGAACTTTCTGCTCACTTGGATCCATTGCGGGTTGGCGGCGCTGCTTTATTTCCACAACGCCAAGGTGT
TGCAGGCTGCTCCTGCCCAAGAGGATGGAGAGAAGCAACCAGATGAAGTTATCCCATTTATGACGGTCTT
TGAGCGGAGTGCCTGCAGGCCCATTGAGACCATGGTAGATATTTACCAGGAGTATCCTGATGAGGTGGAA
TACATGTTCAAACCATCCTGTGTGCTCCTGATGAAATGTGGAGGATTCTGTAATGACGAAGCGCTAGAAT
GTGTGCCCACAGAGGTGTACAATGTCAGCATGGAGATCATGAAACTCAGACACTTTCAGAGCCAGCATAT
ACAACTGATGAGCTTCCAGCAGCACAGTAAATGTCAATGCAGACAAAAGAAACCAATGAGAATTAAACAA
GAAAATCACTGTGAACCTTGCTCAGAAAGGAGAAAGCACTTGTACAAACAAGATCCCTTGACCTGTAAAT
GTTCCTGCAAATTCACAGACTCACGTTGCAAGTCGAAGCAGCTTGAGTTAAACGAGCGCACTTGCAGATG
TGACAAACCAAGGCGGTGA

>GAHI00000000.1|GAHI01000000 TSA: Suta fasciata, transcriptome shotgun assembly, transcriptome shotgun assembly

>GAHI01000001 Suta fasciata 3FTx-Sut-45 mRNA sequence
ATGAAAACTCTGCTGCTGACCTTGGTGGTGGTGACAATCGTGTGCCTGGACTTAGGTGACAGCTTGATAT
GCTACCTGGGACCTAATAACCCTCAGACTTGTCCACCTGGGGAGAAGCTATGCTTTACAAAGGCTTGGTG
TGATGCTTGGTGTGGCATCAGAGGAAAGCGAATGGAATTTGGATGTGCTGCTACTTGCCCTACAGGGAAG
CCCGGTGTGGATATTACCTGTTGCTCAACAGACAAGTGCAACCCACATCCGAAACAGCAAAGTCGTTAA

>GAHI01000002 Suta fasciata 3FTx-Sut-50 mRNA sequence
ATGAAAACTCTGCTGCTGACCTTGGTGGTGGTGACAATCGTGTGCCTGGACTTAGGTGACAGCTTGATAT
GCTACCTGGGACCTAATAACCCTCAGACTTGTCCACCTGGGGAGGAGCTATGCTTTACAAAGGCTTGGTG
TGATGCTTGGTGTGGCATCAGAGGAAAGCGAATGGAATTTGGATGTGCTGCTACTTGCCCTACAGGGAAG
CCCGGTGTGGATATTACCTGTTGCTCAACAGACAAGTGCAACCCACATCCGAAACAGCAAAGTCGTTAA

>GAHI01000003 Suta fasciata 3FTx-Sut-59 mRNA sequence
ATGAAAACTCTGCTGCTGACCTTGGTGGTGGTGACAATCGTGTGCCTGGACTTAGGTGACAGCTTGATAT
GCTACCTGGGACCTAATAACCCTCAGACTTGTCCACCTGGGGAGAAGCTATGCTTTACAAAGGCTTGGTG
TGATGCTTGGTGTGGCATCAGAGGAAAGCGAATGGAATTTGGATGTGCTGCTACTTGCCCTACAGGGAAG
CCCGGTGTGGATATTACCTGTTGCTCAACAGACAAGTGCAACCCACATCCGAAACAGCAAGGTCGTTGA

>GAHI01000004 Suta fasciata 3FTx-Sut-134 mRNA sequence
ATGAAAACTCTGCTGCTGACCTTGGTGGTGGTGACAATCGTGTGCCTGGACTTAGGTGACAGCTTGATAT
GCTACCTGGGACCTAATAACCCTCAGACTTGTCCACCTGGGGAGAAGCTATGCTTTACAAAGGCTTGGTG

TGATGCTTGGTGTGGCATCAGAGGAAAGCGAATGGAATTTGGATGTGCTGCTACTTGCCCTACAGGGAAG
CCCGGTGTGGATATTACCTGTTGCTCAACAGACAAGTGCAACCCACACTACCGAAACAGACAAAGTCGTT
AA

>GAHI01000005 Suta fasciata ACN-Sut-1 mRNA sequence
ATGCCCTCCTGCCAGCCTGGCAAGATGCCTGCACCGTGGCCTTGGTGCCTCCAGCTGGTCCTTTGCATCC
TGTCCTCCGTAGCAGTCTTGCCTGGCCGGGCAAGCGAGCTGAAAGTGTCCACGCAGATGGGCTGGGTGCG
TGGCCTGAGCCTGCCGGTTCTCGACGGTCACGTCTCGGCCTTCCTGGGTGTCCCCTTTGCGGAGCCTCCC
GTGGGCAGGATGCGCTTCCTGCGCCCTGAGCCCGTCAAGCCCTGGCAGCACGTCCTGGACGCCACTTCCT
ACCAGCGCGCCTGTTACCAGATGGTGGACACCAGCTACCCAGGCTTCCAGGGCACGGAGATGTGGAACCC
CAACTGGGGCATGAGCGAGGACTGCCTCTACCTCAACATCTGGGTGCCCTCCCCGCGCCCCAGAGACGTC
CCCGTCCTGGTCTGGATCTACGGAGGGGGCTTCTACAGTGGCGCTGCCTCCCTGGACGTCTACGATGGTC
GCTTCCTGACCTACACACAGAACGTCATCCTGGTCTCCCTCAGCTACCGCGTGGGGGCCTTGGGCTTTTT
GGGCCTCCCGGGCAGCCCGGAGGCGCCGGGGAACATGGGCCTGCTGGACCAGCGCTTGGCCCTGCAGTGG
ATCCAGAACAACATCAATTATTTTGGGGGCAATCCCAACGCGGTCACCATCTTTGGGGAGAGTGCCGGGG
CCGCCTCGGTGGGCATGCACCTGCTTTCCACCCACAGCCGCGGCCTCTTCCAGCGGGCCATCCTGCAGAG
CGGCGGCCCCAACGCACCGTGGGCCACCGTCACGCCTGCCGAAAGCCGGCGCCGGGCGGCCCTTCTGGGA
AAGCAGCTGGGCTGCCAGTTCAACAACGATTCCGAGCTGGTCAGTTGCTTGCGCTCGAAAACCCCGCAGG
AGCTGATCGACGAGGAGTGGTCCGTCCTGCCCTACAAGAGCATTTTCCGCTTCCCCTTCGTCCCGGTCAT
TGACGGGAACTTCTTTCCGGACACCCCCGAGGCCATGCTGAGCTCGGGCAACTTCAAGGAGACCCAGGTG
TTGCTGGGCGTGGTGAAGGACGAGGGCTCCTACTTTCTGATCTACGGGCTGCCCGGCTTCAGCAAGGACA
ACGAGAGCCTCATCAACCAGGCCGACTTCTTGGAAGGGGTGCGGATGAGCGTGCCTCACGCCAACGACAT
CGCCACCGAGGCCGTGGTGCTGCAGTACACTGACTGGCAGGACCAGGACAACGGGGAGAAGAACAGGGAG
GCCATGGACGACATCGTGGGGGACCACAACGTCATCTGCCCGGTCGTGCAGTTCGCCAATGACTACGCCA
AGCGCAACAACAAGGTGTACGCCTACCTCTTTGACCACCGGGCCTCCAACCTGCTCTGGCCTCCCTGGAT
GGGGGTCCCCCACGGCTACGAGATCGAGTTTGTCTTTGGATTGCCCCTCAACGACAGCCTGAACTACACG
CCCCAAGAGAAGGAGCTGAGCCGCAGGATGATGCGCTACTGGGTCAACTTCGCCCGGACGGGGAACCCCA
TCGACTCTACCGAGAAGAACGGGGCCTGGCCCACCTACACCGCCTCCCGGCCGCAATATGTCCAGCTCAA
CACCCAGCCGCTGGCCACCCAACCCAGTCTGCGGGCCCAGATCTGCGCCTTCTGGAACCAATTCCTCCCC
AAACTGCTGAACGCCACAG

>GAHI01000006 Suta fasciata CRiSP-Sut-19 mRNA sequence
ATGATTGCCTTCATTGTCTTGCTAAGTCTTGCTGCAGTGCTGCAACAGTCTTCTGGAACTGTTGATTTTG
CTTCTGAGTCAAGTAACCAAAAAGATTACCAAAAGGAGATTGTTGACAAGCACAATGCTTTAAGGAGATC
AGTGAAACCAACTGCTAGGAACATGTTACAAATGAAATGGAATTCTCGTGCTGCTCAAAATGCAAAACGT
TGGGCAGATAGATGTACTTTTGCTCACAGTCCACCACATACAAGAACTGTGGGAAAACTCCGTTGTGGTG
AAAATATATTCATGTCAAGTCAACCTTCTTCATGGAGTGTCATAGTTCAGGCTTGGTATGATGAAGTCAA
AAAATTTGTCTATGGCATTGGAGCAAACCCACCAGGTTCTGTTATTGGCCATTATACCCAGATAGTTTGG
TACAAAAGTCGCTTTCTTGGTTGTGCTTCTGCCAAATGTTCTTCAACCGAATACTTCTACGTTTGTCAAT
ACTGCCCAGCAGGGAACATGGAAGGTTCAATTGCTACTCCATATAAATCAGGGCCAACTTGTGGGGACTG
TCCTTCGGCTTGTGTCAACGGACTATGCACAAATCCCTGTGAACATAACGATGACTTCACGAACTGCAAA
GATTTAGCGAAAGAAAGTAAATGCCAGACTGAATGGATCAAGTCAAAATGCCCTGCTACTTGCTTCTGCC
ACACTGAAATAATATAG

>GAHI01000007 Suta fasciata CRiSP-Sut-27 mRNA sequence
ATGATTGCCTTCATTGTCTTGCTCAGTCTTGCTGCAGTGTTGCAACAGTCTTCTGGAACTGTTGATTTTG
CTTCTGAGTCAAGTAACAAAAAAGATTACCGAAAGGAGATTGTTGACAAGCACAATGCTTTAAGGAGATC
AGTGAAACCAACTGCTAGGAACATGTTACAAATGAAATGGAATTCTCGTGCTGCTCAAAATGCAAAACGT
TGGGCAGATAGATGTACTTTTGCTCACAGTCCACCACATACAAGAACTGTGGGAAAACTCCGTTGTGGTG
AAAATATATTCATGTCAACTCAACCTTTTGCATGGAGTGGTGTAGTTCAGGCTTGGTATGATGAAGTCAA
AAAATTCGTCTATGGCATTGGAGCAAAGCCCCCAGGTTCTGTTATTGGCCATTATACCCAGGTAGTTTGG
TACAAAAGTCACCTTCTTGGTTGTGCTTCTGCCAAATGTTCTCCAACCAAATACCTCTACGTTTGTCAAT
ACTGCCCAGCAGGGAACATCGAAGGTTCAATTGCTACTCCATATAAATCAGGGCCACCTTGTGGGGACTG
TCCTTCGGCTTGTGTCAAGGGGCTATGCACAAATCCTTGCAAACATAATGATGACTTCTCGAACTGCAAA
GATTTAGCGAAAAAAAGTAAATGCAAGACTGAATGGATCAAGTCAAAATGCCCTGCTACTTGCTTCTGCC
ACAATAAAATAATATAG

>GAHI01000008 Suta fasciata fX-Sut-7 mRNA sequence
ATGGCTCCTCAGCTACTTCTCTGTCTGATCCTCACTTTTCTATGGAGTCTCCCAGAGGCTGAAAGTAATG
TATTCTTAAAAAGCAAAGTGGCAAATAGATTTTTGCAAAGAACAAAACGATCTAATTCACTGTTTGAGGA
ATTTAAAGCTGGAAACATTGAAAGGGAATGCATTGAGGAGAAATGTTCAAAAGAAGAAGCCAGGGAGGTA
TTTGAAGATAACGAAAAAACTGAGACCTTCTGGAATGTTTATGTAGATGGGGATCAGTGTTCATCAAACC
CTTGTCATTATCGCGGGACATGCAAAGATGGCATTGGTAGCTATACCTGTACCTGCTTGCCTAACTATGA
CGGGAAAAACTGTGAAAAAGTCTTATACGAGTCCTGCAGAGTGGACAATGGTAACTGTTGGCACTTCTGC
AAAAGTGTTCAAAACGAAGTTCAGTGTTCATGTGCTGAAAGTTACCGTTTGGGAGATGATGGGCGCTCTT
GTGTTGCTGAAGGTGACTTTTCATGTGGTAGAAATATAAAAGCAAGGAACAAGAGGGAAGCAAGTCTGCC
TGACTTTGTGCAGTCCCAGAAGGCAACTTTGCTGAAAAAATCTGATAATCCAAGCCCTGATATCAGAATT
GTTAATGGAATGGATTGCAAACTGGGTGAATGTCCATGGCAGGCAGTTCTGCTAGATGAAAAAGAAAAAG
TGTTTTGTGGAGGAACAATTCTGAGTCCCATC

>GAHI01000009 Suta fasciata KP-Sut-1 mRNA sequence
ATGTCTTCTGGAGGTCTTCTTCTCCTGCTGGGACTCCTCACCCTCTGGSCAGAGATGASSSYCATCTCTG
GCCAGGACCGTCCAAAGTATTGTGATCTTCCTGCTAACCCTGGACCATGTAGAGCAAGAATCACTCGCTT
TTACTACAACTCGGATTCAAAACAATGTGAAGAGTTTATTTATGGTGGATGCCACGGCAATGCCAACAAT
TTTGAGACCAAAGATAAATGTCATTACACCTGTGTTGAAAAGCCTGGGGTGTGTCCCAAACCCGGTCCAG
ACGTTGTCACTATTTGTCTCGTGAGATGTGAAAGTGACTGGAAATGTCCTGGGAAGGAGAAGTGCTGCAG
TTACGCATGCCTGCGTGATTGCATGGAGCCTGTCTAA

>GAHI01000010 Suta fasciata KP-Sut-2 mRNA sequence
ATGTCTTCTGGAGGTCTTCTTCTCCTGCTGGGACTCCTCACCCTCTGGGAGGGGCTGACCCTCGTCTCCA
GCACGGACCGTCCAGACTTCTGTGAACTGCCTGATGATTCCGGACCATGTAAAGGCAGCTTCGAAGCCTT
CTACTACAACGCAGATCAACATAAATGCCTAGAGTTTATTTATGGGGGATGCGACGGGAATGCTAACAAT
TTTAAGACCATAGAGGAATGCGAACACACCTGTGCTGCATGA

>GAHI01000011 Suta fasciata KP-Sut-4 mRNA sequence
ATGCCACAAGTCAGATGTGCCAGGAATTCATTTTTTGGAGGTTGCAAAGGCAATGCCAACAACTTCGTCT
CCAAACAGGACTGCTTCCAGACGTGTATCAGAGGAGGGGCTGCAGAGGCAACAGTGGTACCTAGCAGGCC
AGCCACTGAAGTGGCTACACCAAGAGCTGGACATCTCCCAGAGGCCTACGAAAACAGGCCTGGCTTCAGA
GAATTCTGTGCTGCGCCCCGGGTGGTAGGTCCCTGCCGGGCCTCCTTCCTCCGTTGGTACTTCGACCTAG
AGAGCCGGATGTGCAAGATGTTCGTCTATGGGGGCTGTCGTGGCAACAAGAACAACTATCTCTTCGAAGA
GCACTGCTGGAGCCAGTGCACAGGTGATGGAGAGATAACTGAAGAGCCAGGAGATGCAGGCGCCCAGCCT

CCCCTCCCCTCCGAACCCTTCAGCTTCTCCACCAGAGGTGTGGTCCTTGCTGTCCTTCTGGCCATCCTGG
TGACTATCCTGCTGGGCTCGATMGGGGGTTTTCTTTGTTAA

>GAHI01000012 Suta fasciata LP-Sut-3 mRNA sequence
ATGGCGCAGTTCATCGTTGTGAGCGTCAGCTTGCTGATCGTGGCCCTCTCCTTAACGGGTAACGTAGAGG
CTGATCACCACTTGTTCCTACGCGGTTGGGCCGTCCTACTAATGAGTTTTGTCTACATGGTCTACAACAG
ATCCGTGACCTGGAACAATGCCGAGAGATTCTGCCTGCAGCAAGAGACAAACTGCCACCTCGCCTCCATC
CATAGCAAGCCAGAGGCAGCCTTCATAGTCAGGCTGGTCTACCACAGGGCCAGCGCCGGGTCCAGTGTCT
GGATCGGGTTGAACGATCCAGAGAAAAGACGCGCCTGGCAATGGAGTGATGGCTCCAGCTTAAAGTACAG
ATCCTGGATGCCAGGAGAGCCCAACAACCATGCAAACAACGAGTATTGTGTTGTGCTGTCAGCCTGGTCA
CGTTATGTCGGATGGAACGATCAGGACTGTGGGTCCAGCCATAATTTTGTCTGCAAGTTCCAGCCAAAGC
CGAGAGTAACACCGTCAATGTTCCAAAGAGGAAGTAATGAAGTCCTATTCCCTATTGAAACCTCTGCTCT
GCACCTTGTCGCTTCATGGATGCTTTC

>GAHI01000013 Suta fasciata NP-Sut-1 mRNA sequence
ATGGTCGGCCTCTCACGTCTGGCGGGCGGCGGGCTGCTGCCGCTGCTGCTGCTGGCCCTGCTGCCTCTCG
CCCTCGACGGGAAGCCGGCGCCGGTGCCTCAGGCGCTGCCCGAGGCTCCGGCGGGCGGCACGACGGCGTG
GCGGCGGGACCTGACGGAGCAGCAGCCGCCGCCGGCGGAGGAGTCCTCGGGTCCCGCGGCCGGGCGCAGC
GGCTCCAAGGCGGGCCAGGCAGCCCCGACGCCGCCGAAAGGCAAAGGGGCGGCGGTGTCGGCGGCCGCCT
CGCGGCTCCTGCGAGATTTGCGCCGCGACGTCAAGGAGTCGCGCCCCACCTCGGGCCGGTTGGCGTATCC
CGAATACCCCGCAGGAGGAGGAGGAGGAGGCGGCGGCGGCGGACAGTCGCGGGACACCTGGGGCCGGTTG
GCGTACCCCGAGTACCCCGCAGGAGGCGGAGGCGGCGGCGGCGCCTGGCGTCGTGCGAAGAGGCCGCCTA
AGAAAGGGCGGACCTTCAAGAGCTGCTTCGGCCTCCCGCTCGACCGCATCGGCACCAAGAGCGGCCTGGG
CTGCTGA

>GAHI01000014 Suta fasciata NP-Sut-10 mRNA sequence
ATGGTCGGCCTCTCACGTCTGGCGGGCGGCGGGCTGCTGCTGCTGCTGGCCCTGCTGCCTCTCGCCCTCG
ACGGGAAGCCGGCGCCGGTGCCTCAGGCGCTGCCCGAGGCTCCGGCGGGCGGCATGATGGCGTGGCGGCG
GGACCTGACGGAGCAGCAGAAGCCGCAGCCGATGGCGGAGTCCCCGGGTCCCGCGGCCGAGCGCAGCGAC
TCCAAGACGGCCACGAAAGGGGACGGCTGCTTCGGCGTCCGGATCGACCGTATCGGCAGCGTAAGCGGCA
TGGGCTGCGGAGGGGTCCCGAAGCCGACGCCTGGCGGATCCTAA

>GAHI01000015 Suta fasciata NP-Sut-13 mRNA sequence
ATGGTCGGCCTCTCACGTCTGGCGGGCGGCGGGCTGCTGCCGCTGCTGCTGCTGGCCCTGCTGCCTCTCG
CCCTCGACGGGAAGCCGGCGCCGGTGCCTCAGGCGCTGCCCGAGGCTCCGGCGGGCGGCACGATGGGGTC
GCGGCCGCTGCTGACGGAGCAGCAGAAGCAGCCGCAGCCGCTGGCGGAGTCCCCGGGTCCCGCGGCGGAG
CGCAGCGACTCCAAGACGGCCACGAAAGGGAACGGCTGCTTCGGCCACCGGATCGACAACATCGGCAGCG
TAAGCGGCATGGGCTGCGGAGGCAAGCCGAAGCCGAAGCCTGGCGGATCCTAA

>GAHI01000016 Suta fasciata NP-Sut-14 mRNA sequence
ATGGTCGGCCTCTCACGTCTGGCGGGCGGCGGGCTGCTGCTGCTGCTGGCCCTGCTGCCTCTCGCCCTCG
ACGGGAAGCCGGCGCCGGTGCCTCAGGCGCTGCCCGAGGCTCCGGCGGGCGGCACGATGCTGACGGAGCA
GCAGAAGCAGCCGCAGCCGCTGGCGGAGTCCCCGGGTCCCGCGGCGGAGCGCAGCGACTCCAAGACGGCC
ACGAAAGGGAACGGCTGCTTCGGCGTCCGGATCGACCGTATCGGCAGCGTAAGCGGCATGGGCTGCGGAG
GCAAGCCGAAGCCGACGCCTGGCGGATCCTAA

>GAHI01000017 Suta fasciata NP-Sut-19 mRNA sequence
ATGGTCGGCCTCTCACGTCTGGCGGGCGGCGGGCTGCTGCCGCTGCTGCTGGCCCTGCTGCCTCTCGCCC
TCGACGGGAAGCCGGCGCCGGTGCCTCAGGCGCTGCCCGAGGCTCCGGCGGGCGGCACGATGCTGACGGA
GCAGCAGAAGCAGCCGCAGCCGCTGGAGGAGTCCCCGGGTCCCGCGGCGGAGCGCAGCGACTCCAAGACG
GCCACGAAAGGGAACGGCTGCTTCGGCCACCGGATCGACAACATCGGCAGCGTAAGCGGCATGGGCTGCG
GAGGCAAGCCGAAGCCGAAGCCTGGCGGATCCTAA

>GAHI01000018 Suta fasciata NP-Sut-24 mRNA sequence
ATGGTCGGCCTCTCACGTCTGGCGGGCGGCGGGCTGCTGCTGCTGCTGGCCCTGCTGCCTCTCGCCCTCG
ACGGGAAGCCGGCGCCGGTGCCTCAGGCGCTGCCCGAGGCTCCGGCGGGCGGCACGATGCTGACGGAGCA
GCAGAAGCAGCCGCAGCCGCTGGCGGAGTCCCCGGGTCCCGCGGCGGAGCGCAGCGACTCCAAGACGGCC
ACGAAAGGGAACGGCTGCTTCGGCGTCCGGATCGACCGTATCGGCAGCGTAAGCGGCATGGGCTGCGGAG
GCAAGCCGAAGCCG

>GAHI01000021 Suta fasciata PLA2-Sut-19
ATGTATCCTGCTCACCTTCTGGTTCTGTTGGCAGTTTGTGTCTCCCTCTT
AGGAGCCGCCGGCATTCCTCCGCAGCCTCTCAACCTCTATCAGTTCAAGG
GCATGATTGAATGTGGCAACCGTGGCAATCGAGATGCATGGGATTATAAT
AACTACGGTTGCTACTGCGGCCAGGGAGGTAGCGGGACACCAGTAGACGA
CTTGGATAGGTGCTGCAAAGTACATGACGACTGCTATGGTGAAGCCGAAA
ACAGCCTGAATTGCTCCCCCTACTGGACATTGTAT
ACTTGGGACTGTACTGAAAATGTACCCACCTGCAAATCAAAATCGGGGTG
TCCACGTTCTGTGTGTGAATGTGACGCCGTAGCAGCCAAGTGCTTTGCCA
AAGCCGATTACAACGACGCGAACAAGGGTATCAAAGGCACTGCACGTTGC
CAATGA
>GAHI01000022 Suta fasciata PLA2-Sut-22
ATGTATCCTGCTCACCTTCTGGTTCTGTTGGCAGTTTGTGTCTCCCTCTT
AGGAGCCGCCGGCATTCCTCCGCAGCCTCTCAACCTCTATCAGTTCAAGG
GCATGATTGAATGTGGCAACCGTGGCAATCGAGATGCATGGGATTATAAT
AACTACGGTTGCTACTGCGGCCAGGGAGGTAGCGGGACACCAGTAGACGA
CTTGGATAGGTGCTGCAAAGTACATGACGACTGCTATGGTGAAGCCGAAA
ACAGCCTGAATTGCTCCCCCTACTGGACATTGTAT
ACTTGGGACTGTACTGAAAATGTACCCACCTGCAAATCAAAATCGGGGTG
TCCACGTTCTGTGTGTGAATGTGACGCCGTAGCAGCCAAGTGCTTTGCCA
AAGCCAATTACAACGACGCGAACAAGGGTATCAAAGGCACTGCACGTTGC
CAATGA
>GAHI01000023 Suta fasciata PLA2-Sut-20
ATGTATCCTGCTCAACTTCTGGTCCTGTTGGCAGTTTGTGTCTCCCTCTT
AGGAGCCGCCAGGATTCCTCCGCAGCCTCTCAACCTCCTACACTTCGGCT
TCATGATTCAATGTGCCAACCGTCGCAGTCGAGGTGCATTGGATTATAAT
AACTACGGTTGCTACTGCGGCTCGGGAGGTAGCGGGACACCGGTAGACGA
CTTGGATAGGTGCTGCAAAGTACATGACGACTGCTATGATAAAGCTACCA
AAAGCTACAGTTGCTCCCCCTACTGGACATTGTAT
ACTTGGGACTGTATTGCAAATGTACCCACCTGCAATTCAAAAACGAGATG
TCAACGTTTTGTGTGTGCTTGTGACGCCGTAGCAGCCAAGTGCTTTGCCA

AAGCCAATTACAACGACGCGAACAAGGGTATCAAAGGCACTGCACGTTGC
CAATGA
>GAHI01000024 Suta fasciata PLA2-Sut-23
ATGTATCCTGCTCAACTTCTGGTCCTGTTGGCAGTTTGTGTCTCCCTCTT
AGGAGCCACCAGGATTCCTCCGCAGCCTCTCAACCTCCTACACTTCGGCT
TCATGATTCAATGTGCCAACCGTCGCAGTCGAGGTGCATTGGATTATAAT
AACTACGGTTGCTACTGCGGCTCGGGAGGTAGCGGGACACCGGTAGACGA
CTTGGATAGGTGCTGCAAAAGACATGACGACTGCTATGATAAAGCTACCA
AAAGCTACAGTTGCTCCCCCTACTGGACATTGTAT
ACTTGGGACTGTATTGAAAATGTACCCACCTGCAATTCAAAAACGAGATG
TCAACGTTTTGTGTGTGCTTGTGACGCCGTAGCAGCCAAGTGCTTTGCCA
AAGCCAATTACAACGACGCGAACAAGGGTATCAAAGGCACTGCACGTTGC
CAATGA
>GAHI01000025 Suta fasciata PLA2-Sut-14
ATGTATCTTGCTCACCTTTTGGTCCTGTTGGCAGTTTGTGTCTCCCTCTT
AGGAGCCTCCGACAATTCTCCGCAGCCTCTCAACCTCATACAATTCGGCT
TCATGATTAAATGTGGCAACAAACGCAGACAACCTCTTTTTCGTTATCTG
GACTACGGTTGCTACTGCGGCAAAGGAGGTCGCGGGACACCGGTAGACGA
CTTGGATATGTGCTGCAAAATACATGACGACTGCTATGCTAAAGCTACCA
AAAGCTACAGTTGCTCCCCCTACTGGACATTGTAT
AGTTGGAAATGTTACGCAAGTGGACCCTACTGCAGTTCAAAAACGAGATG
TCAACGTTTTGTGTGTGCTTGTGACGCCGCAGCATCCAGATGCTTTGCCA
GGGCCCATTACAACCCCGCGAACTATAATATCAACACCAAGAAACGTTGC
CAATGA
>GAHI01000026 Suta fasciata PLA2-Sut-25
ATGTATCCTGCTCACTTTCTTGTCCTGTTGGCAGTTTGTGTCTCCCTCTT
AGGAGCCGCCAGGATTCCTCCGCGGCCTCTCAACCTCATACAATTCGGCG
ACATGATTGAATGTGCCAACCAGAACACTCGAAGTGCATTGGATTATATG
GATTACGGTTGCTACTGCGGCAAAGGAGATAGCGGGACCCCGGTAGACGA
CTTGGATAGGTGCTGCAAAATACATGACGACTGCTATGGTGAAGCCGAAA
ACAGCCTGAATTGCTCCCCCTATTGGGCGTGGTAT
ACTTGGCAATGTACTGAAAATGTACCCACCTGCGATTCAAACACGGGGTG
TAAAAGTTCTGTGTGTGATTGTGACGCCGCAGCAGCCAAGTGCTTTGCCG
AAGCCCAATACAACAAGGAGAACTACAATATCGACACCGACACACGTTGC
CAATGA
>GAHI01000027 Suta fasciata PLA2-Sut-1
ATGTATCCTGCTCACCTTCTGGTCCTGTTGGCAGTTTGTGTCTCCCTCCT
AGGAGCCACCGGCATTCCTCCGCGGCCTCTCACCCTCCTACAATTTGACA
CCATGATTGAATGTGCCAACAATCGCAGTCGAAGTTCTAACGATTATATG
GACTACGGTTGCTACTGCGGCGAAGGAGGTAGCGGGACACCGGTAGACGA
CTTGGATAGGTGCTGCAAAATACATGACGACTGCTATGGTGAAGCCAAAA
AGCACGAATGCGACCCCCTGTCGCTGAAGTAT
AGGTGGCGATGTGGCCGACAGGGACCTTACTGCCGTTCAAAAACGCAGTG
TCAACGTTTTGTGTGTCTTTGTGACCTCCTGGCAGCCAAGTGCATTGCCC
AAGAAACTTACAACAGGGAGAACTACAATATCGACACCAAGAAACGTTGC
CAATGA
>GAHI01000028 Suta fasciata PLA2-Sut-36
ATGTATCCTGCTCACCTTCTGGTCCTGTTGCCAGTTTGTGTCTCCCTCTT
AGGAGCCGCCAGGATTCCTCCACGGCCTCTCAACCTTGTACAATTCGACA
ACATTATTCAATGTGCCAACTATGGCAGTCGACCTTCTTGGCATTATCTG
GACTATGGTTGCTACTGCGGCTCAGGAGGTAGCGGGACACCGGTAGACGA
CTTGGATAGGTGCTGCAAAATACATGACGACTGCTATGCTGAAGCCGACA
AAAAAGGATGCTTCCCCAAGTTCTCGGTGTAT
AGTTGGAAATGTGGCAGCGATGGACCCACCTGCAGTGACAACAATGAGTG
TAAAGCCTTTATTTGTAATTGTGACCGCACAGCAGCCAAGTGCTTTGCCG
GAACCCCTTTCAACCAGGAGAACTGGAGTATCGACACCGAGACACGTTGC
CAATGA
>GAHI01000029 Suta fasciata PLA2-Sut-16
ATGTATCCTGCTCAGCTTCTGGTCCTGTTGGGAGTTTGTGTCTCCCTCTT
AGGAGCCGCCAGCATTCCTCCGCAGCCTCTCAACCTCTATCAGTTCAAGA
ACATGATTCAATGTGCTAACCATGGCAGGAGACCTACTTGGCATTACATG
GACTATGGTTGCTACTGCGGTCCAGGAGGTAGCGGGACACCGGTAGATGA
GTTGGATAGGTGCTGCAAAATACATGACGACTGCTATGCTAAAGCTGAAA
CATCCGAATGCAACCCCCAAGTTGCAGATTTTAT
ATTTGGCAATGTGGCAGCGATGGACCACAGTGCGGCAATTCAGAAACGGG
GTGTAAAAGTTCTGTGTGTGCATGTGACGCCGCAGCAGCCAAGTGCTTTG
CCGAAGCCCATTACAACGACGCGAACTGGGATATCAAAACCGACACACCT
TGTCAATGA
>GAHI01000030 Suta fasciata PLA2-Sut-8
ATGTATCCTGCTCACCTTCTGGTCCTGTTGGCAGTTTGTGTCTCCCTCTT
AGGAGCCTCTGACATTCCTCAGCCGTCTTTCGACTTCGAACAATTCAGCA
ACATGATTCAATGTACCATCCCCTGCGAAGAATCTTGCTCGGGTTATATG
GACTACGGTTGCTACTGCGGTCCAGGAGGTAGCGGGACACCGGTAGACGA
CTTGGATAGGTGCTGCCAGACTCATGACAACTGCTATGCTGAAGCTGGAA
AACTTCCTGCATGTAAGGCCATGCTGAGTGAGTCCGACAACGACAGCTAT
TCATACAGCTGTATTGAAGGCCAACTCACCTGCAACGATGACAACGATGA
GTGTAAAGCCTTTATTTGTAATTGTGACCGCACAGCAGTCAACTGCTTCG
CCAGAGCCCCTTACAACGACTCGAACTACGATATCGGCACCACTGAACAT
TGCAAATGA

>GAHI01000019 Suta fasciata SVMP-Sut-51 mRNA sequence
ATGATCCAAGCTCTCTTGGTAACTATATGCTTCACGGTTTTTCCATATCAAGGGAGCTCTATCATCCTGG
AATCCGGGAATGTTAATGATTATGAAGTAGTGTATCCACAAAAAGTGCCTGCATTGCCCAAAGGAGGAGT
TCAGAATCCTCAGCCAGAGACCAAGTATGAAGATACAATGCAATATGAATTTCACGTGAATGGAGAGCCA
GTCGTCCTTCACTTAGAAAGAAATAAAGGACTTTTTTCAGAAGATTACACTGAAACTCATTATGCCCCTG
ATGGCAGAGAAATTACAACAAGCCCTCCAGTTCAGGATCACTGCTATTATCATGGCTACATTCAGAATGA
AGCTGATTCAAGTGCAGTCATCAGTGCATGCGACGGCTTGAAAGGACATTTCAAGCATCGAGGGGAGACA
TACTTTATTGAGCCCTTGAAGCTTTCCGACAGTGAATCCCATGCAATCTACAAAGATGAAAATGTAGAAA
AAGAGGATGAGATCCCCAAAATCTGTGGGGTAACCCAGACTACTTCGGAGTCAGATGAGCCCATTGAAAA
GATCGCTCAATTAACTAATACTGCTGAAGAAGACAAGTACTTGCGGGTCAAAAAATACATTGAGTTTTAC
GTGGTTGTGGACAACGAAATGTACAAGAATTACAGCAGCAGGAAACCTGCTATAAAAACAAGAGTATATG

AAATGGTCAACCATTTAAACGTGATGTACAGACCTTTGAATTTTTTCATTGCACTGATTGGCCTAGAGAT
TTGGAGCAACAAAGATCAGATTAAGGTTGAACCACAGGTGAATGTCACTGTGAAATCATTTGGAGAATGG
AGAGAGAAAAAATTGCTGCCACGCAAAAGGAATGATAATGCTCAGTTACTCACGGGCATTGACTTCAACG
GAGATACTATAGGATATGCTTACGTCGGCTCCCTCTGCCTTCCGACGCAATCTGTAGCAATTATTCAGAA
TTATCACAACAAAGCAAGCATGGTGGCATCCGTAATGGCCCATGAGTTGGGCCATAATCTTGGCATTCAT
CATGACAAGCCTTCCTGTAATTGCAATTCTGGACGATGCATTATGGCTGCAAGAACAAGTGATGAACCTC
ATTCTGAGTTCAGCTCTTGTAGTGTCCAGGATCATCAGAGGTATCTTCTTAGAGAAAGACCACAATGCAT
TCTCAACAAACCCTTGAGCACGGATATTGTTGCACCCCCAGTTTGTGGAAATTACTTTGTGGAGCGGGGA
GAAGAATGTGACTGTGGCTCTCCTCAGGATTGTCAAAGTGCCTGCTGCAATGCTACAACTTGTAAACTGC
TACATGAGGCACAGTGTGACTCCGGAGAGTGTTGTGAGAAATGCAAATTTAAGAAAGCAGGAGTAGAATG
CCGGGCAGCAAAGGATGACTGTGACTCGCCTGAAAGCTGCACTGGCCAATCTGCTAAGTGTCCCACGGAC
AGTTTCCAGAGGAATGGACATCCATGCCAAAGCAACCAGGGTTACTGCTACAATGGGAAATGCCCCCTCA
TGACAAACCAGTGCATTGCTCTCGGGGGACCAGGTGTAAATGTGTCTCCAGATGGATGTTTTACGTTGAA
CAAGAATGGCCAAGGTTGTAGCTTCTGCAGAATGGAAAATGGTAAAAAGATTCCATGTGCAGCAAAGGAT
GTAAAGTGTGGCATGTTATACTGCGAA

>GAHI01000020 Suta fasciata VEGF-Sut-1 mRNA sequence
ATGAACTTTCTGCTCACTTGGATCCATTGCGGATTGGCGGCGCTGCTTTATTTCCACAACGCCAAGGTGT
TGCAGGCTGCTCCTGCCCAAGAGGATGCAGCGAAGCAACCAGAAGAAGTTATCCCATTTATGACGGTCTT
TGAGCGGAGTGCCTGCAGGCCCATTGAGACCATGGTAGATGTTTACCAGGAGTATCCTGATGAGGTGGAA
TACCTGTTCAAACCATCCTGTGTTCTCCTGATGAAATGTGGAGGATTCTGTAATGACGAAGCGCTAGAAT
GCGTGCCCATAGAGGTGCACAATATCAGCATGGAGATCATGAAACTCAGACACTTGCAGAGCCAGCATAT
ACAACGGATGAGCTTCCAGCAGCACAGTAAATGTCAATGCAGACCAAAGAAACCAATAAGAATTAAACAA
GAAAAAATGCAGAATTACCTCCCCATCTTTCACTATTACCTGCCTGTAGGTGAACAGCCTATAATGATGC
CTTCTGATGGTTCCAGTGGGAATACTCAAAAATAA

>GAHJ00000000.1|GAHJ01000000 TSA: Vermicella annulata, transcriptome shotgun assembly, transcriptome shotgun assembly

>GAHJ01000001 Vermicella annulata 3FTx-Ver-100 mRNA sequence
ATGAAAACTCTGCTGCTGACCTTGGTGGTGGTGACAATCGTGTGCCTGGACTTAGGATACACCCTAAAAT
GTTACAAATCTCACGCTGTTGGGGGTTATGAGGTTTGTAAAGAACACGAGACCAAATGCATTGGATATAA
CATTTGTGCATTCAGAAATCATGCGATAGTCGTCAGGGGATGTGGTACTTCTTGCCCTGAAGGAAATCGT
CCTGTGTGTTGCAGTACAGACATGTGCAACAAGTAG

>GAHJ01000002 Vermicella annulata 3FTx-Ver-70 mRNA sequence
ATGAAAACTCTGCTGCTGACCTTGGTGGTGGTGACAATCGTGTGCCTGGACTTAGGATACACCCTAAAAT
GTTACAAATCTCACGCTCCTGGGGGTTATGAGGTTTGTAAAGAACACGAGACCAAATGCATTGGATATAA
CATTTGTGTATTCAGAAATCGTGCGATAGTCGTCAGGGGATGTGGTGCTTCTTGCCCTACAAAAAATCTT
CCTGTGTGTTGCAGTACAGACATGTGCAACAAGTAG

>GAHJ01000003 Vermicella annulata 3FTx-Ver-49 mRNA sequence
ATGAAAACTCTGCTGCTGACCTTGGTGGTGGTGACAATCGTGTGCCTGGACTTAGGATACACCCTAAAAT
GTTACAAATCTCACGCTCCTGGGGGTTATGAGGTTTGTAAAGAACACGAGACCAAATGCATTGGATATAA
CATTTGTGTATTCAGAAATCGTGCGATAGTCGTCAGGGGATGTGGTGCTTCTTGCCCTACAGGAAATCTT
CCTGTGTGTTGCAGTACAGACATGTGCAACAAGTAG

>GAHJ01000004 Vermicella annulata 3FTx-Ver-47 mRNA sequence
ATGAAAACTCTGCTGCTGACCTTGGTGGTGGTGACAATCGTGTGCCTGGACTTAGGATACACCCTAAAAT
GTTACAACCCTAAGACTGCTGAGGGTTATGAGGTTTGTAAAGAACACGAGACCAAATGCATTGGATATAA
CATTTGTCTATTCAGAAATCATGCGGTAGTCGTCAGGGGATGTGGTGCTTCTTGCCCTACAGAAAATCTT
CCTGTGTGTTGCAGTACAGACATGTGCAACAAGTAG

>GAHJ01000005 Vermicella annulata 3FTx-Ver-124 mRNA sequence
ATGAAAACTCTGCTGCTGACCATGGTGGTGGTGACAATTGTGTGCCTGGACTTAGGATACACCCTTAAAT
GTCATAAATCTCACCTTGATAAGGGTTATGTGGTTTGTAAAGAACACGAGACCATTTGCTTTGAATATAG
AGCATGTATATTCAGAAATGAAGTGTTGTACATCAGGGGATGTAGTGCTTCTTGCCCTGGAGGATATAAG
CATGTGTGTTGCAGTACAGACCTGTGCAACAAGTAG

>GAHJ01000006 Vermicella annulata 3FTx-Ver-128 mRNA sequence
ATGAAAACTCTGCTGCTGACCTTGGTGGTGGTGACAATCGTGTGCCTGGACTTAGGATACACCCTAAGAT
GTAAAAAAACTTATGAGGATTCTGTGGTTTGTAAAGAACACGAGACCATTTGCTATCGATATCCTACTTA
TATATTTAAAAGTAAACTGATAATCCTGAAGGGATGTAGTGCTTCTTGCCCTGACAAAACTAGTGTTCTG
TGTTGCAGTACAGATATGTGCAACACTACAAGTGGCTAA

>GAHJ01000007 Vermicella annulata 3FTx-Ver-130 mRNA sequence
ATGAAAACTCTGCTGCTGACCTTGGTGGTGGTGACAATTGTGTGCCTGGACTTAGGATACACCCTAAAAT
GTCACATAACTGGGAGTCCTAATGATTATGTGGTTTGTAAAGAACACGAGACCGTTTGCTTTCAAAAAAC
TGTTTCTCCATTTAGAGATCGTATGTTAGTCCTTAGGGGATGTAGTACTCCTTGCCCTGAAGGAGATAAT
GCTATGTGTTGCAGTACAGATATGTGCAACAAGTAG

>GAHJ01000008 Vermicella annulata 3FTx-Ver-3 mRNA sequence
ATGAAAACTCTGCTGCTGACCTTGGTGGTGGTGACAATCGTGTGCCTGGACTTCGGATACACCAGGTCAT
GCTTGAAAACACCTGAAATTAAATCTGAGCCTTGTCCACCTGGGCAGAACCTATGCTATACAAAGACTTG
GTGTGATCGTTTTTGTACAATGAGAGGAAAGGTAATCGAACTGGGATGTGCTGCTACTTGCCCTCCAGCA
GAGCCAAGAAAGGATATTACCTGTTGCTCAACAGACAATTGCAACACAGTTCCGTAA

>GAHJ01000009 Vermicella annulata 3FTx-Ver-5 mRNA sequence
ATGAAAACTCTGCTGCTGACCTTGGTGGTGGTGACAATCGTGTGCCTGGACTTAGGTGACAGCTTGATAT
GCTACGTGGGATATAATATCCCTCAGATTTGTCCAACTGGGGAGGTGGTATGCTTTACAAAGACTTGGTG
TGATGCTCACTGTGGCGAAAGAGGAAAACGAGTCGAATTGGGATGTGCTGCTACTTGCCCTATAGTGAAG
CCCGGTATTGATATTACCTGTTGCTCAACAAACAATTGCAACCCATTTCCGTTCTGGCCACGTCCTAGAC
CAGGTCGTTGA

>GAHJ01000010 Vermicella annulata 3FTx-Ver-10 mRNA sequence
ATGAAAACTCTGCTGCTGACCTTGGTGGTGGTGACAATCGTGTGCCTGGACTTCGGTGACAGCTTGGTAT
GCTACCTGGGATATAATACCCCTCGGACTTGTCCACCTGGGCAGAACCTATGCTATACAAAGACTTGGTG
TGATGGTCACTGTGGCAGCAGAGGAAAGCGAATTGTTTTGGGATGTGCTGCTACTTGCCCTATAGTGAAG

CCCGGTGTCGATATTTCCTGTTGCTCAACAGACAAGTGCAACCCACATCCGAAACAGGAACGTCGTTGA

>GAHJ01000011 Vermicella annulata 3FTx-Ver-24 mRNA sequence
ATGAAAACTCTGCTGCTGACCTTGGTGGTGGTGACAATCGTGTGCCTGGACTTAGGATACACCATGACAT
GTTACAACCAACAGTCATCGGAAGCTCAAACCACTACAACTTGTCCAGGTGGGGTGAGCTCTTGCTATAA
AAAGACTTGGAGAGATCACCGTGGAACTATAATTGAAAGGGGATGTGGTTGCCCTGACGTGAAGCCCGGT
GTTGGACGTACATGTTGCCAAGCAGACACATGCAACAATTAG

>GAHJ01000012 Vermicella annulata 3FTx-Ver-32 mRNA sequence
ATGAAAACTCTGCTGCTGACCTTGGTGGTGGTGACAATCGTGTGCCTGGACTTAGGATACACCATGACAT
GTTACAACCAACAGTCATCAGAAGCTCAAACCACTACAACTTGTCCAGGTGGGGTGAGCTCTTGCTATAA
AAAGACTTGGAGAGATCACCGTGGAACTATAATTGAAAGGGGATGTGGTTGCCCTGACGTGAAGCCCGGT
GTTGGACGTACATGTTGCCAAGCAGACACATGCAACAATTAG

>GAHJ01000013 Vermicella annulata 3FTx-Ver-30 mRNA sequence
ATGAAAACTCTGCTGCTGACCTTGGTGGTGGTGACAATCGTGTGCCTGGACTTAGGATACACCATGACAT
GTTACAACCAACAGTCATCGGAAGATCAAACCACTACAACTTGTCCAGGTGGGGTGAGCTCTTGCTATAA
AAAGACTTGGAGAGATCACCGTGGAACTATAATTGAAAGGGGATGTGGTTGCCCTGACGTGAAGCCCGGT
GTTGGACGTACATGTTGCCAAGCAGACAAATGCAACAATTAG

>GAHJ01000014 Vermicella annulata 3FTx-Ver-27 mRNA sequence
ATGAAAACTCTGCTGCTGACCTTGGTGGTGGTGACAATCGTGTGCCTGGACTTAGGATACACCATGACAT
GTTACAACCATCAGTCATCGGAAGCTCAAACCACTACAACTTGTCCAGGTGGGGTGAGCTCTTGCTATAA
AAAGACTTGGAGAGATCACCGTGGAACTATAATTGAAAGGGGATGTGGTTGCCCTGACGTGAAGCCTGGT
GTTGGACGTACATGTTGCCAAGCAGACACATGCAACAATTAG

>GAHJ01000015 Vermicella annulata 3FTx-Ver-28 mRNA sequence
ATGAAAACTCTGCTGCTGACCTTGGTGGTGGTGACAATCGTGTGCCTGGACTTAGGATACACCATGACAT
GTTACAACCATCAGTCATCGGAAGCTCAAACCACTACAAGTTGTCCAGGTGGGGTGAGCTCTTGCTATAA
AAAGACTTGGAGAGATCACCGTGGAACTATAATTGAAAGGGGATGTGGTTGCCCTGACGTGAAGCCCGGT
GTTGGACGTACATGTTGCCAAGCAGACACATGCAACAATTAG

>GAHJ01000016 Vermicella annulata 3FTx-Ver-7 mRNA sequence
ATGAAAACTCTGCTGCTGACCTTGGTGGTGGTGACAATCGTGTGCCTGGACTTAGGTGACAGCGTGATAT
GCTACGTGGGATATAATGCCCATTTGACTTGTCCACCTGGGCAGAACAAATGCTATACAAAGACTTCGTT
TGAAGGTTTTKKTAGCCAGAGAGGACCGCGAGTCGAAATGGGATGTACTGCTACTTGCCCTACAGCGAAG
CCCGGTGTGAAGATTACCTGTTGCTCAACAGACAAGTGCAACCCATTTCCGACACGGCCACGTCCTACAC
CAGGTCATTGA

>GAHJ01000017 Vermicella annulata CRiSP-Ver-3 mRNA sequence
ATGATTGCCTTCATTGTCTTGCTAAGTCTTGCTGCAGTGCTGCAACAGTCTTCTGGAAGTGTTGATTTTG
CTTCTGAGTCAAGTAACAAAAGAGAAATCCAAAAGCAGATTGTTGACAAGCACAATGCTTTAAGGAGATC
AGTGAAACCAACTGCTAGGAACATGTTACAAATGGAATGGAATTCTCGTGCTGCTCAAAATGCACAACGT
TGGGCGCATAGTTGTTCTTTTGATCACAGTCCACCACATACAAGAACTGTGGGAAAACTCAGTTGTGGTG
AAAATATATTCATGTCAAGTCAACCTTTTGCATGGAGTAGAATAATTCAGGCTTGGTATGATGAAAACAA
AGAATTCGTCTATGGCATTGGAGCAAAGCCACCAGGTTCTGTTATTGTCCATTATACCCAGATAGTTTGG
TACAAAAGTCACCTTCTTGGTTGTGCTTCTGCCAGATGTTCTTCATCCAAATACCTCTATGTTTGTCAAT
ACTGCCCAGCAGGGAACATCAGAGGTTCAACTGCTACTCCATATAAATCAGGGCCACCTTGTGGGGACTG
TCCTTCGGCTTGTGTCAACGGACTTTGCACAAATCCTTGCAAATATAAGGATGACTTATCGAACTGCAAA
TCTTTAGCGAGAAACAGTAGATGCCAGACTGAATGGATCAAGTCAAAATGCCCTGCTTCTTGCTTCTGCC
ACACTGAAATAATATAG

>GAHJ01000018 Vermicella annulata KP-Ver-2 mRNA sequence
ATGTCTTCTGGAGGTCTTCTTCTTCTGCTGGGACTCCTCACCCTCTGGGAGGGGCTGACCCCCGTCTCCA
GCAAGGACCGTCCACACTTCTGTCATCTGCCTCATGACACCGGACCATGTAAAGGCATTTTCCAAGCCTT
CTACTACAACCCAGTTCAACATCAATGCCGAGAGTTTATTTATGGTGGATGCGGAGGGAATCCTAACAAT
TTTAAGACCATAGATGAATGCAAACAAACCTGTGCTGCATGA

>GAHJ01000019 Vermicella annulata KP-Ver-3 mRNA sequence
ATGTCTTCTGGAGGTCTTCTTCTTCTGCTGGGACTCCTCACCCTCTGGGAGGGGCTGACCCCCGTCTCCA
GCAAGGACCGTCCACACTTCTGTCATCTGCCTCATGACACCGGACCATGTAAAGGCATTTTCCAAGCCTT
CTACTACAACCCAGTTCAACATCAATGCCGAGAGTTTATTTATGGTGGATGCGGAGGGAATGCTAACAAT
TTTAAGACCATAGATGAATGCAAACAAACCTGTGTTGCATGA

>GAHJ01000020 Vermicella annulata KP-Ver-5 mRNA sequence
ATGTCTTCTGGAGGTCTTCTTCTTCTGCTGGGACTCCTCACCCTCTGGGAGGGGCTGACCCCCGTCTCCA
GCAAGAACCGTCCACACATCTGTCATCTGCCTGCTAACCCCGGATTATGTAATGGCAAGTTCCAAGCCTT
CTACTACAACCGAATTCAACGTTCATGCCTAATGTTTATGTATGGTGGATGCGGAGGGAATGCTAACAAT
TTTAAGACCATAGATGAATGCAAACAAACCTGTGTTGTATGA

>GAHJ01000021 Vermicella annulata KP-Ver-13 mRNA sequence
ATGTCTTCTGGAGGTCTTCTTCTCCTGCTGGGACTCCTCACCCTCTGGGAGGGGCTGACCCCCGTCTCCA
GCAAGGACCGTCCACACTTCTGTCATCTGCCTCATGACACCGGACCATGTAAACGCAACATCCAAGCCTT
CTACTACAACCCAGTTCAACATCAATGCCGAGAGTTTATTTATGGTGGATGCGGAGGGAATCCTAACAAT
TTTAAGACCATAGATGAATGCAAACAAACCTGTGCTGCATGA

>GAHJ01000022 Vermicella annulata LP-Ver-1 mRNA sequence
ATGGGGCGATTCCTCTTTGCGAGCCTCGGCTTGCTGGTCGTGGCTTTCTCCCTAAGTGGAATTGGAGCCA
ATCGCTATTGTCCCTTCGATTGGTTCTCCTACAATATGTCTTGCTACAAGCTCTTCTACTATCCGATGAC
CTGGGACCAAGCACAGAGGTTCTGTGTGGAACAGCAGGAAAACTCCCATCTCGCCTCCATCCATGACGTG
GGAGAGTCGGTGAAGTTGTCCAACTACATCTCCCAAAGATTGAAATTCTTTGACGTTTGGATGGGACTGA
GGCTTTCAAAGAGAAAAGGCATCTGGGAGTGGAGTGATGGCTCCAACCTCACTCACACATCCTGGGAAGA
AGGTGAACCCAACAACCTTTTCAACAAGGAATTCTGTGCTGTGCTGTCAGCCGGAACACGGTATCTCCAA
TGGAACGATAAGAGCTGCATGCACTGGCATAAATTTGTCTGCAAGTTCCGGCCCCGGAGTGAGGCAGGCA
GCTAA

>GAHJ01000023 Vermicella annulata SVMP-Ver-17 mRNA sequence
GATCACTGCTATTATCATGGCTACATTCAGAATGAAGCTGATTCAAGTGCAGTCATCAGTGCATGCAACG
GCTTGAAAGGACATTTCAAGTATCGAGGGGAGACATACTTTATTGAGCCCTTGGAGCTTTCCGACAGTGA
ATCCCATGCAATCTACAAAGATGAAAATGTAGAAAAAGAGGATGAGATCCCCAAAATCTGTGGGGTAACC
CAGACTACTTTGGAGTCAGATGAGCCCATCGAAAAGATCTCTCAATTAACTAATACTCCTCAAGAAGACA

```
ATTACTTGCAGGTCAAAAAATACATTGAGTTTTACGTGGTTGTGGACAACAAAATGTACAAGAAATACAA
CAGCAACAGACATGCTATAAAAAGAAGAGTATATGAAATGGTCAACAGTTTAAATGTGATATACAGACCT
TTGAATTTTTTCATTGCACTGATTGGCCTAGAAATTTGGACCAACCAAGATAAGATTAAGTTTGAACCAA
AGATGAATGTCACTTTGAAATCATTTGGAAAATGGAGAGAGAAAAATTTGCTGCCACGCAAGACGAATGA
TAATGCTCAGTTACTCACTAGCAGTAATTTCATAGAACCTGCTATAGGAGTTGCTTTCACCGGCTCCCTC
TGCAGTCCAAAGCAATCTGTAGCAGTTATTCGGGATCATAGCAACAGAGCAATCATGGTGGCATCTACAA
TGGCCCATGAGTTGGGTCATAATCTGGGCATTCATCATGACAGAGGTTTCTGTAATTGCCATGCTGGACC
ATGCATTATGACTAAGAGAATAAGTAATGAACCTCATTCTGAGTTCAGCAATTGTAGTGTCCAGGAACAT
CAGAGGTATCTTCTTACTTACAGACCACAATGCATTCTCAACAAACCCTTGAGCACAGATATTGTTGCAC
CTCCAATTTGTGGAAATTACTTTGTGGAGGTGGGAGAAGAATGTGACTGTGGCTCTCCTCAGGATTGTCA
AAATGCCTGCTGCAACGCCAAAACTTGCAAACTGCAATACGACTGTGACTCCGGAGAGTGTTGTGACCAA
TGCAAATTTAAGAAAGCAGGAGCAGAATGCCGGGCAGCAAAGGATGACTGTGACTTGCCTGAAATCTGCA
CTGGCCAATCTGCTGAGTGTCCCATGGACAGCTTCCAGAGGAATGGACATCCATGCCAAAACAACCAAGG
TTACTGCTACAATGGGACATGCCCCCTTATGACAAACCAGTGTATAGCTCTAATGGGGCCAGGTGTAAAT
GTGTCCCCAGATGAATGTTTTACGTTGAAACAGAATGACCCAGATTGTGGCTTCTGCAGAATAGAAAATG
GTAAAAAGATTCCATGTGCAGAAAAGGATAAAATGTGTGGCATGTTAGTATGCGAAAAGGGAAACGCGAC
ATGCATCTGCTTTACTACAACAGATGATCCAGATTATGCAATGGTTGAACCTGGAACAAAATGTGGAGAT
GGAATGGTGTGCAGCAACAGGCAGTGTGTTGATGTGCAGACAGCCTACTGA

>GAHJ01000024 Vermicella annulata WP-Ver-1 mRNA sequence
ATGTTAAAACTGCTAAAGAGGATAGAGATGATGAAATTAGCCAAGCKGATRAAWGAGAAAATCGGGTCCT
GCCCAGATGTGGACAGGCCCATCCCACCTCTGGGTATTTGCAGAACCATGTGTCAAACAGATTCAAACTG
TCCAAATATCAAGAAGTGCTGCAAAAATGGCTGCGGGTTCATGACTTGTTCAACGCCCAAAGCCTGA
```
