# Supplementary material for: Venom Down Under: Dynamic Evolution of Australian Elapid Snake Toxins
Source: Toxins (Basel). 2013 Dec 18;5(12):2621–55. doi: 10.3390/toxins5122621 (PMC3873703; doi:10.3390/toxins5122621)
Supplement: Supplementary File 1 — Supplementary (ZIP, 287 KB) [file toxins-05-02621-s001.zip › Supplementary Table 1 - Molecular evolution of Australian elapid 3FTx-3.pdf]

Supplementary Table 1. Molecular evolution of Australian elapid 3FTx

		SLAC[a]	FEL[b]	REL[c]	FUBAR[d]	Integrative analyses	MEME Sites[e]	BSR[f]	PAML[g]	
						SLAC + FEL + REL + MEME + FUBAR			M8	M2a
Acanthophis wellsi										
	ω>1[h]	0	0	17	8	17			8	3
**Type II**	ω<1[i]	0	2	0	1	2	2	2	(3+5)	(2+1)
	ω=	1.47	–	dN>>dS	–	–			1.76	1.76
Brachyurophis roperi										
	ω>1[h]	0	0	10	12	12			25	25
**Type I**	ω<1[i]	0	1	1	1	1	0	2	(13+12)	(12+13)
	ω=	14.12	–	dN>>dS	–	–			18.95	18.95
	ω>1[h]	0	0	2	12	13			17	16
**Type II**	ω<1[i]	0	2	3	2	4	2	2	(13+4)	(11+5)
	ω=	2.42	–	dN>>dS	–	–			3.52	3.54
Cacophis squamulosus										
	ω>1[h]	0	0	2	1				3	3
**Type I**	ω<1[i]	0	1	3	1			1	(0+3)	(0+3)
	ω=	1.20	–	dN>>dS	–	–			2.51	2.50
Drysdalia coronoides										
	ω>1[h]	0	0	1	2	3			10	10
**Type I**	ω<1[i]	0	0	0	0	0	0	2	(6+4)	(6+4)
	ω=	1.21	–	1.38	–	–			4.45	4.46
	ω>1[h]	0		25	9	25			8	7
**Type II**	ω<1[i]	0		0	1	2	3	5	(6+2)	(6+1)
	ω=	2.97	–	dN>>dS	–	–			2.79	2.37
Echiopsis curta										
	ω>1[h]	0	0	2	7	8			6	3
**Type II**	ω<1[i]	0	2	0	2	2	0	3	(2+4)	(1+2)
	ω=	4.92	–	dN>>dS	–	–			2.43	2.43
Furina ornata										
	ω>1[h]	0	0	5	3	5			6	6
**Type II**	ω<1[i]	2	1	0	1	2	0	0	(5+1)	(3+3)
	ω=	1.16	–	3.07	–	–			1.63	1.67
	ω>1[h]	0	0	4	5	6			9	7
**Type III**	ω<1[i]	0	1	0	1	1	2	3	(3+6)	(1+6)
	ω=	1.28	–	20.7	–	–			1.81	1.82

		SLAC[a]	FEL[b]	REL[c]	FUBAR[d]	Integrative analyses	MEME Sites[e]	BSR[f]	PAML[g]	
Hemiaspis signata										
	ω>1[h]	0	0	3	2	3			0	0
**Type I**	ω<1[i]	0	1	0	4	4	0	0		
	ω=	1.19	–	1.33	–	–			1.18	1.18
	ω>1[h]	0	0	3	12	14			14	13
**Type II**	ω<1[i]	0	2	4	2	4	2	5	(10+4)	(9+4)
	ω=	2.74	–	dN>>dS	–	–			4.26	4.26
Parasuta nigriceps										
	ω>1[h]	0	0	5	2	5			5	3
**Type I**	ω<1[i]	0	2	0	1	2	0	1	(1+4)	(1+2)
	ω=	1.78	–	dN>>dS	–	–			3.0	3.0
	ω>1[h]	0	0	1	2	3			8	5
**Type II**	ω<1[i]	0	3	0	2	3	1	1	(2+6)	(2+3)
	ω=	1.27	–	2.59	–	–			5.97	5.97
Pseudonaja modesta										
	ω>1[h]	0	4	0	14	15			18	13
**Type II**	ω<1[i]	2	3	6	21	7	3	6	(13+5)	(11+2)
	ω=	2.65	–	dN>>dS	–	–			3.28	2.73
	ω>1[h]	2	10	19	25	27			25	22
**Type III**	ω<1[i]	2	4	24	3	24	16	10	(22+3)	(18+4)
	ω=	2.69	–	11.22	–	–			2.82	2.78
Suta nigriceps										
	ω>1[h]	0	0	5	2	5			5	3
**Type I**	ω<1[i]	0	2	0	1	2	0	1	(1+4)	(1+2)
	ω=	1.78	–	dN>>dS	–	–			3.0	3.0
	ω>1[h]	0	0	1	2	3			8	5
**Type II**	ω<1[i]	0	3	0	2	3	1	1	(2+6)	(2+3)
	ω=	1.27	–	2.59	–	–			5.97	5.97
Vermicella annulata										
	ω>1[h]	0	0	0	0	1			0	0
**Type I**	ω<1[i]	0	1	1	1	4	1	1		
	ω=	1.05	–	16.17	–	–			0.63	0.63
	ω>1[h]	0	0	6	5	9			14	11
**Type II**	ω<1[i]	0	1	0	2	2	1	3	(6+8)	(5+6)
	ω=	1.66	–	2.99	–	–			3.89	3.89
	ω>1[h]	0	3	4	13	13			20	19
**Type III**	ω<1[i]	0	2	2	3	3	3	7	(18+2)	(14+5)
	ω=	3.02	–	dN>>dS	–	–			6.84	5.66

Legend:

a: Single Likelihood Ancestor Counting

b: Fixed-effects likelihood

c: Random-effects likelihood

d: Fast Unconstrained Bayesian AppRoximation

e: Sites detected as experiencing episodic diversifying selection (0.05 significance) by the Mixed Effects Model Evolution

f: #of branches detected as episodically diversifying by Branch-site REL

g: Positively-selected sites detected using the Bayes Empirical Bayes approach implemented in M8 and M2a. Sites detected at 0.99 and 0.95 significance are indicated in the parenthesis

h: Number of positively selected sites at 0.05 significance (for SLAC, FEL) or 50 Bayes factor (for REL) / number of sites under pervasive diversifying selection at the posterior probability ≥0.9 (FUBAR)

i: Number of negatively selected sites at 0.05 significance (for SLAC, FEL) or 50 Bayes factor (for REL) / number of sites under pervasive purifying selection at the posterior probability ≥0.9 (FUBAR)

dN>>dS: Extremely large dN, as dS is nearly 0

ω**:** mean dN/dS
