# Supplementary material for: Venom Down Under: Dynamic Evolution of Australian Elapid Snake Toxins
Source: Toxins (Basel). 2013 Dec 18;5(12):2621–55. doi: 10.3390/toxins5122621 (PMC3873703; doi:10.3390/toxins5122621)
Supplement: Supplementary File 1 — Supplementary (ZIP, 287 KB) [file toxins-05-02621-s001.zip › Supplementary Tables 2 and 3.pdf]

Supplementary Table 2

Species	# of sequences	total # of bases	average sequence length	average sequence quality	% GC
Acanthophis wellsi	55,372	15,197,836	274	30.71	50.34
Brachyurophis roperi	55,694	15,892,775	285	30.42	45.89
Cacophis squamulosus	38,985	14,068,260	360	32.21	48.73
Denisonia devisii	28,275	8,059,988	285	30.52	49.75
Echiopsis curta	59,740	17,191,872	287	30.40	49.76
Furina ornate	46,190	13,117,708	283	30.39	49.19
Hemiaspis signata	48,904	18,095,514	370	32.13	57.09
Hoplocephalus bungaroides	47,675	13,050,675	273	32.37	56.52
Pseudonaja modesta	51,984	14,959,557	287	30.51	48.99
Suta fasciata	90,963	26,698,687	293	30.35	47.26
Vermicella annulata	49,960	14,653,238	293	32.71	55.39

Supplementary Table 3

Species	# of contigs	Assembled bases	Average bases per contig
Acanthophis wellsi	2,657	1,284,940	484
Brachyurophis semifasciata	5,493	2,794,630	509
Denisonia devisi	1,572	774,029	492
Echiopsis curta	2,998	1,534,906	512
Furina ornata	3,137	1,555,634	496
Cacophis squamulosus	3,015	1,638,379	543
Hemiaspis signata	1,431	728,481	509
Hoplocephalus bungaroides	3,939	2,150,604	546
Pseudonaja modesta	3,335	1,708,478	512
Suta fasciata	6,180	3,391,483	549
Vermicella annulata	3,711	1,842,636	497
