# Supplementary material for: Venom Down Under: Dynamic Evolution of Australian Elapid Snake Toxins
Source: Toxins (Basel). 2013 Dec 18;5(12):2621–55. doi: 10.3390/toxins5122621 (PMC3873703; doi:10.3390/toxins5122621)
Supplement: Supplementary File 1 — Supplementary (ZIP, 287 KB) [file toxins-05-02621-s001.zip › Supplementary File 1 assembly parameters.pdf]

```
Parameter settings seen for:

Sanger data (also common parameters), 454 data


Used parameter settings:

  General (-GE):

      Project name in (proin)                     : mira

      Project name out (proout)                   : mira

      Number of threads (not)                     : 2

      Automatic memory management (amm)           : yes

          Keep percent memory free (kpmf)         : 15

          Max. process size (mps)                 : 0

      EST SNP pipeline step (esps)                : 0

      Use template information (uti)              :  [san]  yes

                                                     [454]  yes

          Template insert size minimum (tismin)   :  [san]  -1

                                                     [454]  -1

          Template insert size maximum (tismax)   :  [san]  -1

                                                     [454]  -1

          Template partner build direction (tpbd) :  [san]  -1

                                                     [454]  -1

      Colour reads by hash frequency (crhf)       : no


  Load reads options (-LR):

      Load sequence data (lsd)                    :  [san]  no

                                                     [454]  yes

          File type (ft)                          :  [san]  fasta

                                                     [454]  fastq
```

```
        External quality (eq)                    : from SCF (scf)
            Ext. qual. override (eqo)            : no
            Discard reads on e.q. error (droeqe): no
        Solexa scores in qual file (ssiqf)       : no
        FASTQ qual offset (fqqo)                 :  [san]  0
                                                    [454]  0

    Wants quality file (wqf)                     :  [san]  yes
                                                    [454]  yes

    Read naming scheme (rns)                     :  [san] Sanger
Institute (sanger)
                                                    [454]
forward/reverse (fr)

    Merge with XML trace info (mxti)             :  [san]  no
                                                    [454]  yes

    Filecheck only (fo)                          : no

  Assembly options (-AS):
    Number of passes (nop)                       : 5
        Skim each pass (sep)                     : yes
    Maximum number of RMB break loops (rbl)      : 3
    Maximum contigs per pass (mcpp)              : 0

    Minimum read length (mrl)                    :  [san]  80
                                                    [454]  40
```

```
Minimum reads per contig (mrpc)             :  [san]  2
                                               [454]  1
Base default quality (bdq)                  :  [san]  10
                                               [454]  10
Enforce presence of qualities (epoq)        :  [san]  yes
                                               [454]  yes

Automatic repeat detection (ard)            : no
    Coverage threshold (ardct)              :  [san]  2
                                               [454]  2
    Minimum length (ardml)                  :  [san]  400
                                               [454]  200
    Grace length (ardgl)                    :  [san]  40
                                               [454]  20
    Use uniform read distribution (urd)     : no
      Start in pass (urdsip)                : 4
      Cutoff multiplier (urdcm)             :  [san]  1.5
                                               [454]  1.5
Keep long repeats separated (klrs)          : no

Spoiler detection (sd)                      : no
    Last pass only (sdlpo)                  : yes

Use genomic pathfinder (ugpf)               : no

Use emergency search stop (uess)            : yes
    ESS partner depth (esspd)               : 500
```

```
    Use emergency blacklist (uebl)              : yes
    Use max. contig build time (umcbt)          : yes
        Build time in seconds (bts)             : 3600

Strain and backbone options (-SB):
    Load straindata (lsd)                       : no
    Assign default strain (ads)                 :  [san]  no
                                                   [454]  no
        Default strain name (dsn)               :  [san]  StrainX
                                                   [454]  StrainX
    Load backbone (lb)                          : no
        Start backbone usage in pass (sbuip)    : 3
        Backbone file type (bft)                : fasta
        Backbone base quality (bbq)             : 30
        Backbone strain name (bsn)              : ReferenceStrain
            Force for all (bsnffa)              : no
        Backbone rail from strain (brfs)        :
        Backbone rail length (brl)              : 0
        Backbone rail overlap (bro)             : 0
        Also build new contigs (abnc)           : yes

Dataprocessing options (-DP):
    Use read extensions (ure)                   :  [san]  yes
                                                   [454]  no
        Read extension window length (rewl)     :  [san]  30
                                                   [454]  15
        Read extension w. maxerrors (rewme)     :  [san]  2
```

```
                                               [454]  2
        First extension in pass (feip)         :  [san]  0
                                               [454]  0
        Last extension in pass (leip)          :  [san]  0
                                               [454]  0


Clipping options (-CL):
    Merge with SSAHA2/SMALT vector screen (msvs):  [san]  no
                                               [454]  no
        Gap size (msvsgs)                      :  [san]  10
                                               [454]  8
        Max front gap (msvsmfg)                :  [san]  60
                                               [454]  8
        Max end gap (msvsmeg)                  :  [san]  120
                                               [454]  12
        Strict front clip (msvssfc)            :  [san]  0
                                               [454]  0
        Strict end clip (msvssec)              :  [san]  0
                                               [454]  0
    Possible vector leftover clip (pvlc)       :  [san]  yes
                                               [454]  no
        maximum len allowed (pvcmla)           :  [san]  18
                                               [454]  18
    Min qual. threshold for entire read (mqtfer):  [san]  0
                                               [454]  0
        Number of bases (mqtfernob)            :  [san]  0
                                               [454]  0
```

```
Quality clip (qc)                          :  [san]  no
                                              [454]  no
    Minimum quality (qcmq)                 :  [san]  20
                                              [454]  20
    Window length (qcwl)                   :  [san]  30
                                              [454]  30
Bad stretch quality clip (bsqc)            :  [san]  yes
                                              [454]  no
    Minimum quality (bsqcmq)               :  [san]  20
                                              [454]  5
    Window length (bsqcwl)                 :  [san]  30
                                              [454]  20
Masked bases clip (mbc)                    :  [san]  yes
                                              [454]  yes
    Gap size (mbcgs)                       :  [san]  20
                                              [454]  5
    Max front gap (mbcmfg)                 :  [san]  40
                                              [454]  12
    Max end gap (mbcmeg)                   :  [san]  60
                                              [454]  12
Lower case clip (lcc)                      :  [san]  no
                                              [454]  yes
Clip poly A/T at ends (cpat)               :  [san]  no
                                              [454]  yes
    Keep poly-a signal (cpkps)             :  [san]  no
                                              [454]  no
    Minimum signal length (cpmsl)          :  [san]  12
```

```
                                                [454]  12
    Max errors allowed (cpmea)              :   [san]  1
                                                [454]  1
    Max gap from ends (cpmgfe)              :   [san]  9
                                                [454]  20000
Clip 3 prime polybase (c3pp)                :   [san]  no
                                                [454]  no
    Minimum signal length (c3ppmsl)         :   [san]  12
                                                [454]  12
    Max errors allowed (c3ppmea)            :   [san]  2
                                                [454]  2
    Max gap from ends (c3ppmgfe)            :   [san]  9
                                                [454]  9
Clip known adaptors right (ckar)            :   [san]  no
                                                [454]  yes
Ensure minimum left clip (emlc)             :   [san]  yes
                                                [454]  no
    Minimum left clip req. (mlcr)           :   [san]  25
                                                [454]  4
    Set minimum left clip to (smlc)         :   [san]  30
                                                [454]  4
Ensure minimum right clip (emrc)            :   [san]  no
                                                [454]  no
    Minimum right clip req. (mrcr)          :   [san]  10
                                                [454]  10
    Set minimum right clip to (smrc)        :   [san]  20
                                                [454]  15
```

```
    Apply SKIM chimera detection clip (ascdc)   : no
    Apply SKIM junk detection clip (asjdc)      : no

    Propose end clips (pec)                     : no
        Bases per hash (pecbph)                 : 17
        Handle Solexa GGCxG problem (pechsgp)   : yes

    Clip bad solexa ends (cbse)                 : yes

Parameters for SKIM algorithm (-SK):
    Number of threads (not)                     : 2

    Also compute reverse complements (acrc)     : yes
    Bases per hash (bph)                        : 21
    Hash save stepping (hss)                    : 1
    Percent required (pr)                       :  [san]  70
                                                   [454]  80

    Max hits per read (mhpr)                    : 30
    Max megahub ratio (mmhr)                    : 0

    SW check on backbones (swcob)               : no

    Freq. est. min normal (fenn)                : 0.4
    Freq. est. max normal (fexn)                : 1.6
    Freq. est. repeat (fer)                     : 1.9
```

```
    Freq. est. heavy repeat (fehr)            : 8
    Freq. est. crazy (fecr)                   : 20
    Mask nasty repeats (mnr)                  : yes
        Nasty repeat ratio (nrr)              : 100
    Repeat level in info file (rliif)         : 6

    Max hashes in memory (mhim)               : 15000000
    MemCap: hit reduction (mchr)              : 2048

Pathfinder options (-PF):
    Use quick rule (uqr)                      :  [san]  yes
                                                 [454]  yes
        Quick rule min len 1 (qrml1)          :  [san]  200
                                                 [454]  80
        Quick rule min sim 1 (qrms1)          :  [san]  90
                                                 [454]  90
        Quick rule min len 2 (qrml2)          :  [san]  100
                                                 [454]  60
        Quick rule min sim 2 (qrms2)          :  [san]  95
                                                 [454]  95
    Backbone quick overlap min len (bqoml)    :  [san]  150
                                                 [454]  80
    Max. start cache fill time (mscft)        : 5

Align parameters for Smith-Waterman align (-AL):
    Bandwidth in percent (bip)           :  [san]  20
                                            [454]  20
```

```
      Bandwidth max (bmax)                  :  [san]  130
                                               [454]  80
      Bandwidth min (bmin)                  :  [san]  25
                                               [454]  20
      Minimum score (ms)                    :  [san]  30
                                               [454]  15
      Minimum overlap (mo)                  :  [san]  17
                                               [454]  20
      Minimum relative score in % (mrs)     :  [san]  70
                                               [454]  80
      Solexa_hack_max_errors (shme)         :  [san]  0
                                               [454]  0
      Extra gap penalty (egp)               :  [san]  no
                                               [454]  yes
          extra gap penalty level (egpl)    :  [san] low
                                               [454] reject_codongaps
          Max. egp in percent (megpp)       :  [san]  100
                                               [454]  100


  Contig parameters (-CO):
      Name prefix (np)                                        : mira
      Reject on drop in relative alignment score in %
(rodirs) :  [san]  25
                                                                [454]
15
      Mark repeats (mr)                                       : yes
          Only in result (mroir)                              : no
          Assume SNP instead of repeats (asir)                : no
```

Minimum reads per group needed for tagging
(mrpg)   : [san] 2

[454]
4

Minimum neighbour quality needed for tagging
(mnq)   : [san] 20

[454]
20

Minimum Group Quality needed for RMB Tagging
(mgqrt) : [san] 30

[454] 2
5

End-read Marking Exclusion Area in bases
(emea)   : [san] 25

[454]
10

Set to 1 on clipping PEC (emeas1clpec)   : yes

Also mark gap bases
(amgb)   : [san] yes

[454]
no

Also mark gap bases - even multicolumn
(amgbemc) : [san] yes

[454]
yes

Also mark gap bases - need both strands
(amgbnbs): [san] yes

[454]
yes

Force non-IUPAC consensus per sequencing type
(fnicpst) : [san] no

[454]
no

Merge short reads
(msr)   : [san] no

[454]
no

```
          Keep ends unmerged
(msrkeu)                          :  [san]  -1

                                                                    [454]
-1

      Gap override ratio
(gor)                                 :  [san]  66

                                                                    [454]
66


  Edit options (-ED):

      Automatic contig editing (ace)              :  [san]  no

                                                     [454]  yes

     Sanger only:

      Strict editing mode (sem)                   : no

      Confirmation threshold in percent (ct)      : 50


  Misc (-MI):

      Stop on NFS (sonfs)                         : yes

      Extended log (el)                           : no

      Large contig size (lcs)                     : 500

      Large contig size for stats(lcs4s)          : 1000

      Stop on max read name length (somrnl)       : 40


  Directories (-DI):

      Working directory                 :

      When loading EXP files            :

      When loading SCF files            :

      Top directory for writing files   : mira_assembly

      For writing result files          : mira_assembly/mira_d_results
```

```
      For writing result info files     : mira_assembly/mira_d_info

      For writing tmp files             : /tmp//mira_d_tmp

      Tmp redirected to (trt)           : /tmp/

      For writing checkpoint files      : mira_assembly/mira_d_chkpt


  File names (-FN):

      When loading sequences from
FASTA            :  [san]  mira_in.sanger.fasta

                                                      [454]  mira_in.454.
fasta

      When loading qualities from FASTA
quality    :  [san]  mira_in.sanger.fasta.qual

                                                      [454]  mira_in.454.
fasta.qual

      When loading sequences from
FASTQ            :  [san]  mira_in.sanger.fastq

                                                      [454]  mira_in.454.
fastq

      When loading project from CAF                : mira_in.sanger.caf

      When loading project from MAF (disabled)     : mira_in.sanger.maf

      When loading EXP fofn                        : mira_in.sanger.fofn

      When loading project from PHD                : mira_in.phd.1

      When loading strain data                     :
mira_straindata_in.txt

      When loading XML trace info
files            :  [san]  mira_traceinfo_in.sanger.xml

                                                      [454]  mira_tracein
fo_in.454.xml

      When loading SSAHA2 vector screen results    :
mira_ssaha2vectorscreen_in.txt

      When loading SMALT vector screen results     :
mira_smaltvectorscreen_in.txt
```

```
      When loading backbone from MAF              : mira_backbone_in.maf

      When loading backbone from CAF              : mira_backbone_in.caf

      When loading backbone from GenBank          : mira_backbone_in.gbf

      When loading backbone from GFF3             :
mira_backbone_in.gff3

      When loading backbone from FASTA            :
mira_backbone_in.fasta



  Output files (-OUTPUT/-OUT):

      Save simple singlets in project (sssip)     :  [san]  no

                                                     [454]  no

      Save tagged singlets in project (stsip)     :  [san]  yes

                                                     [454]  yes


      Remove rollover tmps (rrot)                 : yes

      Remove tmp directory (rtd)                  : no


    Result files:

      Saved as CAF                      (orc)     : yes

      Saved as MAF                      (orm)     : yes

      Saved as FASTA                    (orf)     : yes

      Saved as GAP4 (directed assembly)  (org)     : no

      Saved as phrap ACE                (ora)     : yes

      Saved as GFF3                    (org3)     : no

      Saved as HTML                     (orh)     : no

      Saved as Transposed Contig Summary (ors)     : yes

      Saved as simple text format       (ort)     : no
```

```
  Saved as wiggle                    (orw)     : no

Temporary result files:
  Saved as CAF                       (otc)     : yes
  Saved as MAF                       (otm)     : no
  Saved as FASTA                     (otf)     : no
  Saved as GAP4 (directed assembly)  (otg)     : no
  Saved as phrap ACE                 (ota)     : no
  Saved as HTML                      (oth)     : no
  Saved as Transposed Contig Summary (ots)     : no
  Saved as simple text format        (ott)     : no

Extended temporary result files:
  Saved as CAF                      (oetc)     : no
  Saved as FASTA                    (oetf)     : no
  Saved as GAP4 (directed assembly) (oetg)     : no
  Saved as phrap ACE                (oeta)     : no
  Saved as HTML                     (oeth)     : no
  Save also singlets               (oetas)     : no

Alignment output customisation:
  TEXT characters per line (tcpl)              : 60
  HTML characters per line (hcpl)              : 60
  TEXT end gap fill character (tegfc)          :
  HTML end gap fill character (hegfc)          :

File / directory output names:
```

```
CAF               : mira_out.caf

MAF               : mira_out.maf

FASTA             : mira_out.unpadded.fasta

FASTA quality     : mira_out.unpadded.fasta.qual

FASTA (padded)    : mira_out.padded.fasta

FASTA qual.(pad): mira_out.padded.fasta.qual

GAP4 (directory): mira_out.gap4da

ACE               : mira_out.ace

HTML              : mira_out.html

Simple text       : mira_out.txt

TCS overview      : mira_out.tcs

Wiggle            : mira_out.wig
```

SK: pr=80-

AL mrs=80

Egp=1

AS:mrpc=1
